# Asymmetric hydrogenation for the synthesis of 2-substituted chiral morpholines†

**DOI:** 10.1039/d1sc04288b

**Published:** 2021-10-28

**Authors:** Mingxu Li, Jian Zhang, Yashi Zou, Fengfan Zhou, Zhenfeng Zhang, Wanbin Zhang

**Affiliations:** Shanghai Key Laboratory for Molecular Engineering of Chiral Drugs, School of Pharmacy, Shanghai Jiao Tong University 800 Dongchuan Road Shanghai 200240 China zhenfeng@sjtu.edu.cn; Frontier Science Center for Transformative Molecules, School of Chemistry and Chemical Engineering, Shanghai Jiao Tong University 800 Dongchuan Road Shanghai 200240 China

## Abstract

Asymmetric hydrogenation of unsaturated morpholines has been developed by using a bisphosphine-rhodium catalyst bearing a large bite angle. With this approach, a variety of 2-substituted chiral morpholines could be obtained in quantitative yields and with excellent enantioselectivities (up to 99% ee). The hydrogenated products could be transformed into key intermediates for bioactive compounds.

## Abstract 2

2-Substituted chiral morpholines were synthesized *via* a newly developed asymmetric hydrogenation of dehydromorpholines catalyzed by a bisphosphine–rhodium complex bearing a large bite angle.

## Graphical abstract

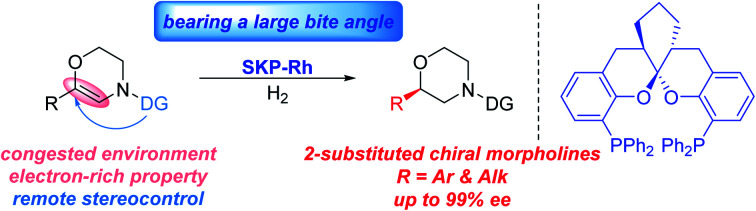

## Introduction

**Fig. 1:**
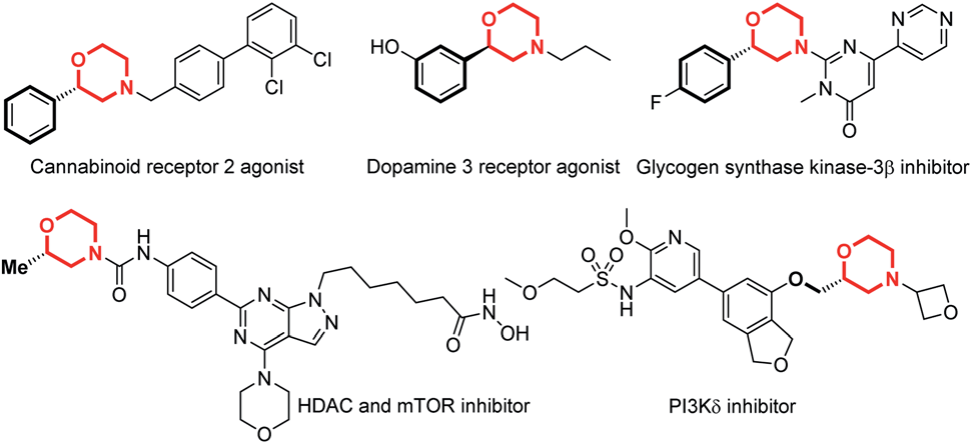
2-Substituted chiral morpholines as bioactive compounds.

**Fig. 2:**
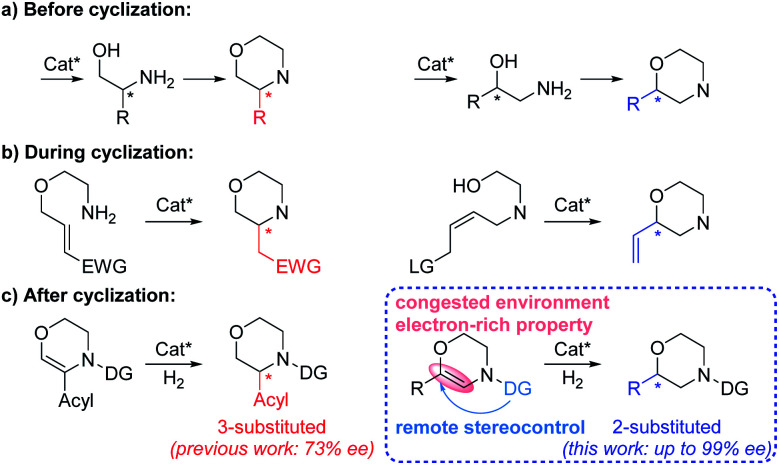
Asymmetric synthesis of 2- and 3-substituted chiral morpholines. (a) Form the stereocenter before cyclization; (b) form the stereocenter during cyclization; (c) form the stereocenter after cyclization.

Chiral N-heterocycles are widely used structural motifs present in a large number of valuable drug candidates and other bioactive compounds, in which chiral morpholine is one of the most important and attractive members (Fig. 1).^1^ Therefore it is no surprise that a variety of asymmetric synthetic approaches to afford chiral morpholines have been reported (Fig. 2).^2–5^ However, most of these methodologies require stoichiometric chiral starting materials or reagents,^2^ while relatively efficient and economical asymmetric catalytic methods have not been well studied.^3–5^ There are a few indirect catalytic examples which form the 2-stereocenter (the stereocenter adjacent to the O-atom) or 3-stereocenter (the stereocenter adjacent to the N-atom) before the cyclization of the morpholine ring (Fig. 2a).^3^ Other methods have been reported for the construction of the 2- or 3-stereocenter during the cyclization of the morpholine ring, such as the preparation of chiral 3-acylmethyl morpholines *via* organocatalyzed intramolecular aza-Michael addition and chiral 2-vinyl morpholines *via* metal-catalyzed allylic substitution (Fig. 2b).^4^ Furthermore, there are only a sporadic number of reports concerning the generation of the 3-stereocenter *via* enantioselective addition of the C=C or C=N bond after the cyclization of the unsaturated morpholine ring (Fig. 2c).^5^ No examples concerning the production of 2-substituted chiral morpholines *via* this way have been reported so far. Therefore, an efficient and universal catalytic method is highly desired to acquire such chiral morpholines, especially 2-substituted chiral compounds.

The transition-metal-catalyzed asymmetric hydrogenation reaction is indisputably one of the most powerful methods for the acquisition of chiral molecules owing to its high efficiency, simple operation, and atom economy.^6^ It has been widely used as an “after cyclization” method for the efficient synthesis of various chiral N-heterocyclic compounds such as (benzo)piperidines and pyrrolidines.^7^ However, to the best of our knowledge, there are only two literature examples concerning asymmetric hydrogenation for the synthesis of 3-substituted chiral morpholines, and only 73% ee was obtained for the endocyclic alkenyl substrates.^5*e*,*f*,8^ In addition, an electron-withdrawing acyl substituent on the alkenyl was always required for the α-branched dehydromorpholines utilized in the asymmetric hydrogenation (Fig. 2c, left). Compared with 3-substituted chiral morpholines, asymmetric hydrogenation for the preparation of 2-substituted chiral morpholines with the stereocenter adjacent to the O-atom is considered to be more challenging and has not been reported at all. The main difficulty lies in the congested environment and electron-rich properties of the dehydromorpholine substrates, which results in very low reactivity. Introducing an *N*-acyl directing group is a universal strategy for the activation of enamine substrates.^9^ However, this is still insufficient and would bring about another difficulty to the untouched β-branched dehydromorpholines due to the challenge in the remote stereocontrol (Fig. 2c, right). Therefore, an efficient strategy needs to be developed and adopted to address the above problems.

In continuation of our long-term pursuit of highly efficient asymmetric catalytic hydrogenations,^10^ we have developed a weak interaction-promoted strategy for substrate activation and stereocontrol.^10*j*,*l*–*p*^ In addition, we have realized highly efficient remote stereocontrol in the asymmetric hydrogenation of acyclic β-branched enamides/enol esters and γ-branched allylic amides by using a bisphosphine-Rh catalyst bearing a large bite angle.^10*h*,*i*,*s*^ Therefore, we envisaged that the first efficient asymmetric hydrogenation of 2-substituted dehydromorpholines could be realized by using a similar catalytic system and control strategy. The approach is also expected to be applied to the asymmetric hydrogenation of other challenging substrates for the efficient synthesis of related chiral N-heterocycles.

## Results and discussion

Based on the above assumptions, 6-phenyl-3,4-dihydro-2*H*-1,4-oxazines with different *N*-substituents (**1a-R**) were tested in the hydrogenation (Table 1, entries 1–5). The complex of (*R*,*R*,*R*)-**SKP** with [Rh(cod)_2_]SbF_6_, which has been successfully applied in the asymmetric hydrogenation of β-branched enol esters and γ-branched enamides in our previous studies,^10*h*,*s*,11^ was chosen as the catalyst. Dichloromethane (DCM), which is thought to have little coordinating ability, was chosen as the solvent. It was found that the substrate **1a** bearing a *N*-Cbz group gave superior enantioselectivity compared to its analogue **1a-NO2** (entry 1 *vs.* 2). Other carbamate-substituted substrates **1a-COOiBu**, and **1a-Boc** also showed high reactivity but relatively lower enantioselectivity (entries 3–4). Changing the *N*-substituent to a Ts group failed to yield any product (entry 5). The dehydromorpholine **1a** was then chosen as the model substrate for further condition optimization. Firstly, several representative chiral diphosphine ligands were evaluated under 50 atm hydrogen pressure at room temperature. Several classic diphosphine ligands possessing large bite angles, including **SDP**, **f-Binaphane** and **JosiPhos** gave positive results (entries 6–8), while others including **DTBM-SegPhos**, **Me-DuPhos**, **Ph-BPE**, and QuinoxP* showed no reactivities (entries 9–12). Secondly, some commonly used solvents were screened using the rhodium complex of (*R*,*R*,*R*)-**SKP** as catalyst (entries 13–18). Only the aprotic and less polar solvents AcOEt and toluene provided moderate conversions (entries 13–14), while dichloroethane (DCE), MeOH, tetrahydrofuran (THF) and 1,4-dioxane possessing certain coordinating abilities resulted in almost no reaction (entries 15–18). Finally, decreasing the hydrogen pressure reduces the reactivity (entries 19–21). Quantitative conversion with identical enantioselectivity could be obtained under 30 atm hydrogen pressure when the reaction time was prolonged to 24 hours (entry 20).

**Table 1:** Condition optimization

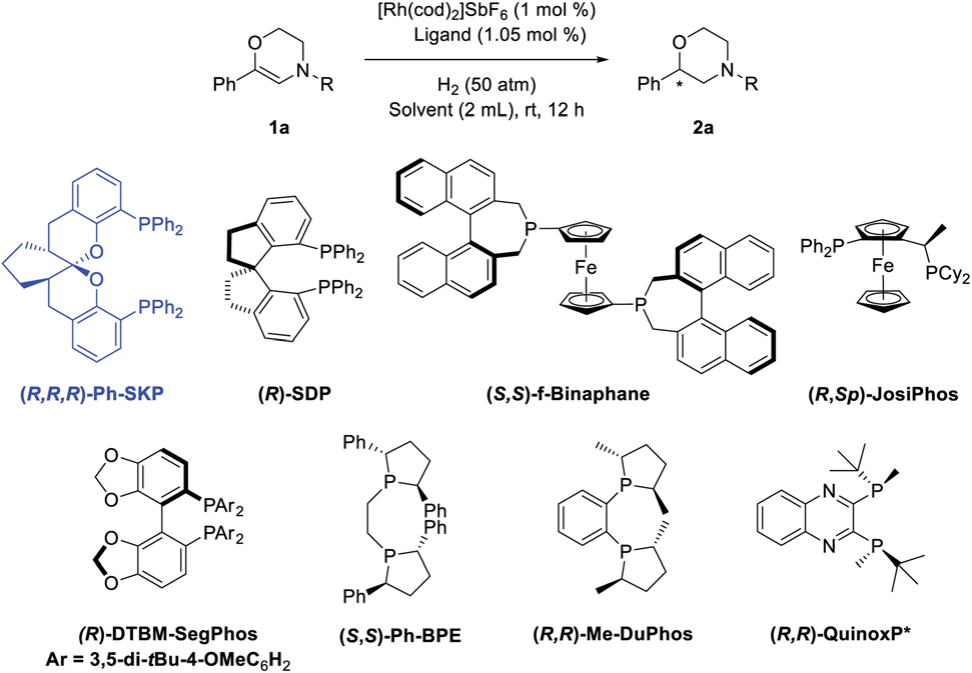					
Entry^a^	R	Ligand	Solvent	Conv.^b^ (%)	ee^c^ (%)
1	Cbz	**SKP**	DCM	>99	92
2	4-NO_2_-Cbz	**SKP**	DCM	>99	26
3	COOiBu	**SKP**	DCM	>99	89
4	Boc	**SKP**	DCM	>99	75
5	Ts	**SKP**	DCM	NR	—
6	Cbz	**SDP**	DCM	24	70
7	Cbz	**f-Binaphane**	DCM	>99	4
8	Cbz	**JosiPhos**	DCM	97	63
9	Cbz	**DTBM-SegPhos**	DCM	NR	—
10	Cbz	**Me-DuPhos**	DCM	NR	—
11	Cbz	**Ph-BPE**	DCM	NR	—
12	Cbz	QuinoxP*	DCM	NR	—
13	Cbz	**SKP**	AcOEt	32	79
14	Cbz	**SKP**	Toluene	42	91
15	Cbz	**SKP**	DCE	<10	—
16	Cbz	**SKP**	MeOH	<10	—
17	Cbz	**SKP**	THF	NR	—
18	Cbz	**SKP**	Dioxane	NR	—
19^d^	Cbz	**SKP**	DCM	98	92
20^e^	Cbz	**SKP**	DCM	>99	92
21^f^	Cbz	**SKP**	DCM	56	92

a Conditions: **1a** (0.2 mmol), [Rh(cod)_2_]SbF_6_ (1 mol%), ligand (1.05 mol%), H_2_ (50 atm), solvent (2 mL), rt, 12 h, unless otherwise noted.

b Conversions were calculated from ^1^H NMR spectra.

c The ee values of **2a** were determined by HPLC using chiral columns.

d 30 atm, 12 h.

e 30 atm, 24 h.

f 10 atm, 24 h.

Using the optimized reaction conditions of entry 20 in Table 1, a variety of substrates bearing different substituents were examined (Table 2). All the unsaturated morpholines were converted into their corresponding products in quantitative yields and with satisfactory enantioselectivities. Among the 4-substituted substrates **1b–h**, the electron-withdrawing trifluoromethyl substituted substrate **1f** gave the corresponding product **2f** with the highest 94% ee. Among the 3-substituted substrates **1i–l**, the electron-donating methoxy substituted substrate **1l** gave the corresponding product **2l** with the highest 94% ee. It is worth noting that substrates **1m–p** bearing a substituent at the 2-position all gave excellent enantioselectivities (99% ee) for both electron-withdrawing and electron-donating species, which can be attributed to the steric effects of the aromatic ring substituents. A similar *ortho*-effect occurs in the **SKP**-catalyzed asymmetric hydrogenation of *E*-allylamides, which points to the same stereocontrol pattern.^10*s*^ 3,4-Disubstituted unsaturated morpholines **1q** and **1r** were also subjected to the reaction conditions, affording the corresponding products in high yields with 94% and 88% ees, respectively. When the phenyl ring was changed to either a naphthalene (**1s**) or thiophene ring (**1t**), satisfactory yields were also observed, albeit with a comparatively low ee value for the thiophene species. The method also applies to alkyl-substituted substrates (**1u–w**) which gave the corresponding products with 81%, 58%, and 91% ees, respectively. The absolute configurations of products were assigned to be the same as **2b** which was confirmed by single-crystal analysis (see the ESI† for details).

**Table 2:** Substrate scope^a^

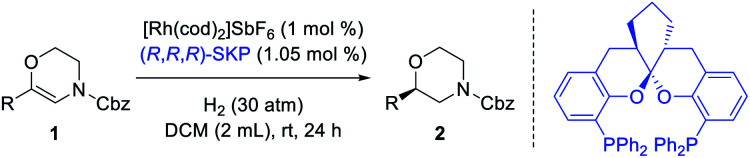
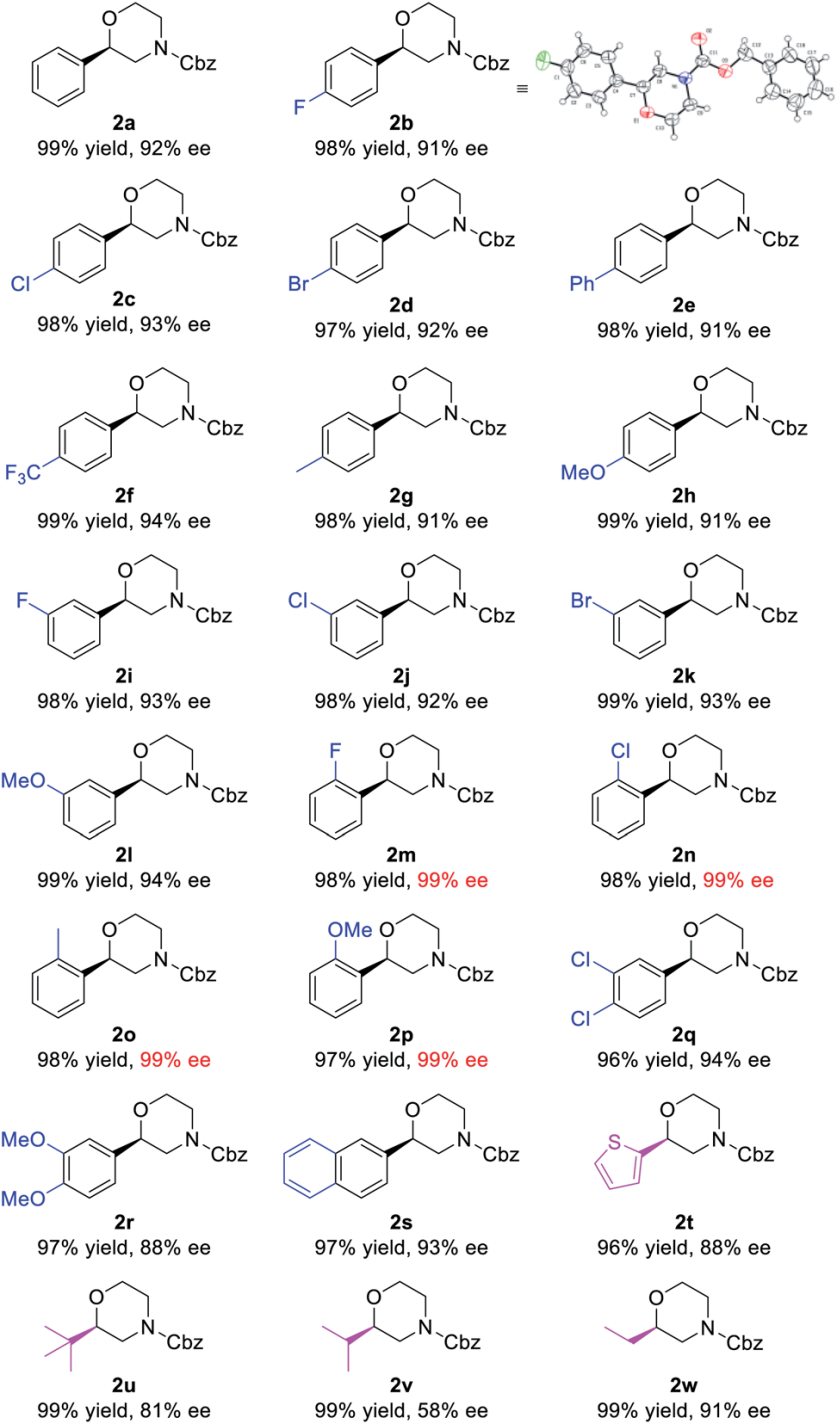

a Conditions: **1** (0.2 mmol), (*R*,*R*,*R*)-**SKP** (1.05 mol%), [Rh(cod)_2_]SbF_6_ (1 mol%), H_2_ (30 atm), DCM (2 mL), rt, 24 h. Yields of isolated products are given. The ee values were determined by HPLC using chiral stationary phases.

**Fig. 3:**
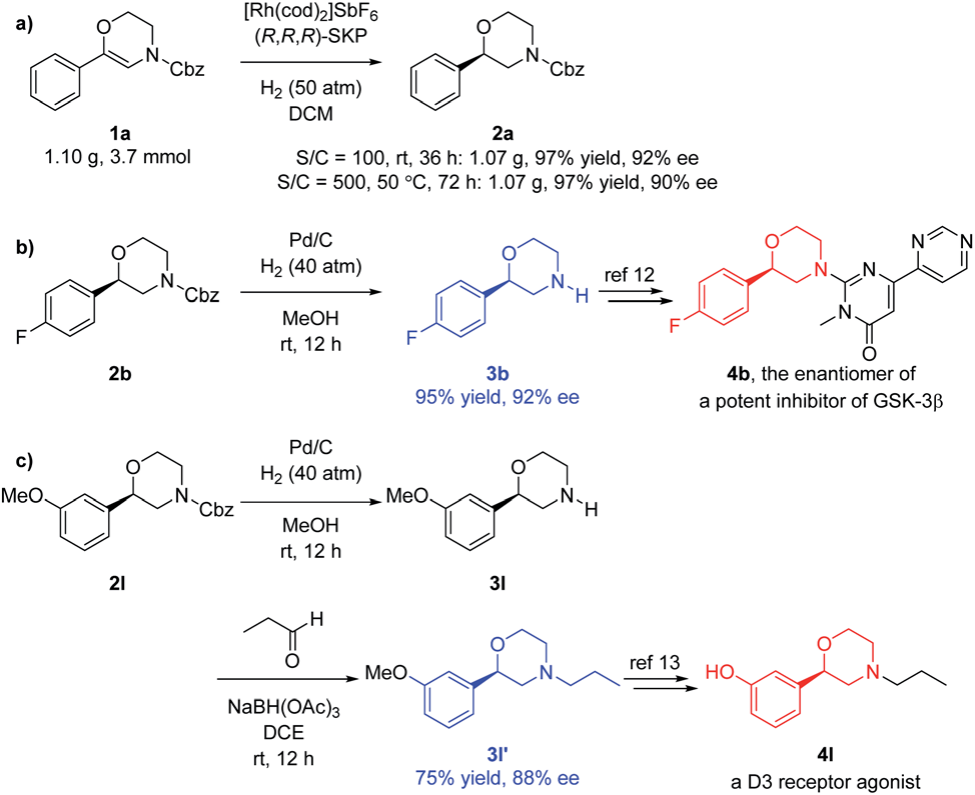
Scale-up and applications. (a) Gram scale hydrogenation; (b) for the synthesis of a potent GSK-3β inhibitor; (c) for the synthesis of a D3 receptor agonist.

To further evaluate the potential application of this methodology, the gram scale hydrogenation of **1a** was performed, delivering the desired product **2a** in 97% yield and 92% ee. Decreasing the catalyst amount from 1 to 0.2 mol% did not obviously affect the result if the reaction time and temperature were increased (Fig. 3a). Using Pd/C–H_2_, the Cbz group of the hydrogenated products **2** can be easily removed to obtain the free NH morpholines **3**, which can be further applied to the synthesis of bioactive compounds. For instance, deprotection of **2b** bearing the electron-withdrawing 4-fluoro substituent afforded the free NH morpholine **3b** in 95% yield and 92% ee. Compound **3b** can be transformed into **4b***via* aromatic nucleophilic substitution, which is the enantiomer of a potent GSK-3β inhibitor (Fig. 3b).^12^ Another deprotected product **3l** bearing an electron-donating 3-methoxy substituent can be further converted to a dopamine 3 receptor agonist **4l***via* reductive amination to **3l′** in 75% yield and 88% ee and further demethylation (Fig. 3c).^13^

**Fig. 4:**
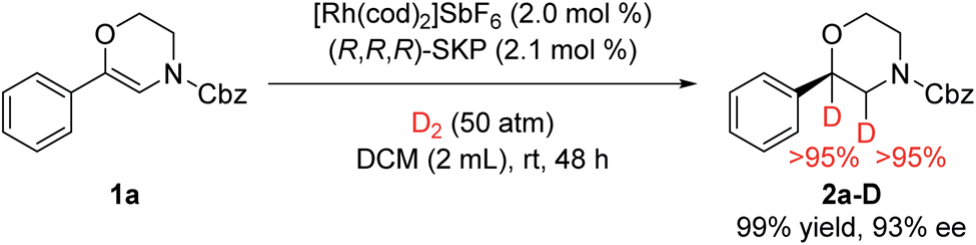
Deuterium labelling experiment.

A deuterium-labelling experiment was also conducted to reveal the mechanism of this reaction (Fig. 4). When H_2_ was replaced by D_2_, two deuterium atoms were substituted for both of the adjacent carbons, which indicates that the hydrogenation only occurs at the C=C bond of the enamide stage.

## Conclusions

In summary, we have developed the first asymmetric hydrogenation of 2-substituted dehydromorpholines catalyzed by the **SKP**–Rh complex bearing a large bite angle. With this method, a variety of 2-substituted chiral morpholines were obtained in quantitative yields and excellent enantioselectivities (up to 99% ee). The reaction can be carried out on a gram scale and the corresponding chiral products could subsequently be transformed into important intermediates required for the preparation of useful drug candidates.

## Data availability

All experimental data associated with this work is available in the ESI.†

## Author contributions

M. L. conducted most of the experiments and wrote the initial manuscript draft. J. Z. screened the initial reaction conditions. Y. Z. and F. Z. performed part of the experiments. Z. Z. conceived the project and finalized the manuscript. Z. Z. and W. Z. directed the project. All the authors co-wrote the paper. All authors discussed the results and commented on the manuscript.

## Conflicts of interest

There are no conflicts to declare.

## Supplementary Material

SC-012-D1SC04288B-s001

SC-012-D1SC04288B-s002
