# Supplementary material for: Asymmetric hydrogenation for the synthesis of 2-substituted chiral morpholines
Source: Chem Sci. 2021 Oct 28;12(45):15061–6. doi: 10.1039/d1sc04288b (PMC8612400; doi:10.1039/d1sc04288b)
Supplement: SC-012-D1SC04288B-s001 [file SC-012-D1SC04288B-s001.pdf]

Electronic Supplementary Material (ESI) for Chemical Science.
This journal is © The Royal Society of Chemistry 2021

Supporting Information

Table of Contents

1. General Details S2
2. Preparation of Substrates S3
3. Asymmetric Hydrogenation S13
4. Deuteration Experiment S23
5. Applicatons S24
6. X-Ray Analysis Data S27
7. NMR Spectra S31
8. HPLC Charts... S100
9. Reference S132

1. General Details

All hydrogenation reactions were performed in an autoclave under an atmosphere of hydrogen. All air- and moisture-sensitive reactions were performed in dried glassware under an atmosphere of nitrogen. The workup was carried out in air, unless otherwise noted. Column chromatography was performed using silica gel. Solvents were dried and distilled before use by standard procedures. Commercially available reagents were used without further purification.

Melting points were measured with SGW X-4 micro melting point apparatus. ^{1}H NMR (400 and 500 MHz), ^{13}C NMR (100 and 125 MHz), and ^{19}F NMR (376 and 470 MHz) were recorded on a Bruker Avance III HD 400 MHz NMR spectrometer and a Bruker Avance III HD 500 MHz NMR Spectrometer. HRMS data were measured with a Waters Micromass Q-TOF Premier Mass Spectrometer at the Analysis Center of Shanghai Jiao Tong University. Optical rotations were measured on a Rudolph Research Analytical Autopol VI automatic polarimeter using a 50 mm path-length cell at 589 nm. Enantiomeric excess (ee) values were measured with a Shimadzu LC-2010A HPLC system using Daicel Chiralcel columns.

2. Preparation of Substrates

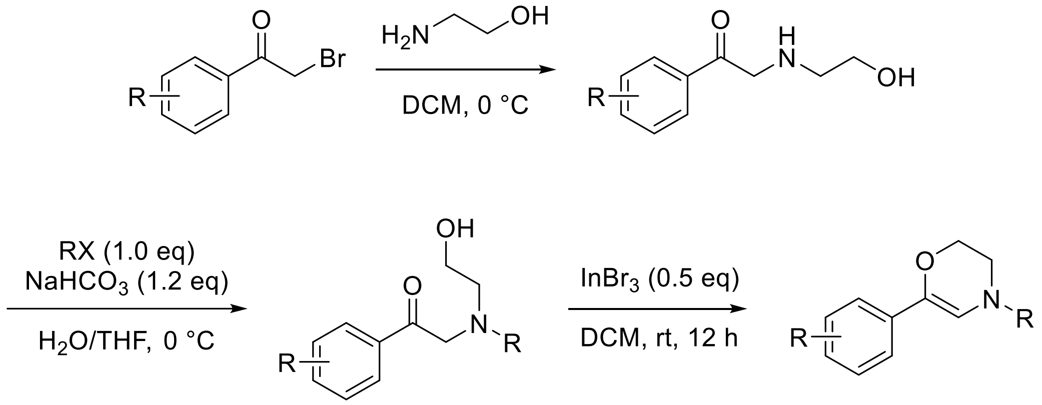

General procedure for 1a-w: According to the procedure reported in the literature.[1]

To a stirred solution of ethanolamine (10.0 equiv) in DCM (50 mL), *α*-bromoacetophenone (5.0 g, 25.1 mmol) dissolved in DCM (50 mL) was added dropwise. The reaction system was stirred at 0 °C. After complete consumption of the ketone substrate (monitored by TLC), the resultant mixture was washed twice with aqueous NaOH solution (5 M, 100 mL × 2). The organic layer was concentrated at reduced pressure and the crude product was used in the next step without further purification.

The crude product obtained above was dissolved in THF/H_2O (80/50 mL), $NaHCO_3$ (1.2 equiv) was added and the mixture was cooled in iced water to 0 °C. Benzyl chloroformate (Cbz-Cl) (1.0 equiv) dissolved in THF (25 mL) was added dropwise at 0 °C and stirred until complete conversion. Then the reaction mixture was diluted with ethyl acetate (EA) and washed with brine. The organic layer was separated, dried over anhydrous $MgSO_4$, filtered and then concentrated under reduced pressure. The residue was purified on a silica gel column with petroleum ether / ethyl acetate (PE/EA = 2/1) as eluent to afford the hydroxyketone intermediate.

An oven-dried 25 mL Schlenk flask was charged with $InBr_3$ (0.5 equiv) and evacuated and backfilled with nitrogen (this sequence was carried out twice). The hydroxyketone intermediate in anhydrous DCM (20 mL) was added into the reaction flask via syringe under N_2 atmosphere and the reaction mixture stirred at room temperature for 12 h. The solvent was removed under reduced pressure and the residue was purified by column chromatography (PE/EA = 10/1).

The 2,3-disubstituted substrate **1y** was synthesized according to the same procedure.

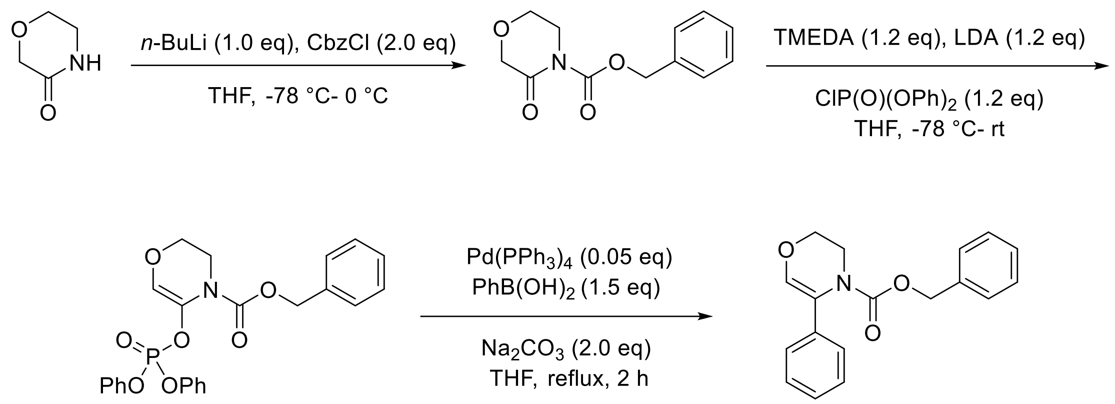

Procedure for 1x: According to the procedure for the synthesis of similar compounds reported in the literatures.[2,3]

To a cold solution (-78 °C) of morpholin-3-one (2.5 g, 25 mmol) in dry THF (200 mL) was added a solution of *n*-BuLi (2.5 M in hexane, 10 mL, 25 mmol) under nitrogen atmosphere. The reaction system was stirred for 30 min at -78 °C and benzyl chloroformate (CbzCl) (50 mmol) was added dropwise. The mixture was stirred overnight at room temperature and treated with saturated ammonium chloride aqueous solution (300 mL) and extracted with DCM for three times (100 mL × 3). The organic layer was separated, dried over anhydrous $MgSO_4$, filtered and then concentrated under reduced pressure. The residue was purified on a silica gel column with PE/EA (3/1) as eluent to afford benzyl 3-oxomorpholine-4-carboxylate.

To a cold solution (-78 °C) of benzyl 3-oxomorpholine-4-carboxylate (2.0 g, 8.5 mmol) in dry THF (80 mL) was added TMEDA (1.9 mL, 12.8 mmol) and LDA (1.7 mL, 12.8 mmol) under nitrogen atmosphere. The reaction system was stirred for 90 min at -78 °C and freshly distilled $ClP(O)(OPh)_2$ (2.7 mL, 12.8 mmol) was added dropwise. The mixture was stirred for 3 h and allowed to warm to room temperature, then treated with water (150 mL) and extracted with DCM for three times (100 mL × 3). The organic layer was separated, dried over anhydrous $MgSO_4$, filtered and then concentrated under reduced pressure. The residue was purified on a silica gel column with PE/EA (1/1) as eluent to afford benzyl 5-((diphenoxyphosphoryl)oxy)-2,3-dihydro-4*H*-1,4-oxazine-4-carboxylate.

To a solution of benzyl 5-((diphenoxyphosphoryl)oxy)-2,3-dihydro-4*H*-1,4-oxazine-4-carboxylate (1.0 g) in THF (50 mL) at room temperature, 2.0 M Na_2CO_3 aqueous solution (10 mL), $Pd(PPh_3)_4$ (124 mg, 2.1 mmol) and benzeneboronic acid (391 mg, 3.2 mmol) were added sequentially. The mixture was refluxed for 2 h and allowed to cool to room temperature, then treated with water (100 mL) and extracted with DCM for three times (50 mL × 3). The organic layer was separated, dried over anhydrous $MgSO_4$, filtered and then concentrated under reduced pressure. The residue was purified on a silica gel column with PE/EA (5/1) as eluent to afford the product **1x**. The total yield of the above three steps was 39% (according to morpholin-3-one).

Benzyl 6-phenyl-2,3-dihydro-4*H*-1,4-oxazine-4-carboxylate (1a)

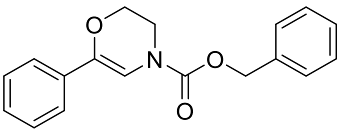

Colorless oil (2.37 g, 32% yield). ^{1}H NMR (400 MHz, $CDCl_3$): (two rotamers: 55/45) δ 7.52-7.46 (m, 2H), 7.42-7.28 (m, 7H), 7.23-7.21 (m, 1H), 7.02 (s, 0.45H), 6.85 (s, 0.55H), 5.25 (s, 1H), 5.22 (s, 1H), 4.24 (t, J = 4.4 Hz, 1H), 4.20 (t, J = 4.4 Hz, 1H), 3.82-3.78 (m, 2H). ^{13}C NMR (100 MHz, $CDCl_3$): δ 152.41, 152.19, 138.99, 137.90, 136.22, 136.14, 134.24, 128.72, 128.46, 128.42, 128.30, 128.29, 127.64, 127.54, 123.66, 123.45, 102.93, 102.31, 67.97, 67.93, 64.86, 64.41, 42.16, 41.55. ESI-MS calcd for $C_{18}H_{18}NO_3$ $[M+H]^+$ 296.1281, found 296.1280.

Benzyl 6-(4-fluorophenyl)-2,3-dihydro-4*H*-1,4-oxazine-4-carboxylate (1b)

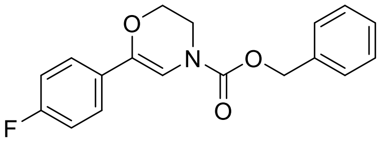

White solid (1.08 g, 15% yield). Mp: 65-66 °C. ^{1}H NMR (400 MHz, $CDCl_3$): (two rotamers: 55/45) δ 7.49-7.33 (m, 7H), 7.02-6.98 (m, 2H), 6.94 (s, 0.45H), 6.77 (s, 0.55H), 5.25 (s, 1H), 5.23 (s, 1H), 4.25 (t, J = 4.4 Hz, 1H), 4.21 (t, J = 4.4 Hz, 1H), 3.83-3.79 (m, 2H). ^{13}C NMR (125 MHz, $CDCl_3$): δ 163.37, 163.33, 161.40, 161.37, 152.34, 152.15, 138.31, 137.20, 136.17, 136.09, 130.41, 130.39, 128.71, 128.45, 128.31, 128.29, 125.46, 125.40, 125.24, 125.18, 115.41, 115.24, 102.63, 102.01, 67.99, 67.94, 64.92, 64.47, 42.06, 41.46. ^{19}F NMR (376 MHz, $CDCl_3$): δ -114.40, -114.61. ESI-MS calcd for $C_{18}H_{17}FNO_3$ $[M+H]^+$ 314.1187, found 314.1183.

1-(6-(4-chlorophenyl)-2,3-dihydro-4*H*-1,4-oxazin-4-yl)-2-phenylethan-1-one (1c)

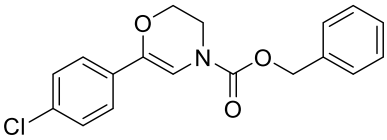

White solid (1.84 g, 26% yield). Mp: 76-77 °C. ^{1}H NMR (400 MHz, $CDCl_3$): (two rotamers: 54/46) δ 7.43-7.34 (m, 7H), 7.27-7.24 (m, 2H), 7.01 (s, 0.46H), 6.83 (s, 0.54H), 5.25 (s, 1H), 5.22 (s, 1H), 4.23 (t, J = 4.4 Hz, 1H), 4.18 (t, J = 4.4 Hz, 1H), 3.82-3.77 (m, 2H). ^{13}C NMR (100 MHz, $CDCl_3$): δ 152.29, 152.13, 137.99, 136.88, 136.11, 136.02, 133.14, 133.04, 132.73, 128.72, 128.54, 128.48, 128.32, 124.83, 124.63, 103.29, 102.65, 68.04, 67.99, 64.83, 64.39, 42.07, 41.47. ESI-MS calcd for $C_{18}H_{17}ClNO_3$ $[M+H]^+$ 330.0891, found 330.0895.

1-(6-(4-bromophenyl)-2,3-dihydro-4*H*-1,4-oxazin-4-yl)-2-phenylethan-1-one (1d)

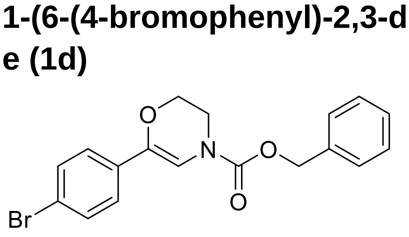

White solid (1.95 g, 29% yield). Mp: 117-118 °C. ^{1}H NMR (500 MHz, $CDCl_3$): (two rotamers: 53/47) δ 7.42-7.31 (m, 9H), 7.01 (s, 0.47H), 6.84 (s, 0.53H), 5.24 (s, 1H), 5.21 (s, 1H), 4.22 (t, J = 4.5 Hz, 1H), 4.18 (t, J = 4.5 Hz, 1H), 3.81-3.76 (m, 2H). ^{13}C NMR (125 MHz, $CDCl_3$): δ 152.29, 152.13, 138.00, 136.89, 136.08, 136.00, 133.18, 131.47,

128.72, 128.48, 128.32, 128.31, 125.11, 124.91, 121.25, 121.16, 103.35, 102.70, 68.06, 68.01, 64.81, 64.37, 42.07, 41.47. ESI-MS calcd for $C_{18}H_{17}BrNO_3$ $[M+H]^+$ 374.0386, found 374.0382.

Benzyl 6-([1,1'-biphenyl]-4-yl)-2,3-dihydro-4*H*-1,4-oxazine-4-carboxylate (1e)

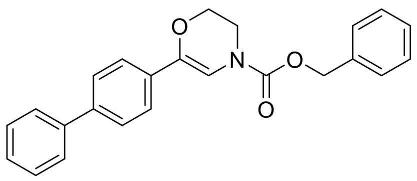

White solid (2.56 g, 38% yield). Mp: 97-98 °C. ^{1}H NMR (400 MHz, $CDCl_3$): (two rotamers: 55/45) δ 7.61-7.55 (m, 6H), 7.45-7.31 (m, 8H), 7.08 (s, 0.45H), 6.91 (s, 0.55H), 5.27 (s, 1H), 5.24 (s, 1H), 4.28 (t, J = 4.4 Hz, 1H), 4.24 (t, J = 4.4 Hz, 1H), 3.85-3.82 (m, 2H). ^{13}C NMR (125 MHz, $CDCl_3$): δ 152.41, 152.20, 140.76, 140.73, 140.32, 140.19, 138.76, 137.66, 136.24, 136.14, 133.24, 128.90, 128.75, 128.50, 128.47, 128.33, 127.42, 127.38, 127.10, 127.01, 124.02, 123.81, 103.08, 102.45, 68.03, 67.99, 64.89, 64.44, 42.22, 41.62. ESI-MS calcd for $C_{24}H_{22}NO_3$ $[M+H]^+$ 372.1594, found 372.1597.

Benzyl 6-(4-(trifluoromethyl)phenyl)-2,3-dihydro-4*H*-1,4-oxazine-4-carboxylate (1f)

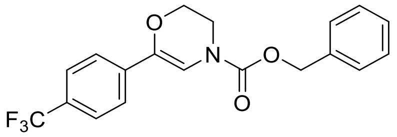

Colorless oil (1.56 g, 23% yield). ^{1}H NMR (400 MHz, $CDCl_3$): (two rotamers: 52/48) δ 7.61-7.53 (m, 4H), 7.43-7.34 (m, 5H), 7.14 (s, 0.48H), 6.95 (s, 0.52H), 5.26 (s, 1H), 5.24 (s, 1H), 4.26 (t, J = 4.4 Hz, 1H), 4.22 (t, J = 4.4 Hz, 1H), 3.85-3.81 (m, 2H). ^{13}C NMR (125 MHz, $CDCl_3$): δ 152.33, 152.24, 137.70, 137.62, 136.52, 136.03, 135.94, 129.61, 129.51, 129.35, 129.25, 129.09, 128.99, 128.79, 128.59, 128.41, 127.58, 127.55, 125.44, 125.41, 125.38, 125.35, 123.56, 123.34, 123.26, 123.23, 121.10, 121.07, 104.81, 104.15, 68.23, 68.19, 64.84, 64.40, 42.18, 41.56. ^{19}F NMR (376 MHz, $CDCl_3$): δ -62.44, -62.47. ESI-MS calcd for $C_{19}H_{17}F_3NO_3$ $[M+H]^+$ 364.1155, found 364.1157.

Benzyl 6-(*p*-tolyl)-2,3-dihydro-4*H*-1,4-oxazine-4-carboxylate (1g)

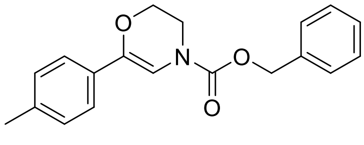

Colorless oil (1.45 g, 20% yield). ^{1}H NMR (400 MHz, $CDCl_3$): (two rotamers: 56/44) δ 7.41-7.32 (m, 7H), 7.13-7.10 (m, 2H), 6.97 (s, 0.44H), 6.79 (s, 0.56H), 5.24 (s, 1H), 5.22 (s, 1H), 4.24 (t, J = 4.4 Hz, 1H), 4.20 (t, J = 4.4 Hz, 1H), 3.82-3.78 (m, 2H), 2.33 (s, 3H). ^{13}C NMR (125 MHz, $CDCl_3$): δ 152.40, 152.15, 139.18, 138.08, 137.48, 137.35, 136.28, 136.18, 131.43, 129.11, 128.70, 128.43, 128.39, 128.29, 128.25, 123.64, 123.42, 102.20, 101.60, 67.89, 67.86, 64.86, 64.41, 42.14, 41.54, 21.29. ESI-MS calcd for $C_{19}H_{20}NO_3$ $[M+H]^+$ 310.1438, found 310.1440.

Benzyl 6-(4-methoxyphenyl)-2,3-dihydro-4*H*-1,4-oxazine-4-carboxylate

(1h)

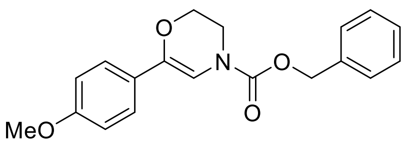

Colorless oil (2.13 g, 30% yield). ^{1}H NMR (400 MHz, $CDCl_3$): (two rotamers: 54/46) δ 7.44-7.30 (m, 7H), 6.89 (s, 0.46H), 6.84-6.81 (m, 2H), 6.71 (s, 0.54H), 5.23 (s, 1H), 5.20 (s, 1H), 4.20 (t, J = 4.4 Hz, 1H), 4.16 (t, J = 4.4 Hz, 1H), 3.79-3.74 (m, 5H). ^{13}C NMR (125 MHz, $CDCl_3$): δ 152.41, 152.18, 148.93, 148.86, 148.77, 139.05, 137.97, 136.31, 136.18, 128.72, 128.66, 128.47, 128.41, 128.31, 128.22, 128.01, 127.85, 127.35, 127.26, 116.44, 116.22, 111.13, 111.11, 107.49, 106,91, 101.76, 101.22, 67.90, 65.01, 64.54, 56.05, 56.04, 56.03, 56.00, 42.15, 41.53. ESI-MS calcd for $C_{19}H_{20}NO_4$ [M+H]$^+$ 326.1387, found 326.1384.

Benzyl 6-(3-fluorophenyl)-2,3-dihydro-4*H*-1,4-oxazine-4-carboxylate (1i)

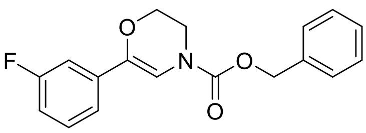

White solid (1.95 g, 27% yield). Mp: 112-113 °C. ^{1}H NMR (400 MHz, $CDCl_3$): (two rotamers: 56/44) δ 7.49-7.33 (m, 7H), 7.03-6.97 (m, 2H), 6.94 (s, 0.44H), 6.77 (s, 0.56H), 5.25 (s, 1H), 5.22 (s, 1H), 4.25 (t, J = 4.4 Hz, 1H), 4.21 (t, J = 4.4 Hz, 1H), 3.82-3.79 (m, 2H). ^{13}C NMR (125 MHz, $CDCl_3$): δ 164.07, 164.03, 162.13, 162.09, 152.31, 152.16, 137.81, 137.79, 136.70, 136.68, 136.60, 136.54, 136.07, 135.99, 129.88, 129.81, 128.74, 128.50, 128.35, 128.33, 119.00, 118.98, 118.80, 118.78, 114.33, 114.21, 114.16, 114.04, 110.70, 110.51, 110.34, 103.88, 103.23, 68.09, 68.04, 64.80, 64.36, 42.12, 41.51. ^{19}F NMR (376 MHz, $CDCl_3$): δ -114.39, -114.59. ESI-MS calcd for $C_{18}H_{17}FNO_3$ [M+H]$^+$ 314.1187, found 314.1186.

Benzyl 6-(3-chlorophenyl)-2,3-dihydro-4*H*-1,4-oxazine-4-carboxylate (1j)

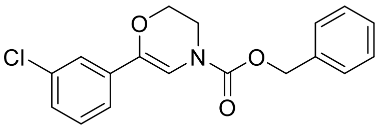

Colorless oil (1.69 g, 24% yield). ^{1}H NMR (500 MHz, $CDCl_3$): (two rotamers: 53/47) δ 7.50-7.33 (m, 7H), 7.24-7.18 (m, 2H), 7.04 (s, 0.47H), 6.86 (s, 0.53H), 5.26 (s, 1H), 5.23 (s, 1H), 4.24 (t, J = 4.0 Hz, 1H), 4.20 (t, J = 4.0 Hz, 1H), 3.83-3.79 (m, 2H). ^{13}C NMR (125 MHz, $CDCl_3$): δ 152.35, 152.19, 137.69, 136.59, 136.12, 136.10, 136.00, 134.55, 134.53, 129.63, 128.77, 128.54, 128.40, 128.37, 127.49, 127.38, 123.76, 123.60, 121.58, 121.33, 103.95, 103.30, 68.13, 68.09, 64.86, 64.42, 42.16, 41.55. ESI-MS calcd for $C_{18}H_{17}ClNO_3$ [M+H]$^+$ 330.0891, found 330.0893.

Benzyl 6-(3-bromophenyl)-2,3-dihydro-4*H*-1,4-oxazine-4-carboxylate (1k)

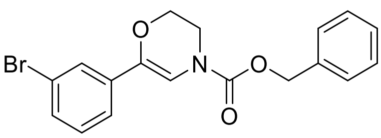

White solid (1.28 g, 19% yield). Mp: 88-89 °C. ^{1}H NMR (400 MHz, $CDCl_3$): (two rotamers: 54/46) δ 7.64 (d, J = 16.0 Hz, 1H), 7.42-7.33 (m, 7H), 7.15 (t, J = 8.0 Hz, 1H), 7.03 (s, 0.46H), 6.85 (s, 0.54H), 5.25 (s, 1H), 5.23 (s, 1H), 4.23 (t, J = 4.4 Hz, 1H), 4.19 (t, J = 4.4 Hz, 1H), 3.82-3.78 (m, 2H). ^{13}C NMR (100 MHz, $CDCl_3$): δ 152.32, 152.16, 137.50, 136.44, 136.36, 136.34, 136.10, 136.00, 130.38, 130.27, 129.88, 128.75,

128.52, 128.39, 126.65, 126.47,122.73, 122.01, 121.77, 103.97, 103.32, 68.11, 68.08, 64.84, 64.40, 42.14, 41.53. ESI-MS calcd for $C_{18}H_{17}BrNO_3$ $[M+H]^+$ 374.0386, found 374.0389.

Benzyl 6-(3-methoxyphenyl)-2,3-dihydro-4*H*-1,4-oxazine-4-carboxylate (1l)

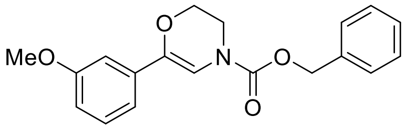

Colorless oil (2.27 g, 32% yield). ^{1}H NMR (400 MHz, $CDCl_3$): (two rotamers: 53/47) δ 7.42-7.33 (m, 6H), 7.24-7.20 (m, 1H), 7.12-7.03 (m, 2.47H), 6.85 (s, 0.53H), 5.25 (s, 1H), 5.23 (s, 1H), 4.25 (t, J = 4.4 Hz, 1H), 4.21 (t, J = 4.4 Hz, 1H), 3.83-3.80 (m, 5H). ^{13}C NMR (100 MHz, $CDCl_3$): δ 159.78, 152.37, 152.16, 138.80, 137.66, 136.20, 136.11, 135.73, 135.69, 129.40, 128.71, 128.46, 128.42, 128.29, 128.27, 116.08, 115.94, 113.52, 113.18, 109.42, 108.73, 103.21, 102.62, 67.96, 64.83, 64.38, 55.35, 42.16, 41.55. ESI-MS calcd for $C_{19}H_{20}NO_4$ $[M+H]^+$ 326.1387, found 326.1387.

Benzyl 6-(2-fluorophenyl)-2,3-dihydro-4*H*-1,4-oxazine-4-carboxylate (1m)

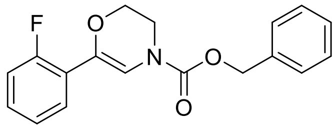

Colorless oil (1.66 g, 23% yield). ^{1}H NMR (400 MHz, $CDCl_3$): (two rotamers: 60/40) δ 7.57 (t, J = 8.0 Hz, 1H), 7.42-7.33 (m, 5H), 7.21-7.10 (m, 3H), 7.07-7.01 (m, 1H), 5.25 (s, 1.2H), 5.23 (s, 0.8H), 4.25 (t, J = 4.4 Hz, 1H), 4.21 (t, J = 4.4 Hz, 1H), 3.85-3.82 (m, 2H). ^{13}C NMR (100 MHz, $CDCl_3$): δ 160.32, 160.26, 157.83, 157.78, 152.35, 152.10, 136.21, 136.13, 133.81, 133.76, 132.48, 132.44, 128.70, 128.47, 128.41, 128.33, 128.04, 127.18, 127.15, 126.96, 126.94, 124.15, 124.12, 124.04, 124.01, 122.25, 122.14, 116.11, 116.03, 115.88, 115.79, 108.16, 107.99, 107.62, 107.45, 67.97, 67.94, 64.71, 64.27, 42.24, 41.64. ^{19}F NMR (376 MHz, $CDCl_3$): δ -112.30, -112.88. ESI-MS calcd for $C_{18}H_{17}FNO_3$ $[M+H]^+$ 314.1187, found 314.1185.

Benzyl 6-(2-chlorophenyl)-2,3-dihydro-4*H*-1,4-oxazine-4-carboxylate (1n)

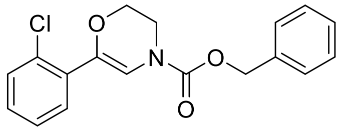

Colorless oil (2.19 g, 31% yield). ^{1}H NMR (400 MHz, $CDCl_3$): (two rotamers: 60/40) δ 7.43-7.30 (m, 7H), 7.23-7.21 (m, 2H), 6.85 (s, 0.40H), 6.72 (s, 0.60H), 5.21 (s, 2H), 4.25 (t, J = 4.4 Hz, 1H), 4.20 (t, J = 4.4 Hz, 1H), 3.85-3.81 (m, 2H). ^{13}C NMR (100 MHz, $CDCl_3$): δ 152.30, 152.15, 137.64, 136.31, 136.09, 133.49, 133.00, 130.44, 130.34, 130.27, 129.40, 129.36, 128.71, 128.68, 128.47, 128.39, 128.32, 128.21, 126.68, 107.23, 106.68, 67.94, 67.92, 64.69, 64.25, 42.14, 41.52. ESI-MS calcd for $C_{18}H_{17}ClNO_3$ $[M+H]^+$ 330.0891, found 330.0895.

Benzyl 6-(*o*-tolyl)-2,3-dihydro-4*H*-1,4-oxazine-4-carboxylate (1o)

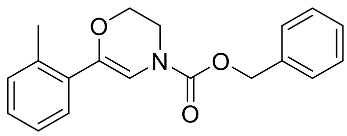

Colorless oil (1.60 g, 22% yield). ^{1}H NMR (400 MHz, $CDCl_3$): (two rotamers: 60/40) δ 7.36-7.26 (m, 6H),

7.20-7.10 (m, 3H), 6.57 (s, 0.40H), 6.41 (s, 0.60H), 5.19 (s, 2H), 4.17 (t, *J* = 4.4 Hz, 1H), 4.13 (t, *J* = 4.4 Hz, 1H), 3.80 (t, *J* = 4.4 Hz, 1H), 3.76 (t, *J* = 4.4 Hz, 1H), 2.35-2.34 (m, 3H). ^{13}C NMR (100 MHz, $CDCl_3$): δ 152.21, 151.99, 140.56, 139.41, 137.13, 136.87, 136.15, 136.11, 134.29, 134.23, 130.46, 128.97, 128.85, 128.59, 128.56, 128.46, 128.35, 128.31, 128.25, 128.18, 128.14, 125.58, 105.24, 104.60, 67.73, 67.66, 64.43, 64.01, 41.88, 41.26, 20.46, 20.34. ESI-MS calcd for $C_{19}H_{20}NO_3$ $[M+H]^+$ 310.1438, found 310.1439.

Benzyl 6-(2-methoxyphenyl)-2,3-dihydro-4*H*-1,4-oxazine-4-carboxylate (1p)

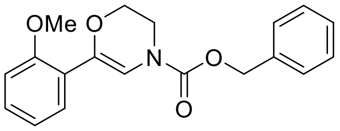

Colorless oil (2.77 g, 39% yield). ^{1}H NMR (400 MHz, $CDCl_3$): (two rotamers: 60/40) δ 7.59-7.56 (m, 1H), 7.45-7.31 (m, 6H), 7.23-7.18 (m, 1H), 6.95 (t, *J* = 7.6 Hz, 1H), 6.89 (t, *J* = 7.6 Hz, 1H), 5.24 (s, 1.2H), 5.22 (s, 0.8H), 4.25 (t, *J* = 4.4 Hz, 1H), 4.20 (t, *J* = 4.4 Hz, 1H), 3.89-3.81 (m, 5H). ^{13}C NMR (100 MHz, $CDCl_3$): δ 156.10, 155.99, 152.40, 152.16, 136.38, 136.29, 135.64, 134.41, 128.68, 128.60, 128.38, 128.23, 128.17, 128.13, 127.85, 127.12, 126.93, 122.85, 122.82, 120.65, 120.48, 110.96, 110.91, 107.90, 107.32, 67.75, 64.56, 64.14, 55.53, 55.42, 42.32, 41.72. ESI-MS calcd for $C_{19}H_{20}NO_4$ $[M+H]^+$ 326.1387, found 326.1386.

Benzyl 6-(3,4-dichlorophenyl)-2,3-dihydro-4*H*-1,4-oxazine-4-carboxylate (1q)

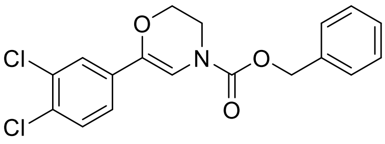

Colorless oil (0.81 g, 12% yield). ^{1}H NMR (400 MHz, $CDCl_3$): (two rotamers: 53/47) δ 7.58-7.55 (m, 1H), 7.41-7.25 (m, 7H), 7.03 (s, 0.47H), 6.85 (s, 0.53H), 5.25 (s, 1H), 5.23 (s, 1H), 4.23 (t, *J* = 4.4 Hz, 1H), 4.19 (t, *J* = 4.4 Hz, 1H), 3.82-3.69 (m, 2H). ^{13}C NMR (100 MHz, $CDCl_3$): δ 152.26, 152.15, 136.86, 136.02, 135.92, 135.74, 134.33, 132.67, 131.03, 130.91, 130.27, 128.76, 128.56, 128.42, 128.36, 125.40, 125.23, 122.60, 122.39, 104.23, 103.57, 68.19, 68.15, 64.84, 64.41, 42.09, 41.49. ESI-MS calcd for $C_{18}H_{16}Cl_2NO_3$ $[M+H]^+$ 364.0502, found 364.0506.

Benzyl
6-(3,4-dimethoxyphenyl)-2,3-dihydro-4*H*-1,4-oxazine-4-carboxylate (1r)[1]

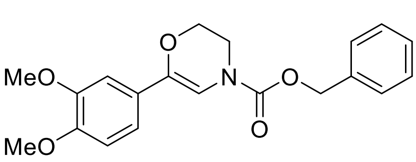

White solid (1.65 g, 24% yield). Mp: 72-73 ^{o}C. ^{1}H NMR (500 MHz, $CDCl_3$): (two rotamers: 50/50) δ 7.41-7.32 (m, 5H), 7.09-7.01 (m, 2H), 6.91 (s, 0.50H), 6.81 (t, *J* = 6.0 Hz, 1H), 6.73 (s, 0.50H), 5.25 (s, 1H), 5.22 (s, 1H), 4.25 (t, *J* = 4.0 Hz, 1H), 4.20 (t, *J* = 4.0 Hz, 1H), 3.89 (s, 3H), 3.87 (s, 1.5H), 3.86 (s, 1.5H), 3.82-3.78 (m, 2H). ^{13}C NMR (100 MHz, $CDCl_3$): δ 152.36, 152.13, 148.91, 148.84, 148.75, 139.02, 137.93, 136.29, 136.16, 128.68, 128.42, 128.37, 128.26, 128.17, 127.33, 127.24, 116.41, 116.21, 111.12, 111.10, 107.47, 106.90, 101.74, 101.19, 67.85, 64.98,

64.50, 56.01, 55.97, 42.12, 41.50. ESI-MS calcd for $C_{20}H_{22}NO_5$ $[M+H]^+$ 356.1492, found 356.1492.

Benzyl 6-(naphthalen-2-yl)-2,3-dihydro-4*H*-1,4-oxazine-4-carboxylate (1s)

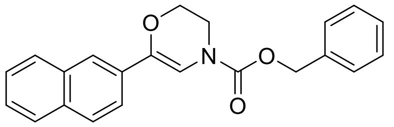

Colorless oil. (1.53 g, 22% yield). ^{1}H NMR (400 MHz, $CDCl_3$): (two rotamers: 52/48) *δ* 7.97 (d, *J* = 6.0 Hz, 1H), 7.82-7.74 (m, 3H), 7.62-7.53 (m, 1H), 7.47-7.34 (m, 7H), 7.17 (s, 0.48H), 7.00 (s, 0.52H), 5.28 (s, 1H), 5.25 (s, 1H), 4.32 (t, *J* = 4.4 Hz, 1H), 4.28 (t, *J* = 4.4 Hz, 1H), 3.88-3.85 (m, 2H). ^{13}C NMR (100 MHz, $CDCl_3$): *δ* 152.42, 152.23, 138.93, 137.81, 136.24, 136.13, 133.51, 132.90, 131.44, 128.75, 128.50, 128.48, 128.34, 128.26, 128.01, 127.69, 126.41, 126.36, 125.92, 125.86, 122.42, 122.11, 121.60, 121.55, 103.69, 103.03, 68.05, 68.01, 64.90, 64.46, 42.26, 41.67. ESI-MS calcd for $C_{22}H_{20}NO_3$ $[M+H]^+$ 346.1438, found 346.1434.

Benzyl 6-(thiophen-2-yl)-2,3-dihydro-4*H*-1,4-oxazine-4-carboxylate (1t)

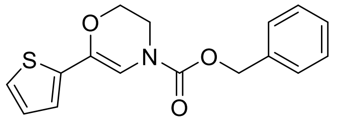

Yellow oil. (0.88 g, 12% yield). ^{1}H NMR (400 MHz, $CDCl_3$): (two rotamers: 52/48) *δ* 7.42-7.33 (m, 5H), 7.16 (d, *J* = 4.8 Hz, 1H), 7.11-7.08 (m, 1H), 6.97-6.94 (m, 1.48H), 6.78 (s, 0.52H), 5.24 (s, 1H), 5.22 (s, 1H), 4.25 (t, *J* = 4.0 Hz, 1H), 4.21 (t, *J* = 4.0 Hz, 1H), 3.82-3.79 (m, 2H). ^{13}C NMR (100 MHz, $CDCl_3$): *δ* 152.24, 151.98, 138.06, 138.00, 136.15, 136.06, 135.54, 134.31, 128.71, 128.48, 128.43, 128.32, 128.26, 127.35, 123.81, 123.73, 121.96, 121.73, 102.36, 101.80, 67.99, 65.15, 64.69, 42.12, 41.50. ESI-MS calcd for $C_{16}H_{16}NO_3S$. $[M+H]^+$ 302.0845, found 302.0849.

Benzyl 6-(*tert*-butyl)-2,3-dihydro-4*H*-1,4-oxazine-4-carboxylate (1u)

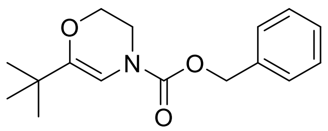

Colorless oil (2.08 g, 27% yield). ^{1}H NMR (400 MHz, $CDCl_3$): (two rotamers: 51/49) *δ* 7.39-7.28 (m, 5H), 6.23 (s, 0.49H), 6.09 (s, 0.51H), 5.20 (s, 1H), 5.17 (s, 1H), 4.03 (t, *J* = 4.0 Hz, 1H), 3.99 (t, *J* = 4.0 Hz, 1H), 3.66-3.63 (m, 2H), 1.08 (s, 4.4H), 1.07 (s, 4.6H). ^{13}C NMR (100 MHz, $CDCl_3$): *δ* 152.43, 152.17, 148.96, 147.71, 136.59, 136.45, 128.59, 128.25, 128.16, 127.99, 99.00, 98.33, 67.45, 67.42, 64.52, 64.12, 41.79, 41.26, 34.25, 34.16, 27.79, 27.76. ESI-MS calcd for $C_{16}H_{22}NO_3$ $[M+H]^+$ 276.1594, found 276.1597.

Benzyl 6-isopropyl-2,3-dihydro-4*H*-1,4-oxazine-4-carboxylate (1v)

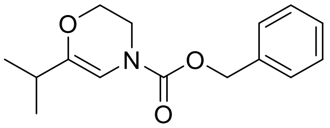

Colorless oil (2.61 g, 33% yield). ^{1}H NMR (400 MHz, $CDCl_3$): (two rotamers: 55/45) *δ* 7.40-7.32 (m, 5H), 6.17 (s, 0.45H), 6.03 (s, 0.55H), 5.19 (s, 1H), 5.17 (s, 1H), 4.05 (t, *J* = 4.0 Hz, 1H), 4.01 (t, *J* = 4.0 Hz, 1H), 3.68-3.66 (m, 2H), 2.31-2.24 (m, 1H), 1.07-1.04 (m, 6H). ^{13}C NMR (100 MHz, $CDCl_3$): *δ* 152.38, 152.09, 146.73, 145.45, 136.54, 136.45, 128.64, 128.30, 128.24, 128.20, 128.13, 99.53, 98.91, 67.52, 67.51, 64.71, 64.30, 41.84, 41.28,

31.10, 20.32. ESI-MS calcd for $C_{15}H_{20}NO_3$ $[M+H]^+$ 262.1438, found 262.1435.

Benzyl 6-ethyl-2,3-dihydro-4*H*-1,4-oxazine-4-carboxylate (1w)

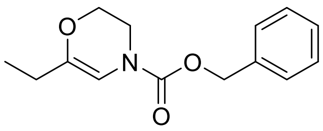

Colorless oil (2.87 g, 35% yield). ^{1}H NMR (400 MHz, $CDCl_3$): (two rotamers: 58/42) δ 7.39-7.29 (m, 5H), 6.15 (s, 0.42H), 6.02 (s, 0.58H), 5.18 (s, 1H), 5.16 (s, 1H), 4.05 (t, J = 4.0 Hz, 1H), 4.01 (t, J = 4.0 Hz, 1H), 3.67-3.63 (m, 2H), 2.10-2.01 (m, 2H), 1.06-1.01 (m, 3H). ^{13}C NMR (100 MHz, $CDCl_3$): δ 152.16, 151.85, 143.00, 141.77, 136.41, 136.35, 128.54, 128.20, 128.16, 128.09, 128.07, 100.22, 99.67, 67.45, 67.39, 64.66, 64.25, 41.58, 41.00, 25.24, 25.22, 11.65, 11.45. ESI-MS calcd for $C_{14}H_{17}NO_3$ $[M+H]^+$ 248.1281, found 248.1282.

Benzyl 5-phenyl-2,3-dihydro-4*H*-1,4-oxazine-4-carboxylate (1x)

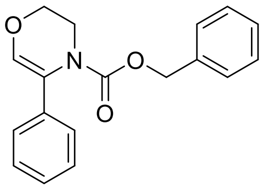

White solid (2.86 g, 39% yield). Mp: 140-141 ^{o}C. ^{1}H NMR (400 MHz, $CDCl_3$): δ 7.28-7.13 (m, 8H), 6.76 (s, 2H), 6.28 (s, 1H), 4.96 (s, 2H), 4.17-4.15 (t, J = 4.0 Hz, 2H), 3.85-3.83 (t, J = 4.0 Hz, 2H). ^{13}C NMR (100 MHz, $CDCl_3$): δ 154.60, 136.65, 135.57, 133.31, 128.35, 128.23, 127.80, 127.48, 126.65, 124.88, 119.32, 67.81, 66.75, 42.43. ESI-MS calcd for $C_{18}H_{18}NO_3$ $[M+H]^+$ 296.1281, found 296.1284.

Benzyl 6-(4-bromophenyl)-5-methyl-2,3-dihydro-4*H*-1,4-oxazine-4-carboxylate (1y)

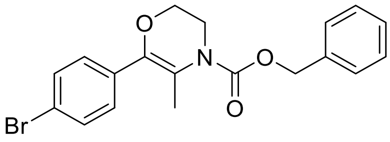

Colorless oil (2.33 g, 35% yield). ^{1}H NMR (400 MHz, $CDCl_3$): δ 7.38-7.16 (m, 9H), 5.12 (s, 2H), 4.11 (t, J = 4.0 Hz, 2H), 3.72 (t, J = 4.0 Hz, 2H), 1.95 (s, 3H). ^{13}C NMR (100 MHz, $CDCl_3$): δ 154.13, 139.38, 136.21, 134.60, 130.81, 128.68, 128.35, 128.25, 122.06, 111.51, 67.85, 66.38, 42.85, 18.58. ESI-MS calcd for $C_{19}H_{19}BrNO_3$ $[M+H]^+$ 388.0543, found 388.0547.

4-Nitrobenzyl 6-phenyl-2,3-dihydro-4*H*-1,4-oxazine-4-carboxylate(1a-NO_2)

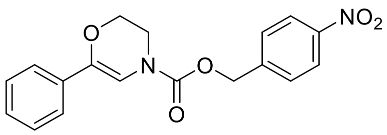

Yellow solid (2.65 g, 31% yield). Mp: 137-138 ^{o}C. ^{1}H NMR (400 MHz, $CDCl_3$): (two rotamers: 58/42) δ 8.24-8.22 (m, 2H), 7.56-7.48 (m, 4H), 7.34-7.24 (m, 3H), 6.98 (s, 0.42H), 6.84 (s, 0.58H), 5.33 (s, 1H), 5.31 (s, 1H), 4.28-4.24 (m, 2H), 3.85-3.81 (m, 2H). ^{13}C NMR (100 MHz, $CDCl_3$): δ 151.89, 151.67, 147.83, 143.51, 143.40, 139.49, 138.46, 134.01, 133.98, 128.45, 128.43, 127.86, 127.71, 123.94, 123.92, 123.70, 123.49, 102.56, 101.76, 66.39, 66.34, 64.82, 64.34, 42.19, 41.62. ESI-MS calcd for $C_{18}H_{17}N_2O_5$ $[M+H]^+$ 341.1132, found 341.1136.

Isobutyl 6-phenyl-2,3-dihydro-4*H*-1,4-oxazine-4-carboxylate (1a-COO*i*Bu)

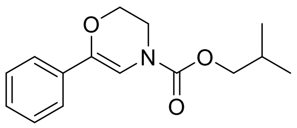

Colorless oil (1.90 g, 29% yield). ^{1}H NMR (400 MHz, $CDCl_3$): (two rotamers: 45/55) δ 7.52-7.47 (m, 2H), 7.33-7.21 (m, 3H), 7.02 (s, 0.45H), 6.84 (s, 0.55H), 4.23-4.18 (m, 2H), 3.99-3.95 (m, 2H), 3.79-3.74 (m, 2H), 2.06-1.92 (m, 1H), 0.99-0.95 (m, 6H). ^{13}C NMR (100 MHz, $CDCl_3$): δ 152.46, 152.27, 138.52, 137.47, 134.24, 134.19, 128.31, 128.26, 127.42, 127.29, 123.42, 123.25, 102.92, 102.25, 72.24, 72.13, 64.73, 64.27, 41.92, 41.30, 27.99, 27.93, 19.11, 19.05. ESI-MS calcd for $C_{15}H_{20}NO_3$ $[M+H]^+$ 262.1438, found 262.1437.

***tert*-Butyl 6-phenyl-2,3-dihydro-4*H*-1,4-oxazine-4-carboxylate (1a-Boc)**

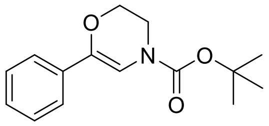

Colorless oil. (2.21g, 34% yield). ^{1}H NMR (400 MHz, $CDCl_3$): (two rotamers: 43/57) δ 7.51-7.47 (m, 2H), 7.33-7.19 (m, 3H), 7.01 (s, 0.43H), 6.78 (s, 0.57H), 4.24-4.18 (m, 2H), 3.76-3.71 (m, 2H), 1.54-1.52 (m, 9H). ^{13}C NMR (100 MHz, $CDCl_3$): δ 151.49, 151.37, 138.21, 136.97, 134.54, 128.39, 127.37, 127.22, 123.49, 123.25, 103.13, 103.02, 81.35, 81.23, 64.93, 64.48, 42.39, 40.95, 28.43. ESI-MS calcd for $C_{15}H_{20}NO_3$ $[M+H]^+$ 262.1438, found 262.1437.

6-Phenyl-4-tosyl-3,4-dihydro-2*H*-1,4-oxazine(1a-Ts)

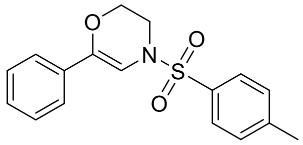

Colorless oil. (1.74 g, 22% yield). ^{1}H NMR (400 MHz, $CDCl_3$): δ 7.69-7.67 (m, 2H), 7.48-7.45 (m, 2H), 7.34-7.25 (m, 5H), 6.71 (s, 1H), 3.79 (t, J = 4.0 Hz, 2H), 3.58 (t, J = 4.0 Hz, 2H), 2.41 (s, 3H). ^{13}C NMR (100 MHz, $CDCl_3$): δ 144.24, 140.82, 133.98, 133.86, 130.07, 128.51, 128.13, 127.52, 123.83, 101.97, 63.48, 43.52, 21.72. ESI-MS calcd for $C_{17}H_{18}NO_3S$ $[M+H]^+$ 316.1002, found 316.1007.

3. Asymmetric Hydrogenation

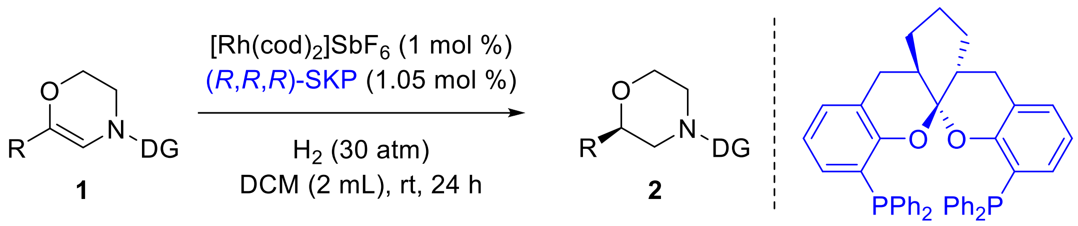

General Procedure: A solution of $[Rh(cod)_2]SbF_6$ (0.002 mmol) and (*R*,*R*,*R*)-SKP (0.0021mmol) in anhydrous and degassed DCM (2 mL) was stirred at room temperature for 30 min. The pre-prepared solution of catalyst was added into substrate **1** (0.2 mmol), which was placed in a 5.0 mL hydrogenation tube equipped with a magnetic stirrer bar. This tube was put into an autoclave and the autoclave was sealed. (The above operation was carried out in a glove box). After purging with hydrogen for three times, the hydrogen pressure was finally adjusted to 30 atm. After stirring at room temperature for 24 h, the reaction mixture was concentrated and purified through a silica gel column with PE/EA = 10/1 as eluent. The enantioselectivity of the product **2** was determined by HPLC using chiral columns. The absolute configuration of the product **2b** was assigned according to X-Ray analysis. Other compounds are considered to have the same configuration as **2b**.

Benzyl (*R*)-2-phenylmorpholine-4-carboxylate (2a)

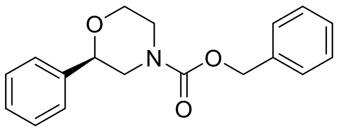

PE/EA = 10/1 as the eluent. Colorless oil (58.8 mg, 99% yield). ^{1}H NMR (400 MHz, $CDCl_3$): δ 7.35-7.28 (m, 10H), 5.24-5.13 (m, 2H), 4.43-4.41 (m, 1H), 4.22-4.02 (m, 3H), 3.68 (t, *J* = 10.8 Hz, 1H), 3.11 (br, 1H), 2.91 (br, 1H). ^{13}C NMR (100 MHz, $CDCl_3$): δ 155.29, 139.13, 136.58, 128.64, 128.58, 128.24, 128.09, 126.22 (2C), 77.90, 67.46, 66.76, 50.17 (br), 43.67 (br). ESI-HRMS: m/z for $C_{18}H_{20}NO_3$ $[M+H]^+$ calcd 298.1438, found 298.1433.

$[\alpha]_D^{25}$ +25.8 (*c* 0.2, CH_2Cl_2). 92% ee. Determined by HPLC analysis using a Daicel Chiralcel IE column (25 cm × 0.46 cm), hexane/isopropanol = 95/5, 0.8 mL/min, 210 nm, t_R (minor) = 16.8 min, t_R (major) = 18.2 min.

Benzyl (*R*)-2-(4-fluorophenyl)morpholine-4-carboxylate (2b)

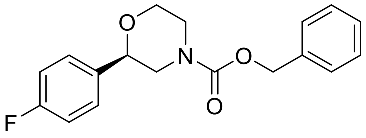

PE/EA = 10/1 as the eluent. Yellow solid (61.8 mg, 98% yield), Mp: 63-65 °C. ^{1}H NMR (400 MHz, $CDCl_3$): δ 7.37-7.31 (m, 7H), 7.03 (t, *J* = 8.8 Hz, 2H), 5.21-5.13 (m, 2H), 4.41-4.39 (m, 1H), 4.18-4.01 (m, 3H), 3.68 (t, *J* = 10.8 Hz, 1H), 3.11 (br, 1H), 2.86 (br, 1H). ^{13}C NMR (100 MHz, $CDCl_3$): δ 163.84 (161.39, d, *J* = 247.5 Hz), 155.29, 136.55, 135.01 (134.98, d, *J* = 3.0 Hz), 128.68, 128.30, 128.15, 128.01 (127.94, d, *J* =

7.1 Hz), 115.60 (115.39, d, *J* = 21.2 Hz), 77.24, 67.54, 66.80, 50.17 (br), 43.70 (br). ^{19}F NMR (376 MHz, $CDCl_3$): *δ* -113.81, -114.02. ESI-HRMS calcd for $C_{18}H_{19}FNO_3$ $[M+H]^+$ 316.1343, found 316.1347.
$[\alpha]_D^{25}$ -2.4 (*c* 0.1, CH_2Cl_2). 91% ee. Determined by HPLC analysis using a Daicel Chiralcel IE column (25 cm × 0.46 cm), hexane/isopropanol = 95/5, 1.0 mL/min, 210 nm, t_R (minor) = 13.1 min, t_R (major) = 14.9 min.

Benzyl (*R*)-2-(4-chlorophenyl)morpholine-4-carboxylate (2c)

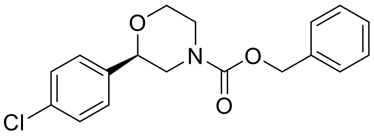

PE/EA = 10/1 as the eluent. White solid (65.0 mg, 98% yield), Mp: 63-65 ^oC. ^{1}H NMR (400 MHz, $CDCl_3$): *δ* 7.36-7.31 (m, 9H), 5.21-5.13 (m, 2H), 4.42-4.40 (m, 1H), 4.20-4.02 (m, 3H), 3.68 (t, *J* = 10.8 Hz, 1H), 3.11 (br, 1H), 2.85 (br, 1H). ^{13}C NMR (100 MHz, $CDCl_3$): *δ* 155.23, 137.64, 136.51, 133.96, 128.74, 128.65, 128.28, 128.12, 127.56, 77.11, 67.53, 66.72, 50.10 (br), 43.64 (br). ESI-HRMS calcd for $C_{18}H_{19}ClNO_3$ $[M+H]^+$ 332.1048, found 332.1043.
$[\alpha]_D^{25}$ +6.8 (*c* 0.9, CH_2Cl_2). 93% ee. Determined by HPLC analysis using a Daicel Chiralcel IE column (25 cm × 0.46 cm), hexane/isopropanol = 90/10, 1.0 mL/min, 210 nm, t_R (minor) = 9.4 min, t_R (major) = 10.7 min.

Benzyl (*R*)-2-(4-bromophenyl)morpholine-4-carboxylate (2d)

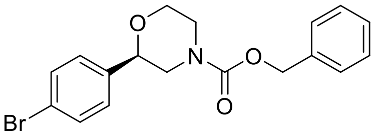

PE/EA = 10/1 as the eluent. Colorless oil (73.0 mg, 97% yield). ^{1}H NMR (400 MHz, $CDCl_3$): *δ* 7.48 (d, *J* = 8.4 Hz, 2H), 7.36-7.32 (m, 5H), 7.25-7.23 (m, 3H), 5.21-5.13 (m, 2H), 4.40-4.38 (m, 1H), 4.21-4.03 (m, 3H), 3.68 (t, *J* = 10.8 Hz, 1H), 3.11 (br, 1H), 2.85 (br, 1H). ^{13}C NMR (100 MHz, $CDCl_3$): *δ* 155.24, 138.16, 136.51, 131.70, 128.66, 128.30, 128.13, 127.89, 122.11, 67.55, 66.73, 49.92 (br), 43.63 (br). ESI-HRMS: m/z for $C_{18}H_{19}BrNO_3$ $[M+H]^+$ calcd 376.0543, found 375.0540.
$[\alpha]_D^{25}$ +40.8 (*c* 0.2, CH_2Cl_2). 92% ee. Determined by HPLC analysis using a Daicel Chiralcel IE column (25 cm × 0.46 cm), hexane/isopropanol = 85/15, 1.0 mL/min, 210 nm, t_R (minor) = 8.8 min, t_R (major) = 10.1 min.

Benzyl (*R*)-2-(1,1'-biphenyl-4-yl)morpholine-4-carboxylate (2e)

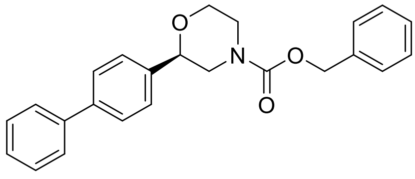

PE/EA = 10/1 as the eluent. Colorless oil (73.2 mg, 98% yield). ^{1}H NMR (400 MHz, $CDCl_3$): *δ* 7.59-7.57 (m, 4H), 7.45-7.32 (m, 10H), 5.22-5.14 (m, 2H), 4.49-4.47 (m, 1H), 4.26-4.04 (m, 3H), 3.72 (t, *J* = 10.8 Hz, 1H), 3.15 (br, 1H), 2.96 (br, 1H). ^{13}C NMR (100 MHz, $CDCl_3$): *δ* 155.30, 141.18, 140.80, 138.12, 136.58, 128.87, 128.65, 128.26, 128.12, 127.47, 127.33, 127.20, 126.67, 77.66, 67.50, 66.78, 50.10 (br), 43.71 (br). ESI-HRMS: m/z for $C_{24}H_{24}NO_3$ $[M+H]^+$ calcd 374.1751, found 374.1756.
$[\alpha]_D^{25}$ +28.6 (*c* 0.2, CH_2Cl_2). 91% ee. Determined by HPLC analysis using a

Daicel Chiralcel IE column (25 cm × 0.46 cm), hexane/isopropanol = 85/15, 1.0 mL/min, 210 nm, t_R (minor) = 11.8 min, t_R (major) = 16.8 min.

Benzyl (*R*)-2-(4-(trifluoromethyl)phenyl)morpholine-4-carboxylate (2f)

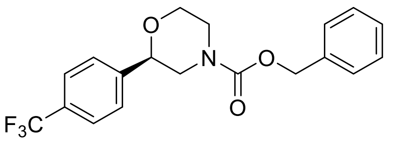

PE/EA = 10/1 as the eluent. Colorless oil (72.3 mg, 99% yield). ^{1}H NMR (400 MHz, $CDCl_3$): δ 7.62 (d, J = 8.0 Hz, 2H), 7.49 (d, J = 7.6 Hz, 2H), 7.37-7.33 (m, 5H), 5.21-5.14 (m, 2H), 4.51-4.49 (m, 1H), 4.24-4.04 (m, 3H), 3.71 (t, J = 10.8 Hz, 1H), 3.13 (br, 1H), 2.86 (br, 1H). ^{13}C NMR (100 MHz, $CDCl_3$): δ 155.29, 143.06 (143.05, q, J = 1.0 Hz), 136.50, 130.89 (130.58, 130,27, 129,95, q, J = 31.3 Hz), 128.71, 128.36, 128.19, 126.51, 125.63 (125.59, 125.55, 125.51, q, J = 4.0 Hz), 125.51 (122.81, q, J = 272.7 Hz), 67.64, 66.77, 50.21 (br), 43.58 (br). ^{19}F NMR (376 MHz, $CDCl_3$): δ -62.59. ESI-HRMS: m/z for $C_{19}H_{19}F_3NO_3$ $[M+H]^+$ calcd 366.1312, found 366.1316.
$[\alpha]^{25}_{D}$ +37.3 (*c* 0.1, CH_2Cl_2). 94% ee. Determined by HPLC analysis using a Daicel Chiralcel IE column (25 cm × 0.46 cm), hexane/isopropanol = 95/5, 1.0 mL/min, 210 nm, t_R (minor) = 9.8 min, t_R (major) = 11.1 min.

Benzyl (*R*)-2-(*p*-tolyl)morpholine-4-carboxylate (2g)

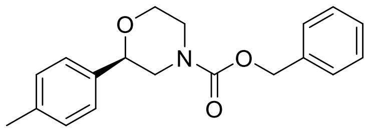

PE/EA = 10/1 as the eluent. Colorless oil (61.0 mg, 98% yield). ^{1}H NMR (400 MHz, $CDCl_3$): δ 7.36-7.32 (m, 5H), 7.25-7.24 (m, 2H), 7.17-7.15 (m, 2H), 5.20-5.13 (m, 2H), 4.40-4.39 (m, 1H), 4.19-4.02 (m, 3H), 3.69 (t, J = 10.8 Hz, 1H), 3.12 (br, 1H), 2.91 (br, 1H), 2.34 (s, 3H). ^{13}C NMR (100 MHz, $CDCl_3$): δ 155.28, 137.95, 136.59, 136.18, 129.23, 128.62, 128.21, 128.07, 126.16, 77.79, 67.43, 66.74, 50.19, 43.66, 21.25. ESI-HRMS: m/z for $C_{19}H_{22}NO_3$ $[M+H]^+$ calcd 312.1594, found 312.1598.
$[\alpha]^{25}_{D}$ +72.5 (c 0.2, CH_2Cl_2). 91% ee. Determined by HPLC analysis using a Daicel Chiralcel IE column (25 cm × 0.46 cm), hexane/isopropanol = 95/5, 1.0 mL/min, 210 nm, t_R (minor) = 14.3 min, t_R (major) = 15.6 min.

Benzyl (*R*)-2-(4-methoxyphenyl)morpholine-4-carboxylate (2h)

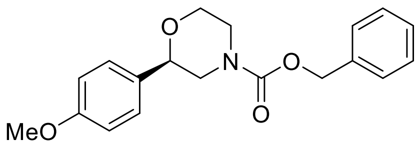

PE/EA = 10/1 as the eluent. Colorless oil (64.8 mg, 99% yield). ^{1}H NMR (400 MHz, $CDCl_3$): δ 7.36-7.24 (m, 7H), 6.88 (d, J = 8.4 Hz, 2H), 5.20-5.13 (m, 2H), 4.38-4.36 (m, 1H), 4.17-4.01 (m, 3H), 3.78 (s, 3H), 3.67 (t, J = 10.4 Hz, 1H), 3.11 (br, 1H), 2.90 (br, 1H). ^{13}C NMR (100 MHz, $CDCl_3$): δ 159.54, 155.27, 136.58, 131.33, 128.62, 128.21, 128.07, 127.53, 113.96, 77.54, 67.42, 66.74, 55.35, 50.12, 43.67. ESI-HRMS calcd for $C_{19}H_{22}NO_4$ $[M+H]^+$ 328.1543, found 328.1547.
$[\alpha]^{25}_{D}$ +28.7 (*c* 0.5, CH_2Cl_2). 91% ee. Determined by HPLC analysis using a Daicel Chiralcel IE column (25 cm × 0.46 cm), hexane/isopropanol = 95/5, 1.0

mL/min, 210 nm, t_R (minor) = 31.0 min, t_R (major) = 38.0 min.

Benzyl (*R*)-2-(3-fluorophenyl)morpholine-4-carboxylate (2i)

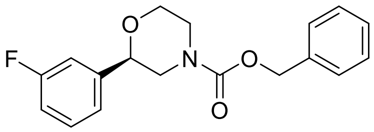

PE/EA = 10/1 as the eluent. Colorless oil (61.9 mg, 98% yield). ^1H NMR (400 MHz, $CDCl_3$): δ 7.37-7.32 (m, 7H), 7.06-7.02 (m, 2H), 5.21-5.13 (m, 2H), 4.42-4.40 (m, 1H), 4.18-4.02 (m, 3H), 3.69 (t, J = 10.8 Hz, 1H), 3.12 (br, 1H), 2.87 (br, 1H). ^{13}C NMR (100 MHz, $CDCl_3$): δ 163.82 (161.37, d, J = 247.5 Hz), 155.27, 136.54, 135.00 (134.97, d, J = 3.0 Hz), 128.66, 128.29, 128.13, 128.00 (127.92, d, J = 8.1 Hz), 115.58 (115.37, d, J = 21.2 Hz), 77.21, 67.52, 66.78, 50.23 (br), 43.63 (br). ^{19}F NMR (376 MHz, $CDCl_3$): δ -113.86, -114.07. ESI-HRMS calcd for $C_{18}H_{19}FNO_3$ $[M+H]^+$ 316.1343, found 316.1347.

$[\alpha]_D^{25}$ +6.0 (c 0.2, CH_2Cl_2). 93% ee. Determined by HPLC analysis using a Daicel Chiralcel IE column (25 cm × 0.46 cm), hexane/isopropanol = 90/10, 1.0 mL/min, 210 nm, t_R (minor) = 9.1 min, t_R (major) = 10.1 min.

Benzyl (*R*)-2-(3-chlorophenyl)morpholine-4-carboxylate (2j)

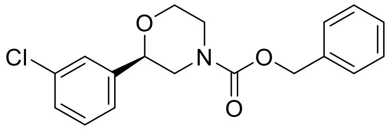

PE/EA = 10/1 as the eluent. Colorless oil (65.1 mg, 98% yield). ^1H NMR (400 MHz, $CDCl_3$): δ 7.38-7.31 (m, 6H), 7.27-7.21 (m, 3H), 5.21-5.13 (m, 2H), 4.41-4.39 (m, 1H), 4.21-4.01 (m, 3H), 3.67 (t, J = 11.2 Hz, 1H), 3.10 (br, 1H), 2.85 (br, 1H). ^{13}C NMR (100 MHz, $CDCl_3$): δ 155.23, 141.13, 136.50, 134.55, 129.85, 128.66, 128.34, 128.30, 128.14, 126.41, 124.31, 77.08, 67.55, 66.73, 50.05, 43.66. ESI-HRMS calcd for $C_{18}H_{19}ClNO_3$ $[M+H]^+$ 332.1048, found 332.1045.

$[\alpha]_D^{25}$ +6.0 (c 0.2, CH_2Cl_2). 92% ee. Determined by HPLC analysis using a Daicel Chiralcel IE column (25 cm × 0.46 cm), hexane/isopropanol = 95/5, 1.0 mL/min, 210 nm, t_R (minor) = 11.9 min, t_R (major) = 14.0 min.

Benzyl (*R*)-2-(3-bromophenyl)morpholine-4-carboxylate (2k)

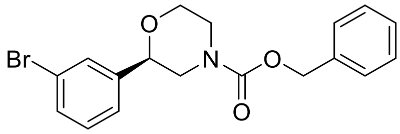

PE/EA = 10/1 as the eluent. Colorless oil (74.5 mg, 99% yield). ^1H NMR (400 MHz, $CDCl_3$): δ 7.54 (s, 1H), 7.43 (d, J = 7.6 Hz, 1H), 7.37-7.31 (m, 5H), 7.27-7.17 (m, 2H), 5.21-5.13 (m, 2H), 4.40-4.38 (m, 1H), 4.21-4.01 (m, 3H), 3.66 (t, J = 11.2 Hz, 1H), 3.10 (br, 1H), 2.85 (br, 1H). ^{13}C NMR (100 MHz, $CDCl_3$): δ 155.22, 141.37, 136.49, 131.27, 130.12, 129.31, 128.65, 128.29, 128.14, 124.77, 122.73, 77.00, 67.55, 66.72, 50.00, 43.62. ESI-HRMS: m/z for $C_{18}H_{18}BrNO_3$ $[M+H]^+$ calcd 376.0543, found 376.0546.

$[\alpha]_D^{25}$ +13.8 (c 0.2, CH_2Cl_2). 93% ee. Determined by HPLC analysis using a Daicel Chiralcel IE column (25 cm × 0.46 cm), hexane/isopropanol = 90/10, 1.0 mL/min, 210 nm, t_R (minor) = 9.8 min, t_R (major) = 11.1 min.

Benzyl (*R*)-2-(3-methoxyphenyl)morpholine-4-carboxylate (2l)

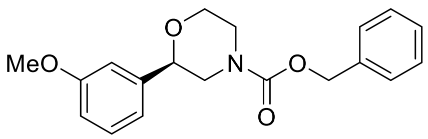

PE/EA = 10/1 as the eluent. Colorless oil (64.8 mg, 99% yield). ^{1}H NMR (400 MHz, $CDCl_3$): δ 7.36-7.23 (m, 6H), 6.92- 6.82 (m, 3H), 5.20-5.13 (m, 2H), 4.41-4.39 (m, 1H), 4.22-4.01 (m, 3H), 3.78 (s, 3H), 3.67 (t, J = 10.8 Hz, 1H), 3.11 (br, 1H), 2.90 (br, 1H). ^{13}C NMR (100 MHz, $CDCl_3$): δ 159.83, 155.29, 140.70, 136.57, 129.62, 128.64, 128.24, 128.09, 118.47, 113.89, 111.60, 77.79, 67.47, 66.73, 55.35, 50.15, 43.72. ESI-HRMS calcd for $C_{19}H_{22}NO_4$ $[M+H]^+$ 328.1543, found 328.1547.

$[\alpha]_D^{25}$ +76.7 (c 0.2, CH_2Cl_2). 94% ee. Determined by HPLC analysis using a Daicel Chiralcel IE column (25 cm × 0.46 cm), hexane/isopropanol = 90/10, 1.0 mL/min, 210 nm, t_R (minor) = 14.7 min, t_R (major) = 18.2 min.

Benzyl (*R*)-2-(2-fluorophenyl)morpholine-4-carboxylate (2m)

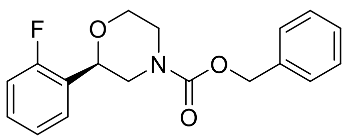

PE/EA = 10/1 as the eluent. Colorless oil (61.8 mg, 98% yield). ^{1}H NMR (400 MHz, $CDCl_3$): δ 7.50-7.46 (m, 1H), 7.37-7.26 (m, 6H), 7.15 (t, J = 7.6 Hz, 1H), 7.03 (t, J = 9.2 Hz, 1H), 5.20-5.14 (m, 2H), 4.75-4.73 (m, 1H), 4.24 (br, 1H), 4.04 (br, 2H), 3.72 (t, J = 10.8 Hz, 1H), 3.11 (br, 1H), 2.87 (t, J = 10.8 Hz, 1H). ^{13}C NMR (100 MHz, $CDCl_3$): δ 160.98 (158.44, d, J = 256.5 Hz), 155.14, 136.58, 129.70 (129.61, d, J = 9.1 Hz), 128.63, 128.20, 128.03, 127.71 (127.67, d, J = 4.0 Hz), 126.37 (126.24, d, J = 13.1 Hz), 124.46 (124.43, d, J = 3.0 Hz), 115.50 (115.29, d, J = 21.2 Hz), 72.07, 67.46, 66.97, 49.06 (br), 43.73 (br). ^{19}F NMR (376 MHz, $CDCl_3$): δ -118.25, -118.89. ESI-HRMS calcd for $C_{18}H_{19}FNO_3$ $[M+H]^+$ 316.1343, found 316.1342.

$[\alpha]_D^{25}$ +10.1 (c 0.3, CH_2Cl_2). 99% ee. Determined by HPLC analysis using a Daicel Chiralcel IE column (25 cm × 0.46 cm), hexane/isopropanol = 90/10, 1.0 mL/min, 210 nm, t_R (minor) = 10.0 min, t_R (major) = 10.8 min.

Benzyl (*R*)-2-(2-chlorophenyl)morpholine-4-carboxylate (2n)

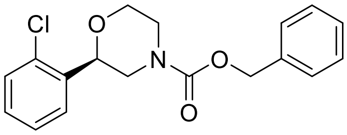

PE/EA = 10/1 as the eluent. Colorless oil (64.9 mg, 98% yield). ^{1}H NMR (400 MHz, $CDCl_3$): δ 7.57-7.54 (m, 1H), 7.37-7.21 (m, 8H), 5.22-5.14 (m, 2H), 4.81-4.79 (m, 1H), 4.38-4.36 (m, 1H), 4.07-4.05 (m, 2H), 3.75 (t, J = 10.8 Hz, 1H), 3.11 (br, 1H), 2.72 (t, J = 10.8 Hz, 1H). ^{13}C NMR (100 MHz, $CDCl_3$): δ 155.15, 136.78, 136.57, 131.83, 129.42, 129.15, 128.59, 128.16, 127.96, 127.54, 127.21, 74.96, 67.43, 66.99, 49.01, 43.67. ESI-HRMS calcd for $C_{18}H_{18}ClNO_3$ $[M+H]^+$ 332.1048, found 332.1043.

$[\alpha]_D^{25}$ -31.6 (c 0.5, CH_2Cl_2). 99% ee. Determined by HPLC analysis using a Daicel Chiralcel IE column (25 cm × 0.46 cm), hexane/isopropanol = 98/2, 1.0 mL/min, 210 nm, t_R (minor) = 20.9 min, t_R (major) = 22.9 min.

Benzyl (*R*)-2-(2-*o*-tolyl)morpholine-4-carboxylate (2o)

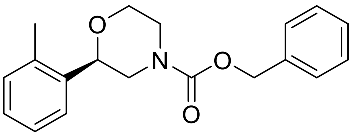

PE/EA = 10/1 as the eluent. Colorless oil (60.9 mg, 98% yield). ^{1}H NMR (400 MHz, $CDCl_3$): *δ* 7.45-7.33 (m, 1H), 7.35-7.30 (m, 5H), 7.22-7.12 (m, 3H), 5.20-5.13 (m, 2H), 4.58-4.56 (m, 1H), 4.18-4.03 (m, 3H), 3.71 (t, *J* = 10.0 Hz, 1H), 3.13 (br, 1H), 2.86 (br, 1H), 2.32 (s, 3H). ^{13}C NMR (100 MHz, $CDCl_3$): *δ* 155.20, 137.20, 136.56, 134.74, 130.43, 128.60, 128.20, 128.04, 127.95, 126.34, 125.81, 75.27, 67.43, 66.98, 49.25, 43.80, 19.06. ESI-HRMS: m/z for $C_{19}H_{22}NO_3$ $[M+H]^+$ calcd 312.1594, found 312.1598.
$[\alpha]_D^{25}$ -66.1 (*c* 0.2, CH_2Cl_2). 99% ee. Determined by HPLC analysis using a Daicel Chiralcel IC-3 column (25 cm × 0.46 cm), hexane/isopropanol = 90/10, 0.5 mL/min, 210 nm, t_R (major) = 19.3 min, t_R (minor) = 21.4 min.

Benzyl (*R*)-2-(2-methoxyphenyl)morpholine-4-carboxylate (2p)

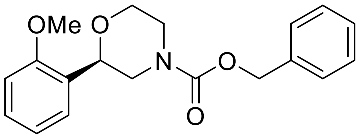

PE/EA = 10/1 as the eluent. Colorless oil (63.5 mg, 97% yield). ^{1}H NMR (400 MHz, $CDCl_3$): *δ* 7.46-7.44 (m, 1H), 7.37-7.24 (m, 6H), 6.97 (t, *J* = 7.6 Hz, 1H), 6.85 (d, *J* = 8.0 Hz, 1H), 5.22-5.13 (m, 2H), 4.80-4.78 (m, 1H), 4.31-4.29 (m, 1H), 4.04 (br, 2H), 3.78-3.70 (m, 4H), 3.13-3.08 (m, 1H), 2.75 (br, 1H). ^{13}C NMR (100 MHz, $CDCl_3$): *δ* 155.93, 155.25, 136.76, 128.90, 128.57, 128.09, 127.81, 127.55, 126.62, 120.80, 110.21, 72.78, 67.26, 66.99, 55.34, 49.27, 43.90. ESI-HRMS calcd for $C_{19}H_{22}NO_4$ $[M+H]^+$ 328.1543, found 328.1540.
$[\alpha]_D^{25}$ -29.4 (*c* 0.5, CH_2Cl_2). 99% ee. Determined by HPLC analysis using a Daicel Chiralcel IC-3 column (25 cm × 0.46 cm), hexane/isopropanol = 90/10, 0.5 mL/min, 210 nm, t_R (major) = 24.4 min, t_R (minor) = 30.0 min.

Benzyl (*R*)-2-(3,4-dichlorophenyl)morpholine-4-carboxylate (2q)

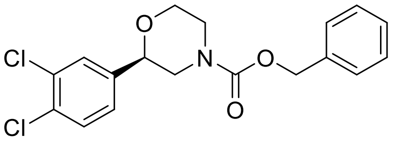

PE/EA = 10/1 as the eluent. Colorless oil (70.0 mg, 96% yield). ^{1}H NMR (400 MHz, $CDCl_3$): *δ* 7.48 (s, 1H), 7.42-7.31 (m, 6H), 7.18 (d, *J* = 8.0 Hz, 1H), 5.21-5.14 (m, 2H), 4.40-4.38 (m, 1H), 4.21-4.01 (m, 3H), 3.67 (t, *J* = 11.2 Hz, 1H), 3.10 (br, 1H), 2.82 (br, 1H). ^{13}C NMR (100 MHz, $CDCl_3$): *δ* 155.22, 139.35, 136.45, 132.81, 132.14, 130.55, 128.69, 128.35, 128.26, 128.17, 125.47, 76.45, 67.63, 66.74, 49.84, 43.59. ESI-HRMS calcd for $C_{18}H_{18}Cl_2NO_3$ $[M+H]^+$ 366.0658, found 366.0656.
$[\alpha]_D^{25}$ +47.2 (*c* 0.5, CH_2Cl_2). 94% ee. Determined by HPLC analysis using a Daicel Chiralcel IE column (25 cm × 0.46 cm), hexane/isopropanol = 90/10, 1.0 mL/min, 210 nm, t_R (minor) = 10.1 min, t_R (major) = 12.2 min.

Benzyl (*R*)-2-(3,4-dimethoxyphenyl)morpholine-4-carboxylate (2r)

PE/EA = 10/1 as the eluent. Colorless oil (69.3 mg, 97% yield). ^{1}H NMR (400 MHz, $CDCl_3$): *δ* 7.37-7.31 (m, 5H), 6.91-6.83 (m, 3H), 5.21-5.13 (m, 2H), 4.38 (br, 1H), 4.20-4.02 (m, 3H), 3.89 (s, 3H), 3.87 (s, 3H), 3.72-3.66 (m, 1H), 3.14

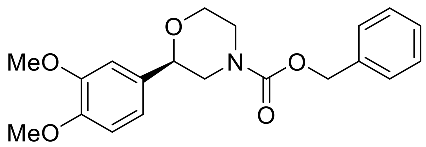

(br, 1H), 2.93 (br, 1H). ^{13}C NMR (100 MHz, $CDCl_3$): δ 154.92, 148.79, 148.61, 136.32, 131.52, 128.31, 127.90, 127.75, 118.27, 110.86, 109.15, 77.32, 67.08, 66.38, 55.66, 55.63, 49.95, 43.34. ESI-HRMS calcd for $C_{20}H_{24}NO_5$ $[M+H]^+$ 358.1649, found 358.1649.

$[\alpha]_D^{25}$ +28.8 (*c* 0.5, CH_2Cl_2). 88% ee. Determined by HPLC analysis using a Daicel Chiralcel IE column (25 cm × 0.46 cm), hexane/isopropanol = 85/15, 1.0 mL/min, 210 nm, t_R (minor) = 47.3 min, t_R (major) = 49.7 min.

Benzyl (*R*)-2-(naphthalen-2-yl)morpholine-4-carboxylate (2s)

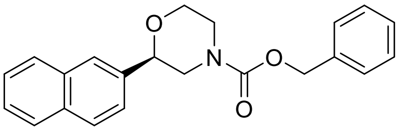

PE/EA = 10/1 as the eluent. Colorless oil (67.4 mg, 97% yield). ^{1}H NMR (400 MHz, $CDCl_3$): δ 7.84-7.82 (m, 4H), 7.48-7.46 (m, 3H), 7.34-7.32 (m, 5H), 5.22-5.15 (m, 2H), 4.60-4.59 (m, 1H), 4.32-4.07 (m, 3H), 3.75 (t, *J* = 10.8 Hz, 1H), 3.17 (br, 1H), 2.99 (br, 1H). ^{13}C NMR (100 MHz, $CDCl_3$): δ 155.30, 136.58, 136.54, 133.29, 133.22, 128.64, 128.33, 128.25, 128.12, 128.09, 127.77, 126.30, 126.17, 125.16, 124.04, 77.92, 67.49, 66.82, 50.20, 43.62. ESI-HRMS: m/z for $C_{22}H_{22}NO_3$ $[M+H]^+$ calcd 348.1594, found 348.1597.

$[\alpha]_D^{25}$ +104.7 (*c* 0.2, CH_2Cl_2). 93% ee. Determined by HPLC analysis using a Daicel Chiralcel IE column (25 cm × 0.46 cm), hexane/isopropanol = 90/10, 1.0 mL/min, 210 nm, t_R (minor) = 14.7 min, t_R (major) = 16.2 min.

Benzyl (*S*)-2-(thiophen-2-yl)morpholine-4-carboxylate (2t)

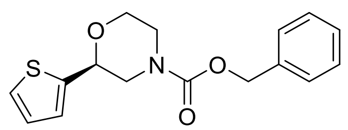

PE/EA = 10/1 as the eluent. Yellow oil (58.2 mg, 96% yield). ^{1}H NMR (400 MHz, $CDCl_3$): δ 7.36-7.31 (m, 5H), 7.27-7.25 (m, 1H), 7.01-6.96 (m, 2H), 5.20-5.13 (m, 2H), 4.70-4.68 (m, 1H), 4.27-3.97 (s, 3H), 3.68 (t, *J* = 10.8 Hz, 1H), 3.12 (br, 2H). ^{13}C NMR (100 MHz, $CDCl_3$): δ 155.16, 141.65, 136.46, 128.63, 128.26, 128.11, 126.71, 125.31, 124.60, 73.68, 67.51, 66.54, 49.85, 43.55. ESI-HRMS: m/z for $C_{16}H_{18}NO_3S$ $[M+H]^+$ calcd 304.1002, found 304.1006.

$[\alpha]_D^{25}$ +51.0 (*c* 0.1, CH_2Cl_2). 88% ee. Determined by HPLC analysis using a Daicel Chiralcel IE column (25 cm × 0.46 cm), hexane/isopropanol = 90/10, 1.0 mL/min, 210 nm, t_R (major) = 11.2 min, t_R (minor) = 12.5 min.

Benzyl (*R*)-2-(*tert*-butyl)morpholine-4-carboxylate (2u)

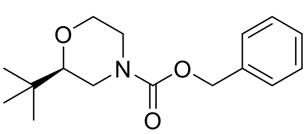

PE/EA = 10/1 as the eluent. Colorless oil (54.9 mg, 99% yield). ^{1}H NMR (400 MHz, $CDCl_3$): δ 7.36-7.28 (m, 5H), 5.15 (s, 2H), 4.04-3.86 (m, 3H), 3.47 (t, *J* = 10.8 Hz, 1H), 2.99-2.94 (m, 2H), 2.72 (br, 1H), 0.92 (s, 9H). ^{13}C NMR (100 MHz, $CDCl_3$): δ 155.52, 136.78, 128.57, 128.09, 127.92, 83.25, 67.22, 66.82, 44.47, 43.90, 33.44, 26.03. ESI-HRMS: m/z for $C_{16}H_{24}NO_3$

[M+H]+ calcd 278.1751, found 278.1753.
$[\alpha]_D^{25}$ +0.8 (*c* 0.5, CH_2Cl_2). 81% ee. Determined by HPLC analysis using a Daicel Chiralcel IA column (25 cm × 0.46 cm), hexane/isopropanol = 95/5, 0.8 mL/min, 210 nm, t_R (minor) = 6.8 min, t_R (major) = 7.2 min.

Benzyl (*R*)-2-isopropylmorpholine-4-carboxylate (2v)

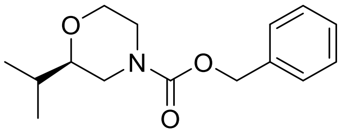

PE/EA = 10/1 as the eluent. Colorless oil (52.1 mg, 99% yield). ^{1}H NMR (400 MHz, $CDCl_3$): *δ* 7.30-7.21 (m, 5H), 5.07 (s, 2H), 3.97-3.80 (m, 3H), 3.40 (t, *J* = 12.0 Hz, 1H), 2.99 -2.89 (m, 2H), 2.61 (br, 1H), 1.65-1.57 (m, 1H), 0.89-0.83 (m, 6H). ^{13}C NMR (100 MHz, $CDCl_3$): *δ* 155.43, 136.72, 128.58, 128.12, 127.96, 80.68, 67.27, 66.53, 46.44, 43.89, 31.10, 18.39. ESI-HRMS: m/z for $C_{15}H_{22}NO_3$ $[M+H]^+$ calcd 264.1594, found 264.1596.
$[\alpha]_D^{25}$ +13.8 (*c* 0.75, CH_2Cl_2). 58% ee. Determined by HPLC analysis using a Daicel Chiralcel OZ-H column (25 cm × 0.46 cm), hexane/isopropanol = 99/1, 0.8 mL/min, 210 nm, t_R (minor) = 16.9 min, t_R (major) = 18.7 min.

Benzyl (*R*)-2-ethylmorpholine-4-carboxylate (2w)

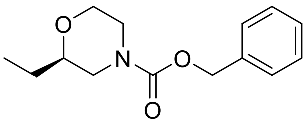

PE/EA = 10/1 as the eluent. Colorless oil (49.4 mg, 99% yield). ^{1}H NMR (400 MHz, $CDCl_3$): *δ* 7.38-7.28 (m, 5H), 5.18-5.11 (m, 2H), 4.00-3.87 (m, 3H), 3.50 (t, *J* = 10.8 Hz, 1H), 3.26 (br, 1H), 2.99 (br, 1H), 2.64 (br, 1H), 1.55-1.41 (m, 2H), 0.95 (t, *J* = 7.6 Hz, 3H). ^{13}C NMR (100 MHz, $CDCl_3$): *δ* 155.27, 136.65, 128.55, 128.11, 127.97, 77.04, 67.26, 66.40, 48.41, 43.84, 26.19, 9.65. ESI-HRMS: m/z for $C_{14}H_{19}NO_3$ $[M+H]^+$ calcd 250.1438, found 250.1437.
$[\alpha]_D^{25}$ +62.3 (*c* 0.2, CH_2Cl_2). 91% ee. Determined by HPLC analysis using a Daicel Chiralcel OJ-H column (25 cm × 0.46 cm), hexane/isopropanol = 95/5, 0.8 mL/min, 210 nm, t_R (major) = 11.5 min, t_R (minor) = 12.4 min.

Benzyl 3-phenylmorpholine-4-carboxylate (2x)

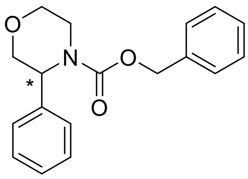

PE/EA = 10/1 as the eluent. Colorless oil (58.9 mg, 99% yield). ^{1}H NMR (400 MHz, $CDCl_3$): *δ* 7.38-7.14 (m, 10H), 5.14-5.06 (m, 3H), 4.29-4.26 (d, *J* = 12.0 Hz, 1H), 3.80-3.76 (m, 3H), 3.54-3.47 (td, *J* = 12.0 Hz, *J* = 4.0 Hz, 1H), 3.14-3.06 (td, *J* = 12.0 Hz, *J* = 4.0 Hz, 1H). ^{13}C NMR (100 MHz, $CDCl_3$): *δ* 155.53, 138.95, 136.51, 128.59, 128.20, 128.06, 127.80, 127.44, 69.05, 67.56, 67.05, 53.56, 40.20. ESI-HRMS: m/z for $C_{18}H_{20}NO_3$ $[M+H]^+$ calcd 298.1438, found 298.1439.
$[\alpha]_D^{25}$ -53.4 (*c* 0.75, CH_2Cl_2). 28% ee. Determined by HPLC analysis using a Daicel Chiralcel IA column (25 cm × 0.46 cm), hexane/isopropanol = 90/10, 1.0 mL/min, 210 nm, t_R (major) = 9.3 min, t_R (minor) = 10.1 min.

Benzyl 2-(4-bromophenyl)-3-methylmorpholine-4-carboxylate (2y)[1]

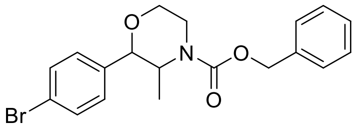

No hydrogenative prodcut was obtained under optimized reaction conditions. PE/EA = 10/1 as the eluent. ^{1}H NMR (400 MHz, $CDCl_3$): *δ* 7.48-7.15 (m, 9H), 5.29-5.14 (m, 2H), 4.62-4.59 (m, 1H), 4.42-4.26 (m, 1H), 4.13-4.01 (m, 1H), 3.92-3.81 (m, 1H), 3.74-3.63 (m, 1H), 3.33-3.20 (m, 1H), 0.89-0.86 (t, *J* = 4.0 Hz, 3H).

4-Nitrobenzyl (*R*)-2-phenylmorpholine-4-carboxylate (2a-NO_2)

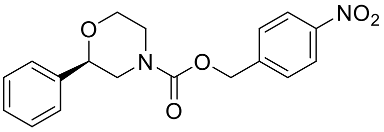

PE/EA = 10/1 as the eluent. Yellow oil (66.7 mg, 98% yield). ^{1}H NMR (400 MHz, $CDCl_3$): *δ* 8.22 (d, *J* = 8.4 Hz, 2H), 7.51 (d, *J* = 8.4 Hz, 2H), 7.37-7.31 (m, 5H), 5.30-5.21 (m, 2H), 4.46 (d, *J* = 10.8 Hz, 1H), 4.23-4.05 (m, 3H), 3.72 (t, *J* = 12.0 Hz, 1H), 3.21-3.12 (m, 1H), 3.00-2.90 (m, 1H). ^{13}C NMR (100 MHz, $CDCl_3$): *δ* 154.73, 147.71, 143.93, 138.90, 128.62, 128.27, 126.17, 123.89, 77.86, 66.67, 65.96, 50.12, 43.69. ESI-HRMS: m/z for $C_{18}H_{19}N_2O_5$ $[M+H]^+$ calcd 343.1288, found 343.1285. $[\alpha]_D^{25}$ -70.0 (*c* 0.2, CH_2Cl_2). 26% ee. Determined by HPLC analysis using a Daicel Chiralcel OX column (25 cm × 0.46 cm), hexane/isopropanol = 90/10, 0.8 mL/min, 254 nm, t_R (minor) = 39.2 min, t_R (major) = 42.9 min.

Isobutyl (*R*)-2-phenylmorpholine-4-carboxylate (2a-COO*i*Bu)

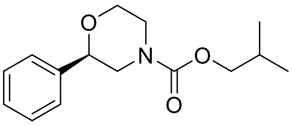

PE/EA = 10/1 as the eluent. Colorless oil (51.7 mg, 99% yield). ^{1}H NMR (400 MHz, $CDCl_3$): *δ* 7.37-7.30 (m, 5H), 4.43 (d, *J* = 10.0 Hz, 1H), 4.20-3.87 (m, 5H), 3.70 (t, *J* = 11.6 Hz, 1H), 3.11 (br, 1H), 2.89 (br, 1H), 2.00-1.90 (m, 1H), 0.94 (d, J = 6.8 Hz, 6H). ^{13}C NMR (100 MHz, $CDCl_3$): *δ* 155.56, 139.21, 128.56, 128.19, 126.17, 77.93, 71.83, 66.78, 50.14, 43.51, 28.06, 19.19. ESI-HRMS: m/z for $C_{15}H_{22}NO_3$ $[M+H]^+$ calcd 264.1594, found 264.1597. $[\alpha]_D^{25}$ +115.0 (*c* 0.2, CH_2Cl_2). 89% ee. Determined by HPLC analysis using a Daicel Chiralcel OD column (25 cm × 0.46 cm), hexane/isopropanol = 90/10, 1.0 mL/min, 210 nm, t_R (minor) = 14.4 min, t_R (major) = 23.9 min.

***tert*-Butyl (*R*)-2-phenylmorpholine-4-carboxylate (2a-Boc)**

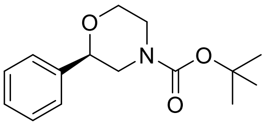

PE/EA = 10/1 as the eluent. Colorless oil (51.0 mg, 98% yield). ^{1}H NMR (400 MHz, $CDCl_3$): *δ* 7.39-7.30 (m, 5H), 4.41 (d, *J* = 9.6 Hz, 1H), 4.20-3.95 (m, 3H), 3.68 (td, *J* = 11.6, 1.6 Hz, 1H), 3.04 (br, 1H), 2.84 (br, 1H), 1.48 (s, 9H). ESI-HRMS: m/z for $C_{15}H_{22}NO_3$ $[M+H]^+$ calcd 264.1594, found 264.1591. $[\alpha]_D^{25}$ +113.5 (*c* 0.2, CH_2Cl_2). 75% ee. Determined by HPLC analysis using a Daicel Chiralcel IC-3 column (25 cm × 0.46 cm), hexane/isopropanol = 90/10, 0.5 mL/min, 210 nm, t_R (major) = 13.3 min, t_R (minor) = 14.0 min.

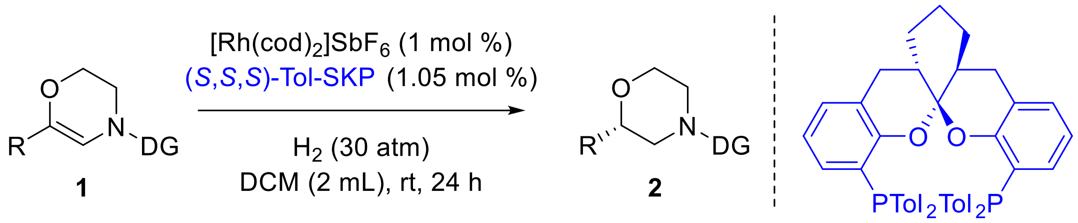

Another ligand (*S*,*S*,*S*)-Tol-SKP bearing electron-donating 4-methyl groups has been tested in the asymmetric hydrogenation of model substrate **1a** and di-OMe-substituted substrate **1r**. The corresponding product **2a** was obtained with 82% yield and 84% ee, while **2r** was obtained with 99% yield and 34% ee.

4. Deuteration Experiment

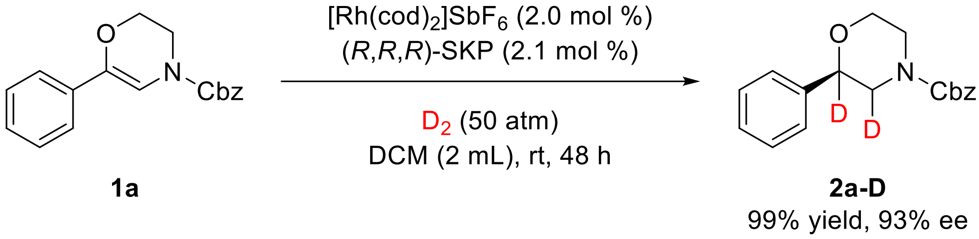

(R,R,R)-SKP ligand (1.18 mg, 0.0042 mmol) and $[Rh(cod)_2]SbF_6$ (2.22 mg, 0.004 mmol) were dissolved in anhydrous and degassed DCM (2 mL) under nitrogen. The mixture was allowed to stir for 30 min at room temperature. The substrate (59.1 mg, 0.2 mmol) was placed in a 5.0 mL tube equipped with a magnetic stirrer bar. This tube was placed in an autoclave. The pre-prepared solution of catalyst was added under a nitrogen atmosphere. After purging with hydrogen for three times, the hydrogen pressure was finally pressurized to 50 atm. The reaction mixture was vigorously stirred at room temperature for 48 h. The conversion of the product was determined by ^{1}H NMR spectroscopic analysis of the crude reaction mixture and the yield was calculated after isolation by flash chromatography.

Benzyl (2*R*)-2,3-*d*$_2$-2-phenylmorpholine-4-carboxylate (2a-D)

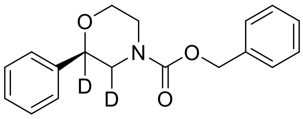

PE/EA = 10/1 as the eluent. Colorless oil (59.3 mg, 99% yield). ^{1}H NMR (400 MHz, $CDCl_3$): δ 7.36-7.30 (m, 10H), 5.21-5.13 (m, 2H), 4.03 (br, 2H), 3.70 (t, J = 10.8 Hz, 1H), 3.12 (br, 1H), 2.92-2.87 (m, 1H).

93% ee. Determined by HPLC analysis using a Daicel Chiralcel IE column (25 cm × 0.46 cm), hexane/isopropanol = 95/5, 0.8 mL/min, 210 nm, t_R (minor) = 17.1 min, t_R (major) = 18.4 min.

5. Applicatons

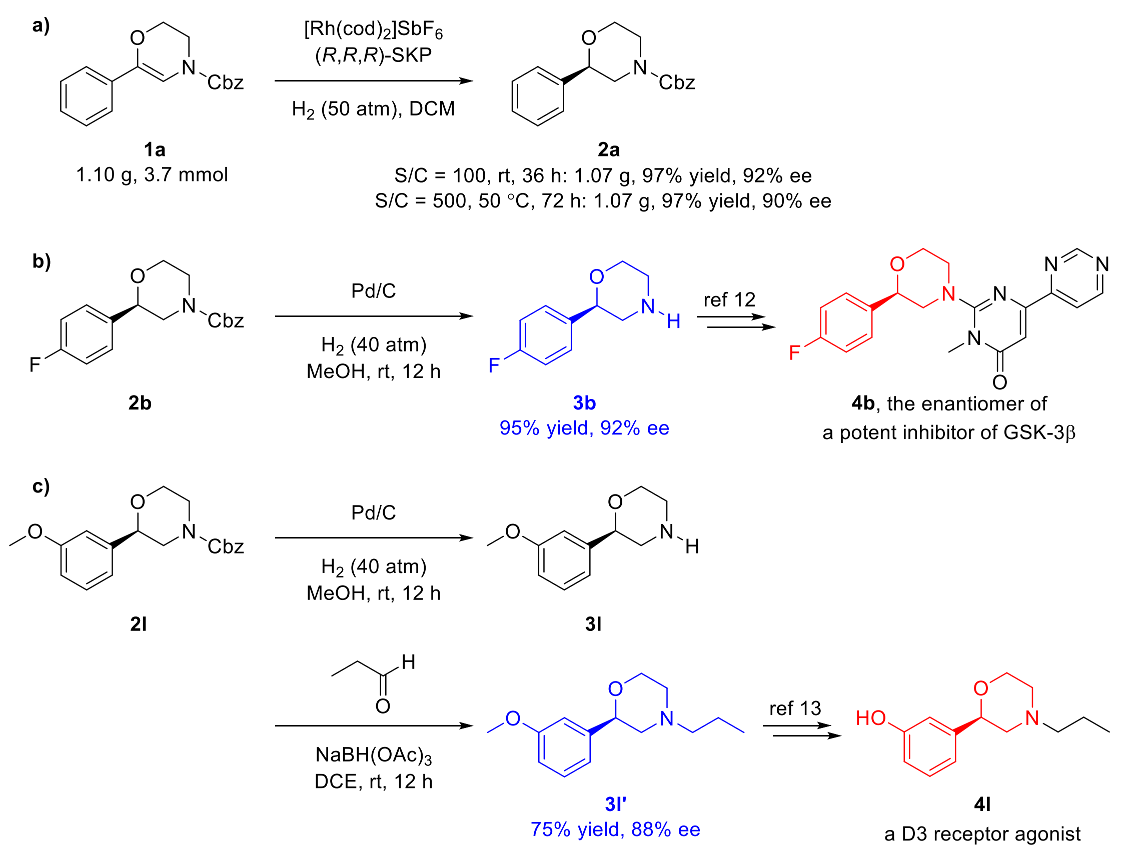

a) Gram-scale hydrogenation:

Substrate **1a** (3.7 mmol, 1.10 g) and Rh[(cod)$_2$]SbF$_6$ (1 mol %), (*R*,*R*,*R*)-SKP (1.05 mol %) were charged in an autoclave. The system was evacuated and filled with hydrogen. After repeating this operation for three times, degassed DCM (30 mL) was added and the hydrogen pressure was adjusted to 50 atm. After vigorous stirring at room temperature for 36 h, the reaction mixture was evaporated under reduced pressure. The desired product **2a** was obtained in 97% yield after flash chromatography (eluent PE/EA = 10/1). The ee value was 92% as determined by chiral HPLC.

When the catalyst loading was reduced to 0.2 mol %, **2a** was obtained in 97% yield and 88% ee after vigorous stirring at room temperature for 72 h at 50 ºC.

b) Synthesis of 3b:

Compound **2b** (63.1 mg, 0.2 mmol) was dissolved in methanol (2 mL) and 10% Pd/C (wetted with ca. 55% Water) (21.3 mg, 0.02 mmol) was added. The reaction system was put into an autoclave, evacuated and filled with hydrogen. After repeating this operation for three times, the hydrogen pressure was adjusted to 40 atm. The reaction mixture was filtered under reduced pressure after vigorous stirring at room temperature for 12 h. The desired product **3b**

was obtained in 95% yield (34.4 mg). The ee value was 92% as determined by chiral HPLC after acylation by CbzCl.

(*R*)-2-(4-fluorophenyl)morpholine (3b)

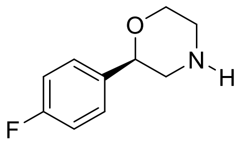

Colorless oil (34.4 mg, 95% yield). ^{1}H NMR (400 MHz, $CDCl_3$): *δ* 7.32 (dd, *J* = 8.4, 5.6 Hz, 2H), 7.02 (t, *J* = 8.8 Hz, 2H), 4.46 (d, *J* = 10.0 Hz, 1H), 4.02 (dd, *J* = 10.8, 2.4 Hz, 1H), 3.77 (t, *J* = 11.6 Hz, 1H), 3.04-2.87 (m, 3H), 2.76 (t, *J* = 10.8 Hz, 1H), 1.88 (brs, 1H). ^{13}C NMR (100 MHz, $CDCl_3$): *δ* 163.61 (161.17, *J* = 246.4 Hz), 136.39 (136.36, *J* = 3.0 Hz), 127.87 (127.79, *J* = 8.1 Hz), 115.40 (115.18, *J* = 22.2 Hz), 78.68, 68.43, 53.29, 45.68. ^{19}F NMR (376 MHz, $CDCl_3$): *δ* -114.75. ESI-HRMS: m/z for $C_{10}H_{13}FNO$ $[M+H]^+$ calcd 182.0976, found 182.0971.

$[\alpha]_D^{25}$ -14.7 (*c* 0.4, CH_2Cl_2). 92% ee. Determined by HPLC analysis using a Daicel Chiralcel IE column (25 cm × 0.46 cm) after acylation by CbzCl, hexane/isopropanol = 95/5, 0.8 mL/min, 210 nm, t_R (minor) = 13.0 min, t_R (major) = 14.7 min.

c) Synthesis of 3l':

Compound **2l** (390.4 mg, 1.2 mmol) was dissolved in methanol (20 mL) and 10% Pd/C (wetted with ca. 55% Water) (127.8 mg, 0.12 mmol) was added. The reaction system was put into an autoclave, evacuated and filled with hydrogen. After repeating this operation for three times, the hydrogen pressure was adjusted to 40 atm. The reaction mixture was filtered under reduced pressure after vigorous stirring at room temperature for 12 h to obtain the desired product **3l**, which was used for the next step without further purification.

An oven-dried 50 mL two-necked bottle was capped with a rubber stopper and then evacuated and backfilled with nitrogen (this sequence was carried out twice). Compound **3l** in anhydrous DCE (15 mL) was added into the reaction flask via syringe under N_2 atmosphere, followed by addition of $NaBH(OAc)_3$ (508.7 mg, 2.4 mmol, 2.0 equiv) and acetaldehyde (0.23 mL, 3.0 mmol, 2.5 equiv). The mixture was stirred at room temperature for 12 h and was quenched with an aqueous solution of saturated sodium carbonate (10 mL). The solution was extracted with diethyl ether (20 mL × 2). The combined organic layer was dried over anhydrous Na_2SO_4, filtered and concentrated under reduced pressure to give the crude product. The residue was purified by column chromatography (PE/EA = 5/1 with 1% NH_4OH) to give product **3l'** in 75% yield over two steps.

(*R*)-2-(3-methoxyphenyl)-4-propylmorpholine (3l')

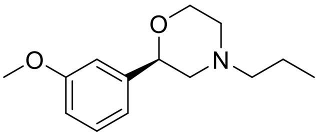

Colorless oil (211.8 mg, 75% yield). ^{1}H NMR (400 MHz, $CDCl_3$): *δ* 7.24 (t, *J* = 7.6 Hz, 1H), 6.94 (s, 2H), 6.82 (d, *J* = 7.2 Hz, 1H), 4.55 (d, *J* = 10.0 Hz, 1H), 4.03 (d, *J* =

10.4 Hz, 1H), 3.86-3.80 (m, 4H), 2.94 (d, *J* = 10.8 Hz, 1H), 2.79 (d, *J* = 11.2 Hz, 1H), 2.33 (t, *J* = 7.2 Hz, 2H), 2.21 (t, *J* = 10.4 Hz, 1H), 2.04 (t, *J* = 10.4 Hz, 1H), 1.58-1.48 (m, 2H), 0.91 (t, *J* = 8.0 Hz, 3H). ^{13}C NMR (100 MHz, $CDCl_3$): *δ* 159.70, 142.24, 129.37, 118.55, 113.42, 111.64, 78.17, 67.16, 60.92, 60.71, 55.35 (55.26, m), 53.04, 19.80, 11.98. ESI-HRMS: m/z for $C_{14}H_{22}NO_2$ $[M+H]^+$ calcd 236.1645, found 236.1647.

$[\alpha]_D^{25}$ +42.6 (*c* 0.2, CH_2Cl_2). 88% ee. Determined by HPLC analysis using a Daicel Chiralcel OX-H column (25 cm × 0.46 cm), hexane/isopropanol = 90/10, 1.0 mL/min, 210 nm, t_R (major) = 9.7 min, t_R (minor) = 10.9 min.

6. X-Ray Analysis Data

X-Ray Crystallographic Data for **1c**:

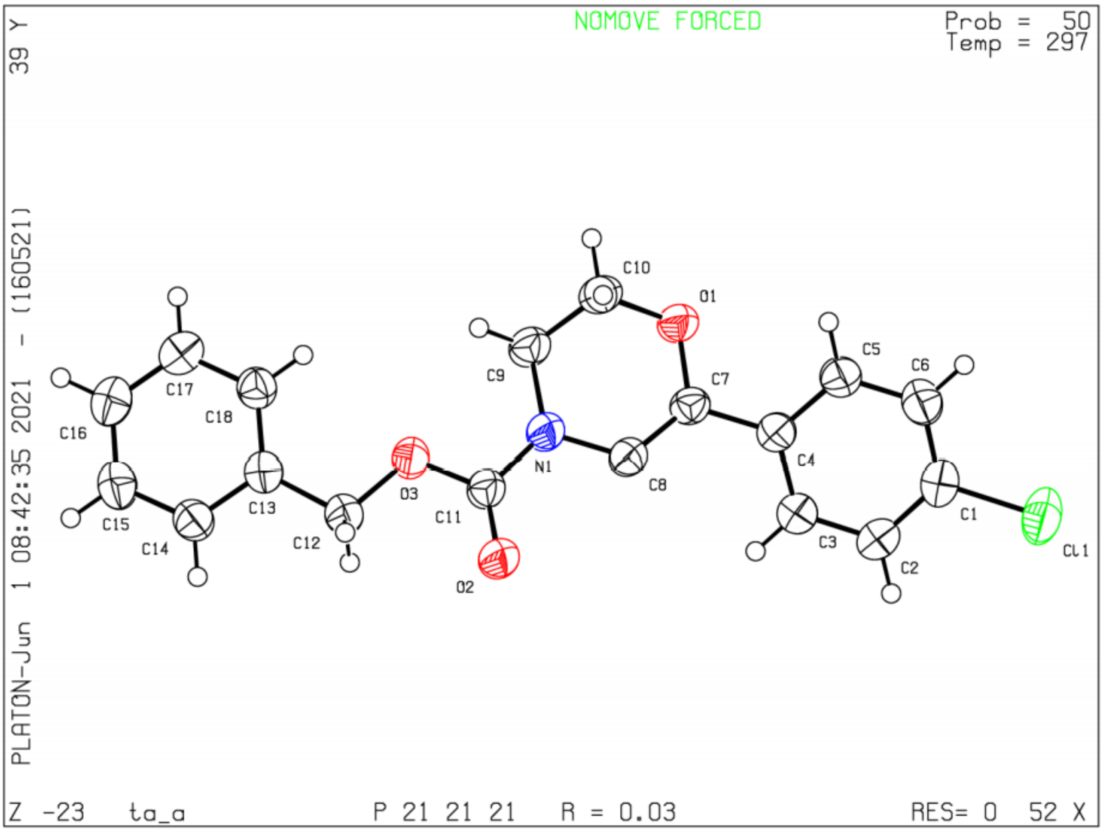

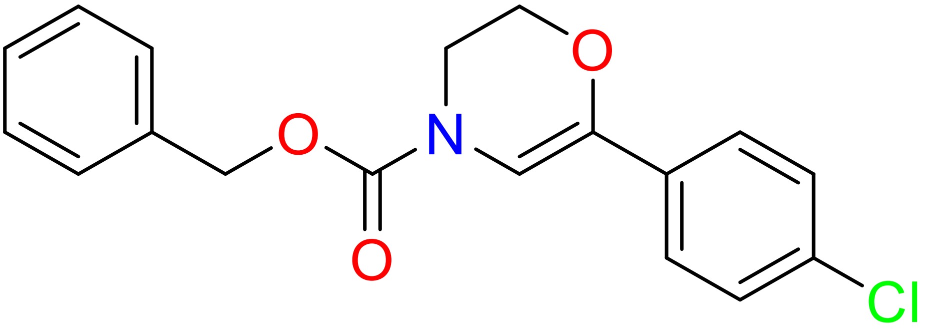

1c

CCDC 2095806

```
Bond precision:   C-C = 0.0041 A          Wavelength=1.54178

Cell:              a=5.5094(2)      b=14.3271(4)      c=19.9017(6)
                   alpha=90         beta=90           gamma=90
Temperature:       297 K

                  Calculated                  Reported
Volume            1570.92(9)                  1570.92(9)
Space group       P 21 21 21                  P 21 21 21
Hall group        P 2ac 2ab                   P 2ac 2ab
Moiety formula    C18 H15 Cl N O3             C18 H15 Cl N O3
Sum formula       C18 H15 Cl N O3             C18 H15 Cl N O3
Mr                328.76                      328.76
Dx,g cm-3         1.390                       1.390
Z                 4                           4
Mu (mm-1)         2.280                       2.280
F000              684.0                       684.0
F000'             687.40
h,k,lmax          6,17,23                     6,17,23
Nref              2883[ 1699]                 2883
Tmin,Tmax         0.636,0.663                 0.641,0.753
Tmin'             0.577

Correction method= # Reported T Limits: Tmin=0.641 Tmax=0.753
AbsCorr = MULTI-SCAN

Data completeness= 1.70/1.00       Theta(max)= 68.165

R(reflections)= 0.0345( 2642)      wR2(reflections)= 0.0996( 2883)

S = 1.040                  Npar= 208
```

Alert level C

```
PLAT340_ALERT_3_C Low Bond Precision on  C-C Bonds ...............    0.00406 Ang.
PLAT975_ALERT_2_C Check Calcd Resid. Dens.  1.05A   From C8             0.41 eA-3
```

Alert level G

```
PLAT343_ALERT_2_G Unusual sp?      Angle Range in Main Residue for         C8 Check
PLAT720_ALERT_4_G Number of Unusual/Non-Standard Labels ..........          1 Note
PLAT883_ALERT_1_G No Info/Value for _atom_sites_solution_primary .    Please Do !
PLAT909_ALERT_3_G Percentage of I>2sig(I) Data at Theta(Max) Still        82% Note
PLAT912_ALERT_4_G Missing # of FCF Reflections Above STh/L=  0.600          1 Note
PLAT965_ALERT_2_G The SHELXL WEIGHT Optimisation has not Converged    Please Check
PLAT978_ALERT_2_G Number C-C Bonds with Positive Residual Density.          0 Info
```

0 **ALERT level A** = Most likely a serious problem - resolve or explain
0 **ALERT level B** = A potentially serious problem, consider carefully
2 **ALERT level C** = Check. Ensure it is not caused by an omission or oversight
7 **ALERT level G** = General information/check it is not something unexpected

1 ALERT type 1 CIF construction/syntax error, inconsistent or missing data
4 ALERT type 2 Indicator that the structure model may be wrong or deficient
2 ALERT type 3 Indicator that the structure quality may be low
2 ALERT type 4 Improvement, methodology, query or suggestion
0 ALERT type 5 Informative message, check

X-Ray Crystallographic Data for **2b**:

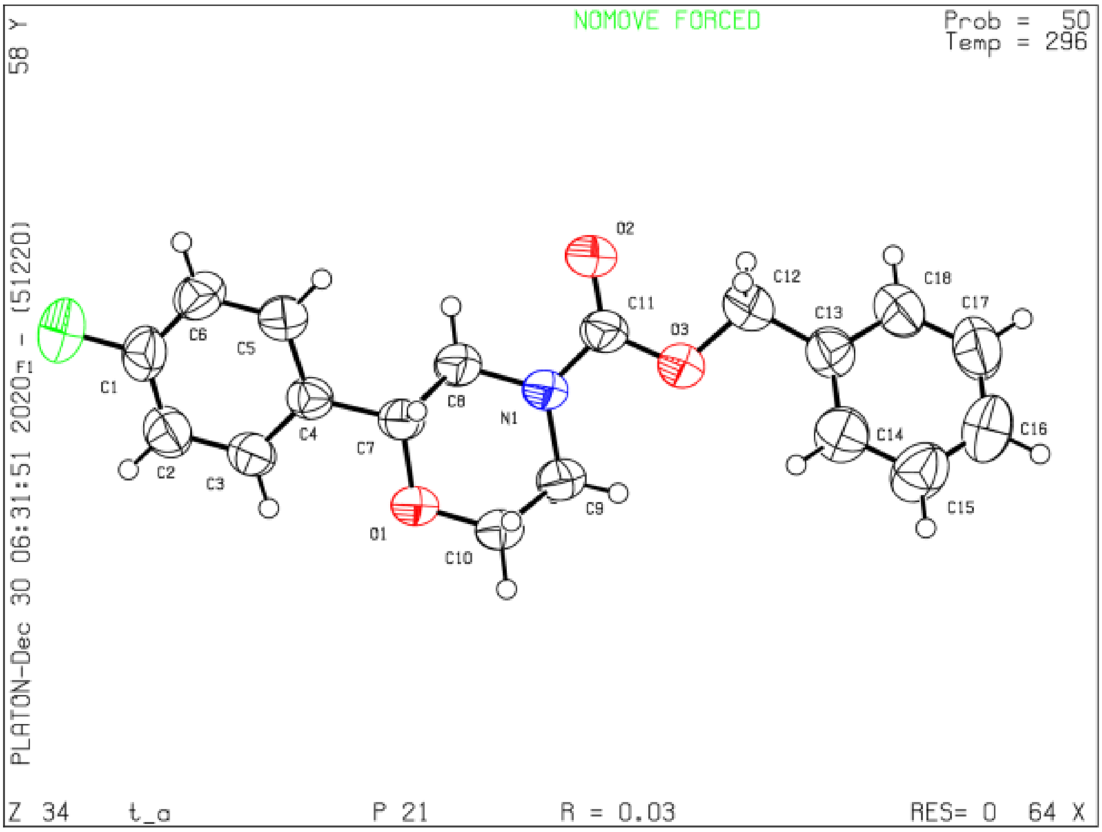

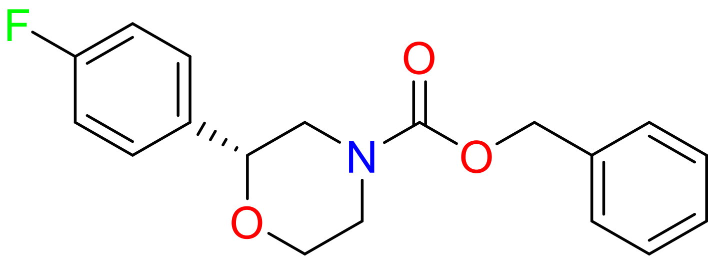

(*R*)-2b

CCDC 2095617

```
Bond precision:   C-C = 0.0039 A          Wavelength=1.54178

Cell:              a=8.798(3)      b=8.047(2)            c=11.933(7)
                   alpha=90        beta=108.18(2)        gamma=90
Temperature:       296 K

                  Calculated                   Reported
Volume            802.7(6)                     802.7(5)
Space group       P 21                         P 21
Hall group        P 2yb                        P 2yb
Moiety formula    C18 H18 F N O3               C18 H18 F N O3
Sum formula       C18 H18 F N O3               C18 H18 F N O3
Mr                315.33                       315.33
Dx,g cm-3         1.305                        1.305
Z                 2                            2
Mu (mm-1)         0.801                        0.801
F000              332.0                        332.0
F000'             333.11
h,k,lmax          10,9,14                      10,9,14
Nref              2952[ 1590]                  2929
Tmin,Tmax         0.866,0.894                  0.672,0.753
Tmin'             0.866

Correction method= # Reported T Limits: Tmin=0.672 Tmax=0.753
AbsCorr = ?

Data completeness= 1.84/0.99       Theta(max)= 68.421

R(reflections)= 0.0317( 2709)      wR2(reflections)= 0.0888( 2929)

S = 1.028                  Npar= 208
```

Alert level C

```
PLAT052_ALERT_1_C Info on Absorption Correction Method   Not Given     Please Do !
PLAT242_ALERT_2_C Low    'MainMol' Ueq as Compared to Neighbors of        C13 Check
PLAT334_ALERT_2_C Small Aver. Benzene C-C Dist C1        -C6             1.37 Ang.
PLAT911_ALERT_3_C Missing FCF Refl Between Thmin & STh/L=     0.600        10 Report
PLAT913_ALERT_3_C Missing # of Very Strong Reflections in FCF ....          9 Note
```

Alert level G

```
PLAT720_ALERT_4_G Number of Unusual/Non-Standard Labels ..........          2 Note
PLAT791_ALERT_4_G Model has Chirality at C7           (Sohnke SpGr)         R Verify
PLAT850_ALERT_4_G Check Flack Parameter Exact Value 0.00 with s.u.      0.05 Check
PLAT883_ALERT_1_G No Info/Value for _atom_sites_solution_primary .   Please Do !
PLAT912_ALERT_4_G Missing # of FCF Reflections Above STh/L=  0.600          4 Note
PLAT965_ALERT_2_G The SHELXL WEIGHT Optimisation has not Converged   Please Check
PLAT978_ALERT_2_G Number C-C Bonds with Positive Residual Density.          0 Info
PLAT992_ALERT_5_G Repd & Actual _reflns_number_gt Values Differ by          2 Check
```

```
   0 ALERT level A = Most likely a serious problem - resolve or explain
   0 ALERT level B = A potentially serious problem, consider carefully
   5 ALERT level C = Check. Ensure it is not caused by an omission or oversight
   8 ALERT level G = General information/check it is not something unexpected

   2 ALERT type 1 CIF construction/syntax error, inconsistent or missing data
   4 ALERT type 2 Indicator that the structure model may be wrong or deficient
   2 ALERT type 3 Indicator that the structure quality may be low
   4 ALERT type 4 Improvement, methodology, query or suggestion
   1 ALERT type 5 Informative message, check
```

7. NMR Spectra

Benzyl 6-phenyl-2,3-dihydro-4*H*-1,4-oxazine-4-carboxylate (1a)

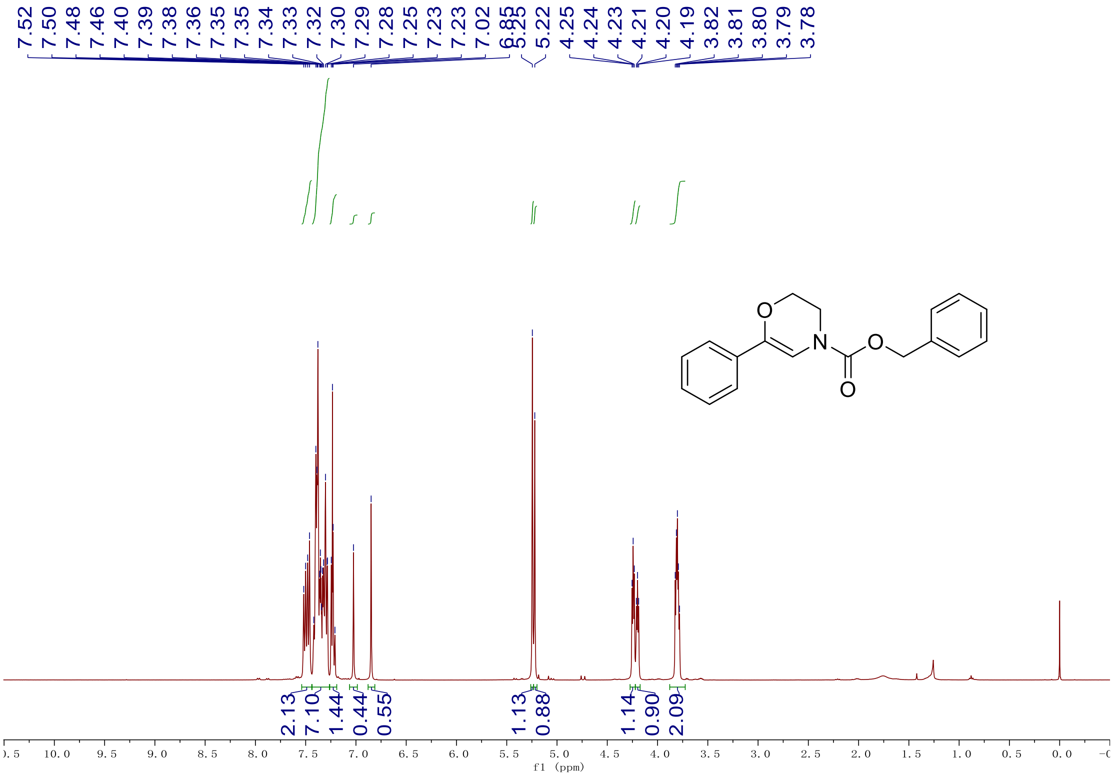

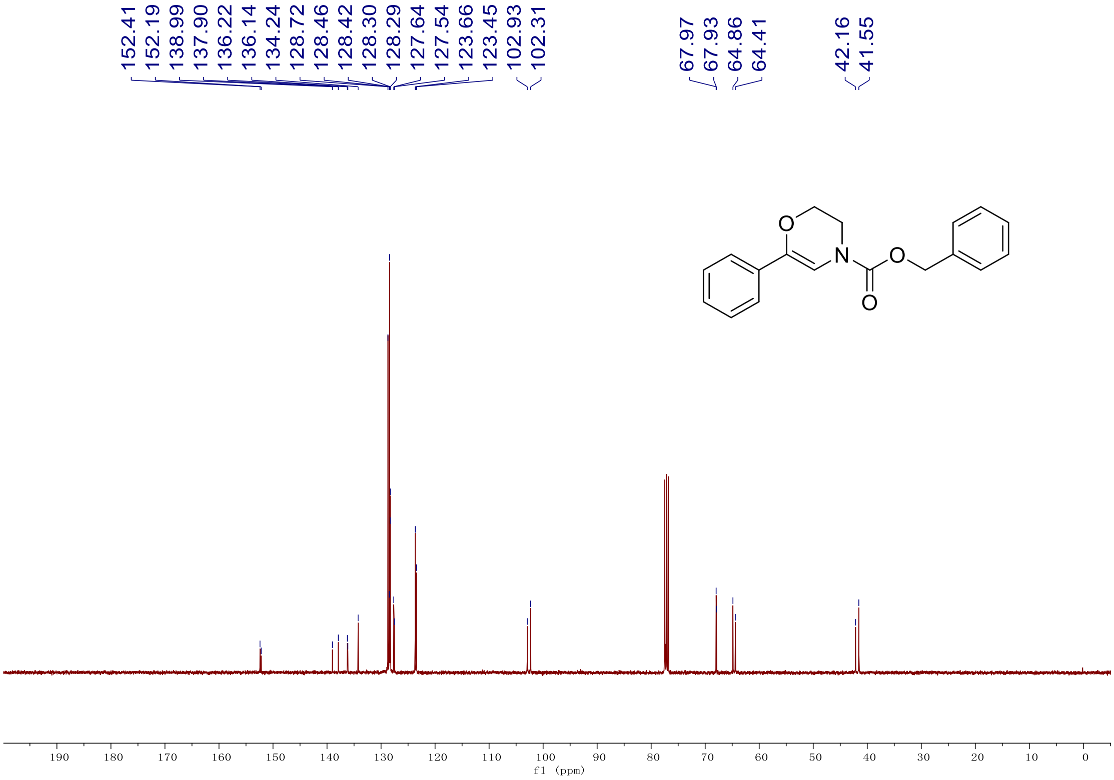

Benzyl 6-(4-fluorophenyl)-2,3-dihydro-4*H*-1,4-oxazine-4-carboxylate (1b)

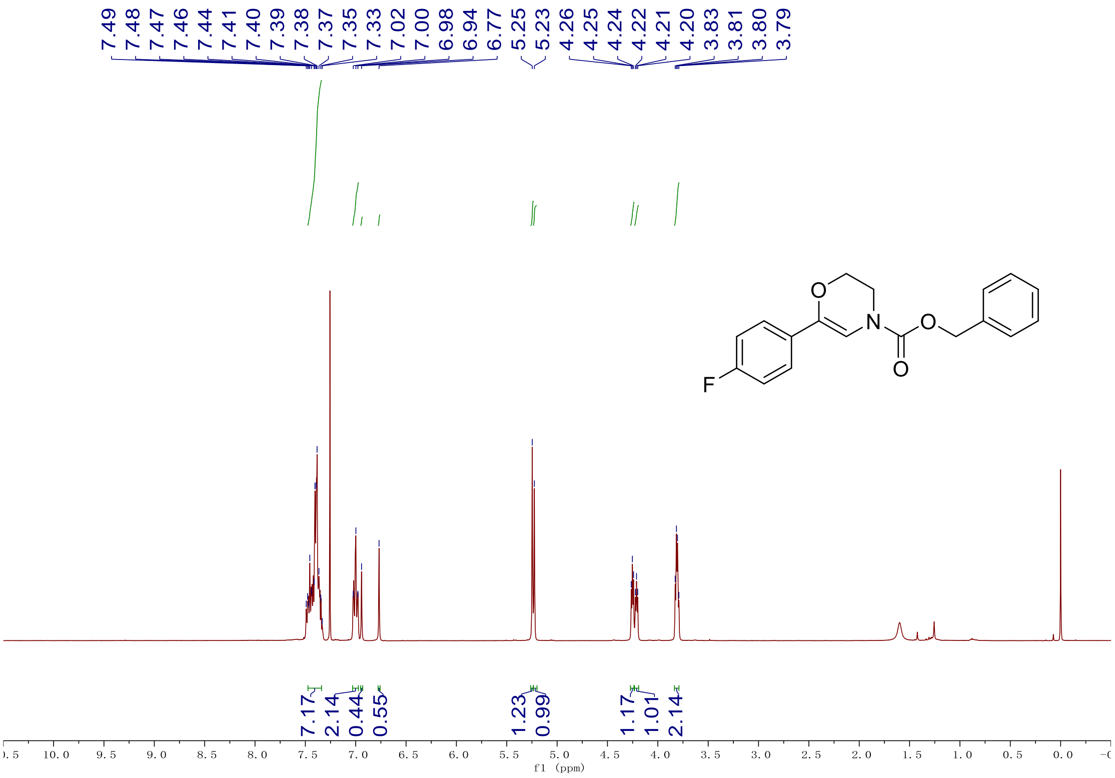

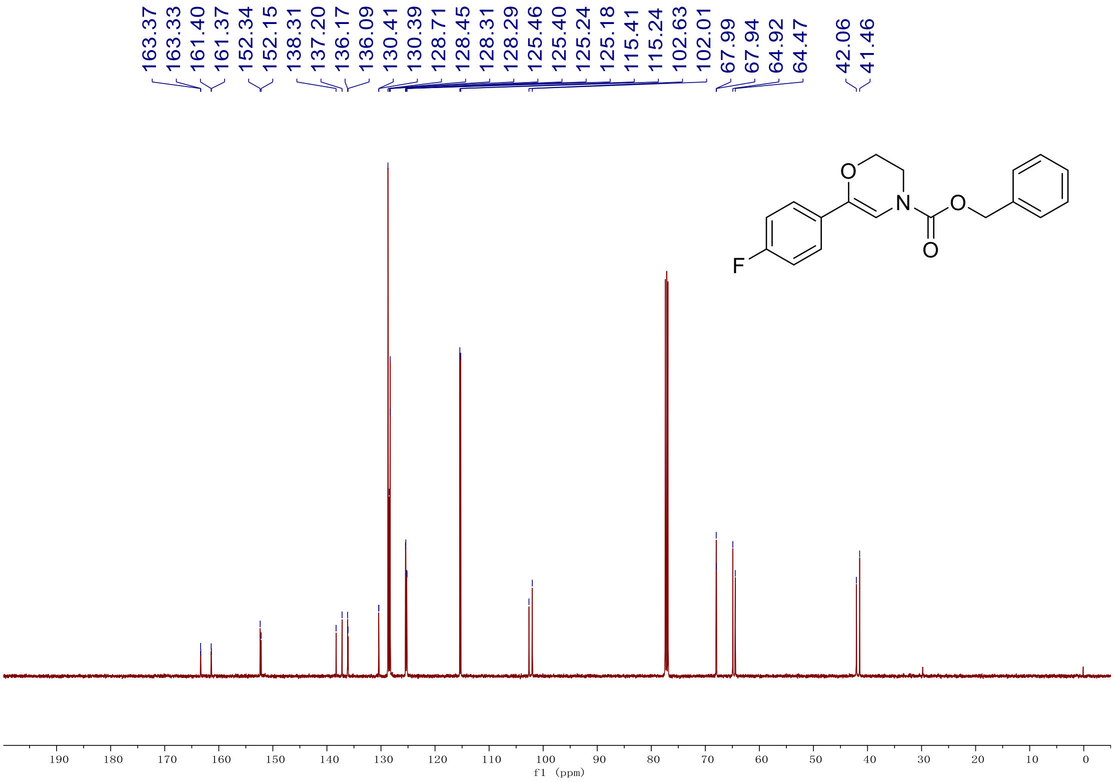

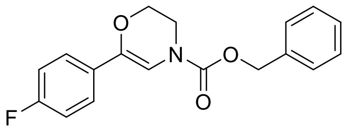
F
O
N
O
O

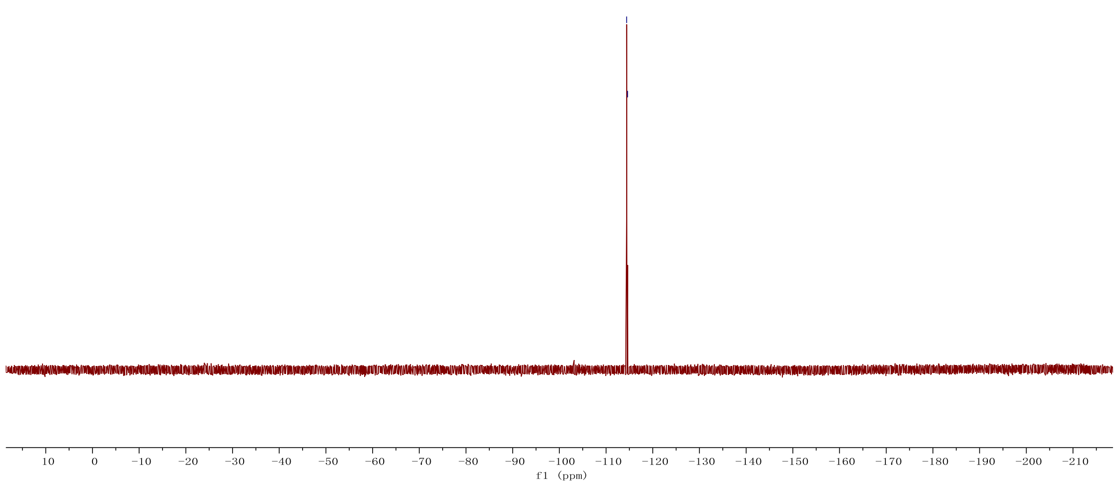
-114.40
-114.61
10
0
−10
−20
−30
−40
−50
−60
−70
−80
−90
−100
−110
−120
−130
−140
−150
−160
−170
−180
−190
−200
−210
f1 (ppm)

1-(6-(4-chlorophenyl)-2,3-dihydro-4*H*-1,4-oxazin-4-yl)-2-phenylethan-1-one (1c)

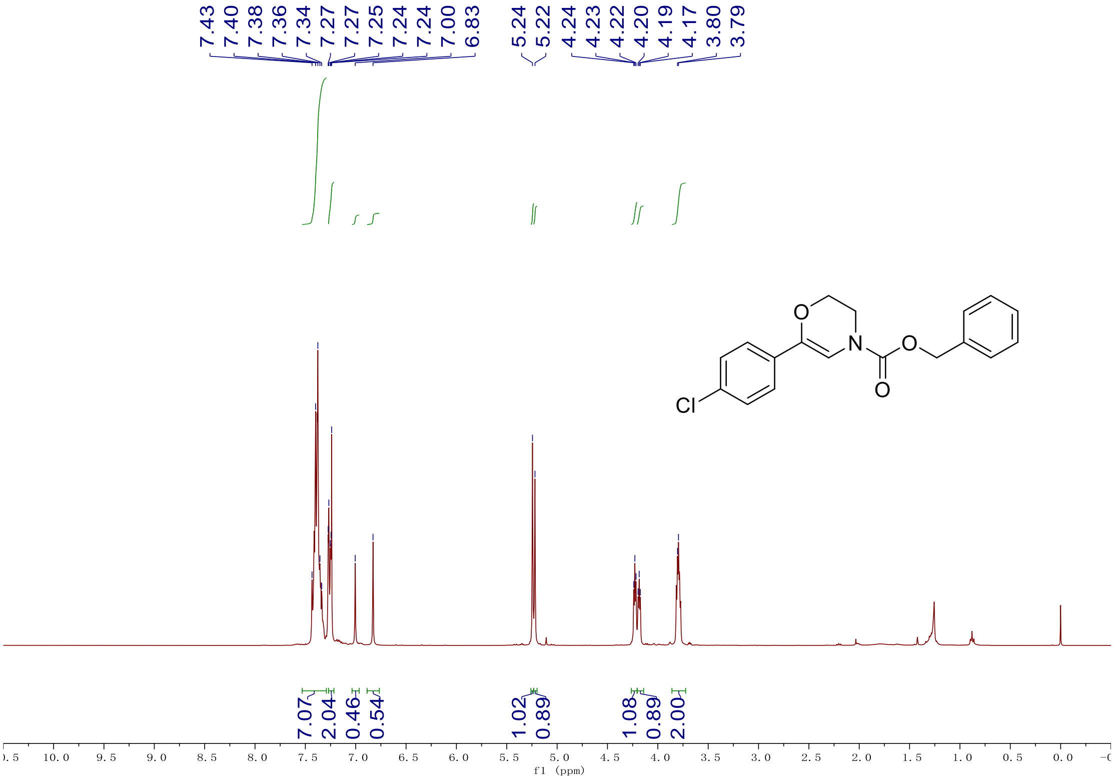

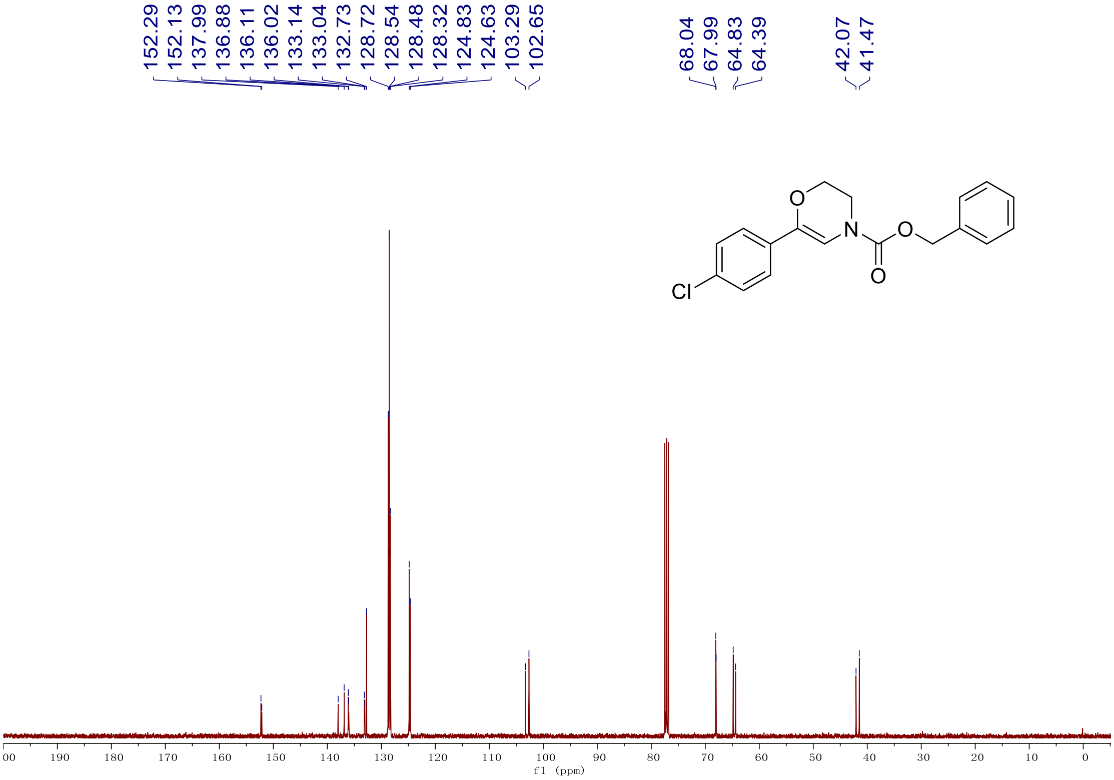

1-(6-(4-bromophenyl)-2,3-dihydro-4*H*-1,4-oxazin-4-yl)-2-phenylethan-1-one (1d)

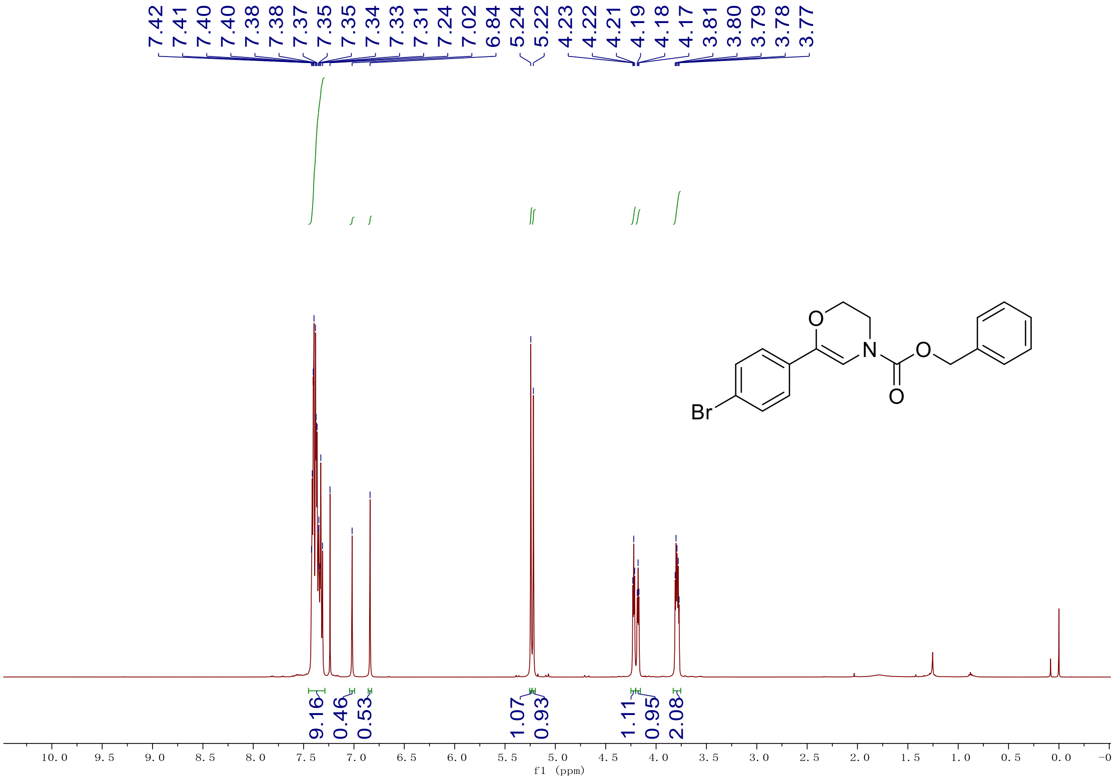

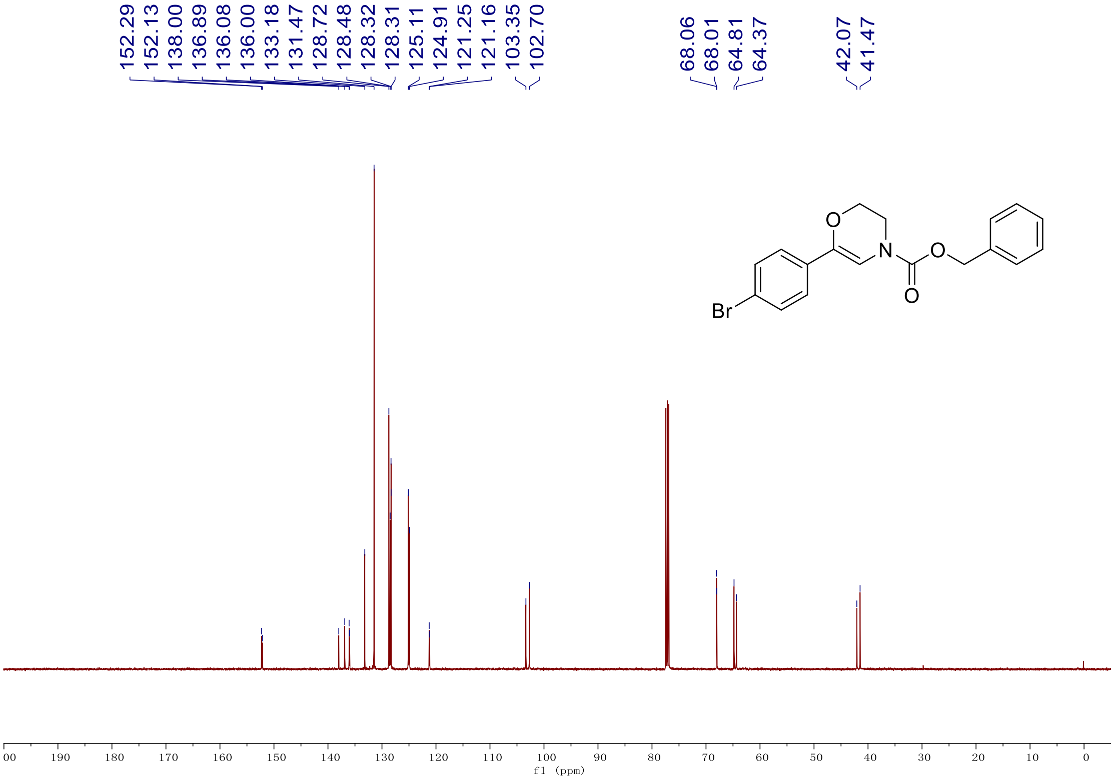

Benzyl 6-([1,1'-biphenyl]-4-yl)-2,3-dihydro-4*H*-1,4-oxazine-4-carboxylate (1e)

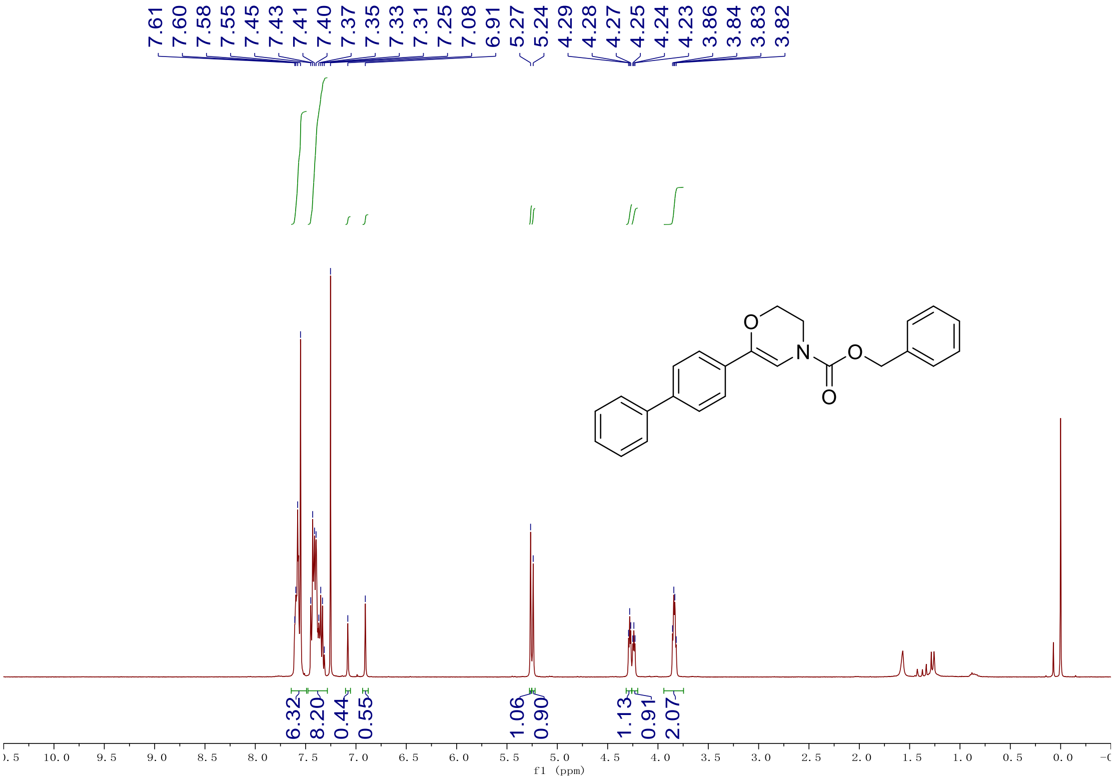

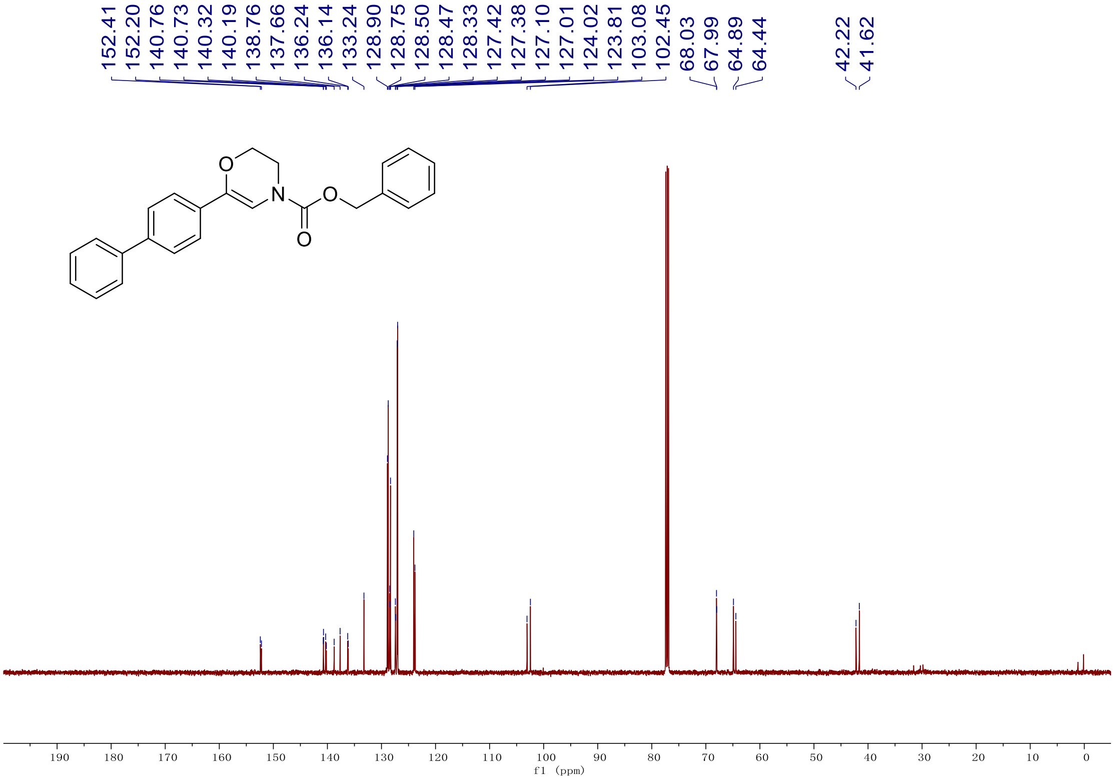

Benzyl 6-(4-(trifluoromethyl)phenyl)-2,3-dihydro-4*H*-1,4-oxazine-4-carboxylate (1f)

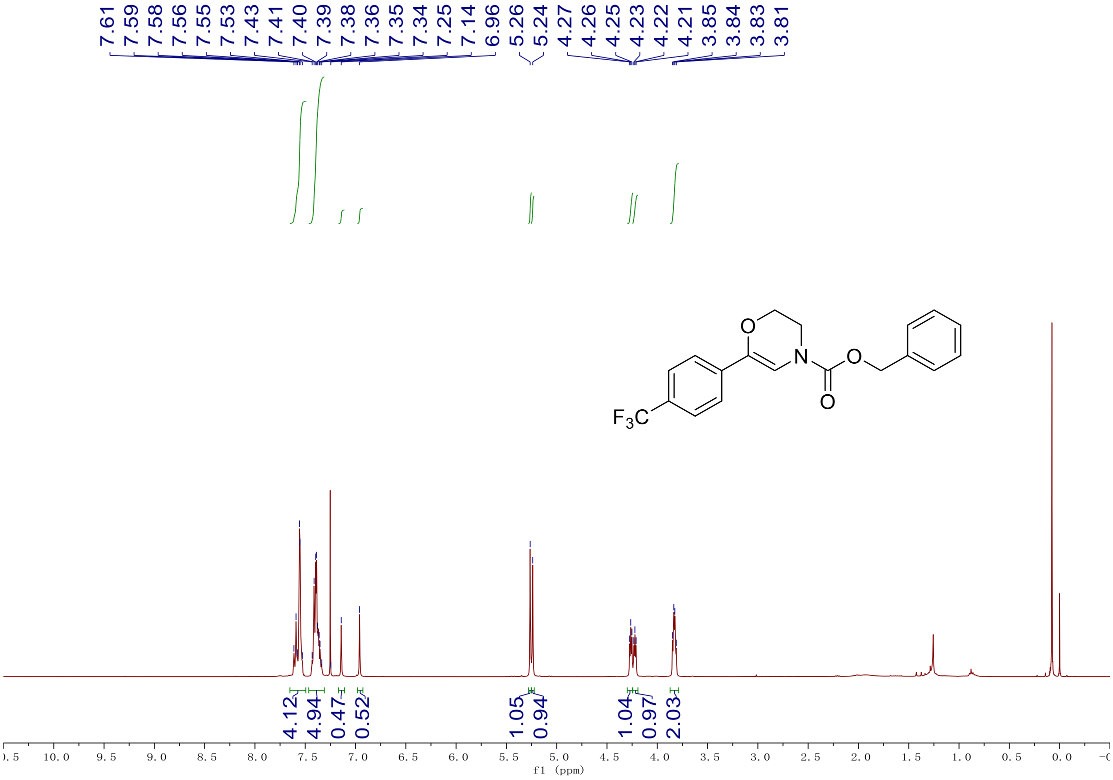

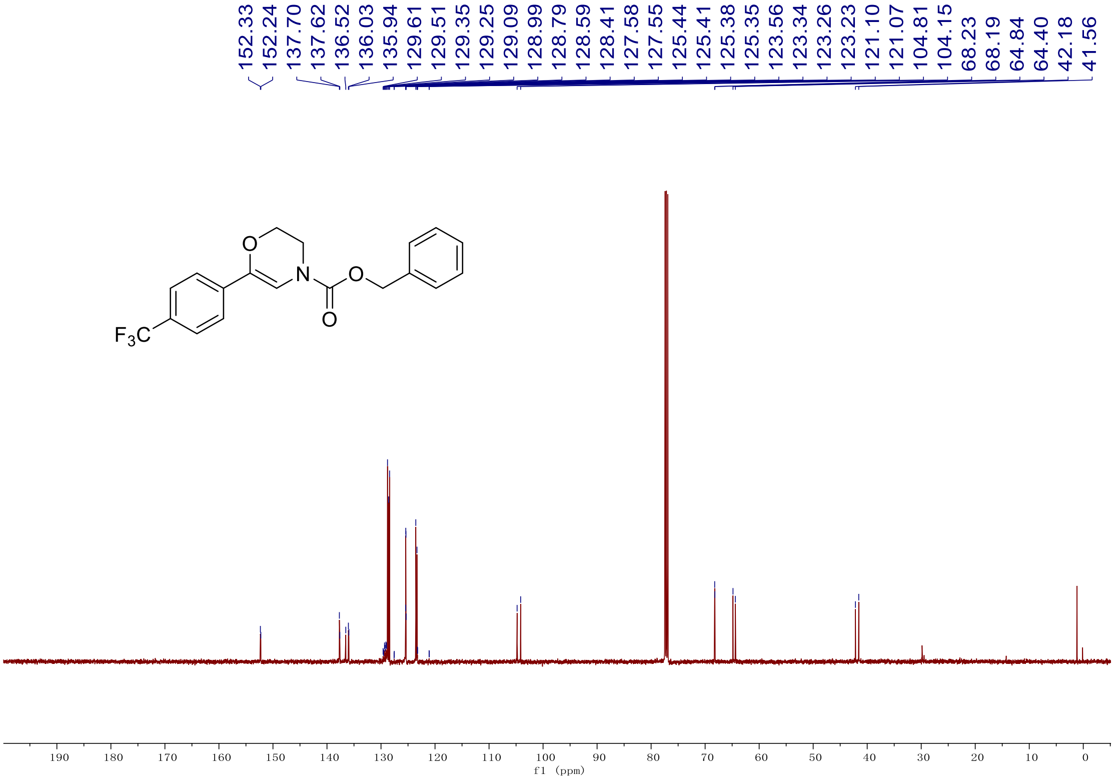

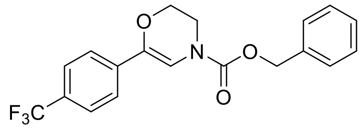

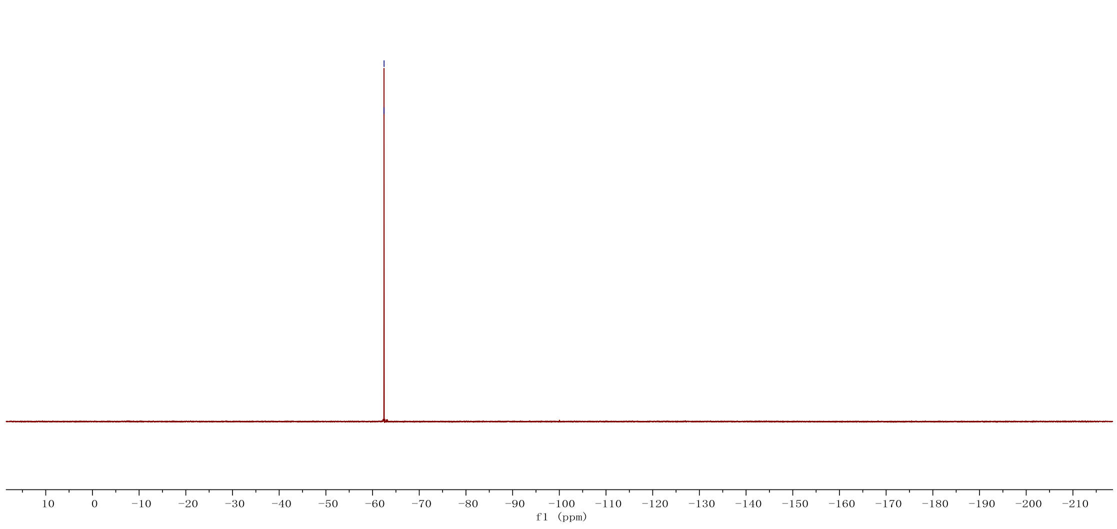
-62.44
-62.47
10
0
−10
−20
−30
−40
−50
−60
−70
−80
−90
−100
−110
−120
−130
−140
−150
−160
−170
−180
−190
−200
−210
f1 (ppm)

Benzyl 6-(p-tolyl)-2,3-dihydro-4*H*-1,4-oxazine-4-carboxylate (1g)

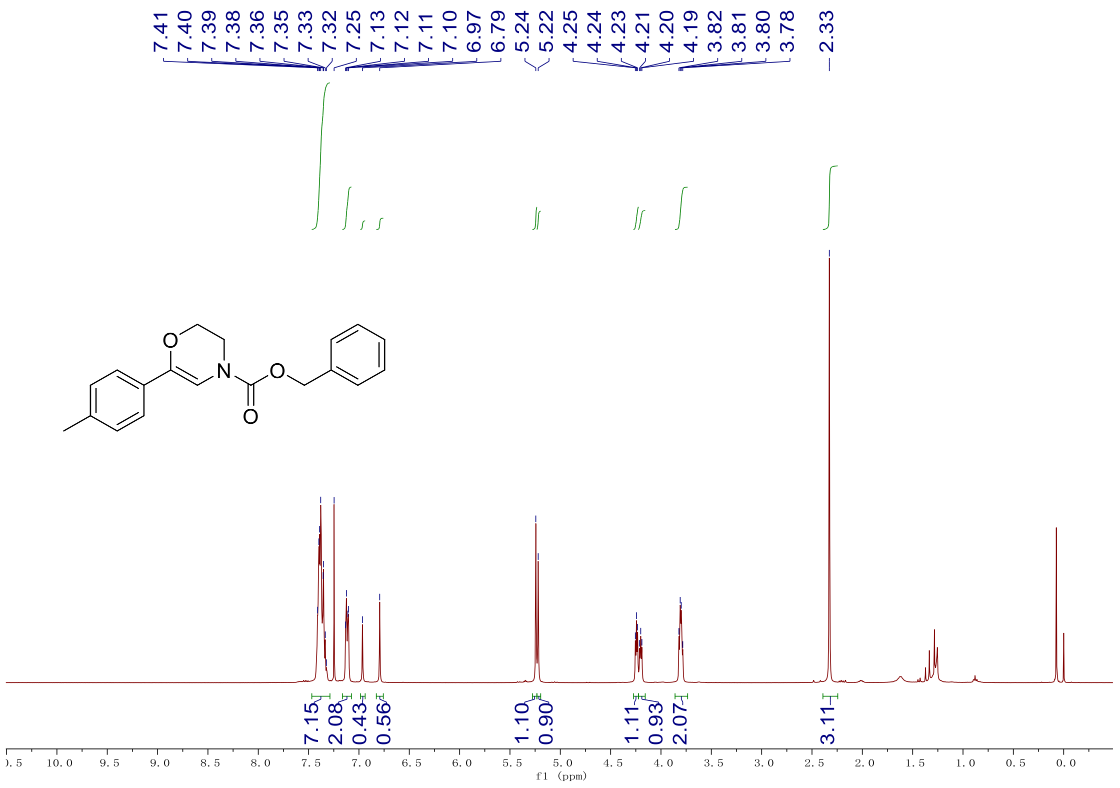

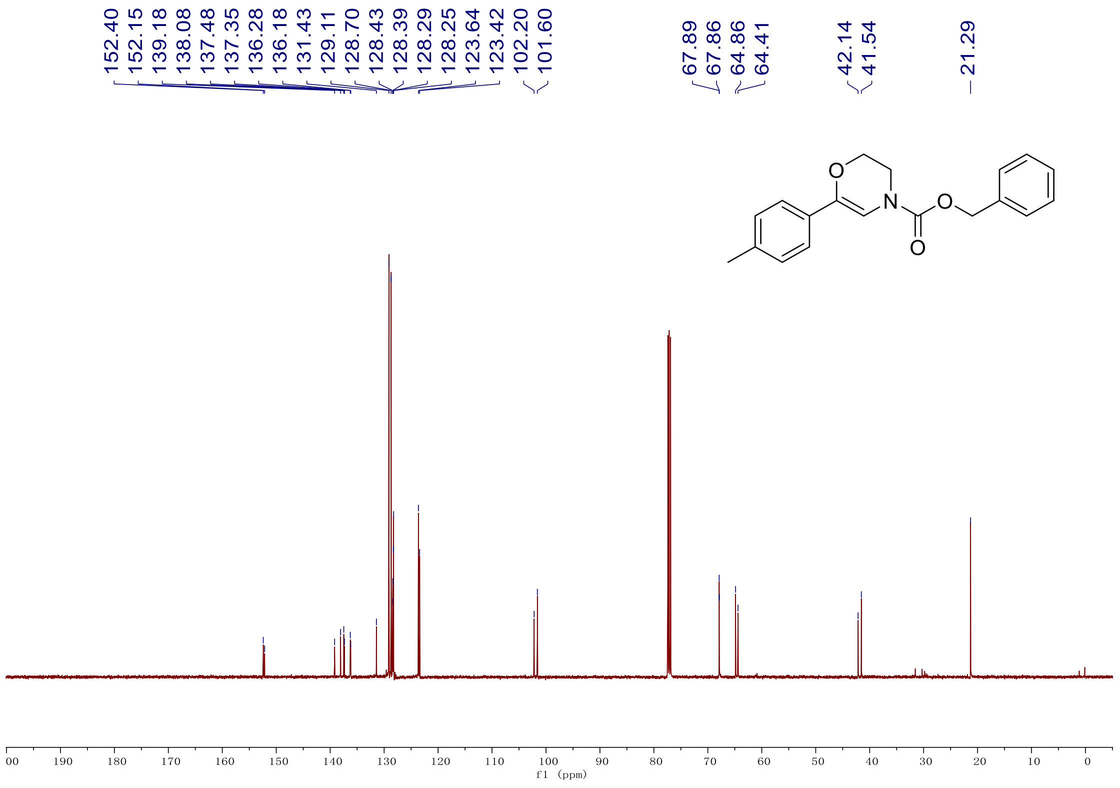

Benzyl 6-(4-methoxyphenyl)-2,3-dihydro-4*H*-1,4-oxazine-4-carboxylate (1h)

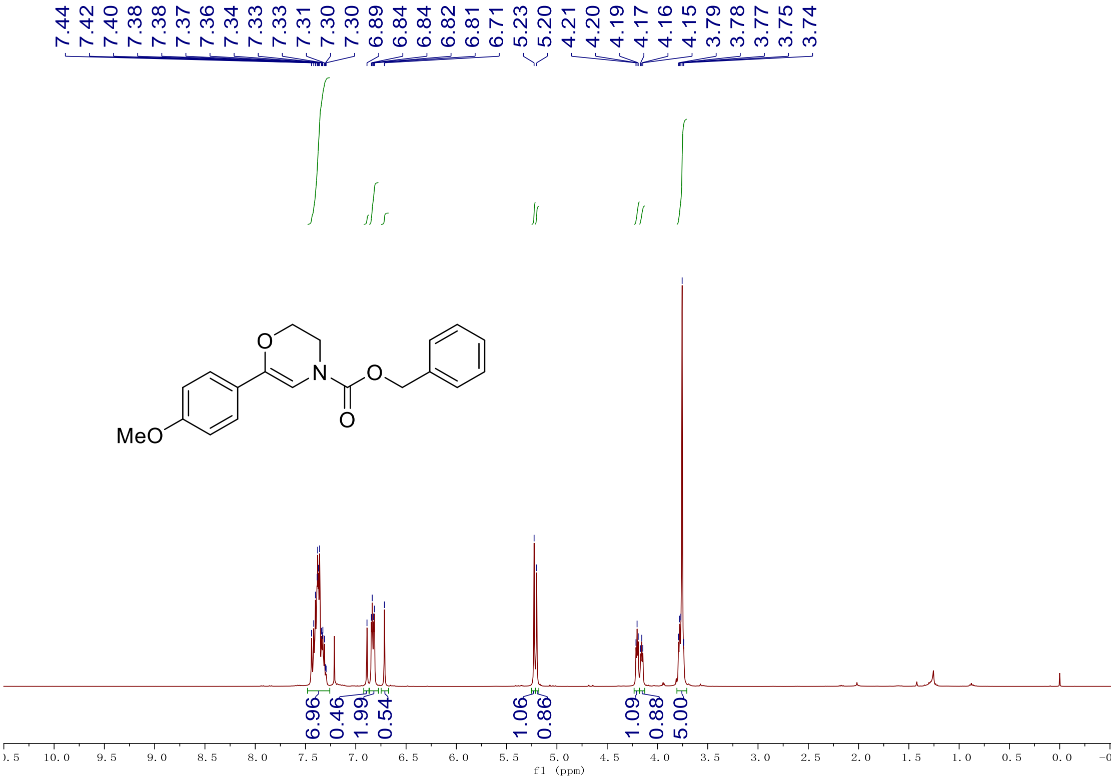

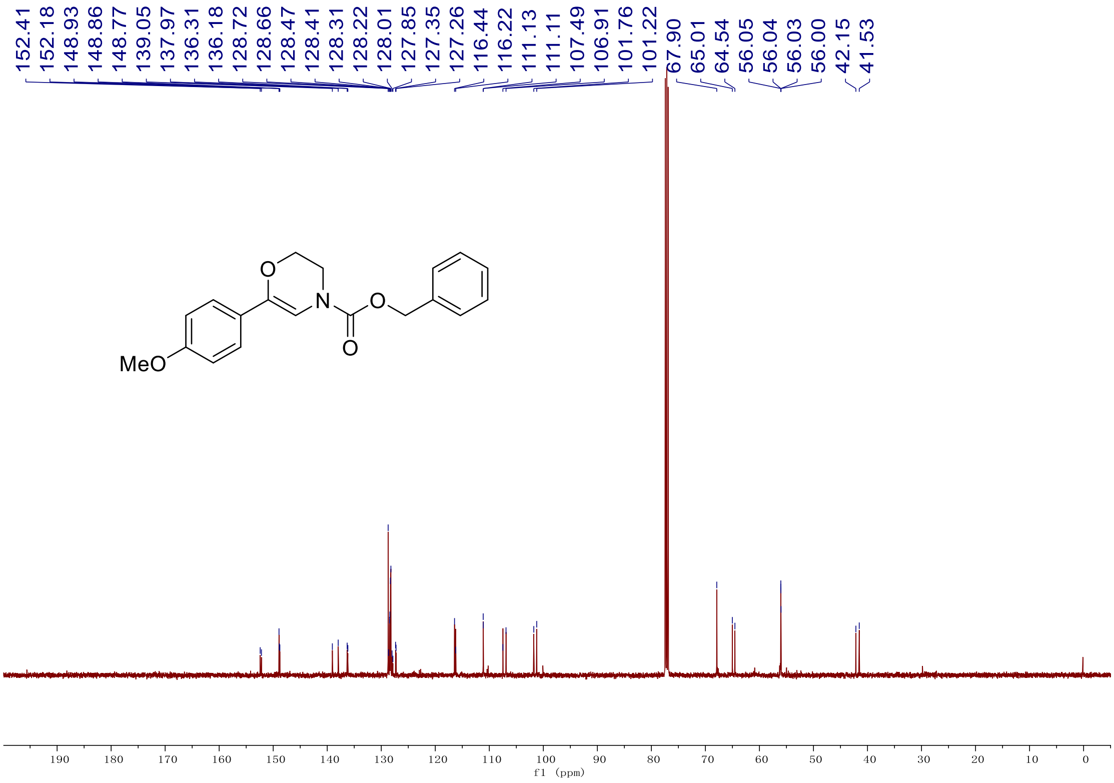

Benzyl 6-(3-fluorophenyl)-2,3-dihydro-4*H*-1,4-oxazine-4-carboxylate (1i)

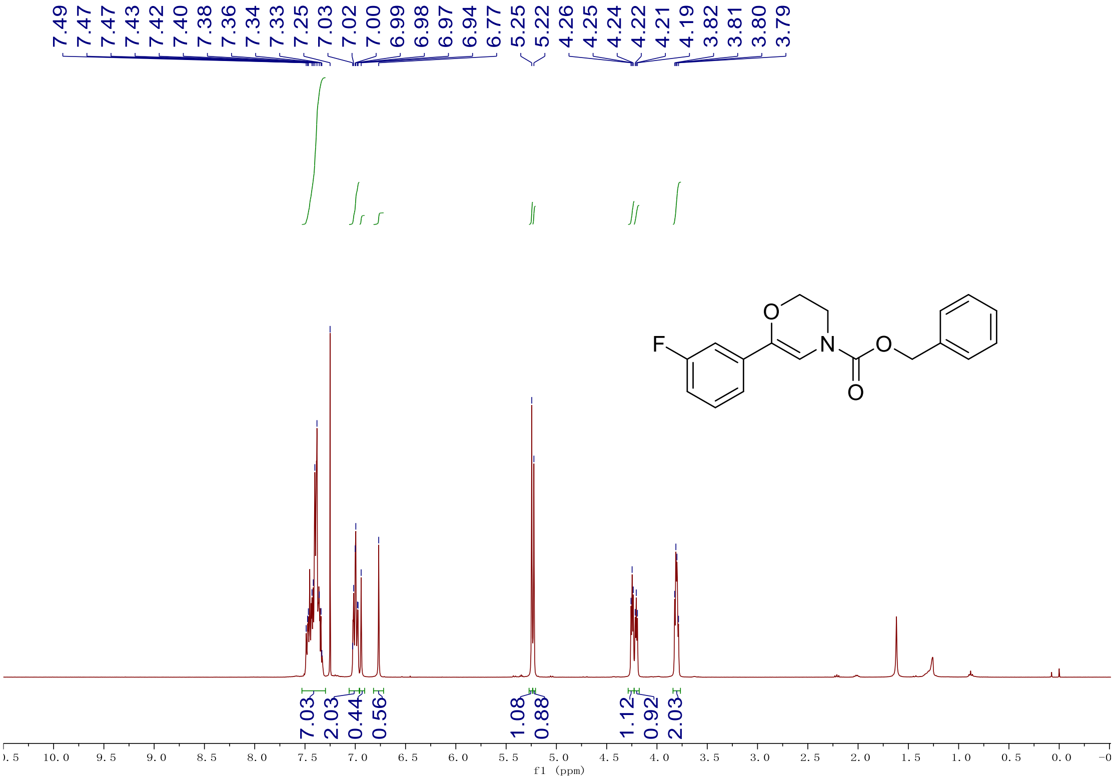

Diffusion ordered spectroscopy (DOSY) of 1i

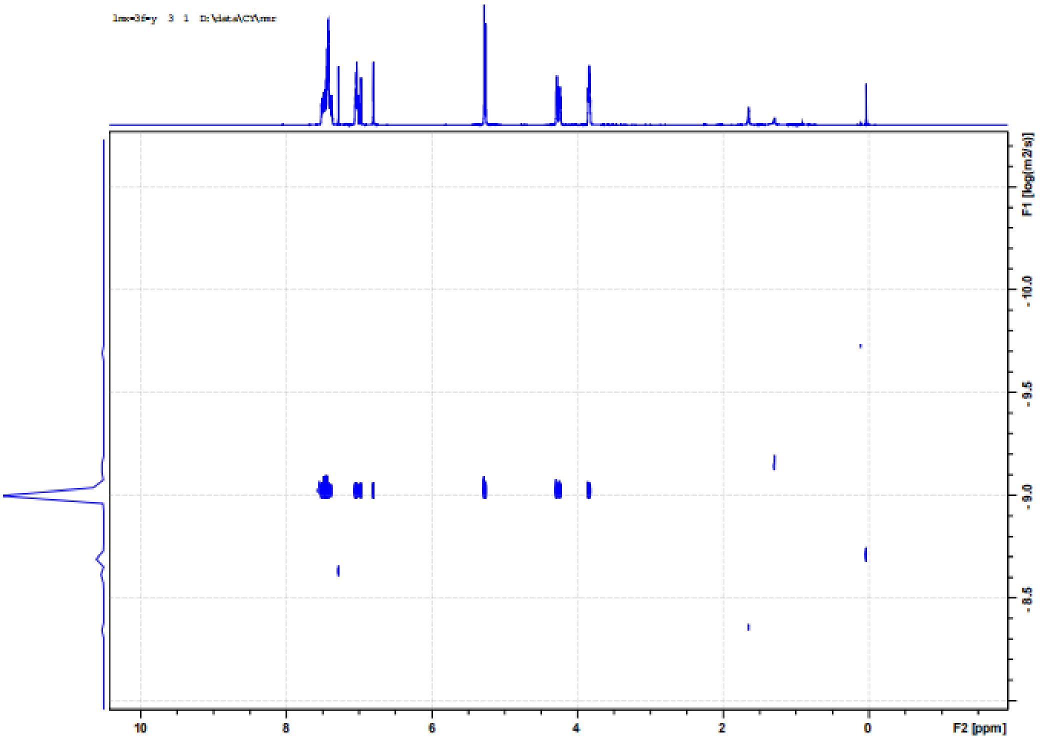

The unique diffusion coefficient indicates that the two sets of NMR data are caused by conformational isomerism.

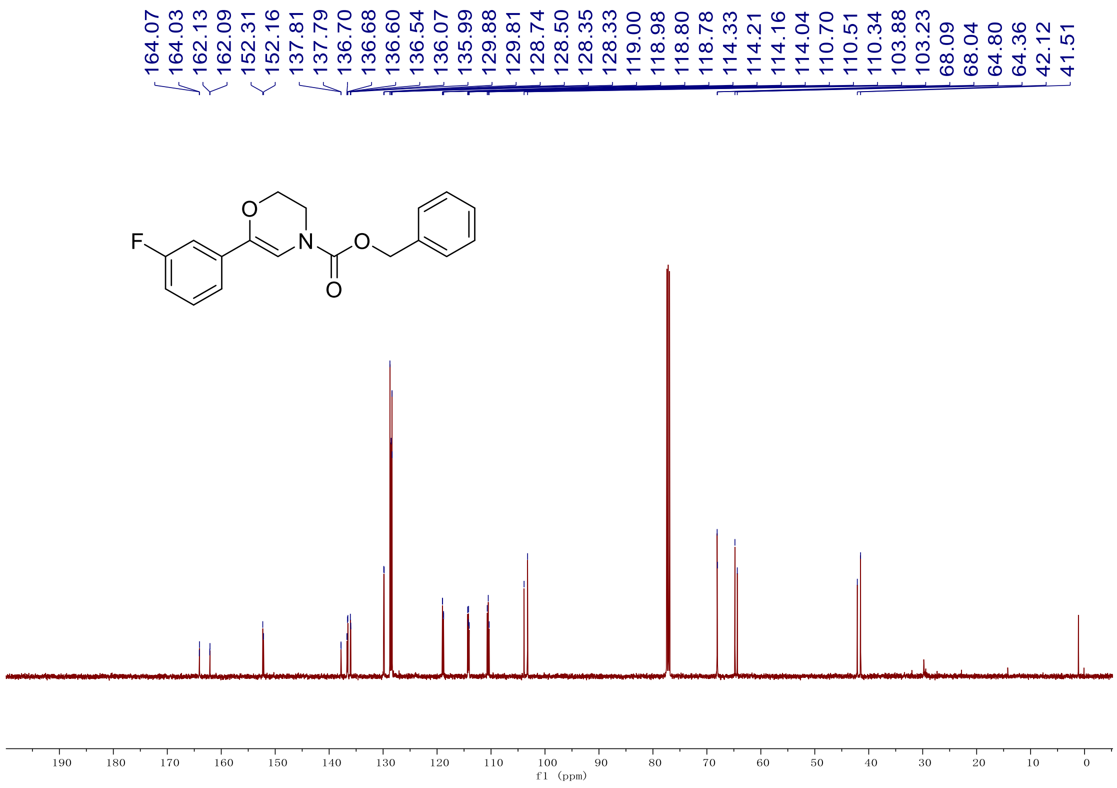
164.07
164.03
162.13
162.09
152.31
152.16
137.81
137.79
136.70
136.68
136.60
136.54
136.07
135.99
129.88
129.81
128.74
128.50
128.35
128.33
119.00
118.98
118.80
118.78
114.33
114.21
114.16
114.04
110.70
110.51
110.34
103.88
103.23
68.09
68.04
64.80
64.36
42.12
41.51
F
O
N
O
O
f1 (ppm)

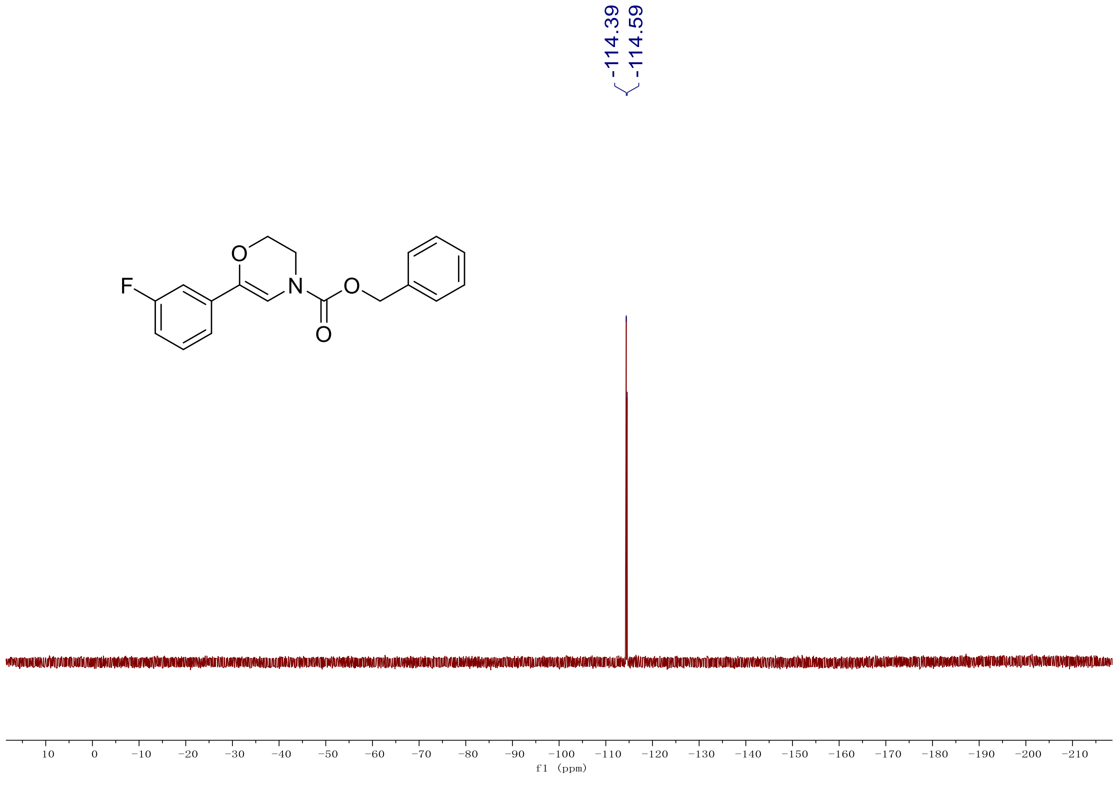
-114.39
-114.59
F
O
N
O
O
f1 (ppm)

Benzyl 6-(3-chlorophenyl)-2,3-dihydro-4*H*-1,4-oxazine-4-carboxylate (1j)

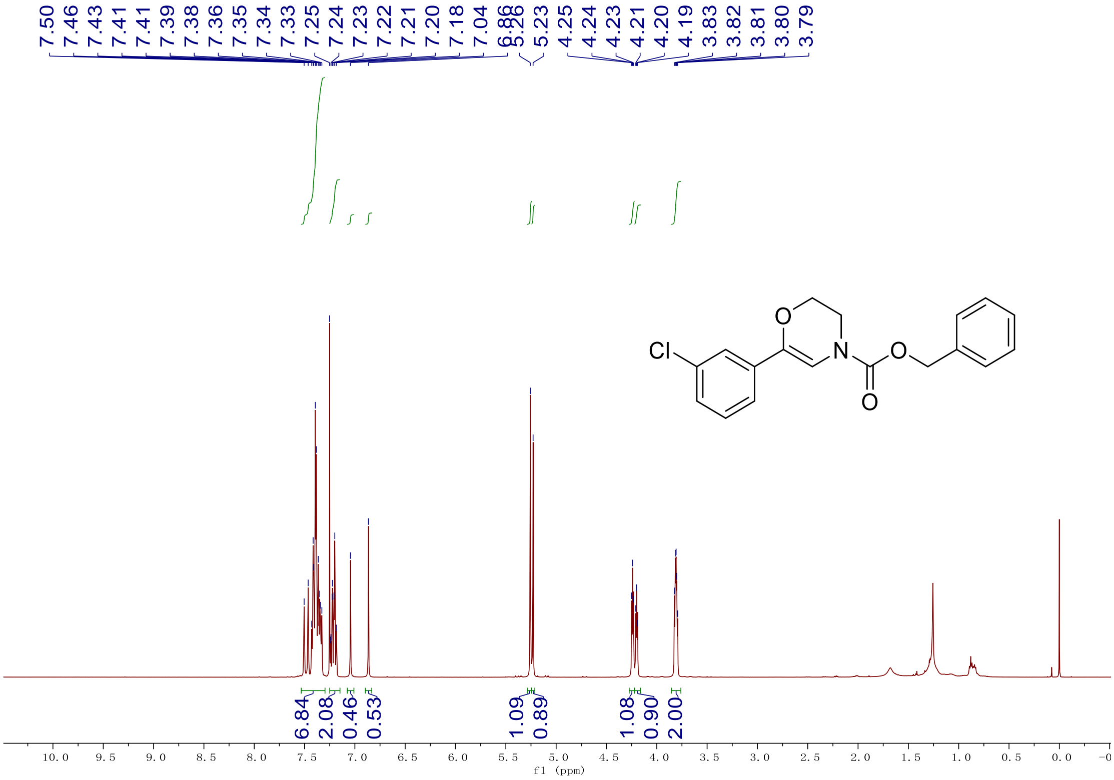

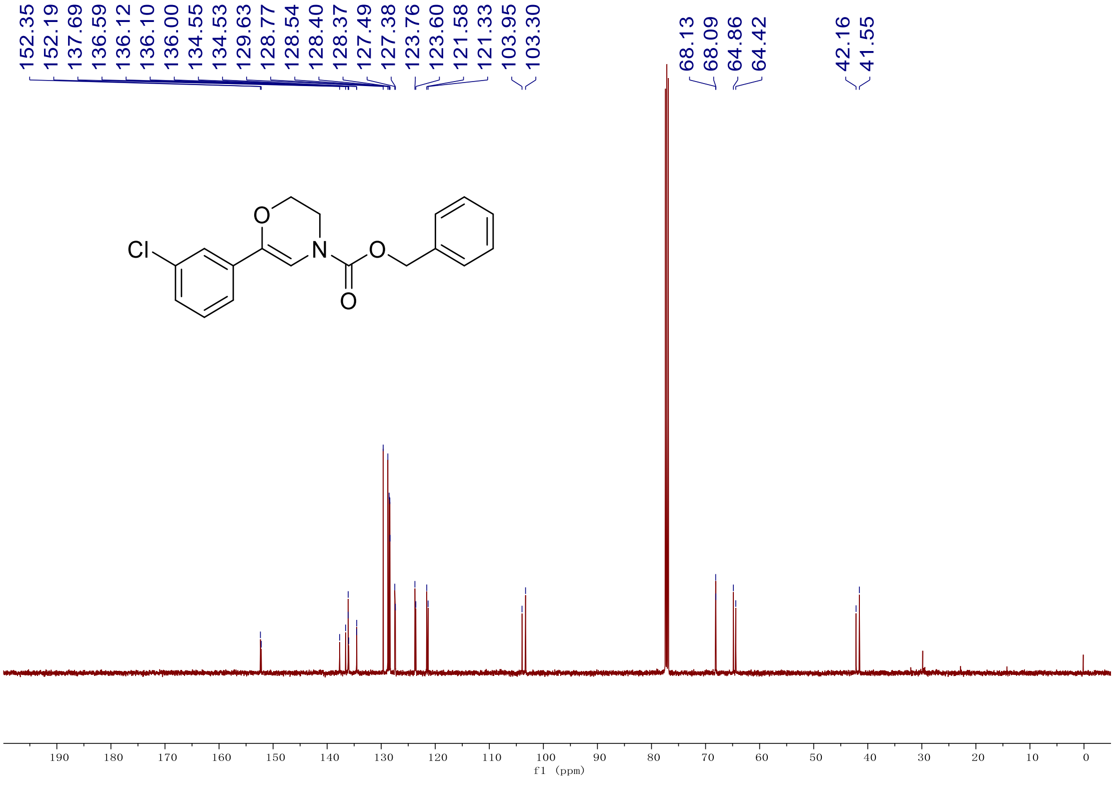

Benzyl 6-(3-bromophenyl)-2,3-dihydro-4*H*-1,4-oxazine-4-carboxylate (1k)

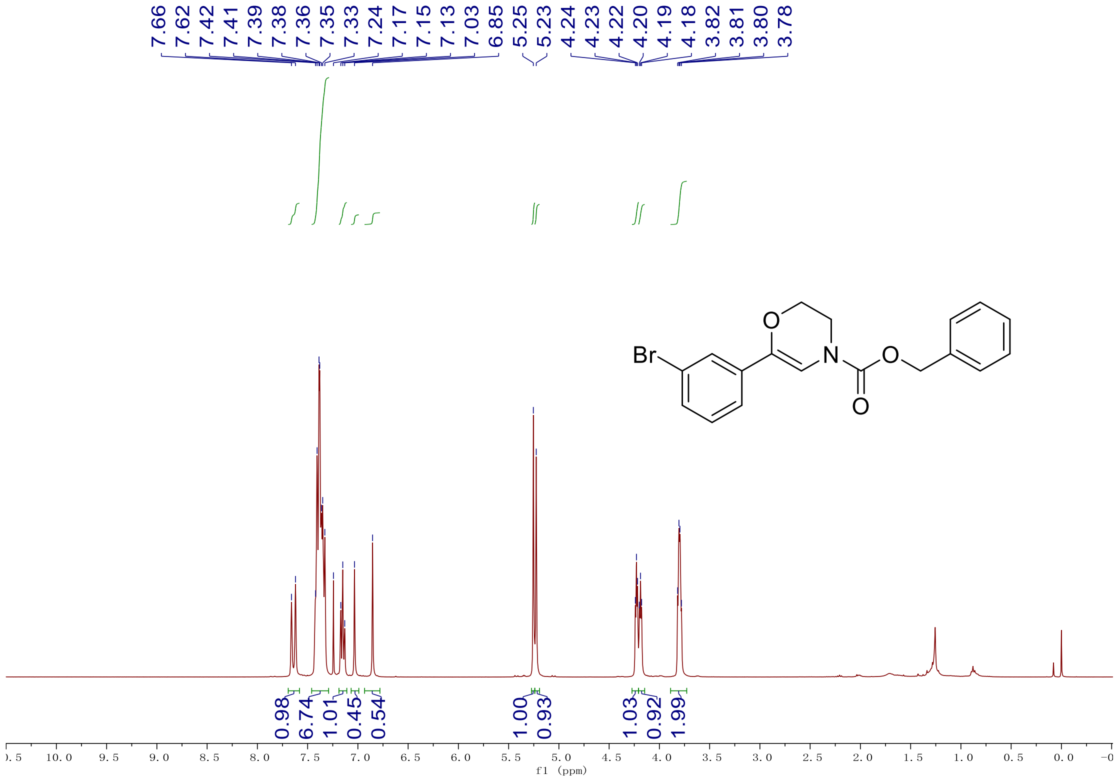

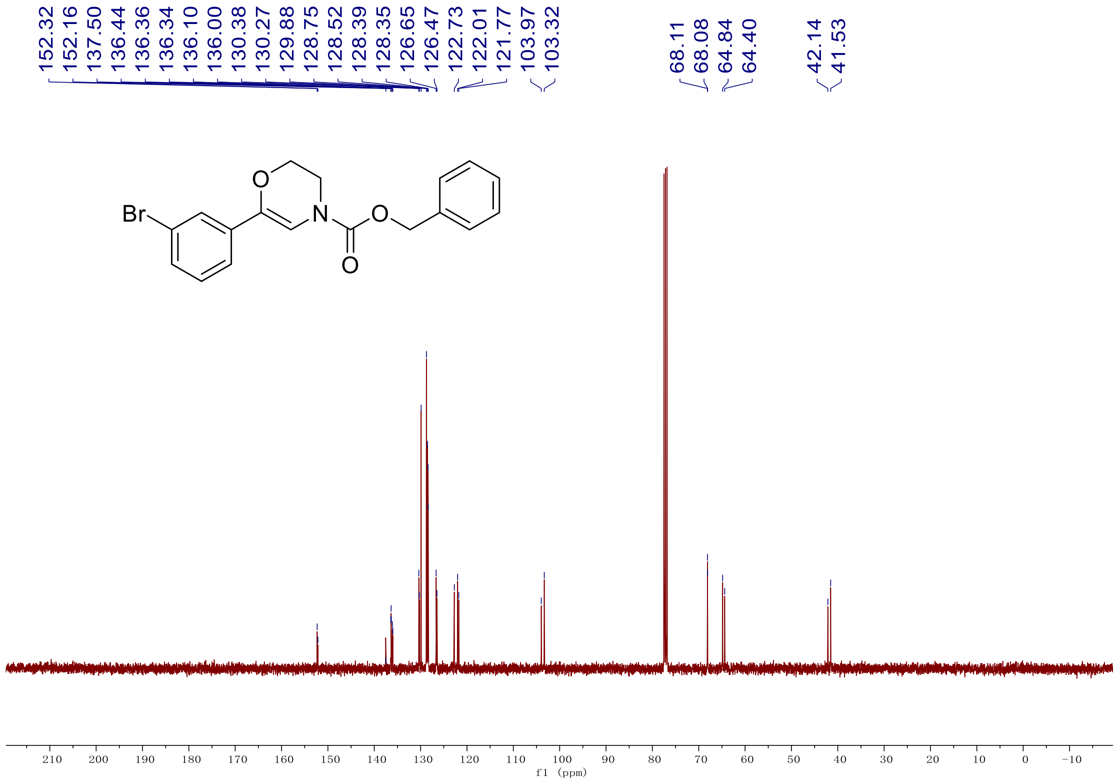

Benzyl 6-(3-methoxyphenyl)-2,3-dihydro-4*H*-1,4-oxazine-4-carboxylate (1l)

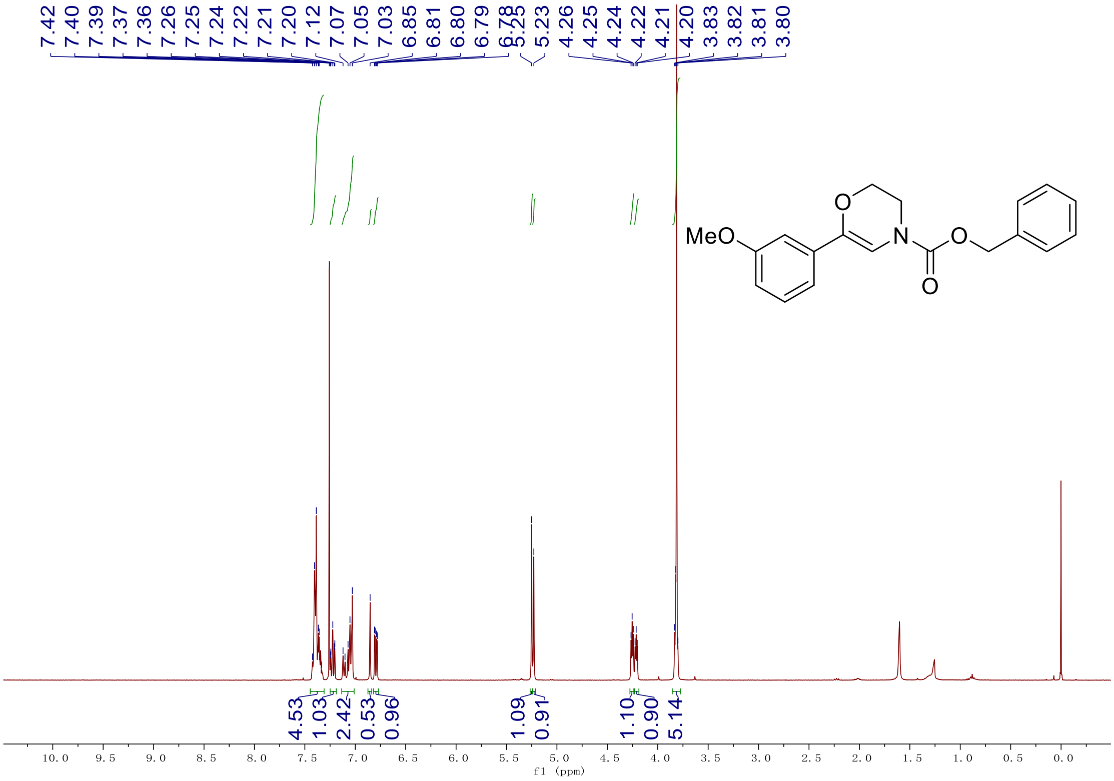

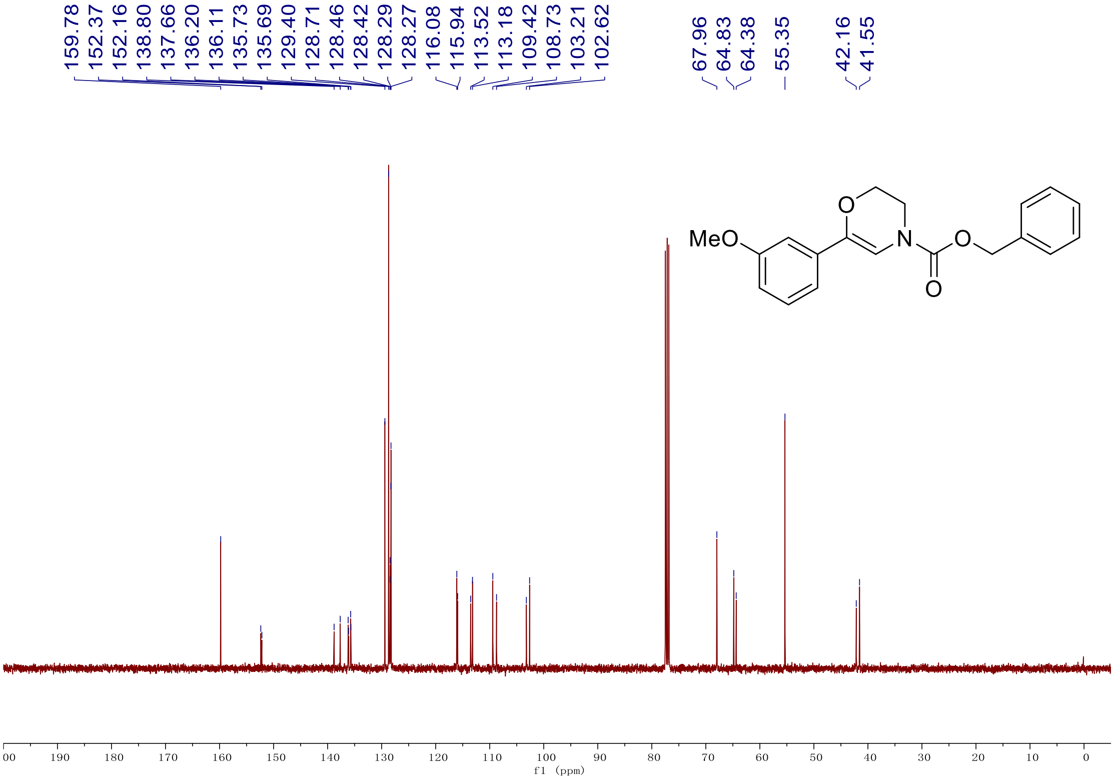

Benzyl 6-(2-fluorophenyl)-2,3-dihydro-4*H*-1,4-oxazine-4-carboxylate (1m)

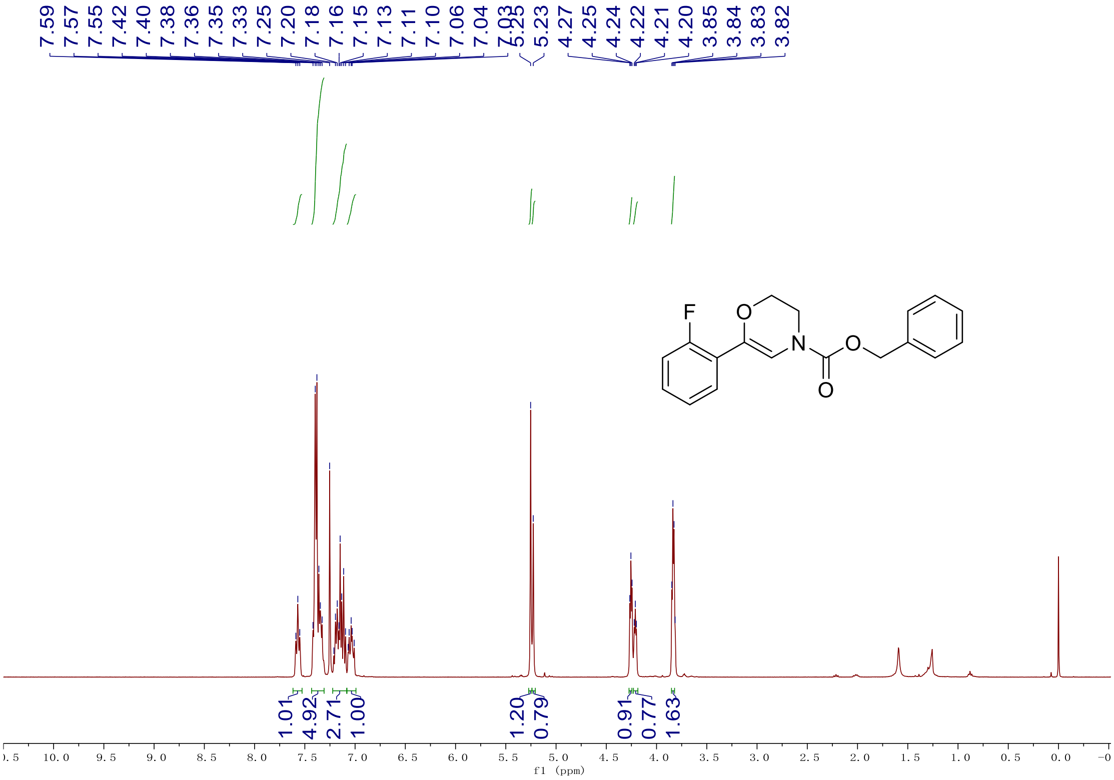

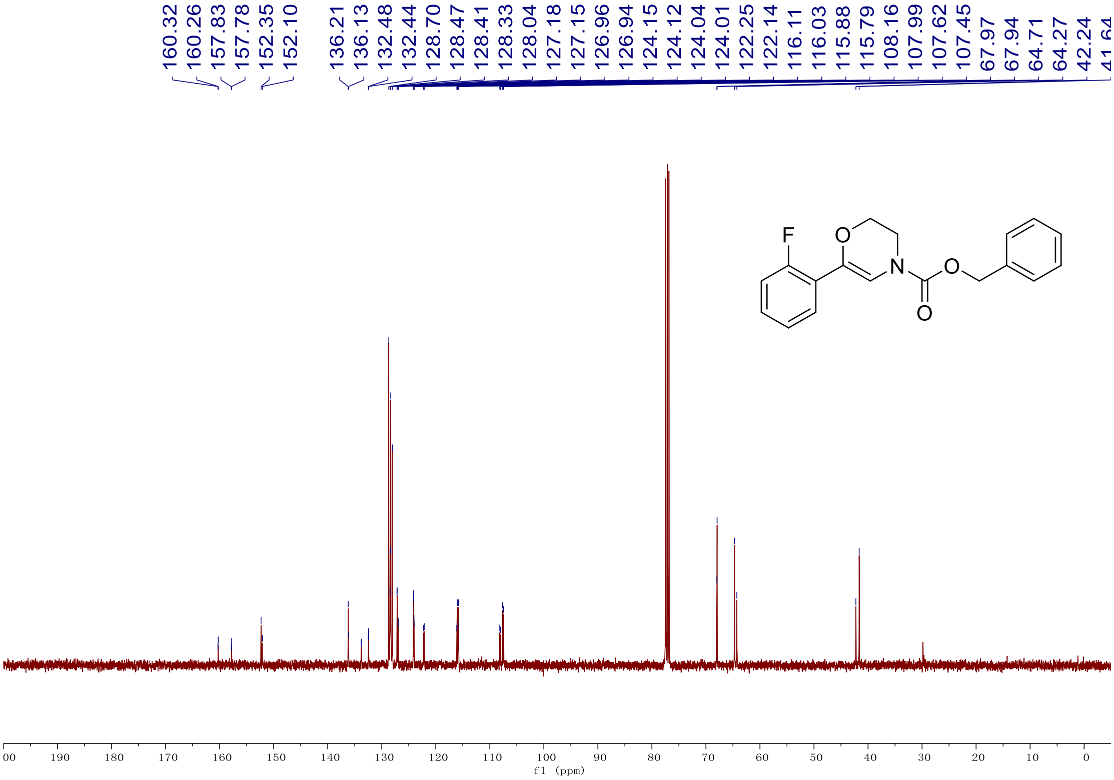

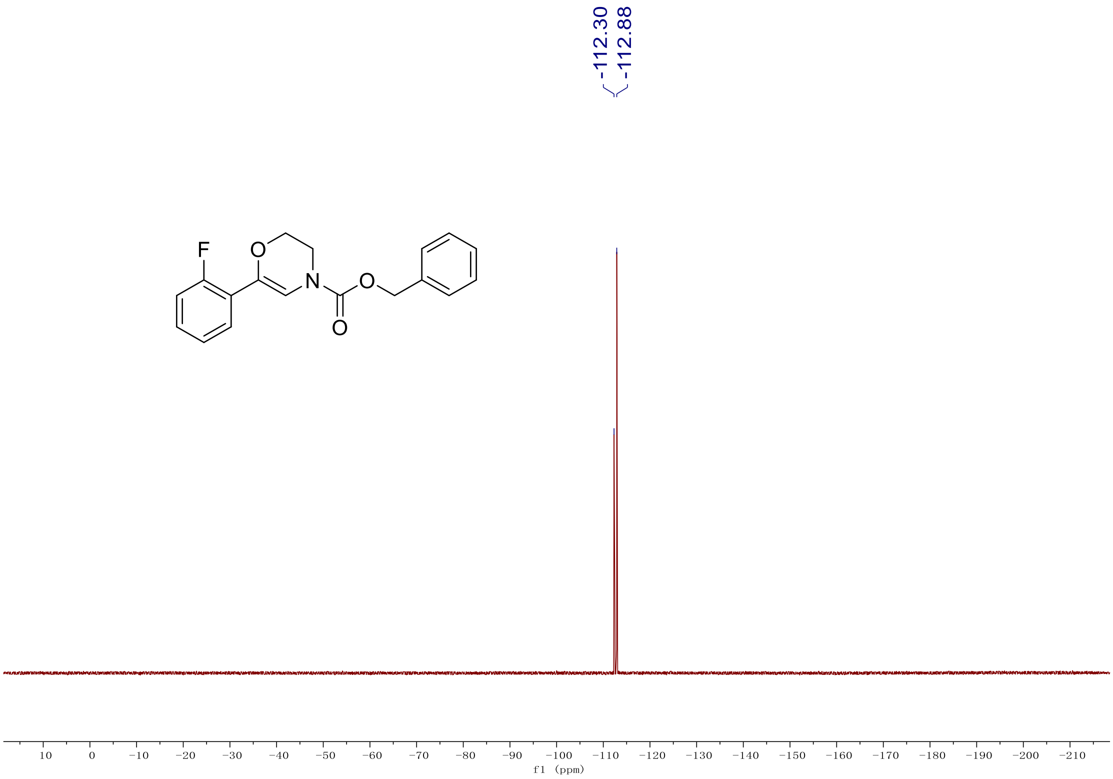

-112.30
-112.88
F
O
N
O
O
10
0
−10
−20
−30
−40
−50
−60
−70
−80
−90
−100
−110
−120
−130
−140
−150
−160
−170
−180
−190
−200
−210
f1 (ppm)

Benzyl 6-(2-chlorophenyl)-2,3-dihydro-4*H*-1,4-oxazine-4-carboxylate (1n)

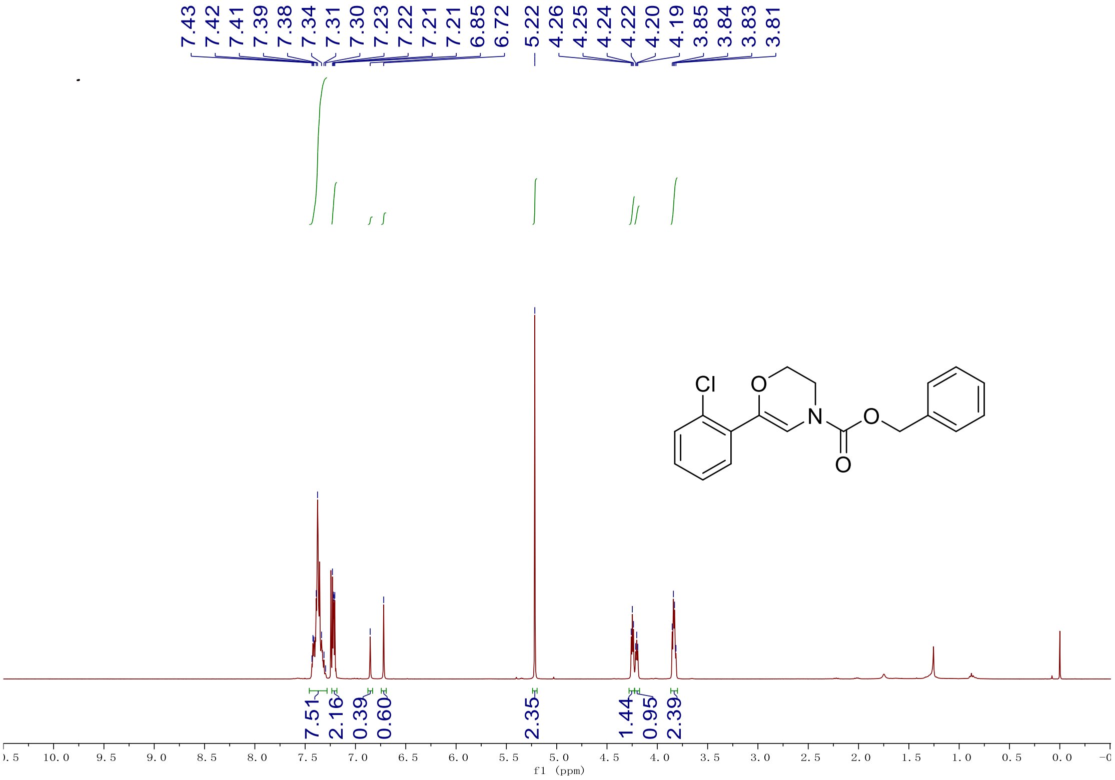

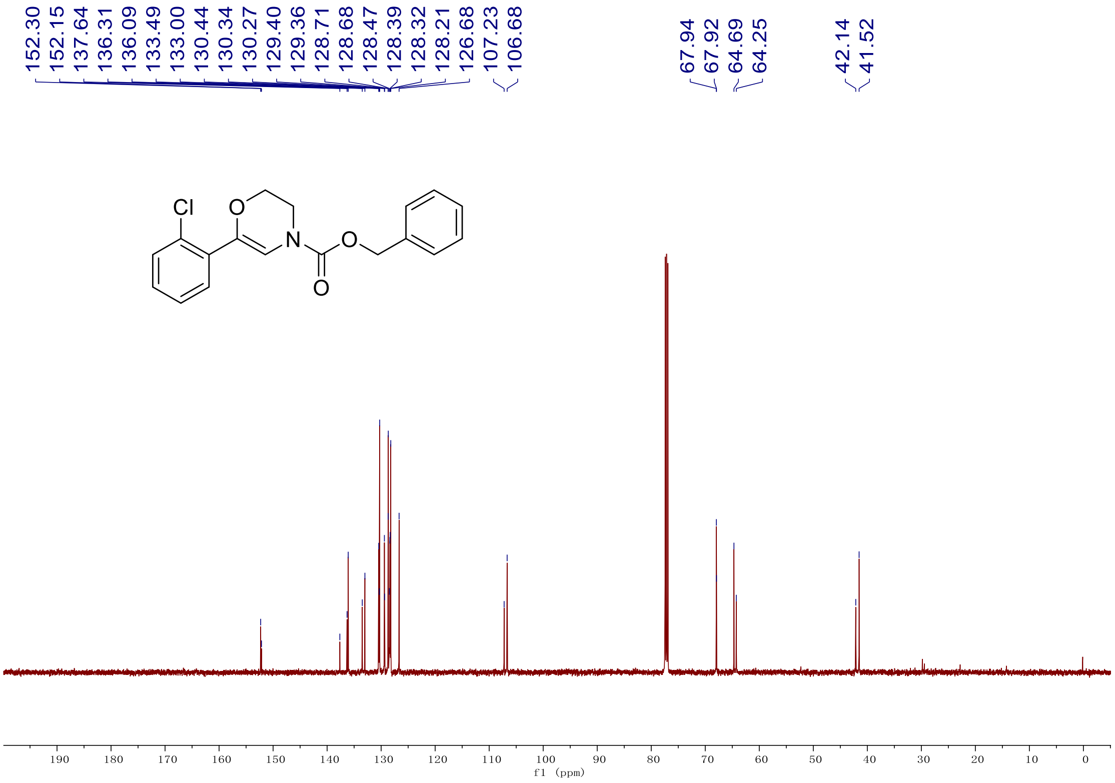

Benzyl 6-(*o*-tolyl)-2,3-dihydro-4*H*-1,4-oxazine-4-carboxylate (1o)

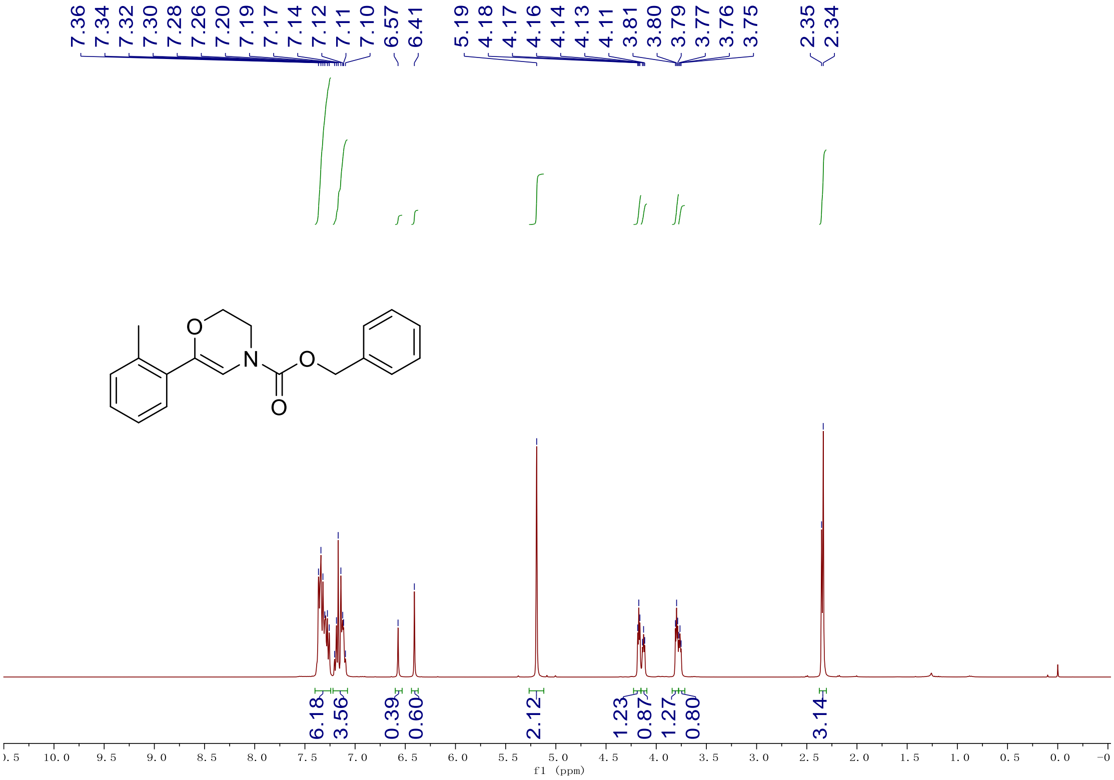

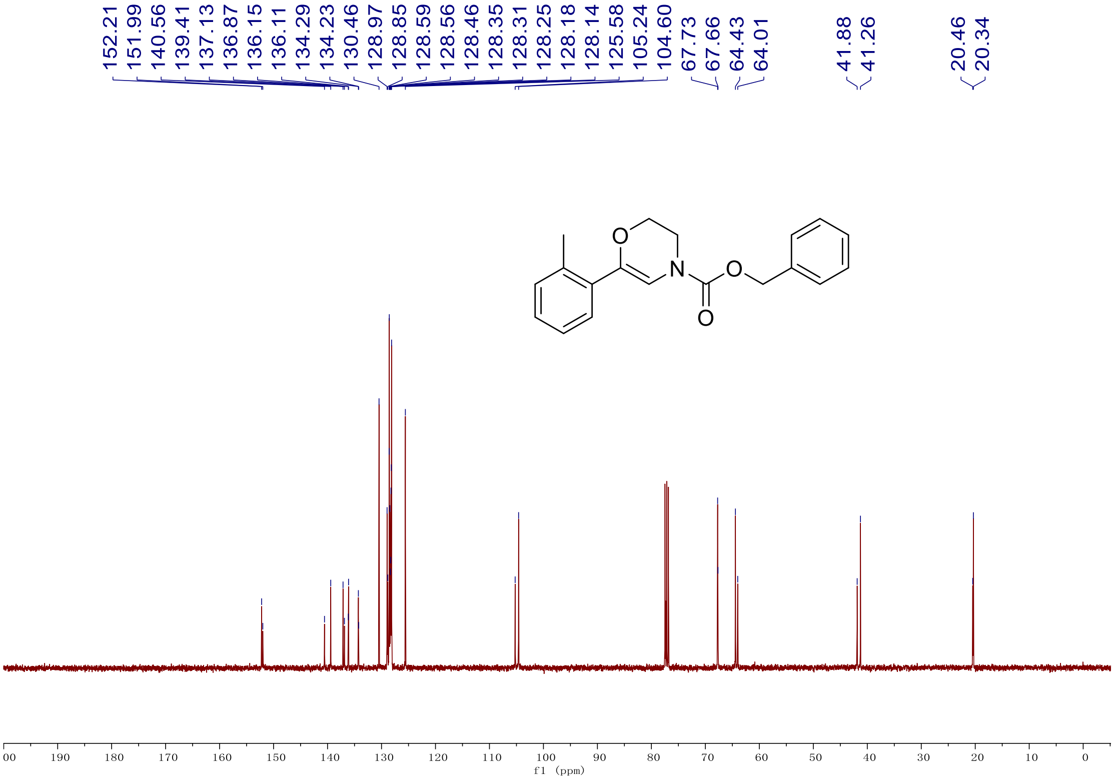

Benzyl 6-(2-methoxyphenyl)-2,3-dihydro-4*H*-1,4-oxazine-4-carboxylate (1p)

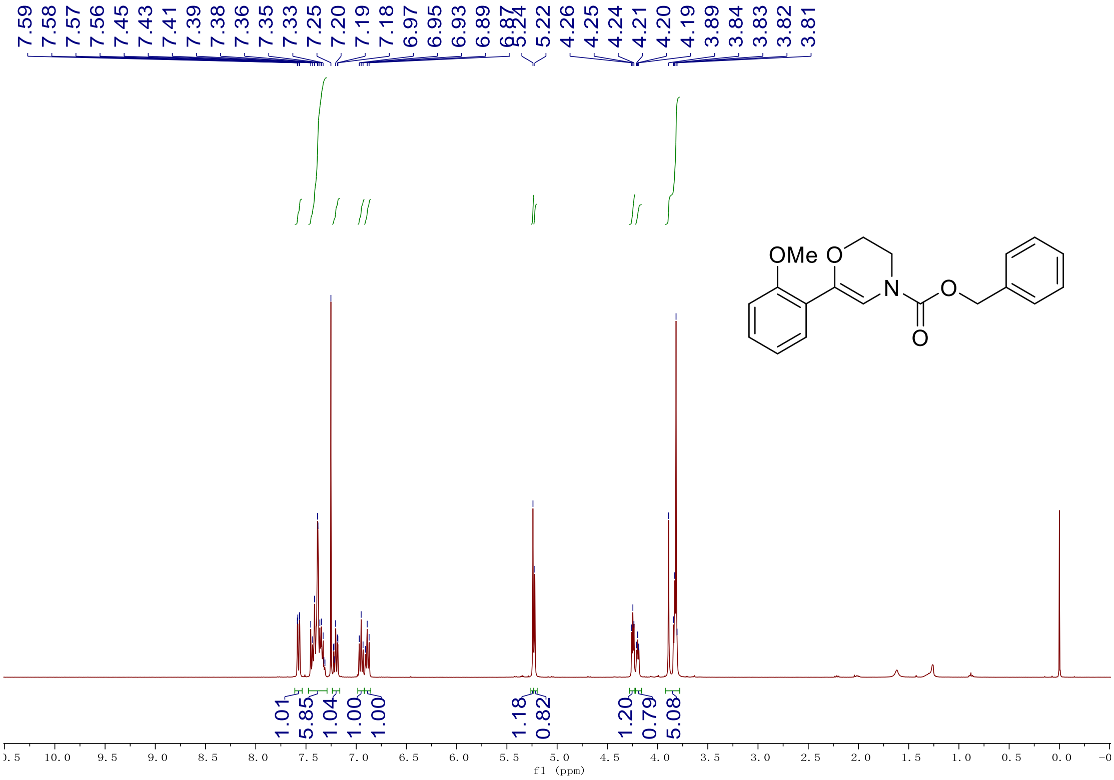

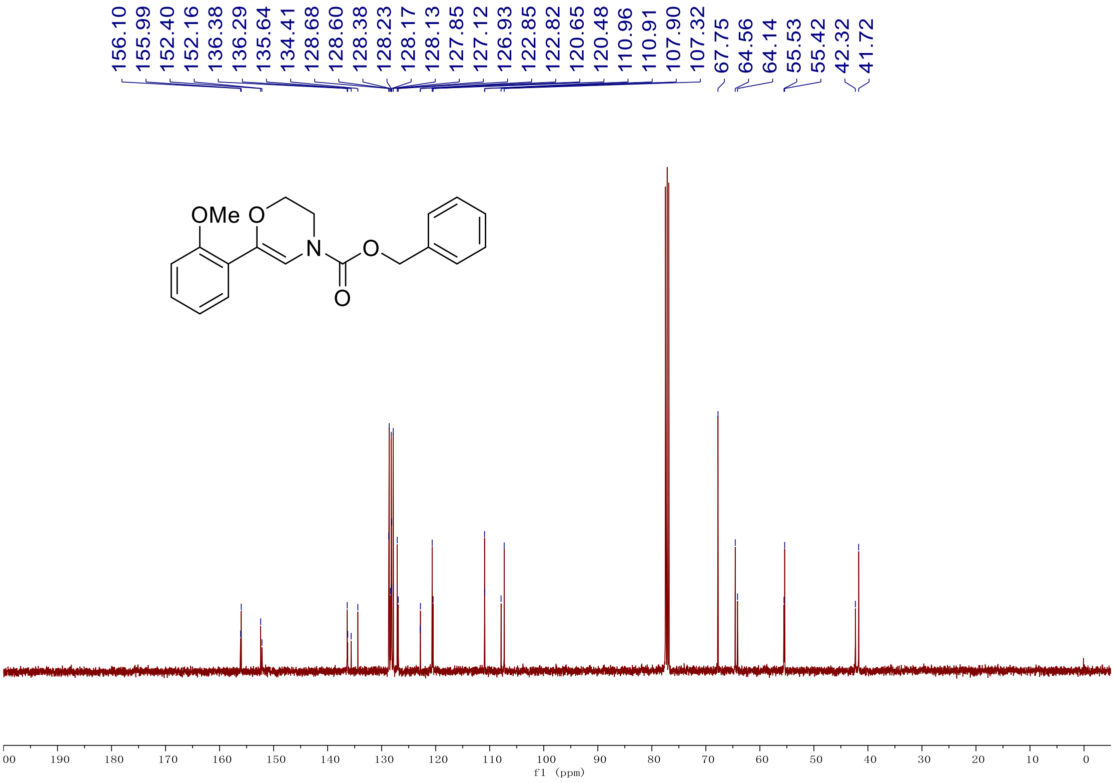

Benzyl 6-(3,4-dichlorophenyl)-2,3-dihydro-4*H*-1,4-oxazine-4-carboxylate (1q)

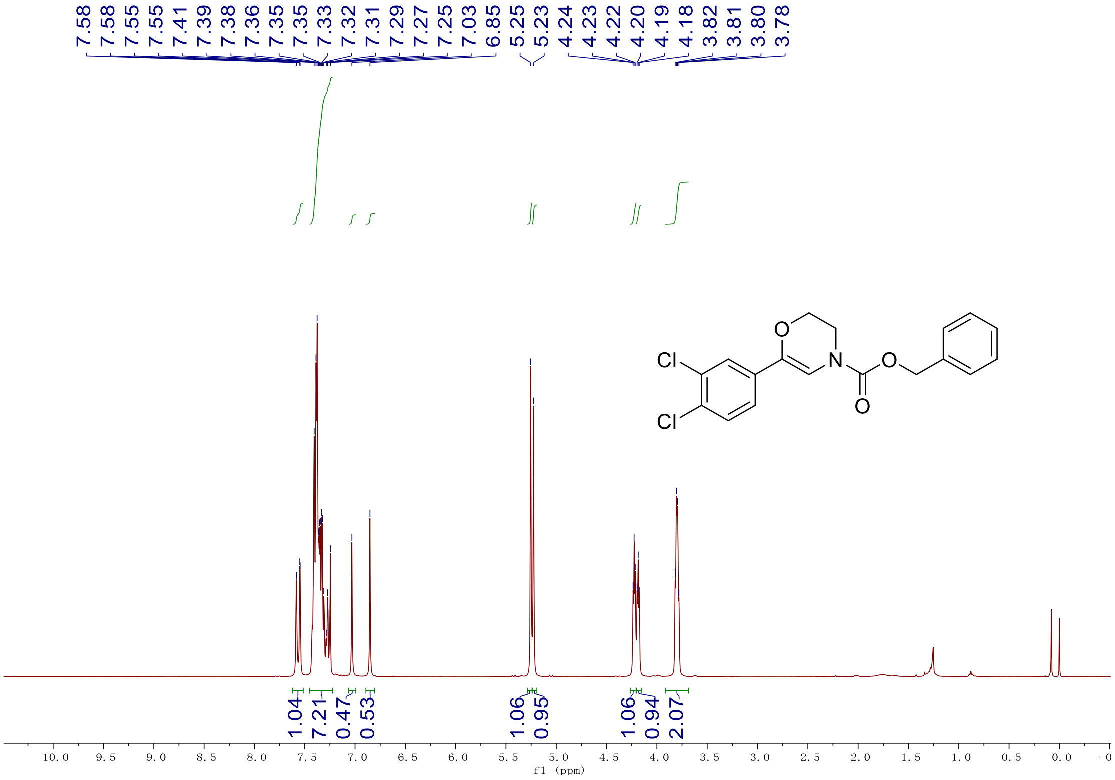

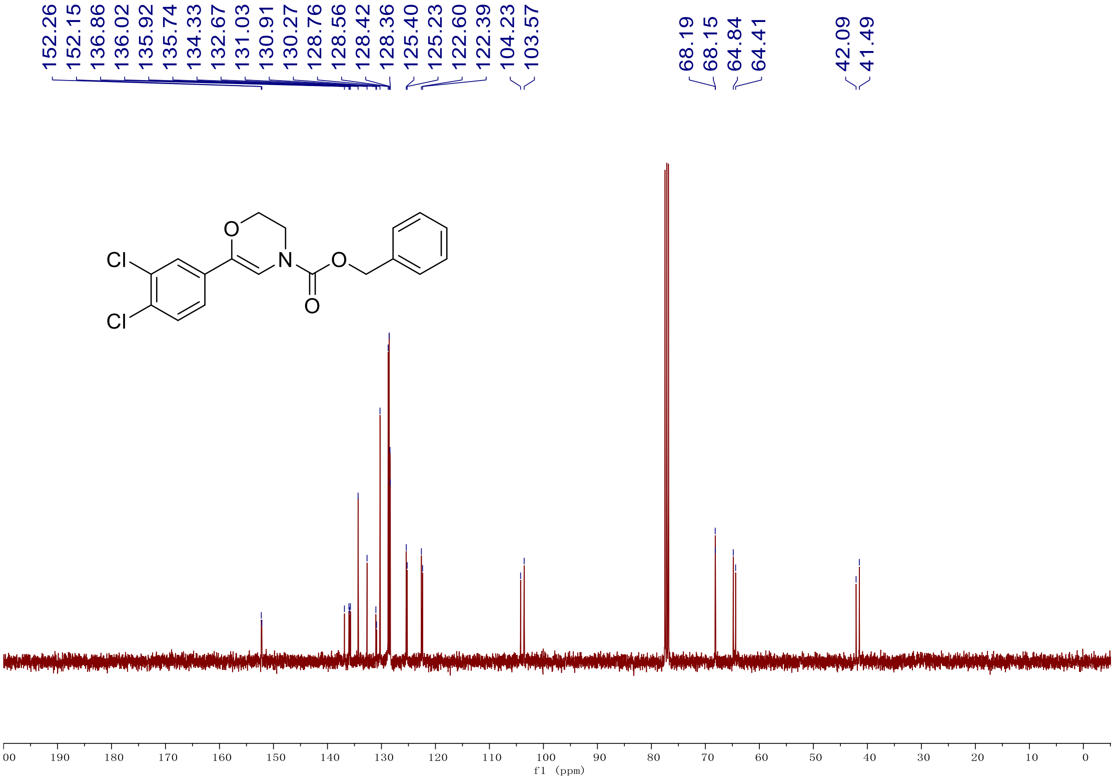

Benzyl 6-(3,4-dimethoxyphenyl)-2,3-dihydro-4*H*-1,4-oxazine-4-carboxylate (1r)

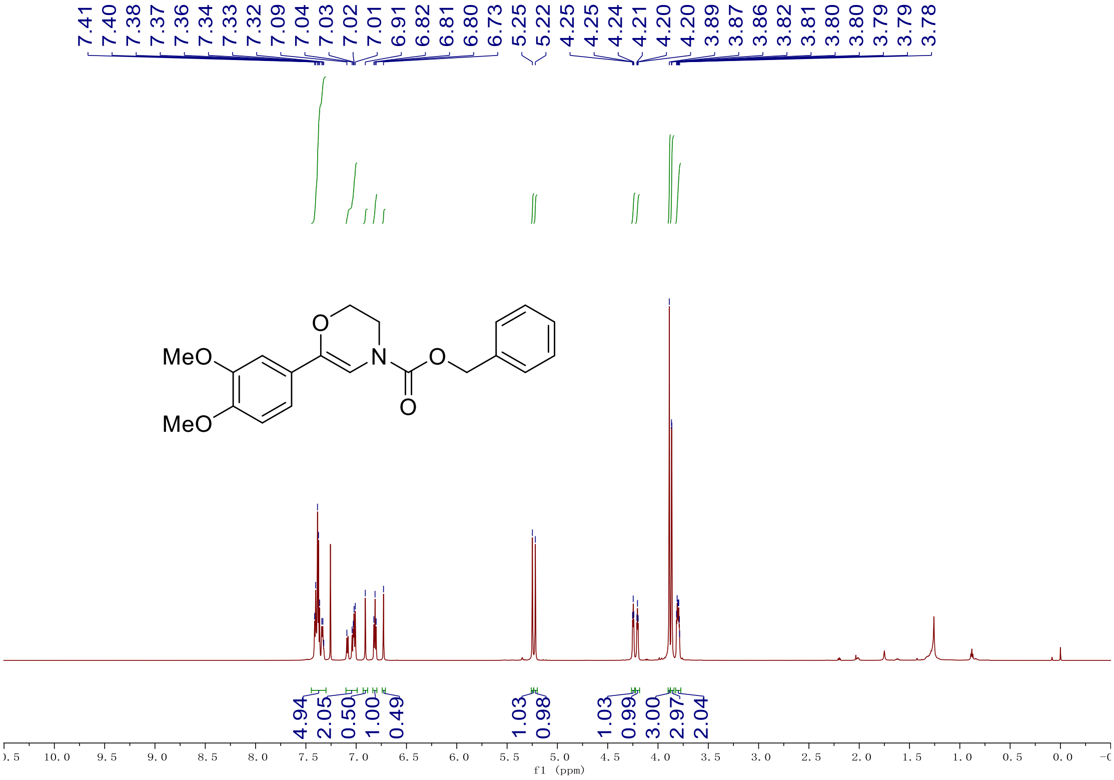

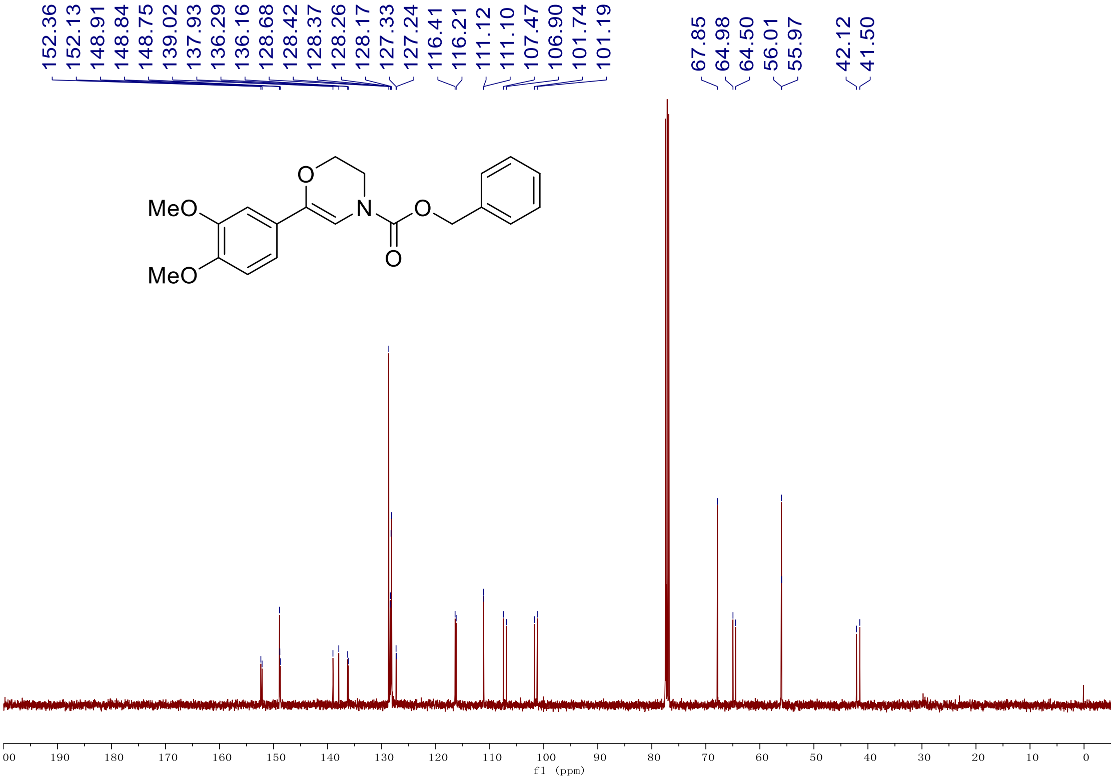

Benzyl 6-(naphthalen-2-yl)-2,3-dihydro-4*H*-1,4-oxazine-4-carboxylate (1s)

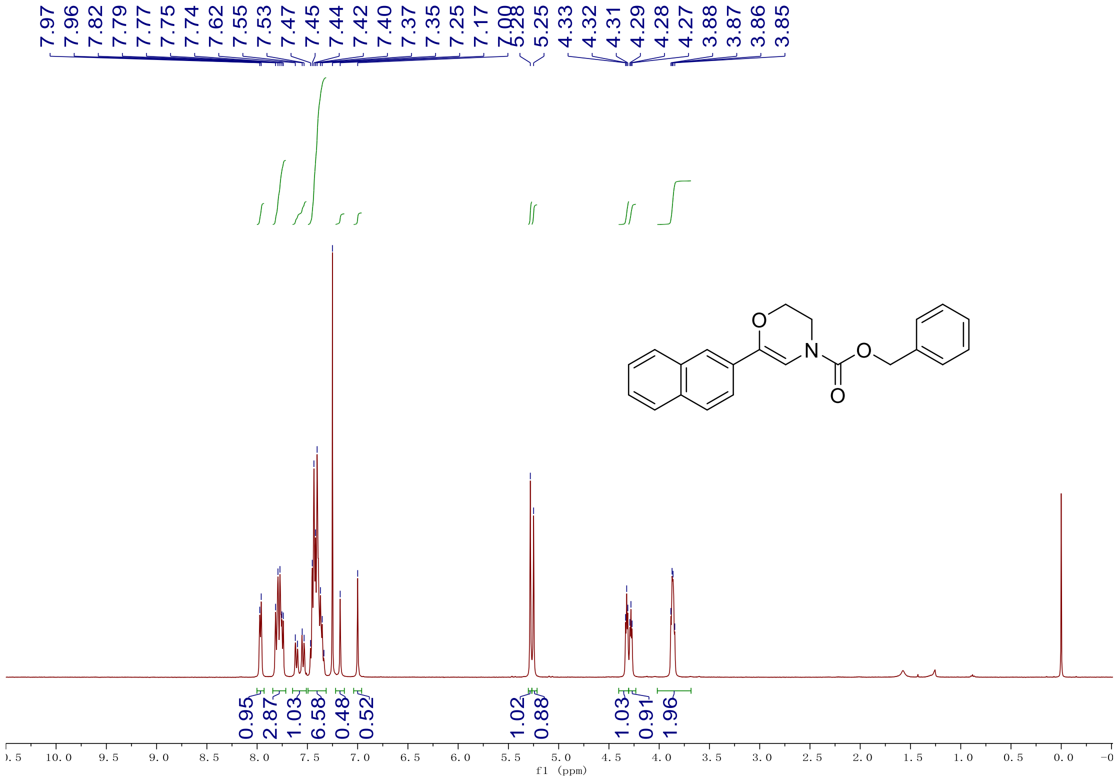

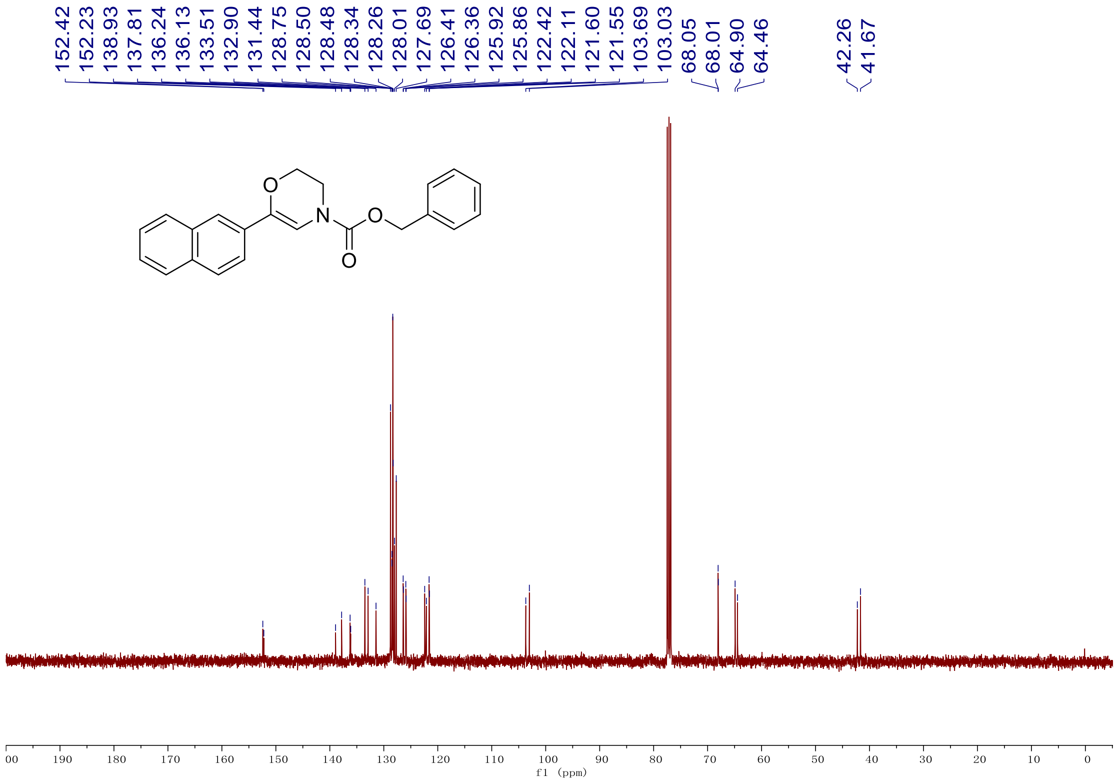

Benzyl 6-(thiophen-2-yl)-2,3-dihydro-4*H*-1,4-oxazine-4-carboxylate (1t)

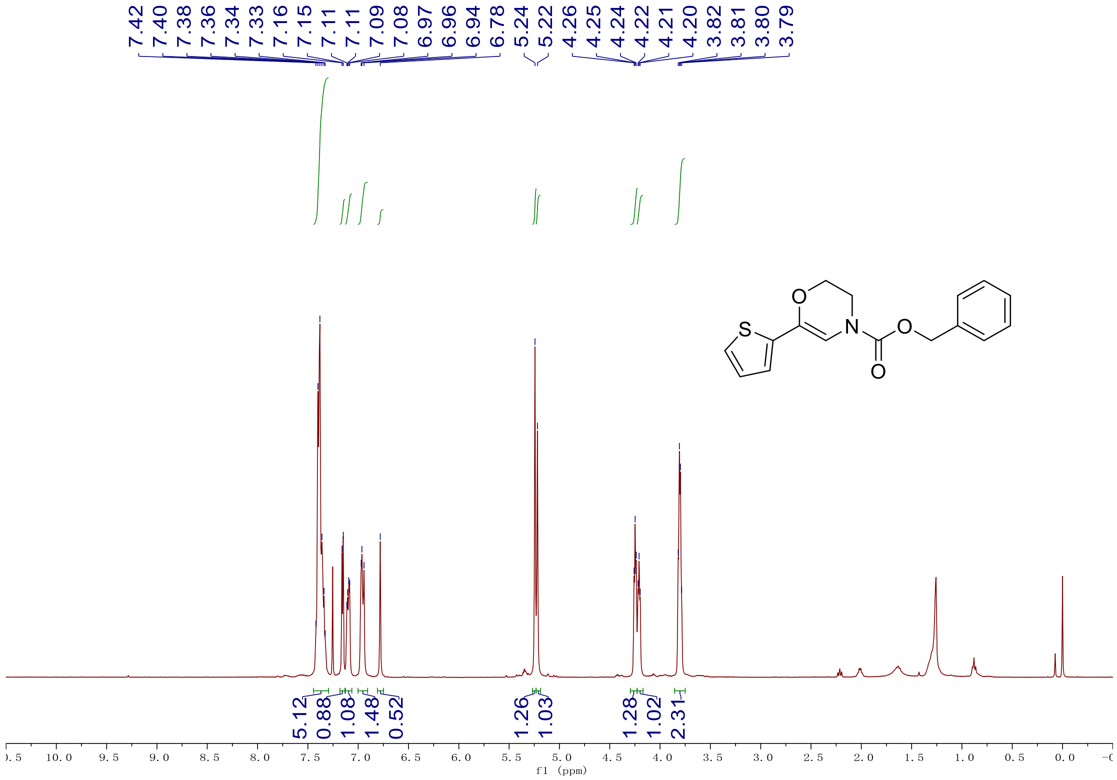

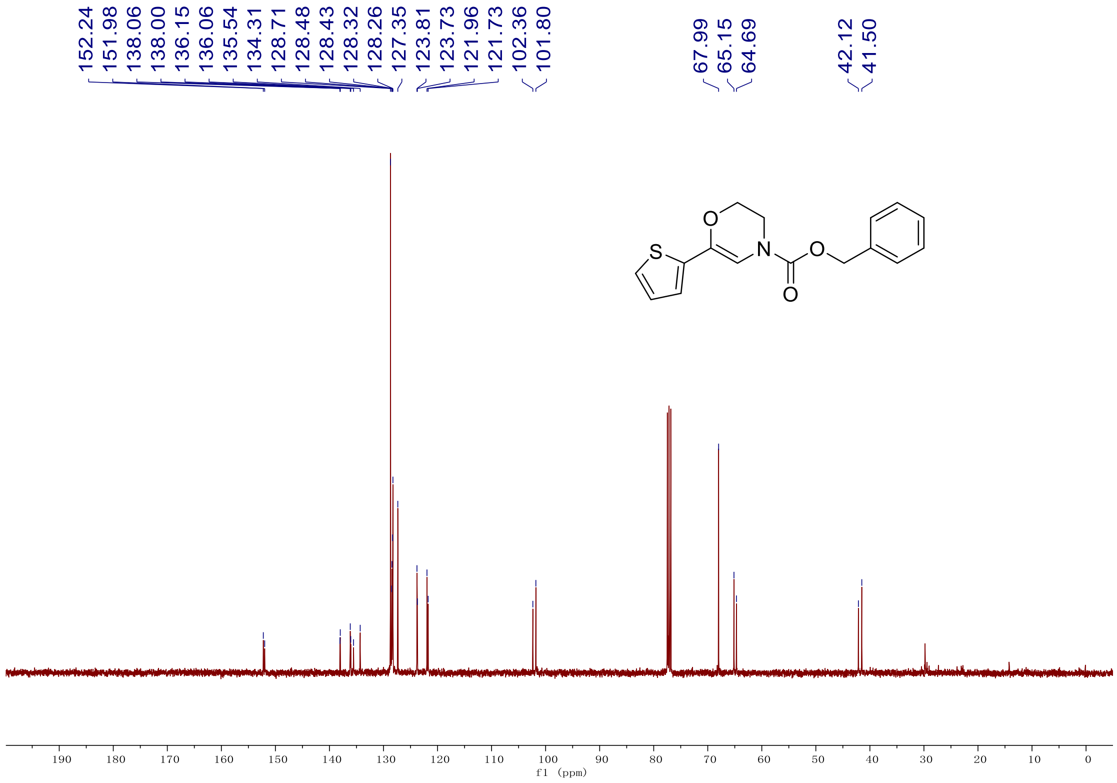

Benzyl 6-(*tert*-butyl)-2,3-dihydro-4*H*-1,4-oxazine-4-carboxylate (1u)

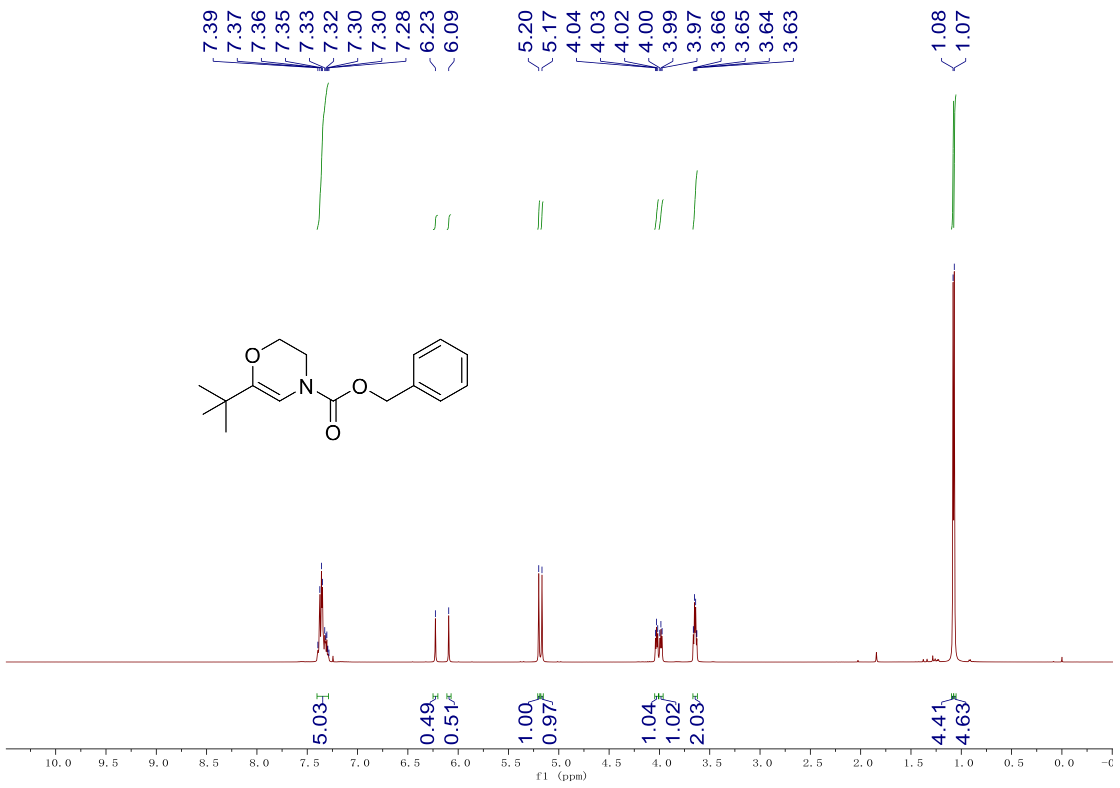

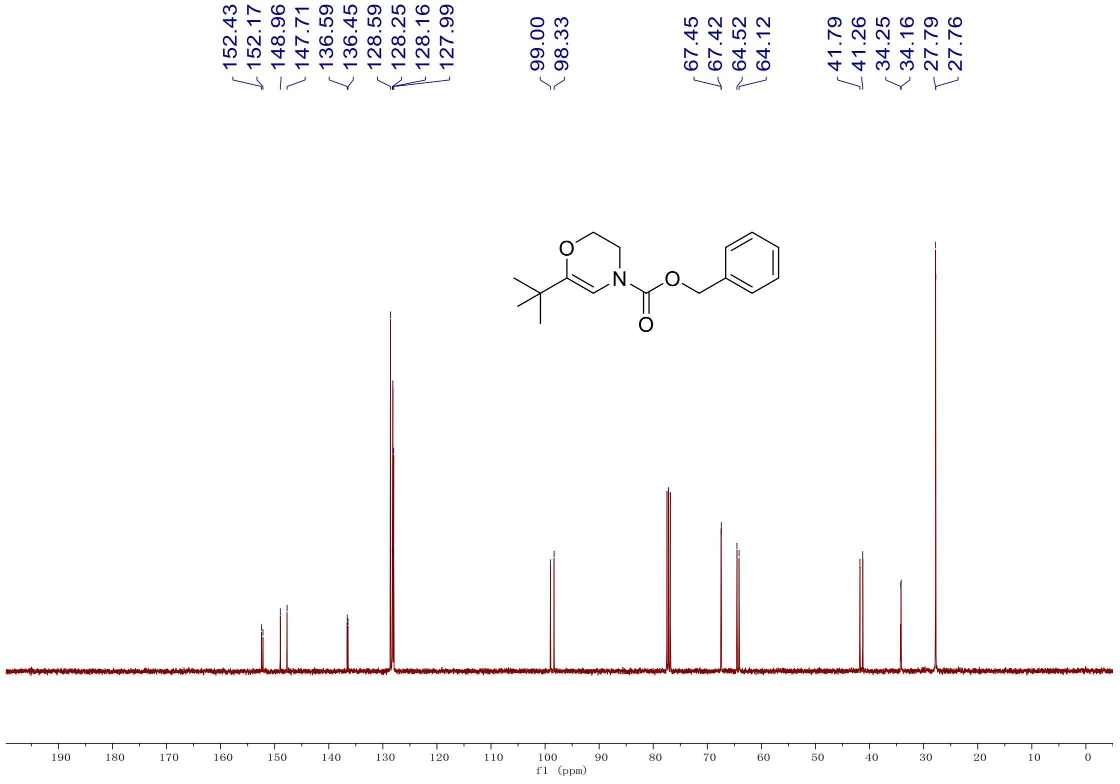

Benzyl 6-isopropyl-2,3-dihydro-4*H*-1,4-oxazine-4-carboxylate (1v)

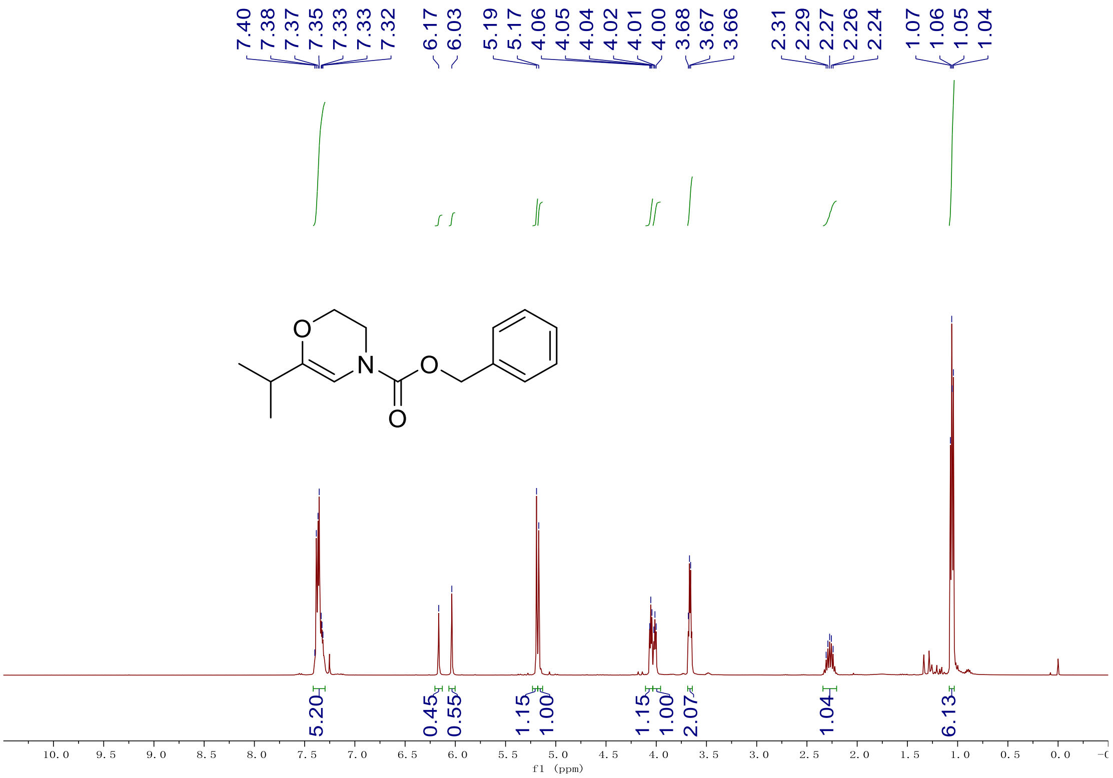

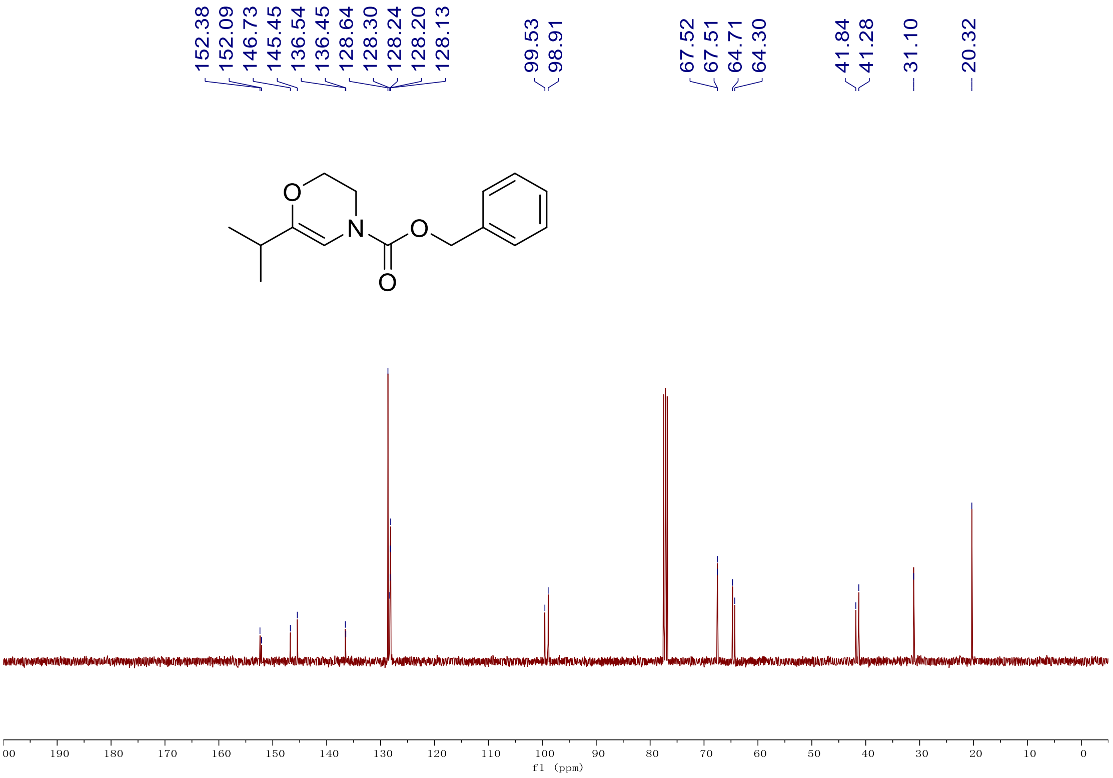

Benzyl 6-ethyl-2,3-dihydro-4*H*-1,4-oxazine-4-carboxylate (1w)

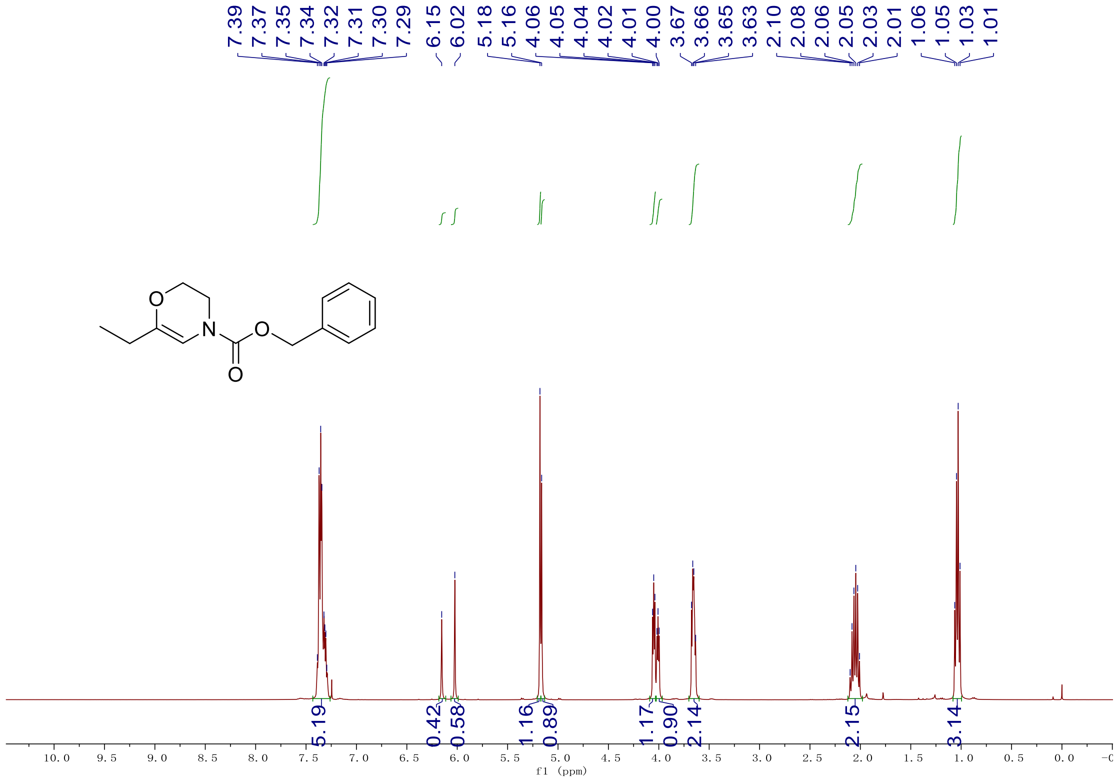

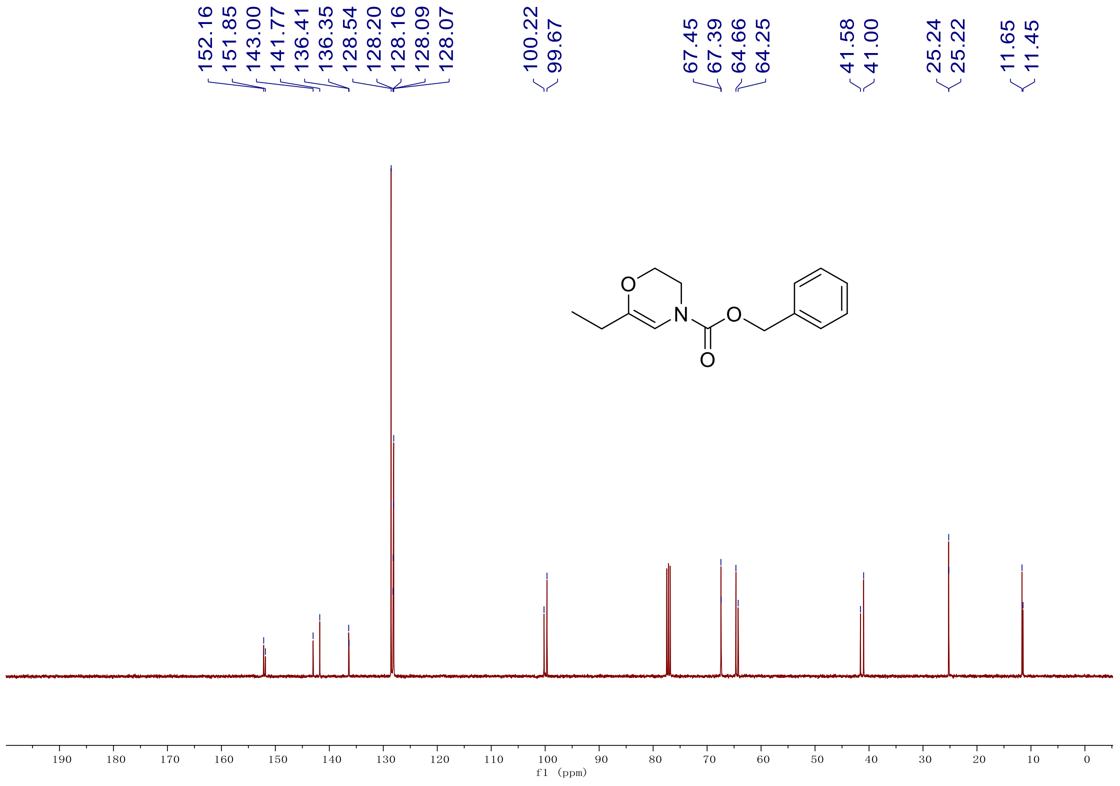

Benzyl 5-phenyl-2,3-dihydro-4*H*-1,4-oxazine-4-carboxylate (1x)

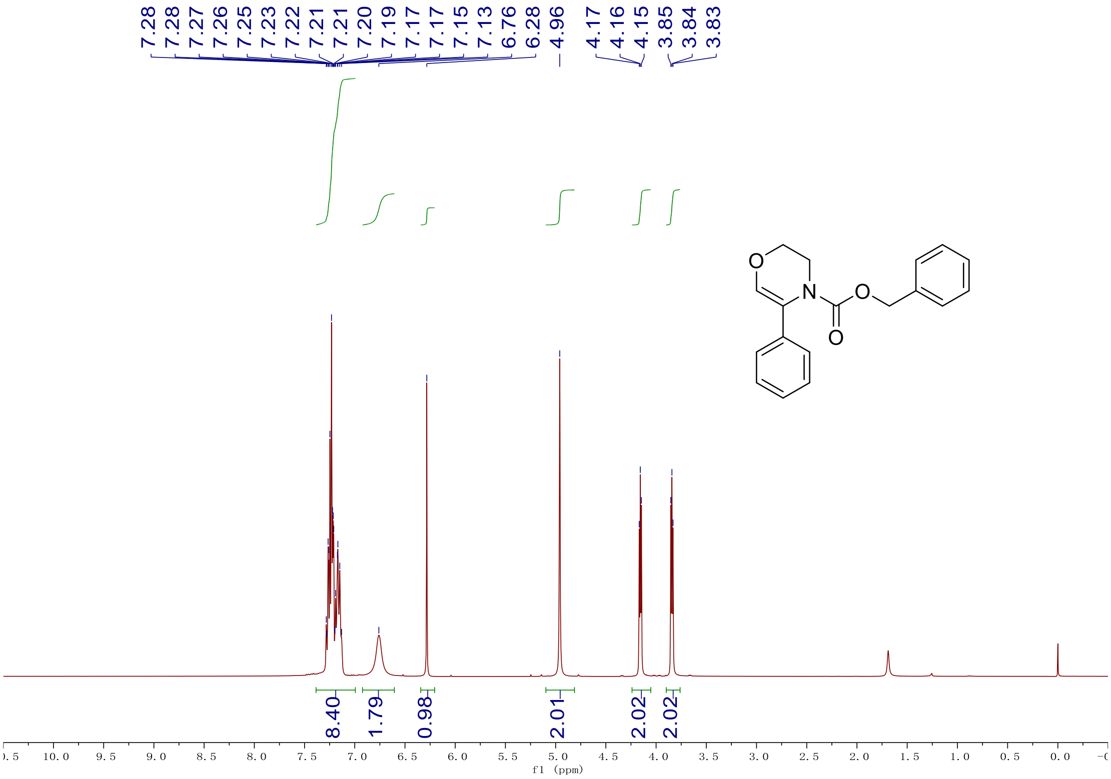

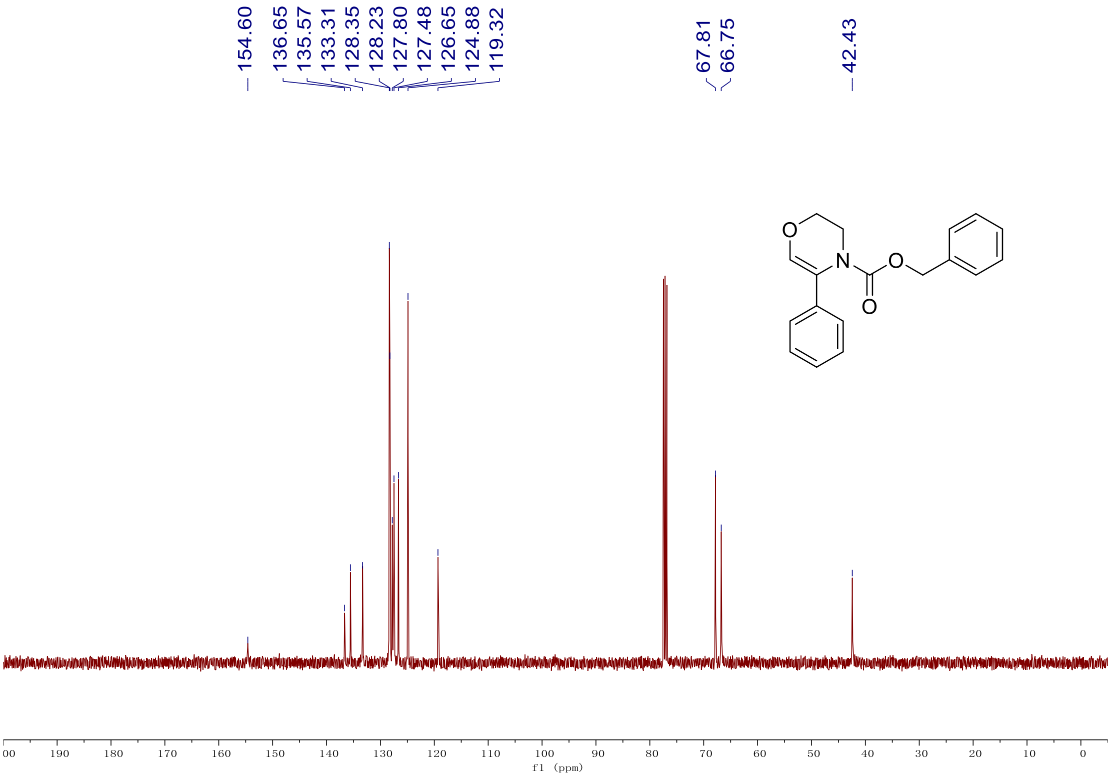

Benzyl 6-(4-bromophenyl)-5-methyl-2,3-dihydro-4*H*-1,4-oxazine-4-carboxylate (1y)

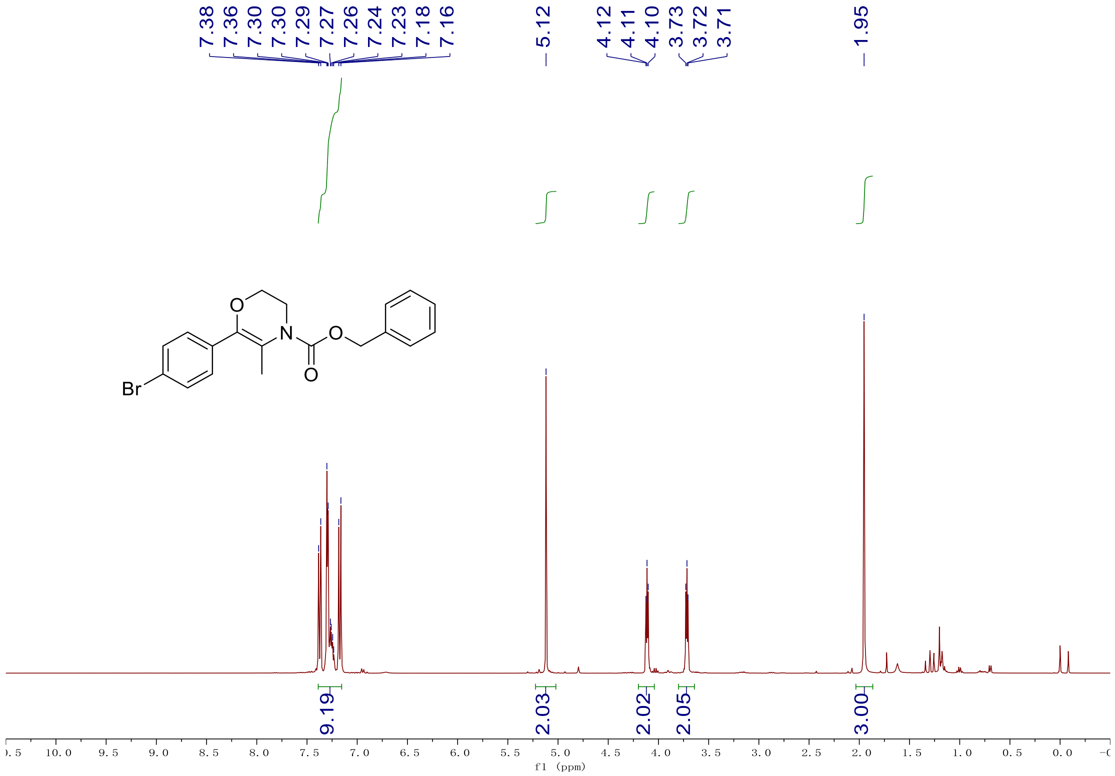

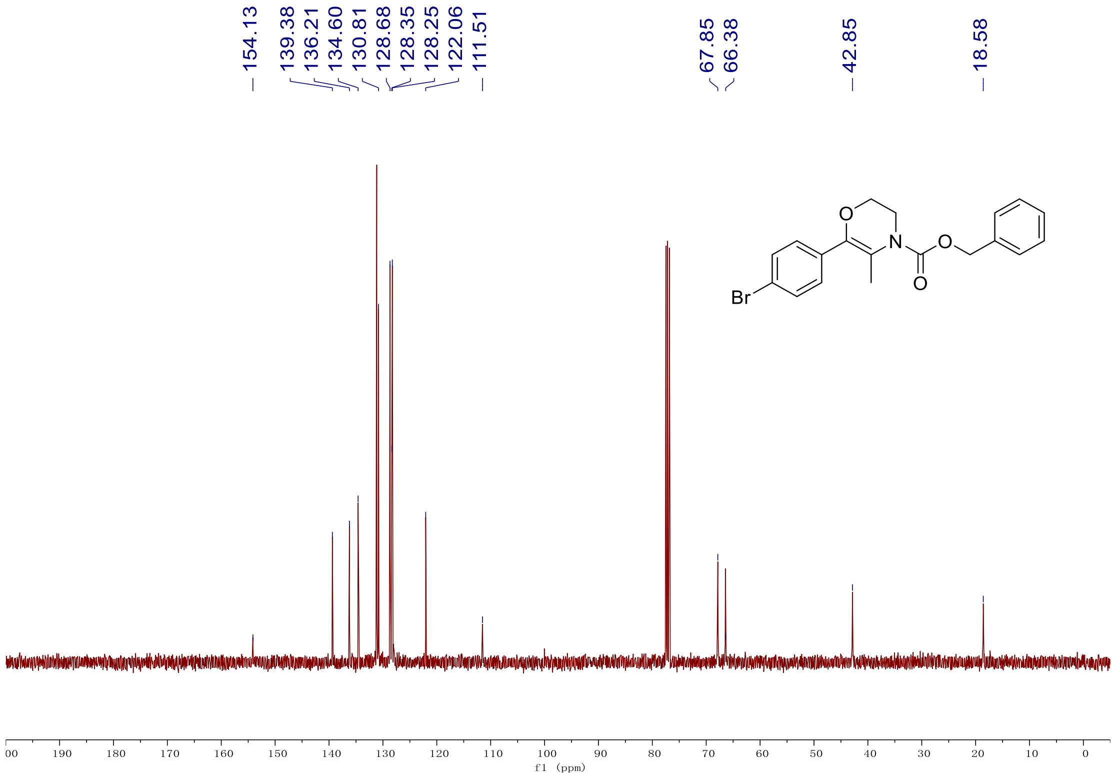

4-Nitrobenzyl 6-phenyl-2,3-dihydro-4*H*-1,4-oxazine-4-carboxylate (1a-NO_2)

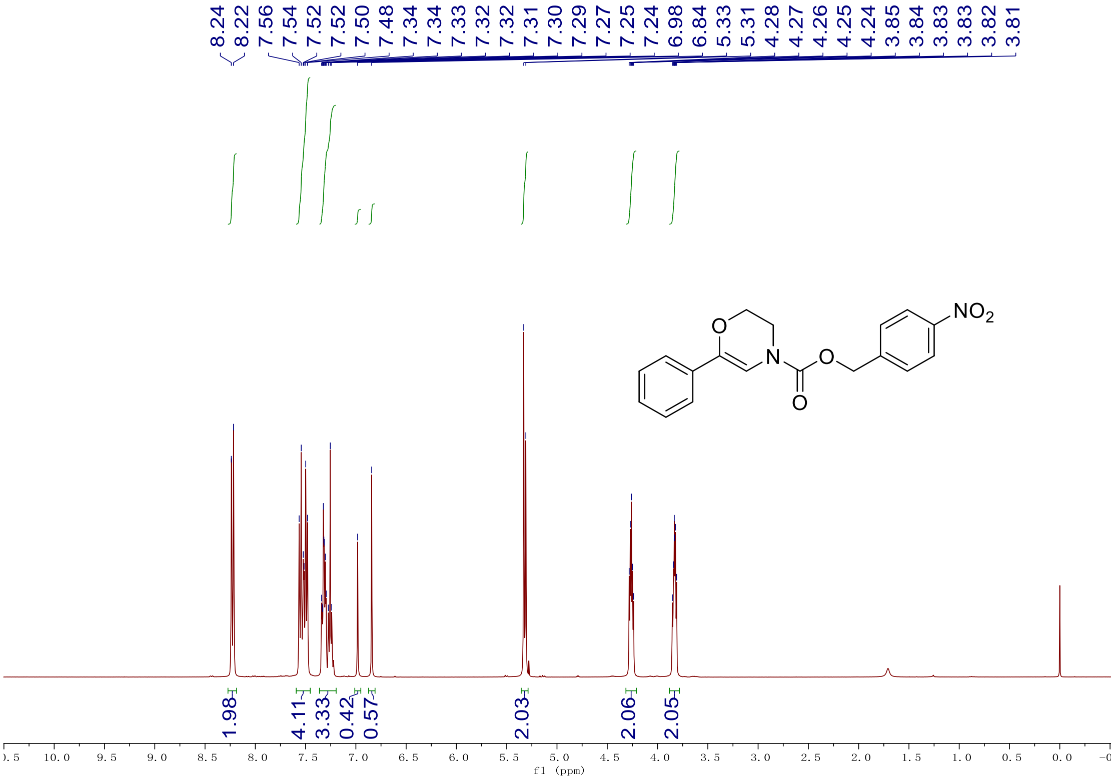

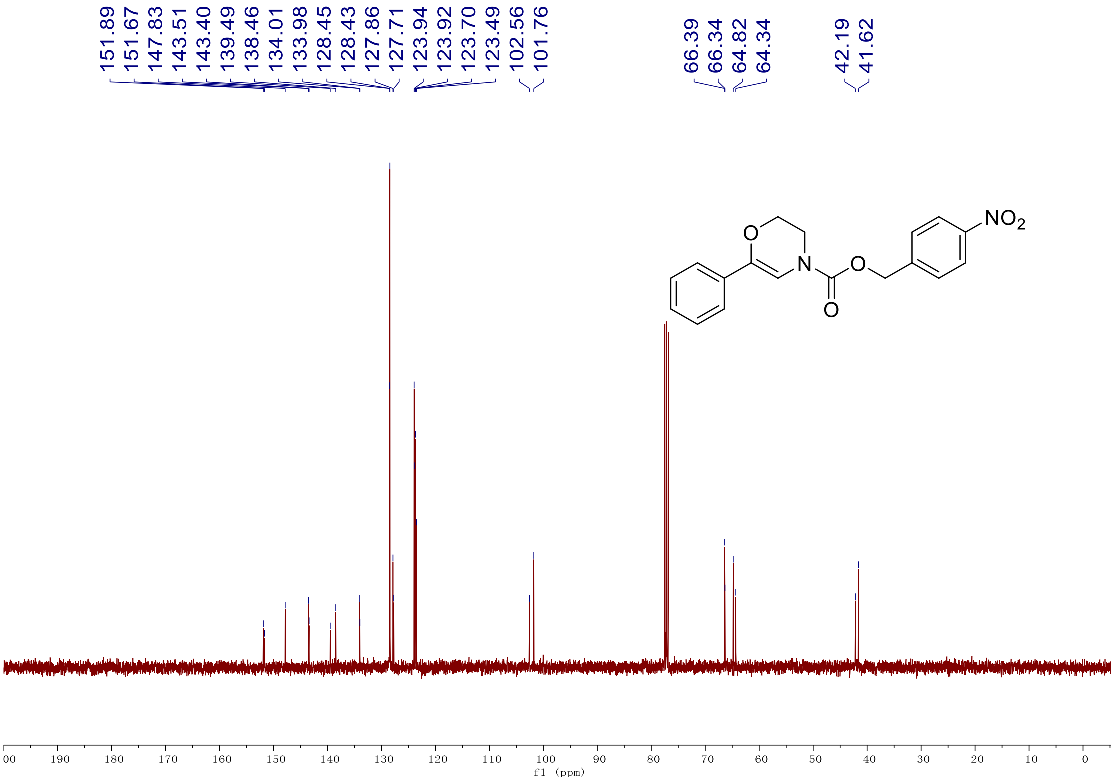

Isobutyl 6-phenyl-2,3-dihydro-4*H*-1,4-oxazine-4-carboxylate (1a-COO*i*Bu)

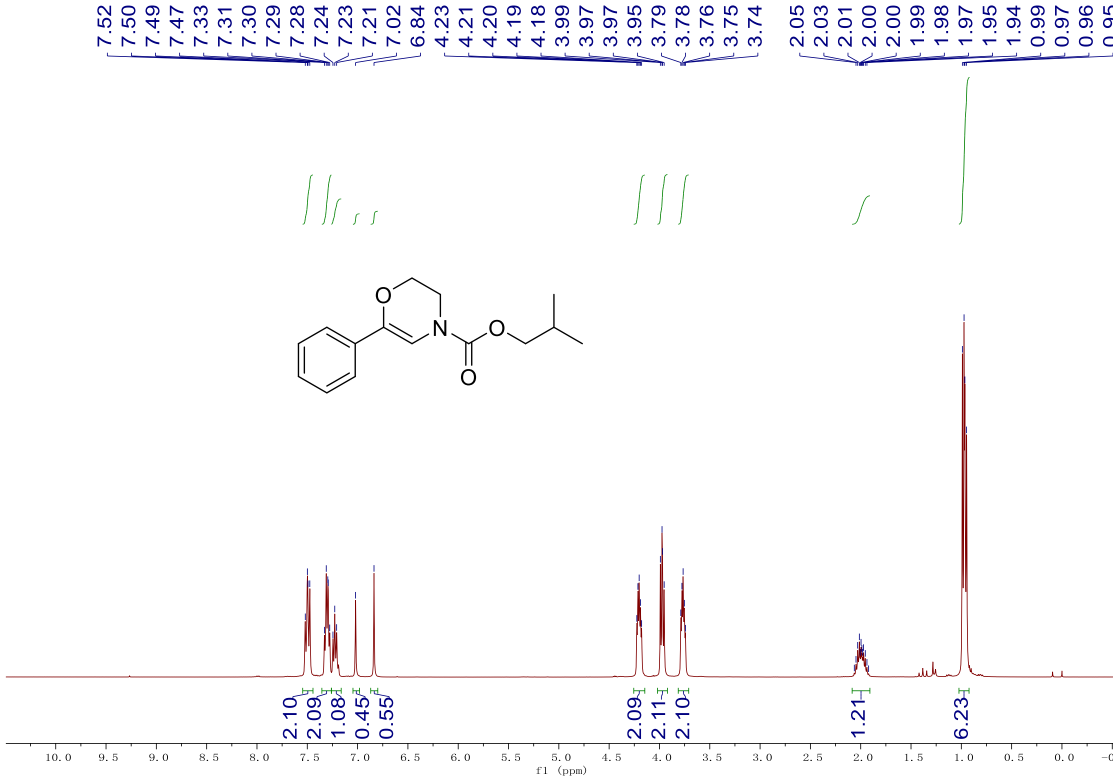

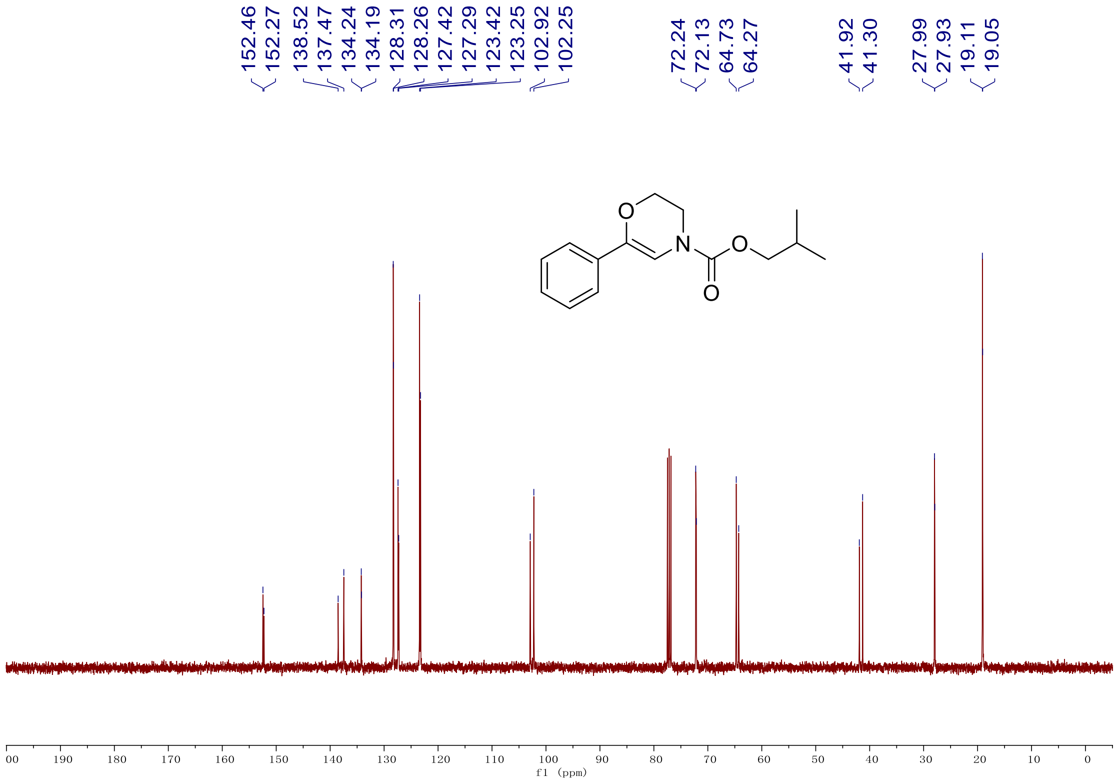

***tert*-Butyl 6-phenyl-2,3-dihydro-4*H*-1,4-oxazine-4-carboxylate (1a-Boc)**

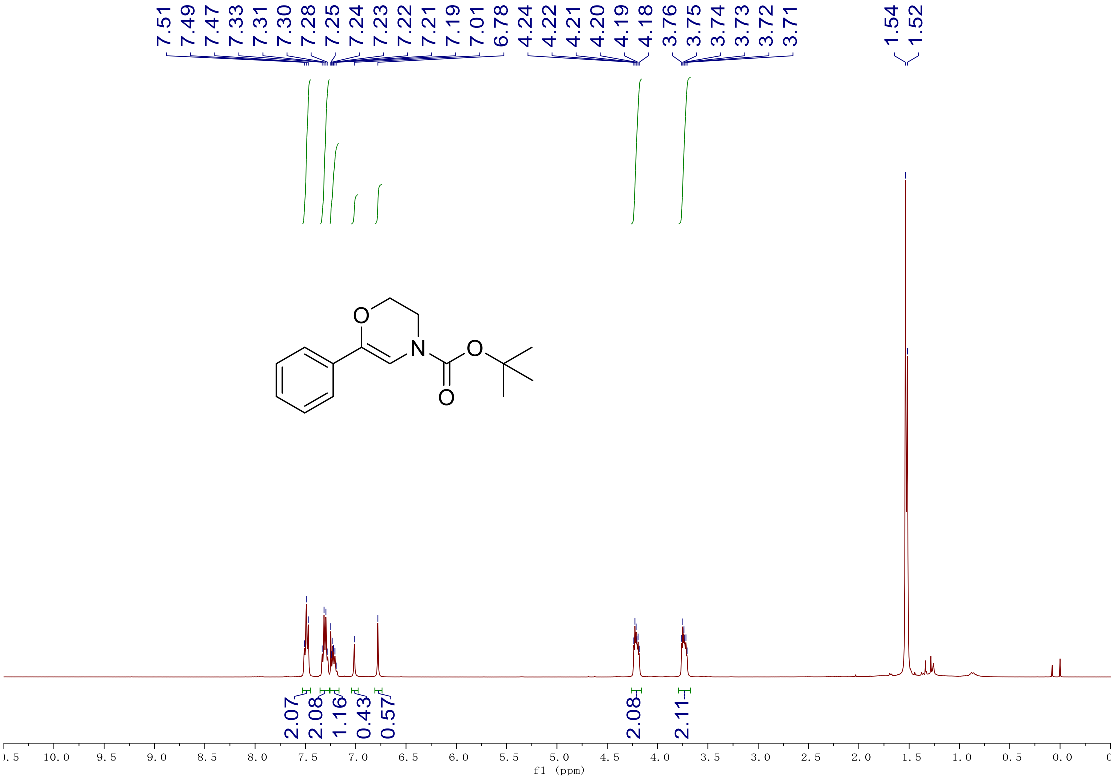

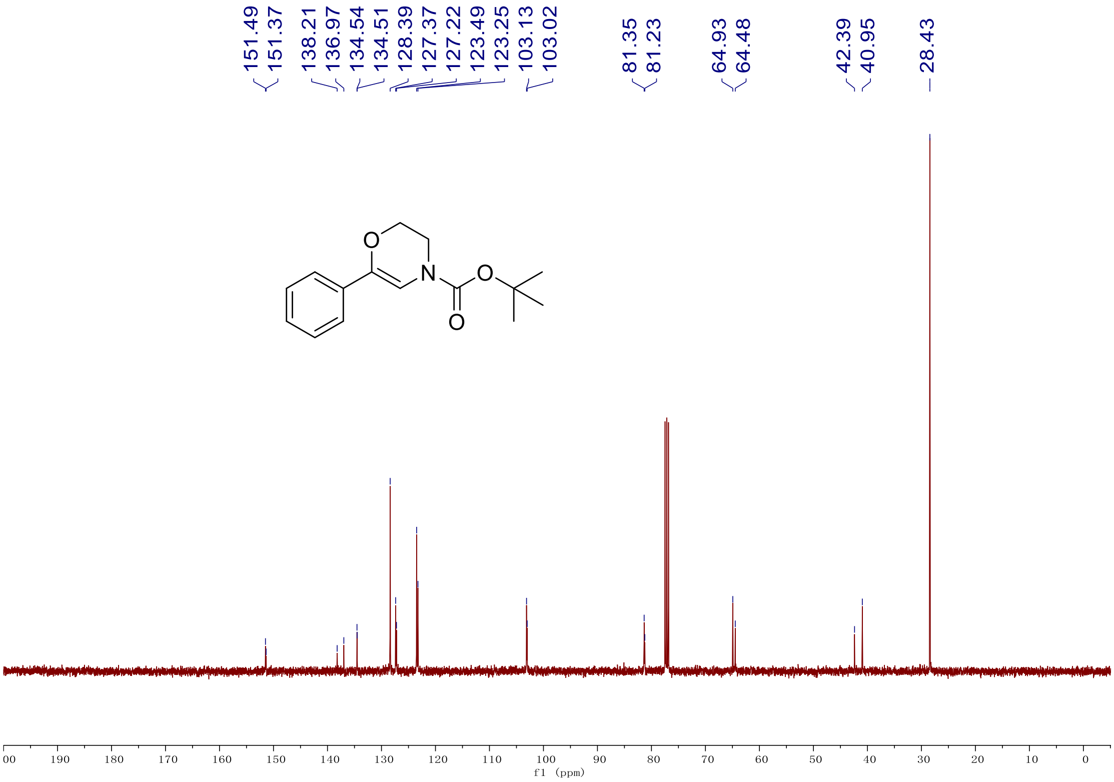

4-Phenyl-4-tosyl-3,4-dihydro-2*H*-1,4-oxazine (1a-Ts)

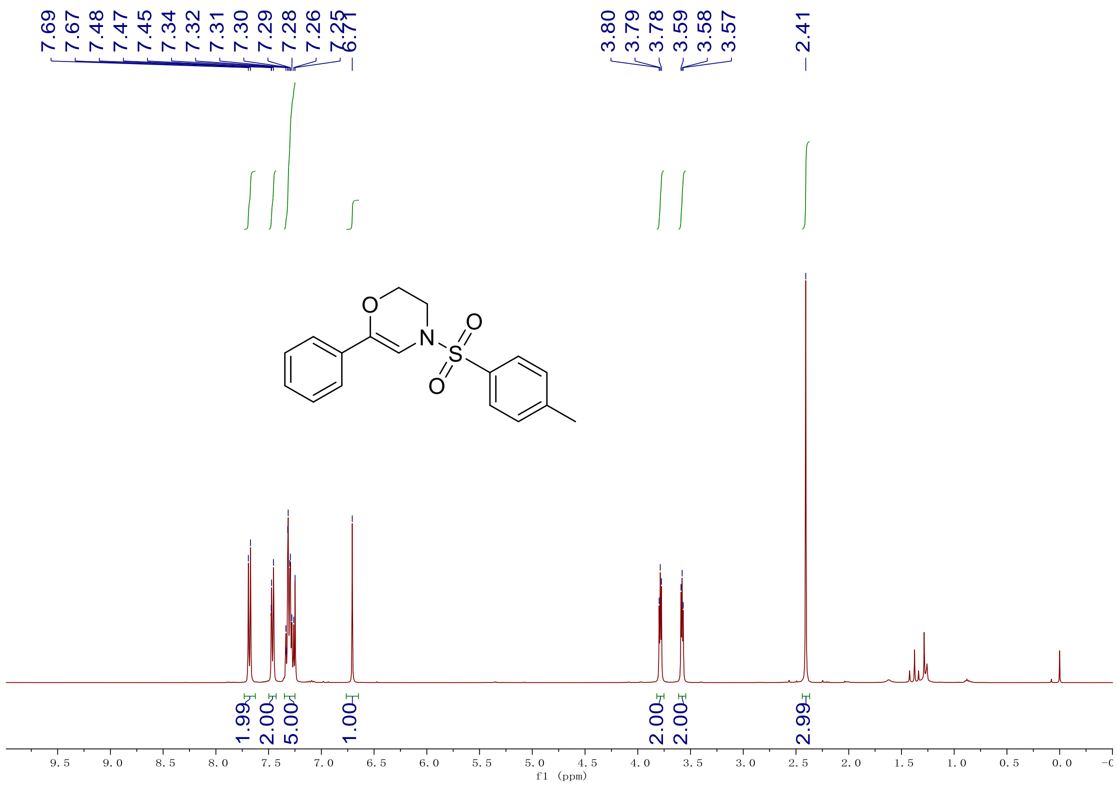

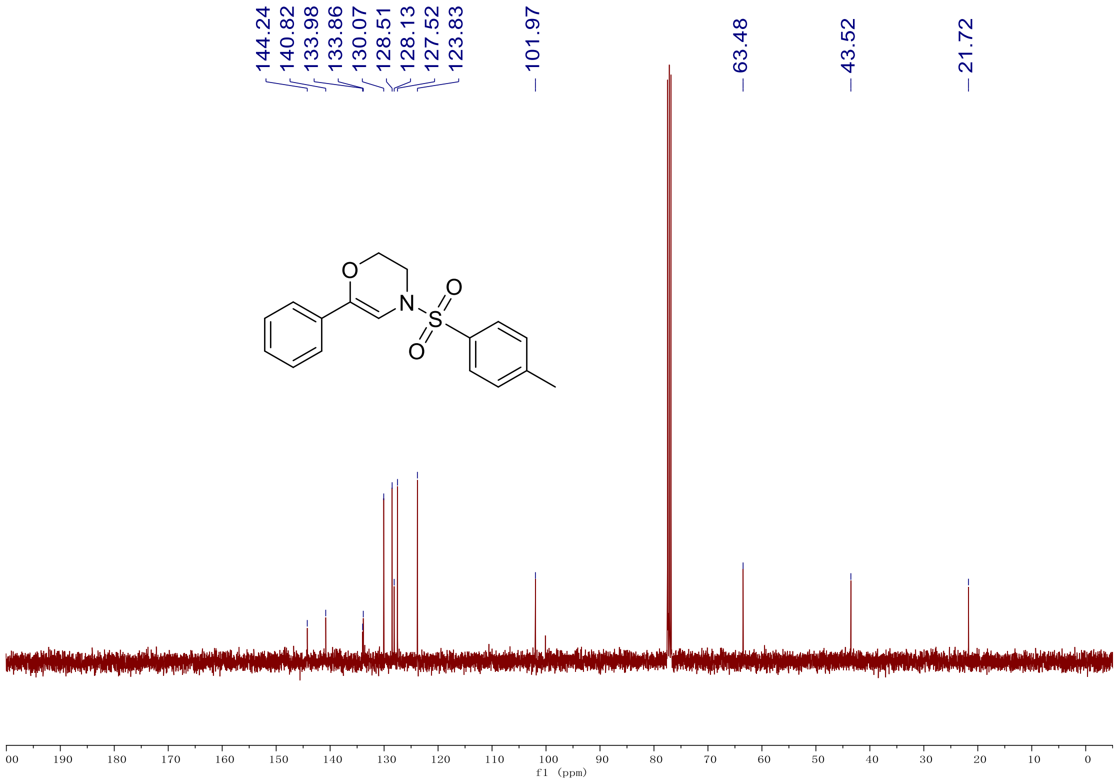

Benzyl (*R*)-2-phenylmorpholine-4-carboxylate (2a)

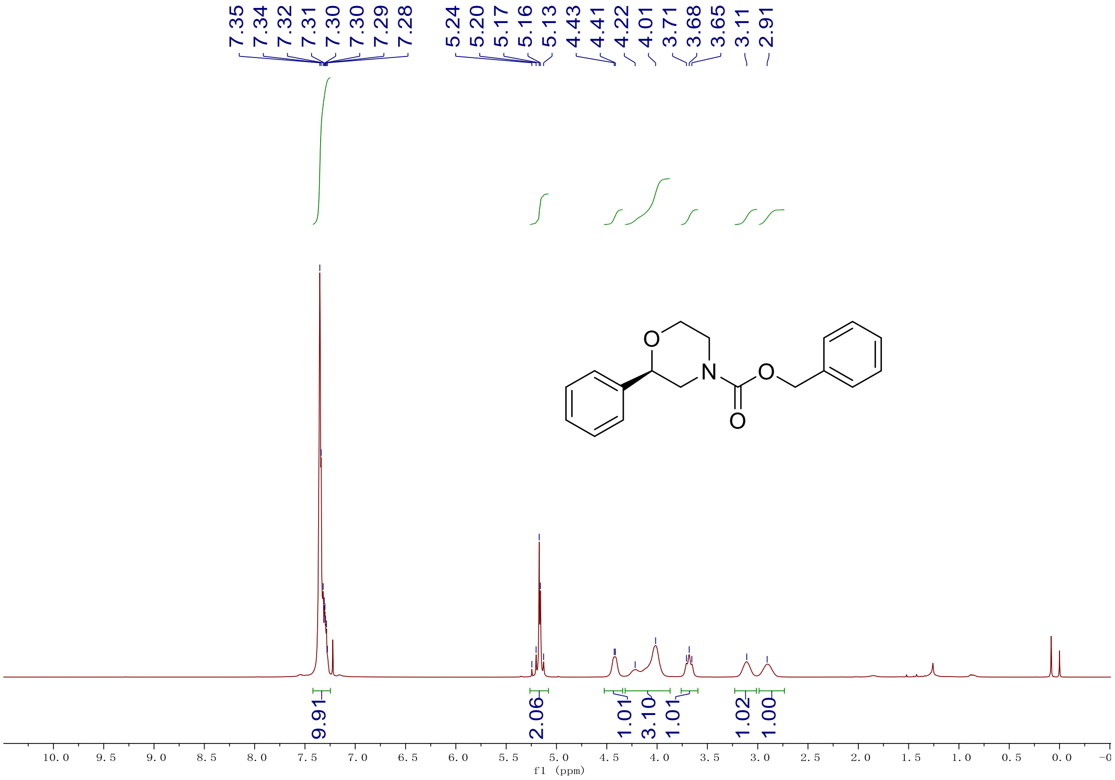

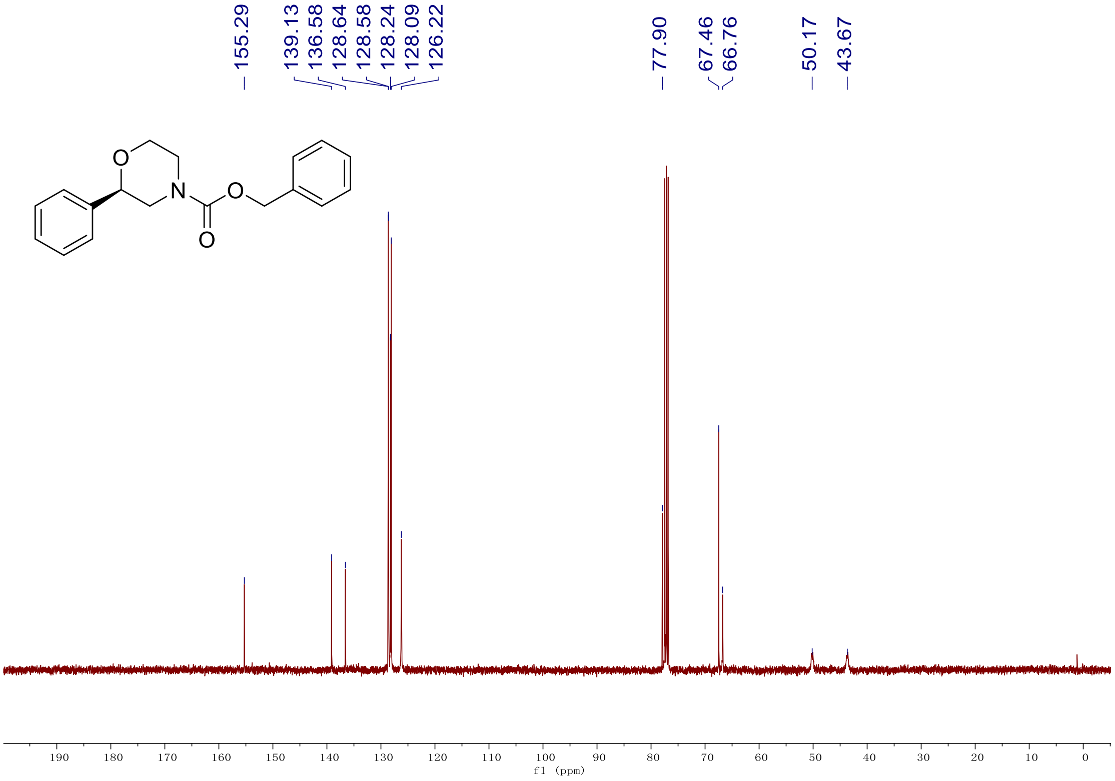

Benzyl (*R*)-2-(4-fluorophenyl)morpholine-4-carboxylate (2b)

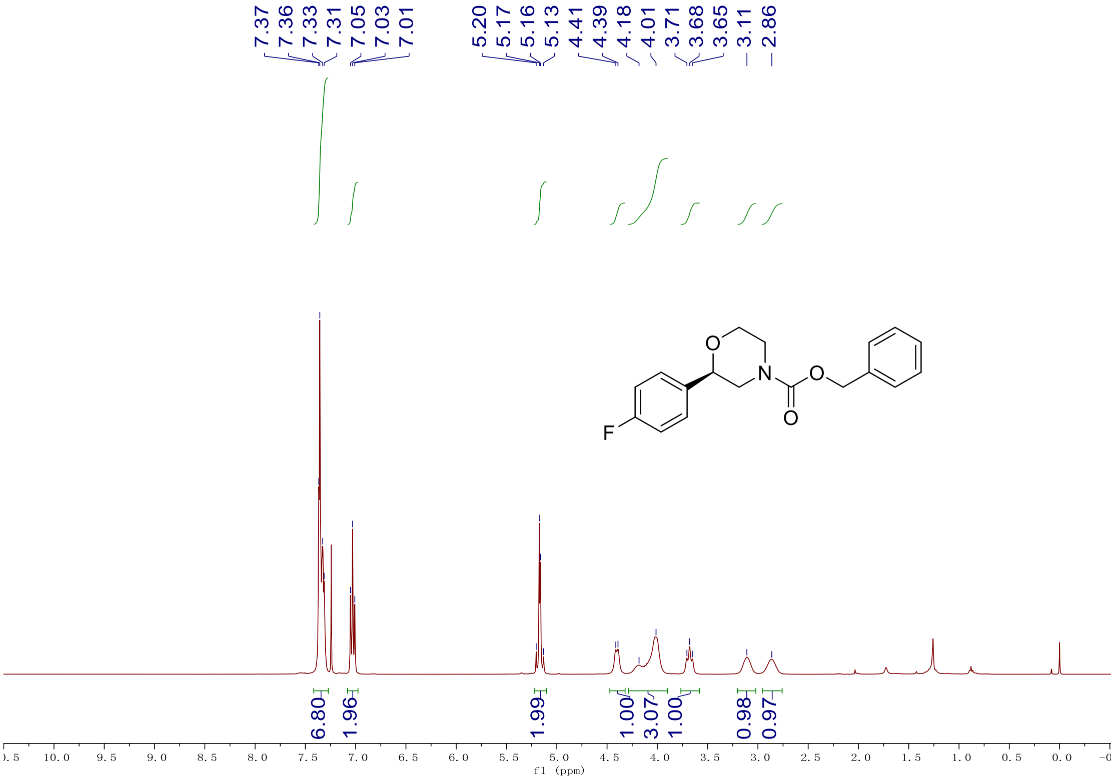

Diffusion ordered spectroscopy (DOSY) of 2b

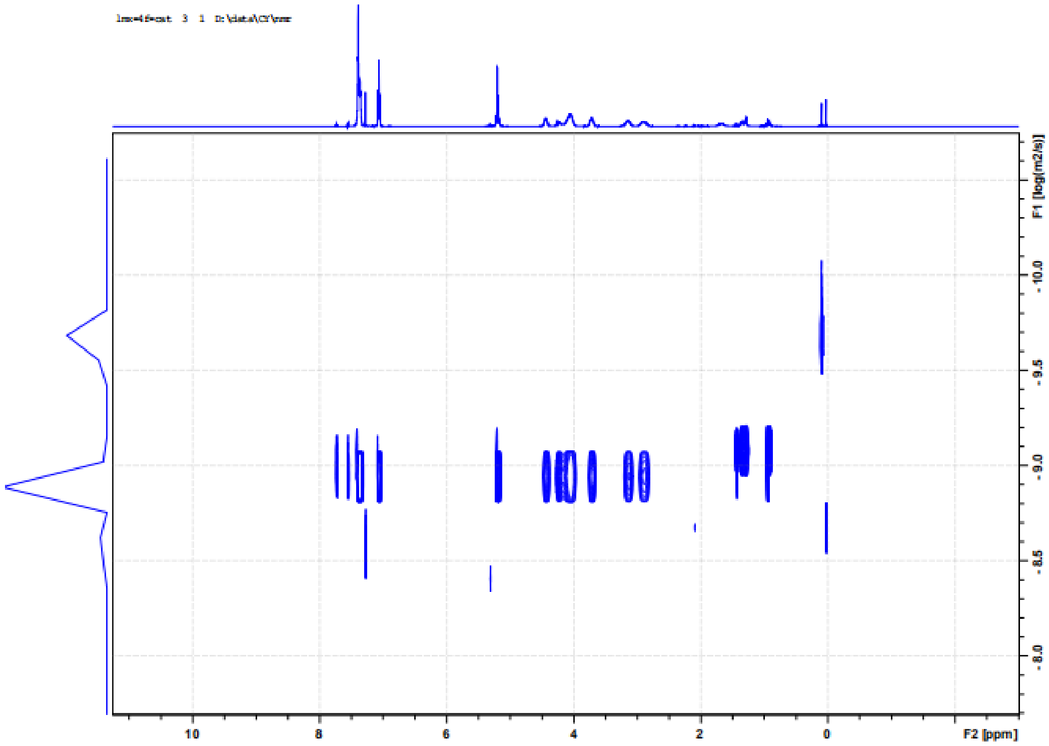

The unique diffusion coefficient indicates that the two sets of NMR data are caused by conformational isomerism.

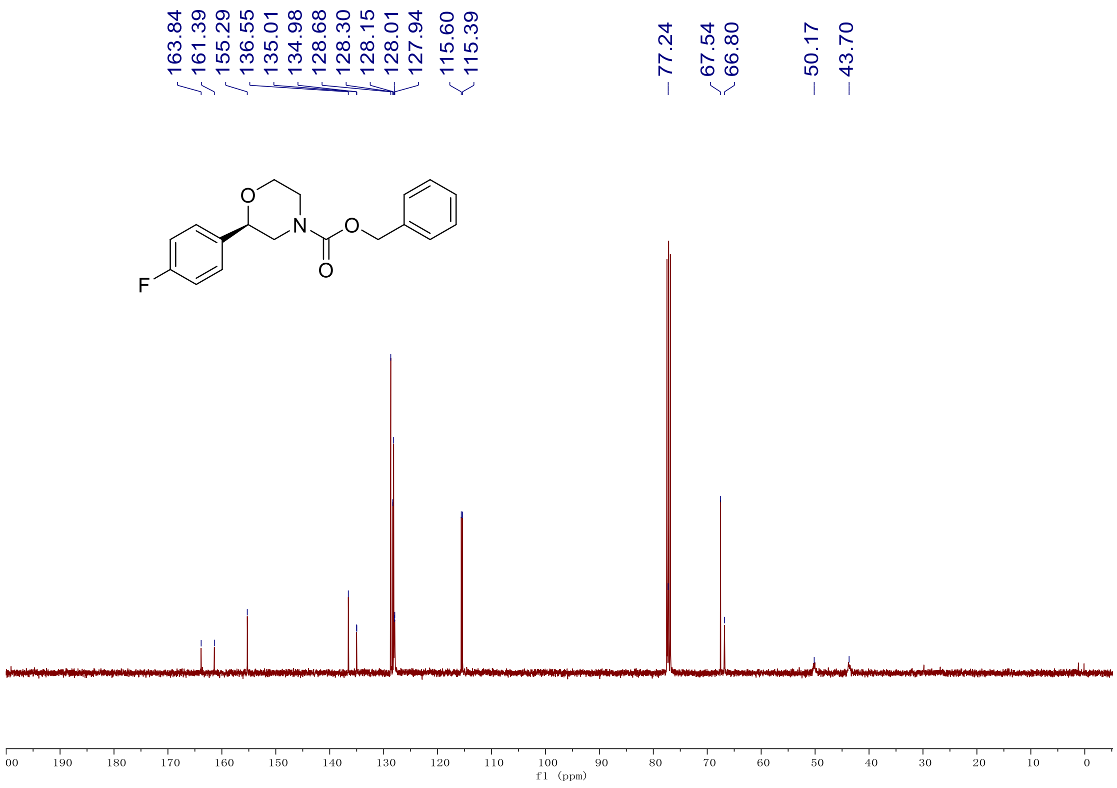
163.84
161.39
155.29
136.55
135.01
134.98
128.68
128.30
128.15
128.01
127.94
115.60
115.39
77.24
67.54
66.80
50.17
43.70
F
O
N
O
O
f1 (ppm)

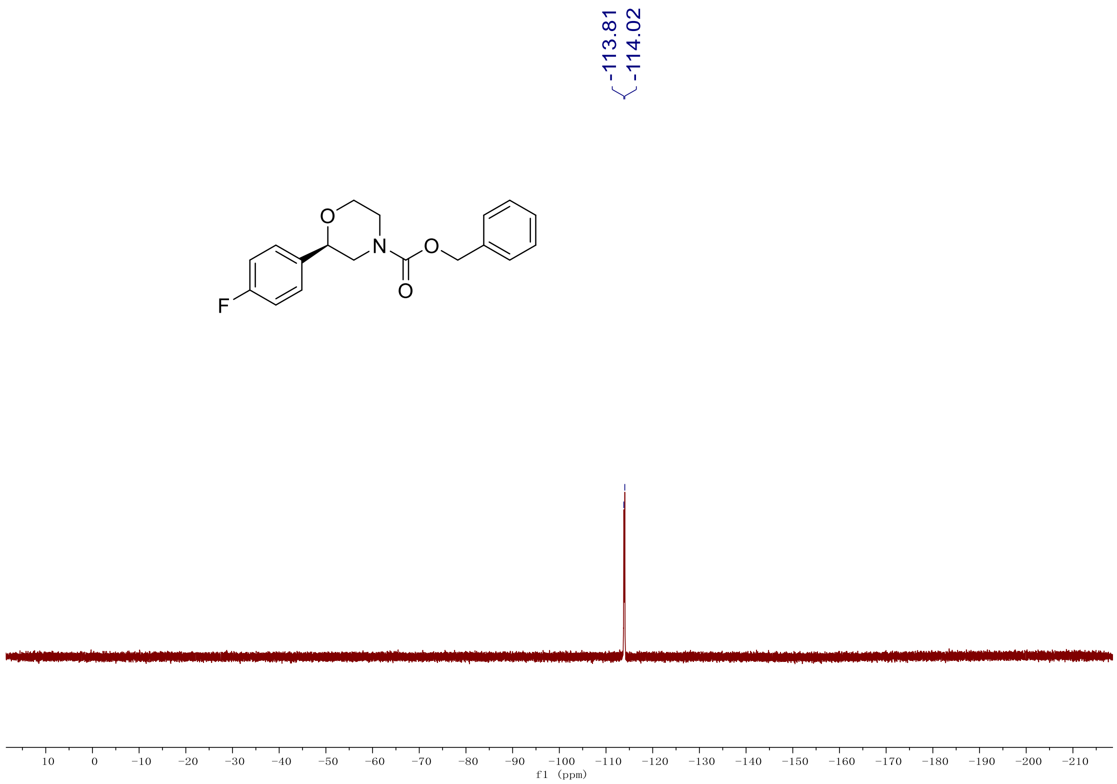
-113.81
-114.02
F
O
N
O
O
f1 (ppm)

Benzyl (*R*)-2-(4-chlorophenyl)morpholine-4-carboxylate (2c)

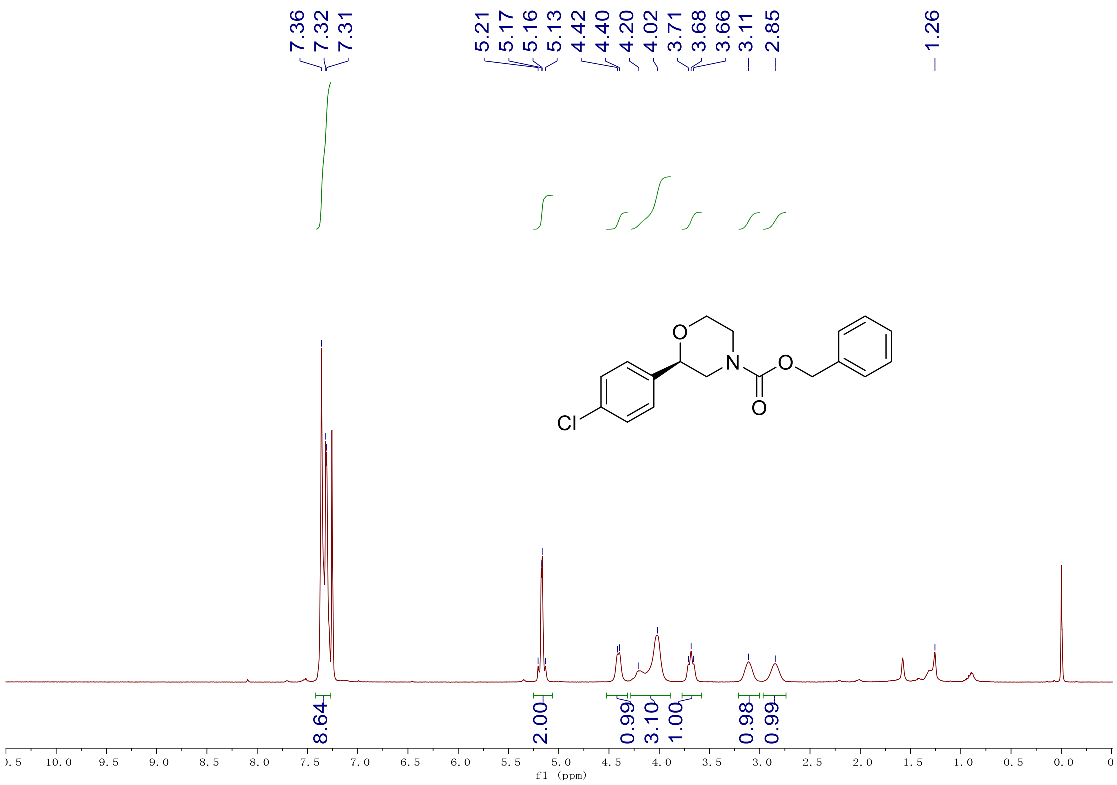

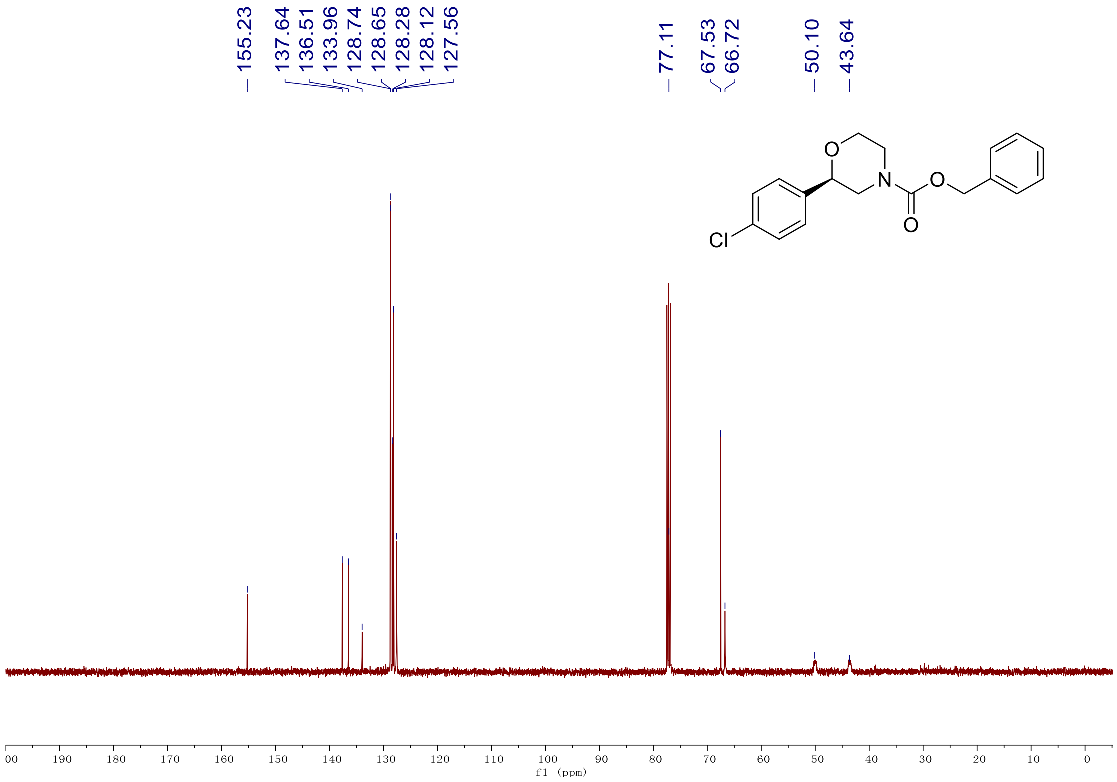

Benzyl (*R*)-2-(4-bromophenyl)morpholine-4-carboxylate (2d)

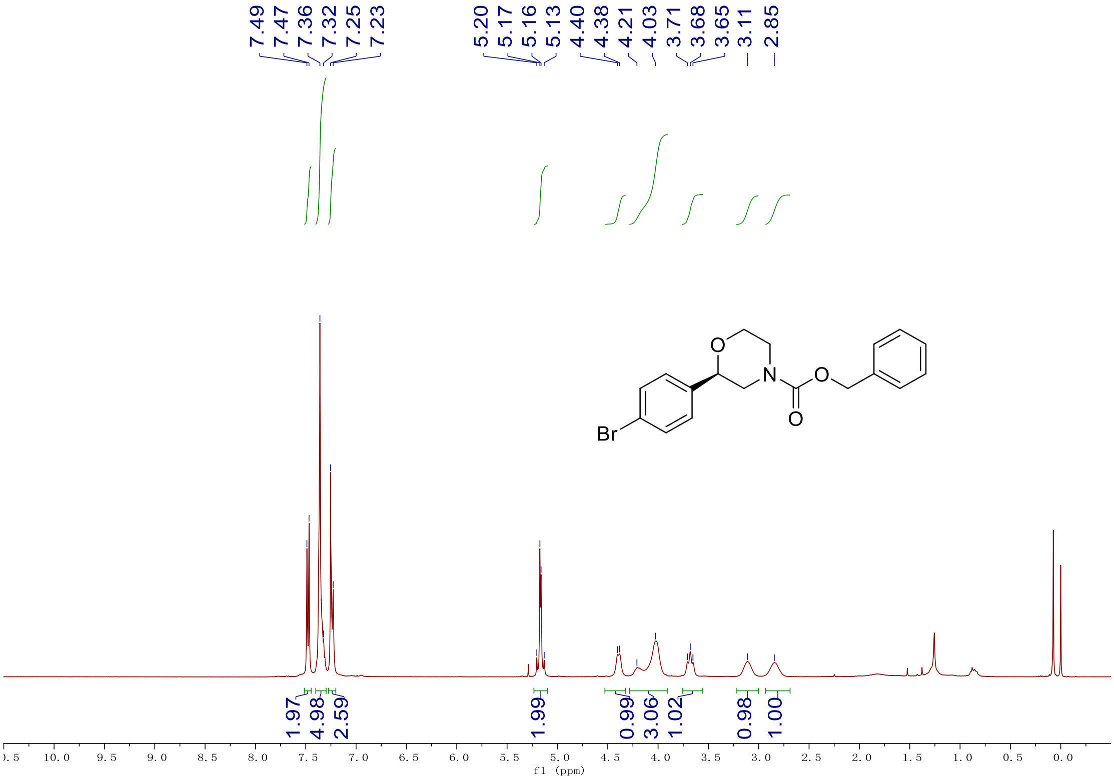

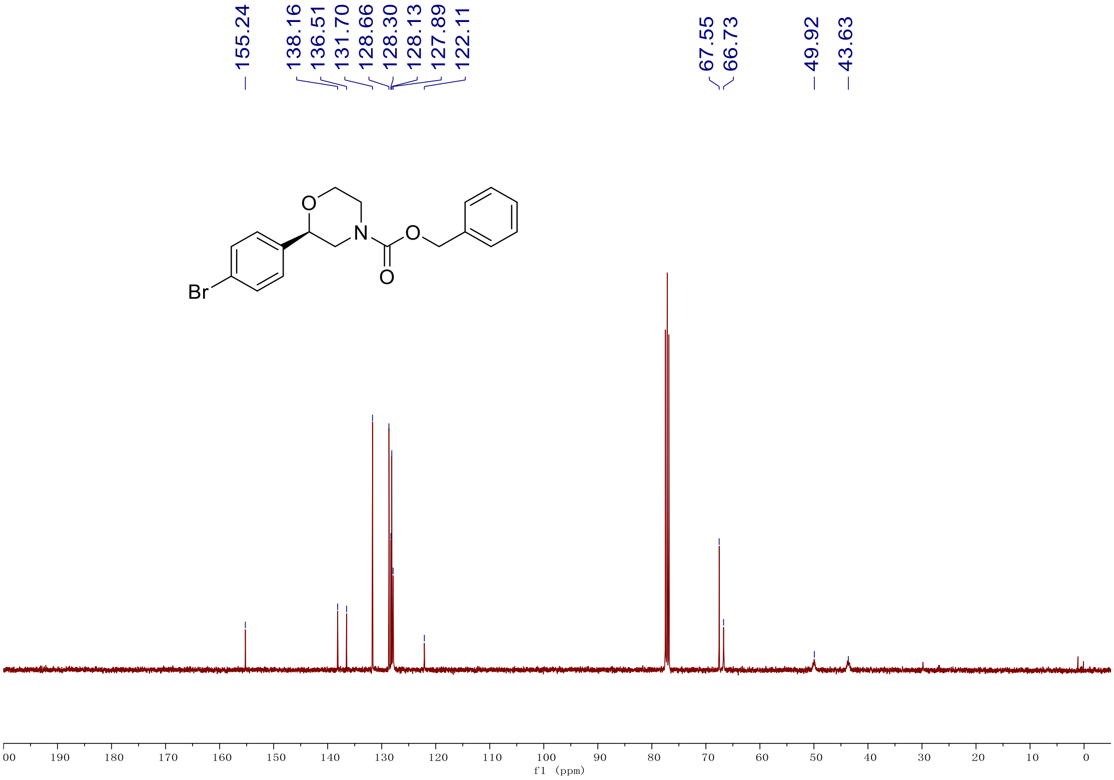

Benzyl (*R*)-2-([1,1'-biphenyl]-4-yl)morpholine-4-carboxylate (2e)

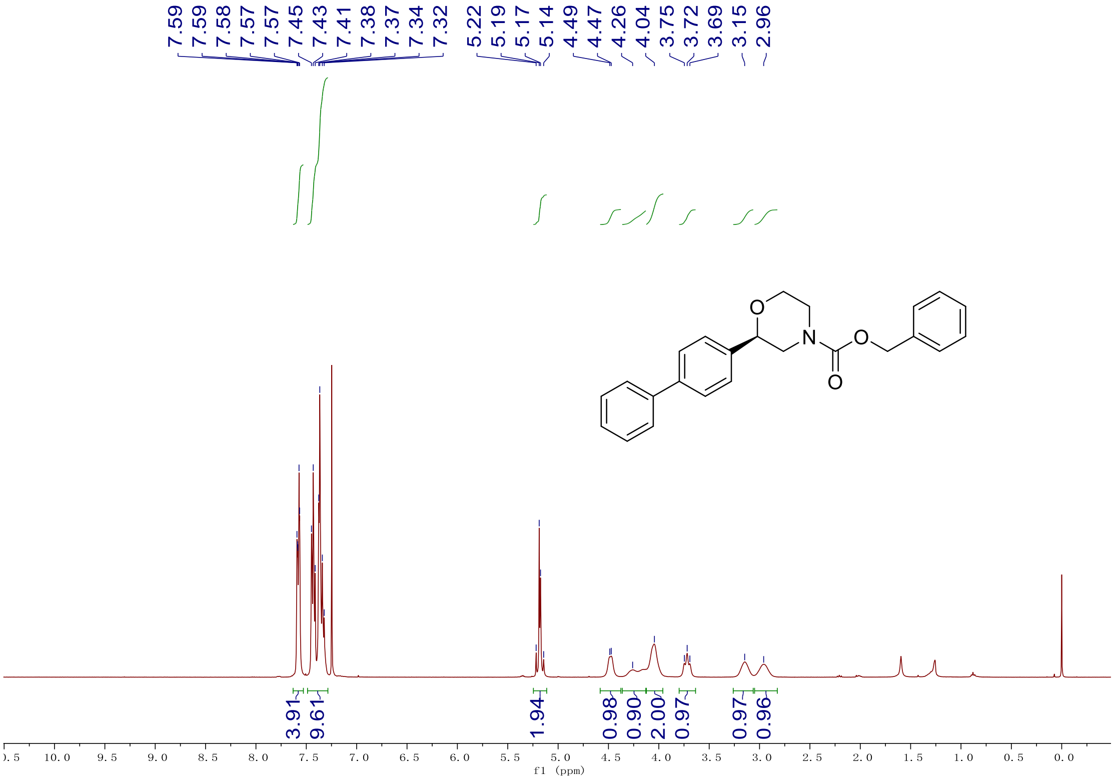

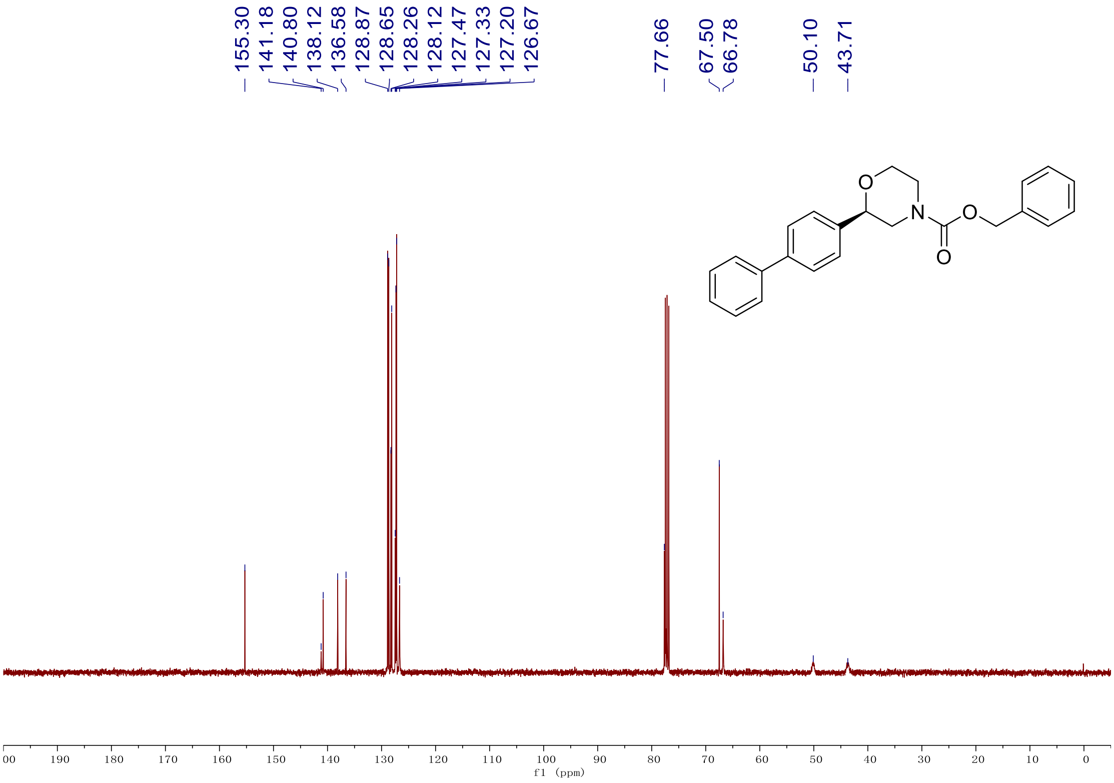

Benzyl (*R*)-2-(4-(trifluoromethyl)phenyl)morpholine-4-carboxylate (2f)

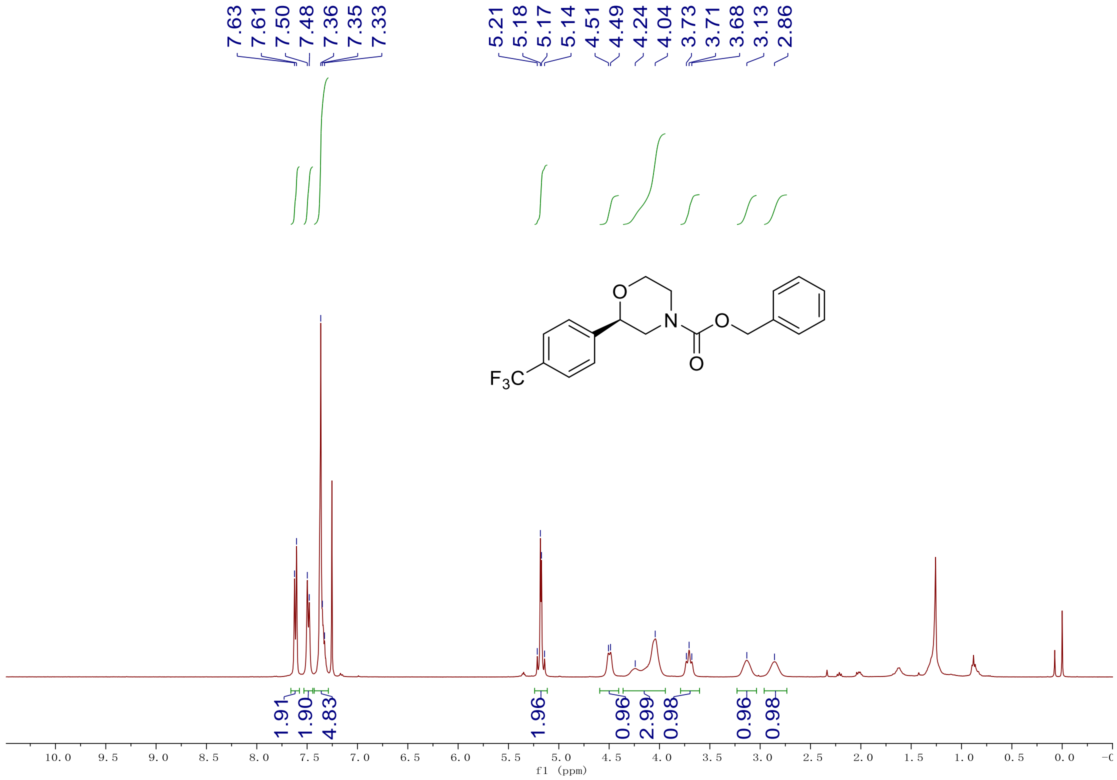

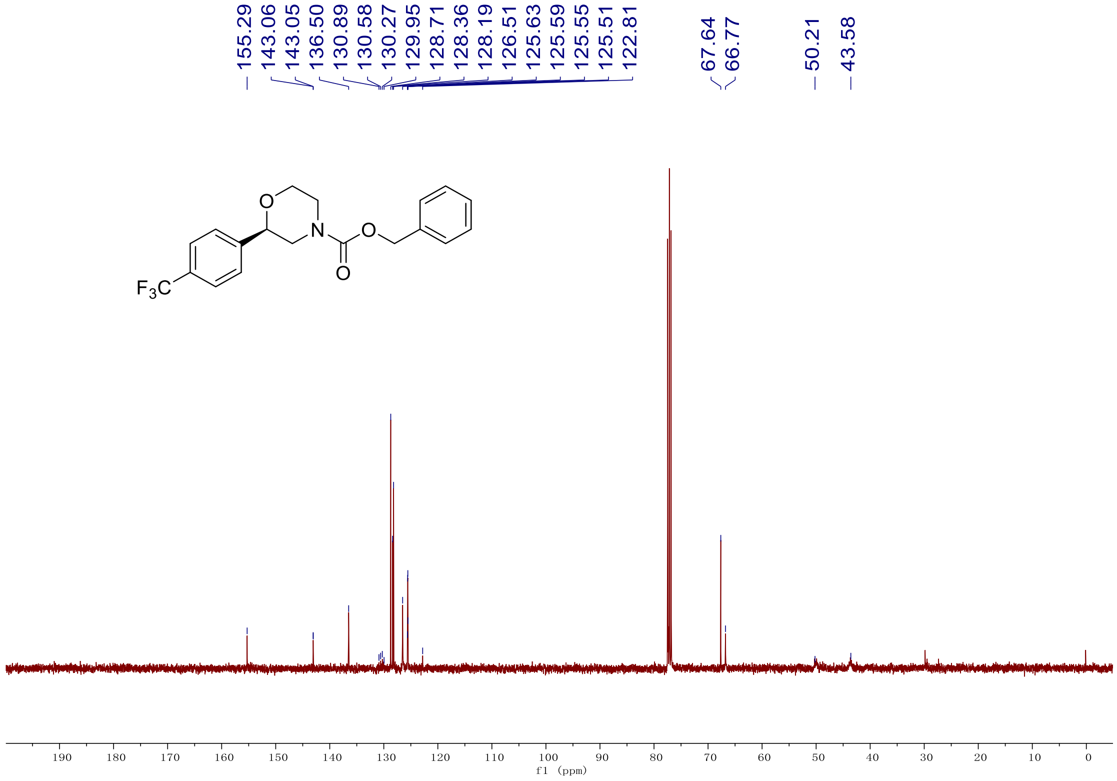

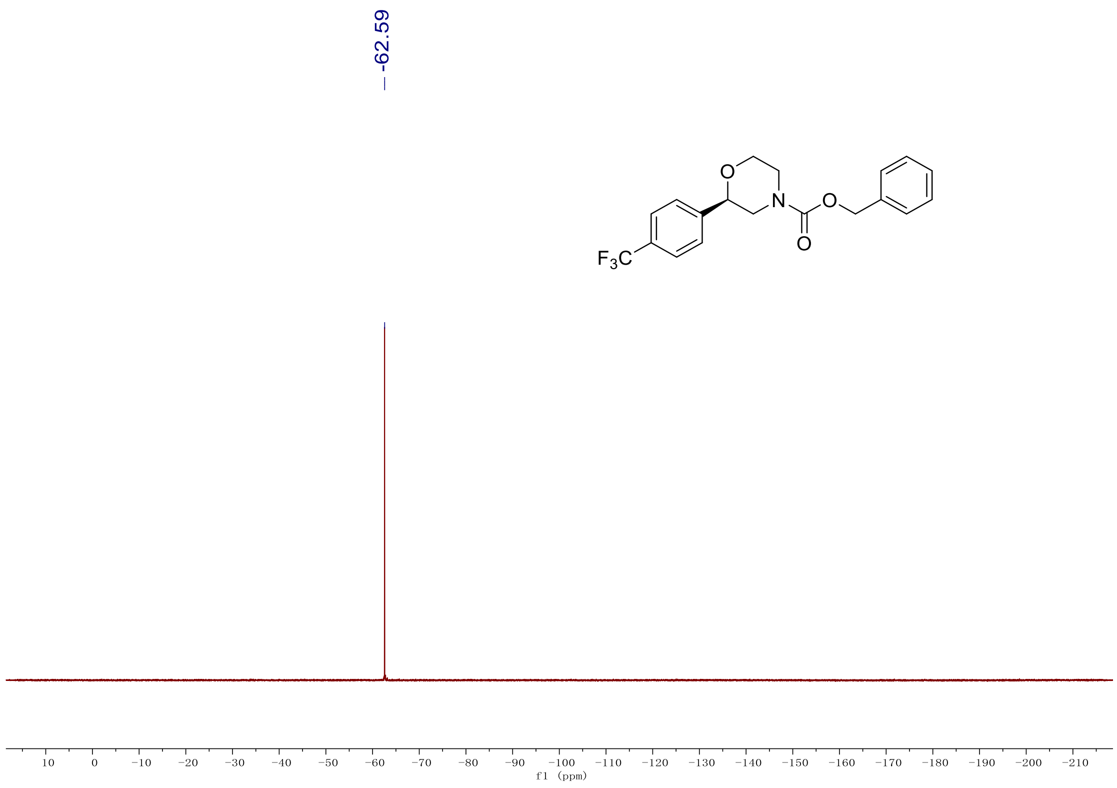

-62.59
F3C
O
N
O
O
10
0
-10
-20
-30
-40
-50
-60
-70
-80
-90
-100
-110
-120
-130
-140
-150
-160
-170
-180
-190
-200
-210
f1 (ppm)

Benzyl (*R*)-2-(*p*-tolyl)morpholine-4-carboxylate (2g)

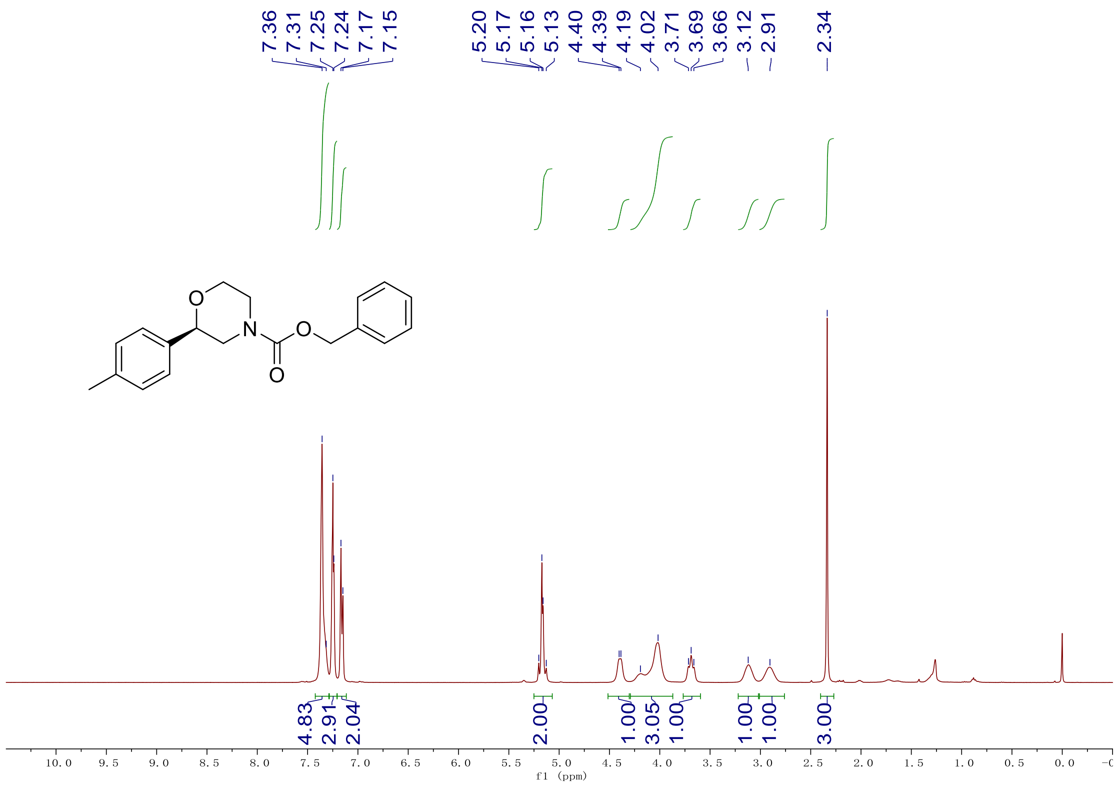

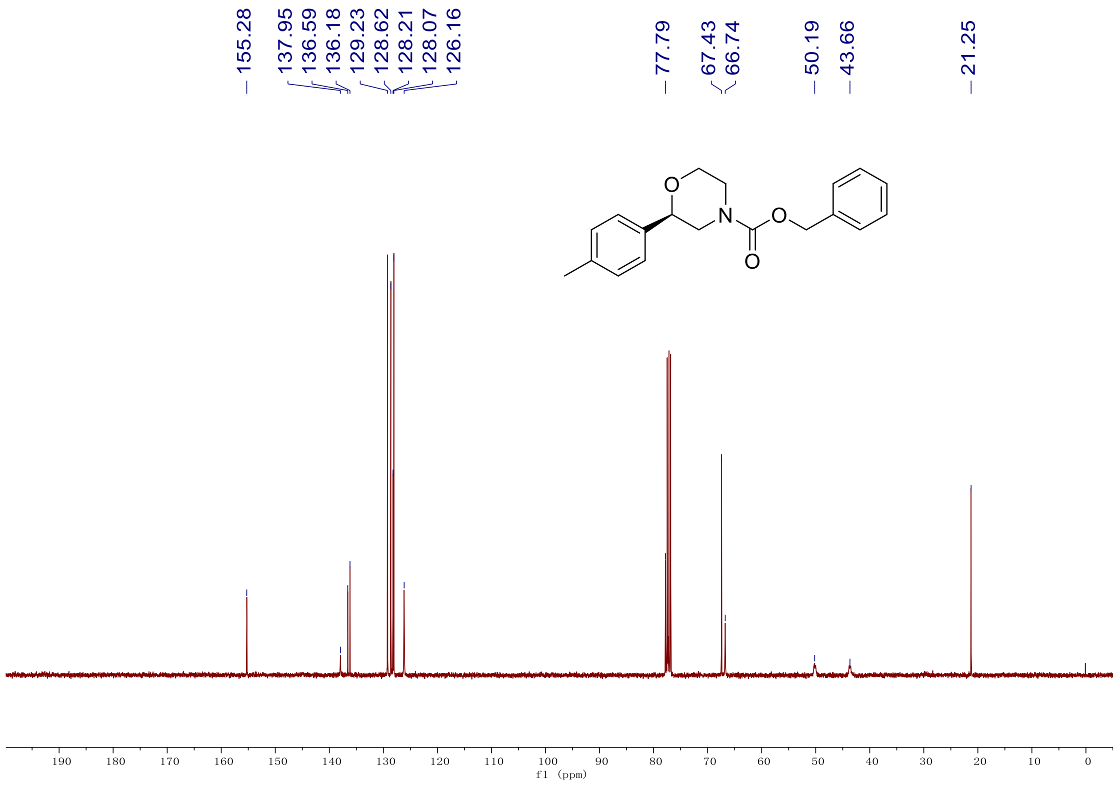

Benzyl (*R*)-2-(4-methoxyphenyl)morpholine-4-carboxylate (2h)

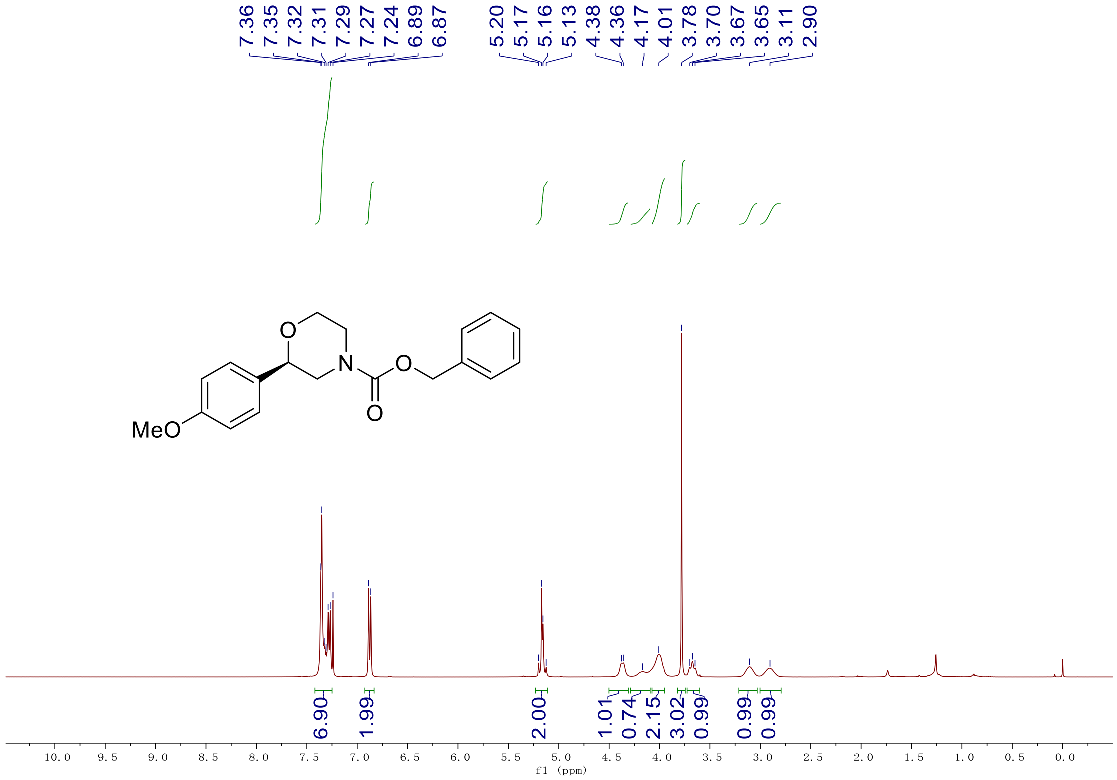

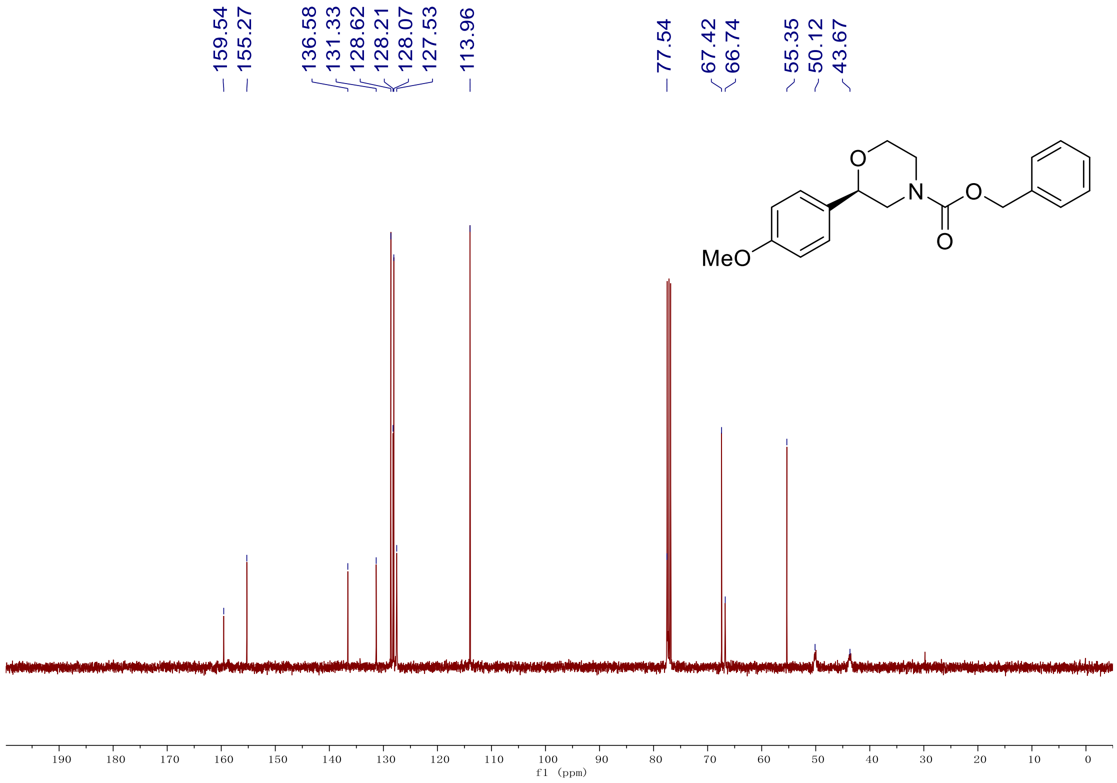

Benzyl (*R*)-2-(3-fluorophenyl)morpholine-4-carboxylate (2i)

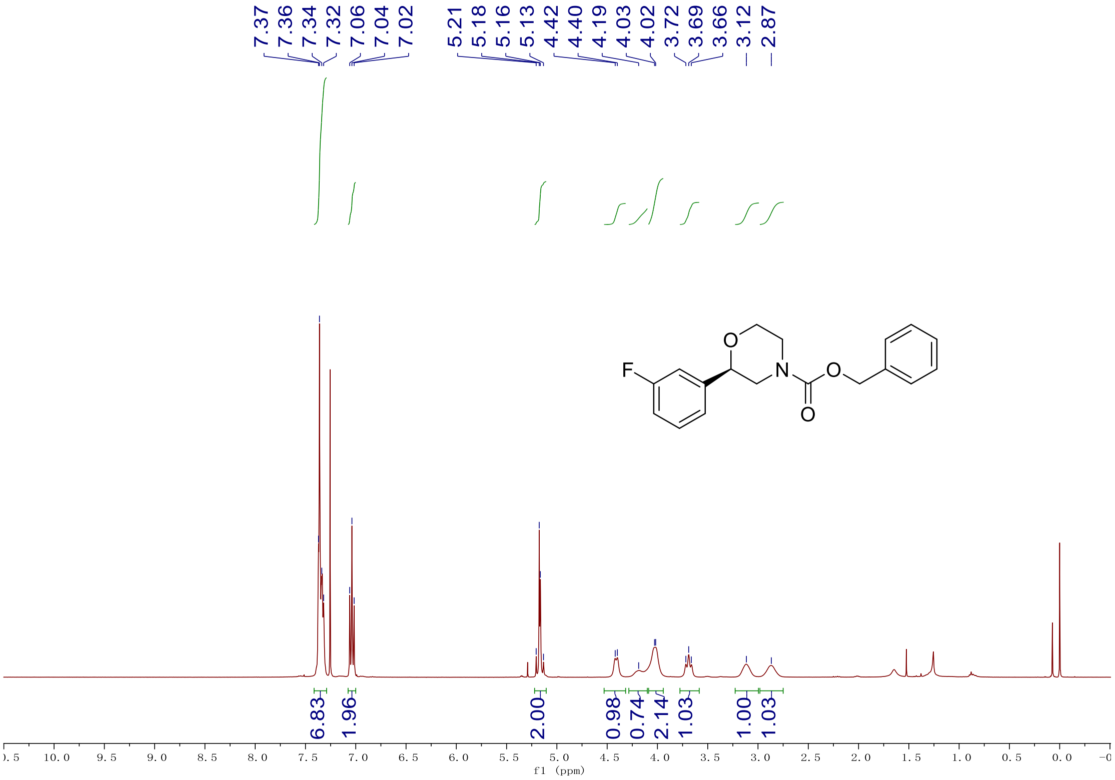

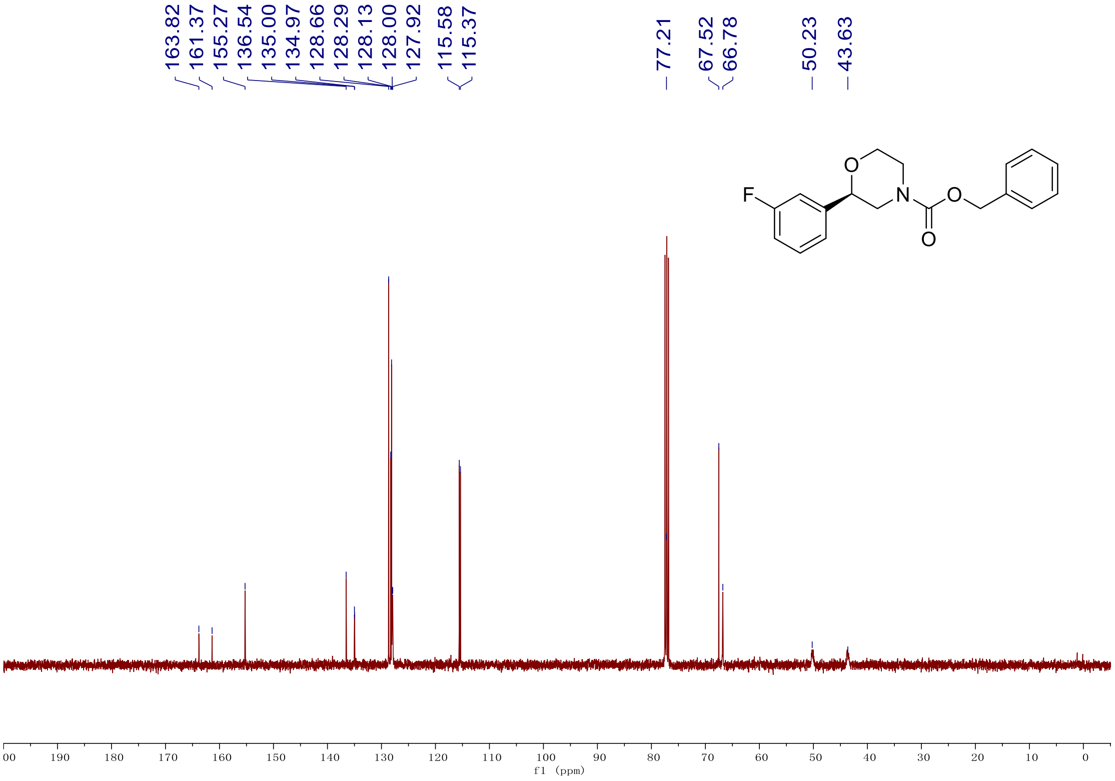

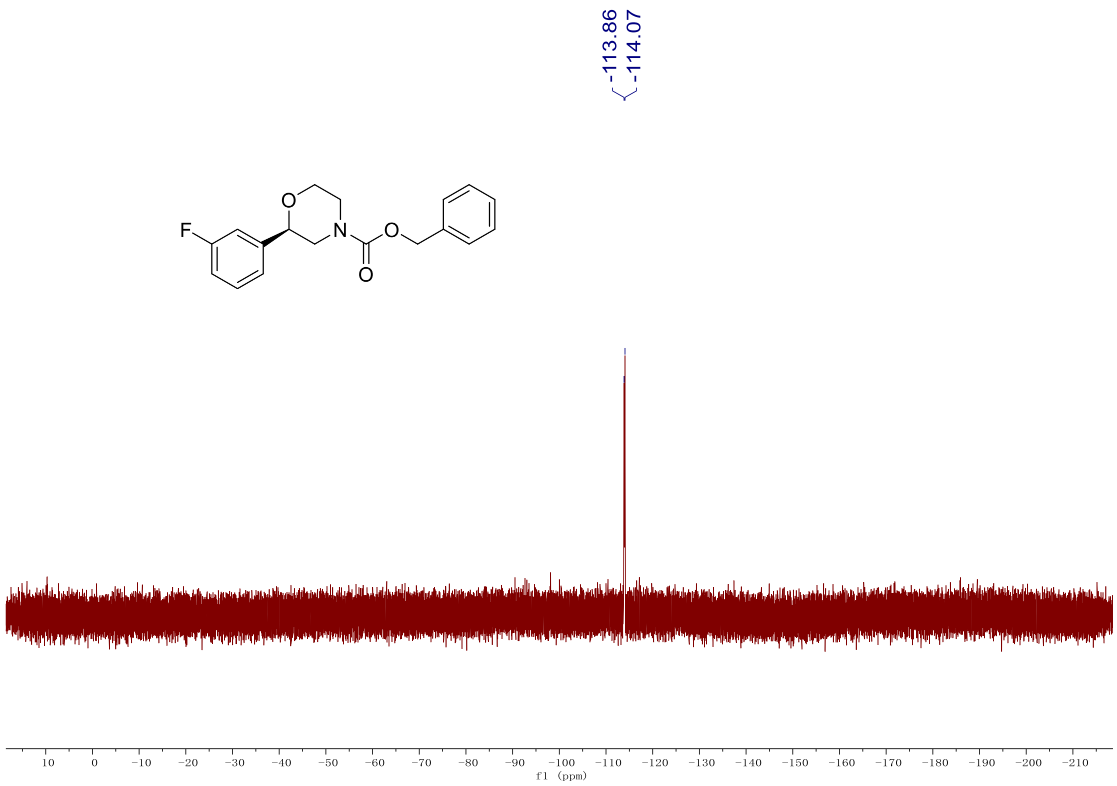

-113.86
-114.07
F
O
N
O
O
10 0 −10 −20 −30 −40 −50 −60 −70 −80 −90 −100 −110 −120 −130 −140 −150 −160 −170 −180 −190 −200 −210
f1 (ppm)

Benzyl (*R*)-2-(3-chlorophenyl)morpholine-4-carboxylate (2j)

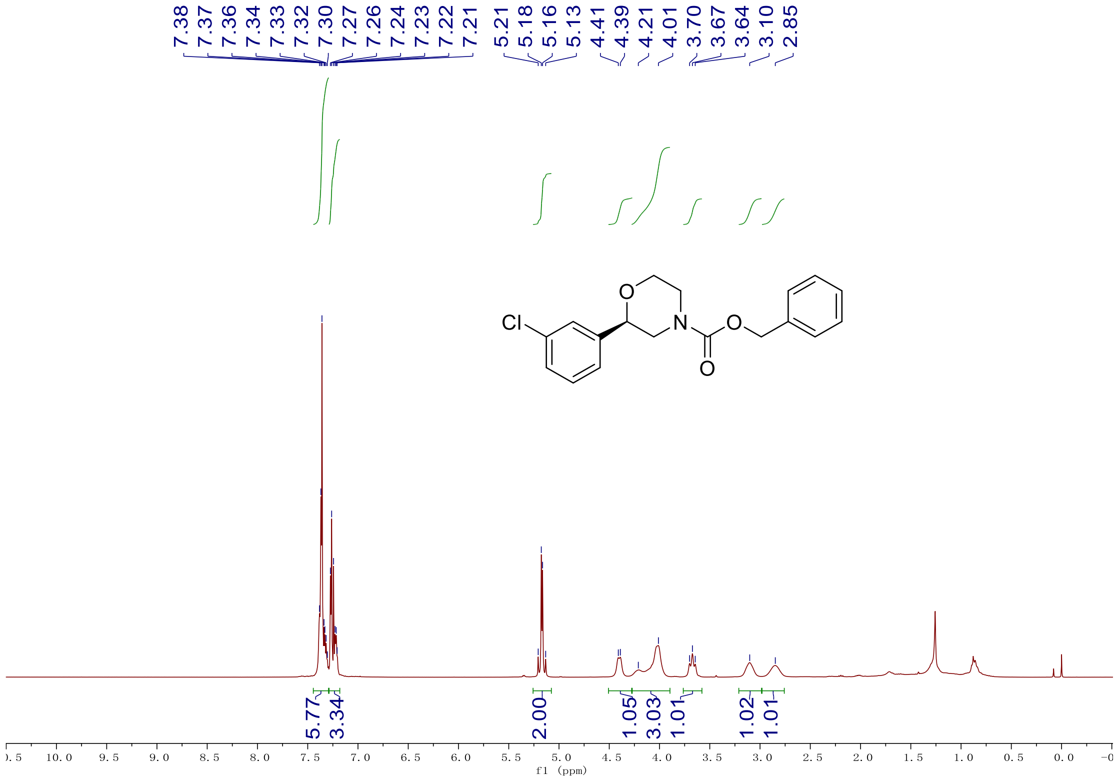

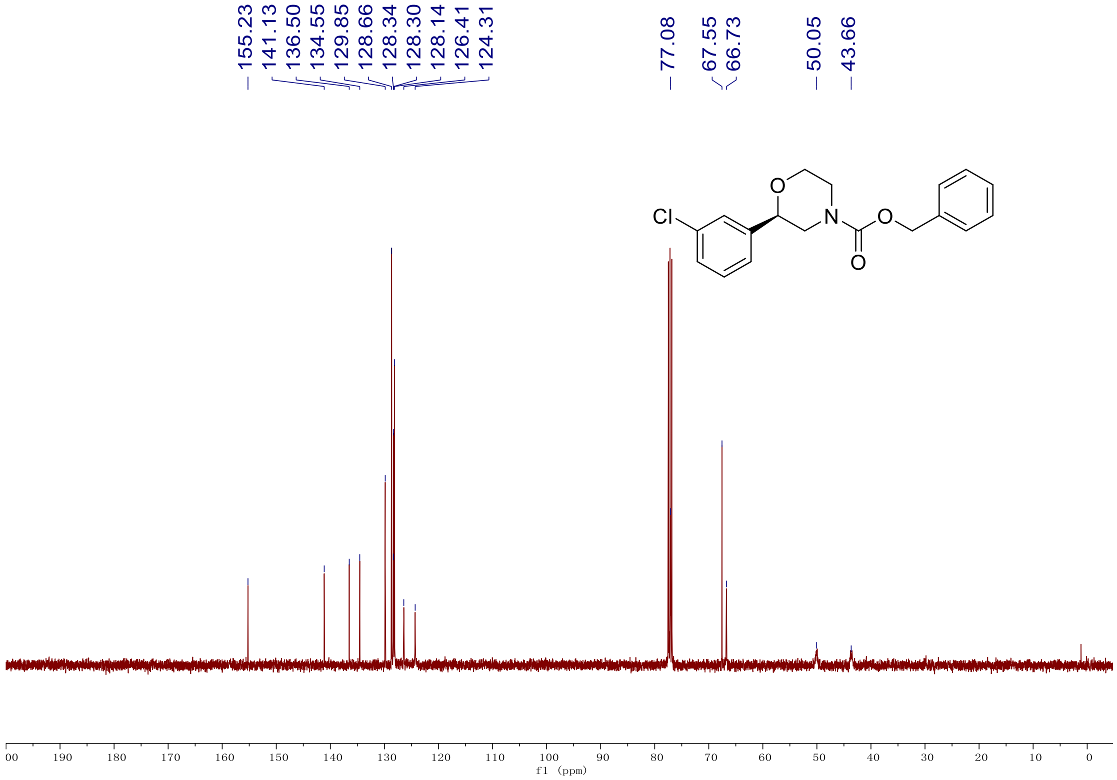

Benzyl (*R*)-2-(3-bromophenyl)morpholine-4-carboxylate (2k)

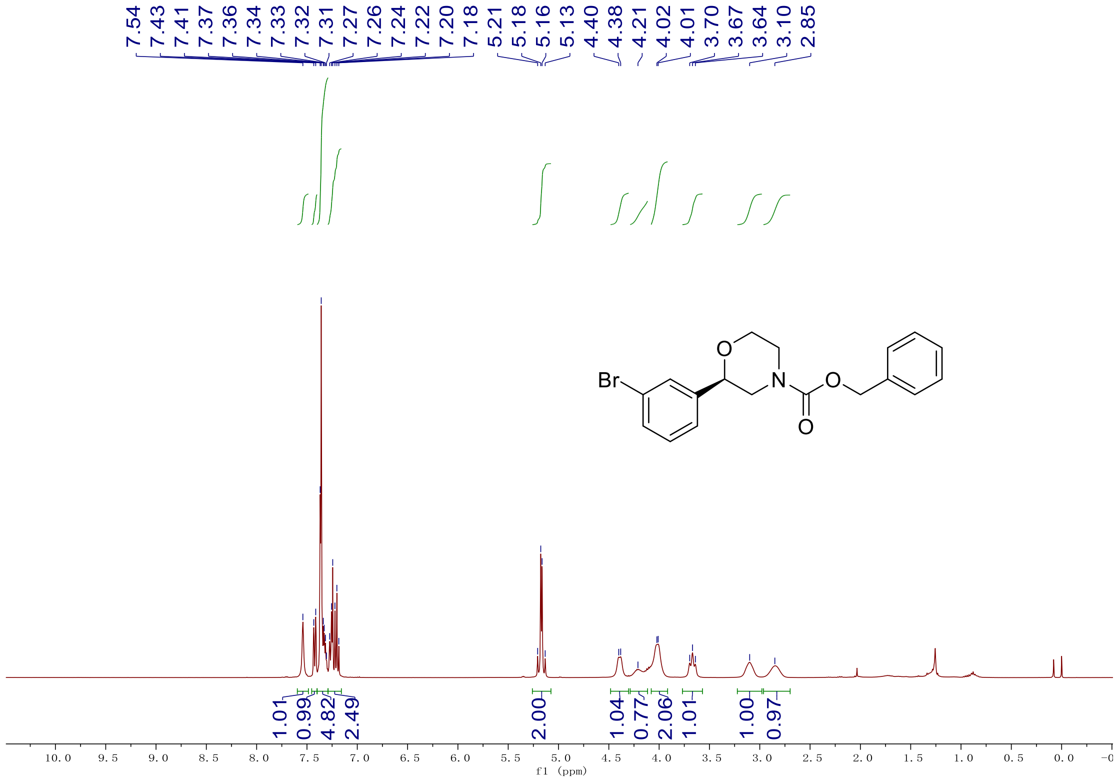

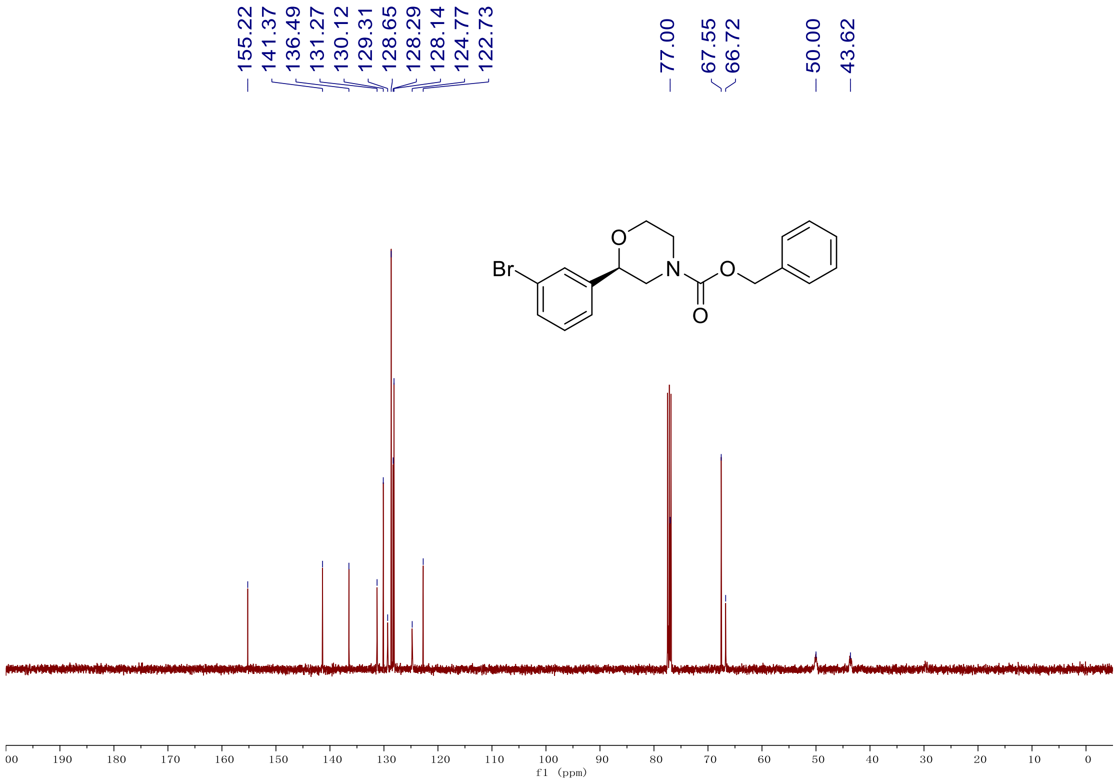

Benzyl (*R*)-2-(3-methoxyphenyl)morpholine-4-carboxylate (2l)

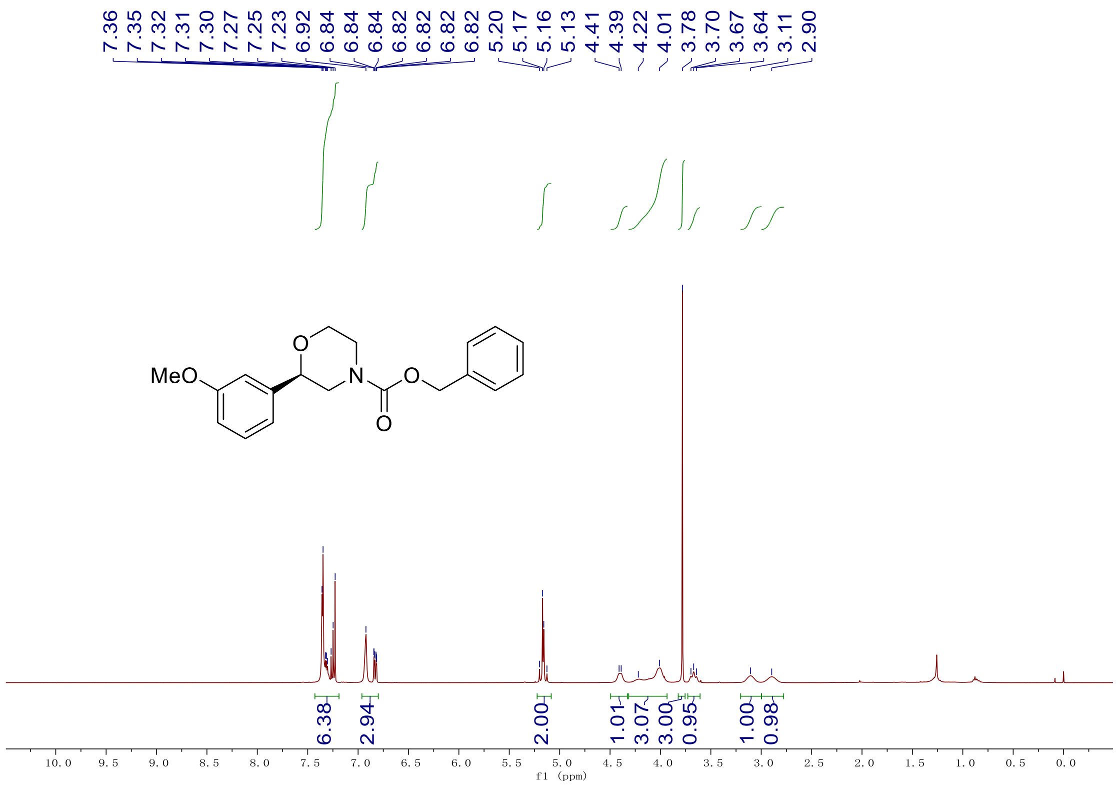

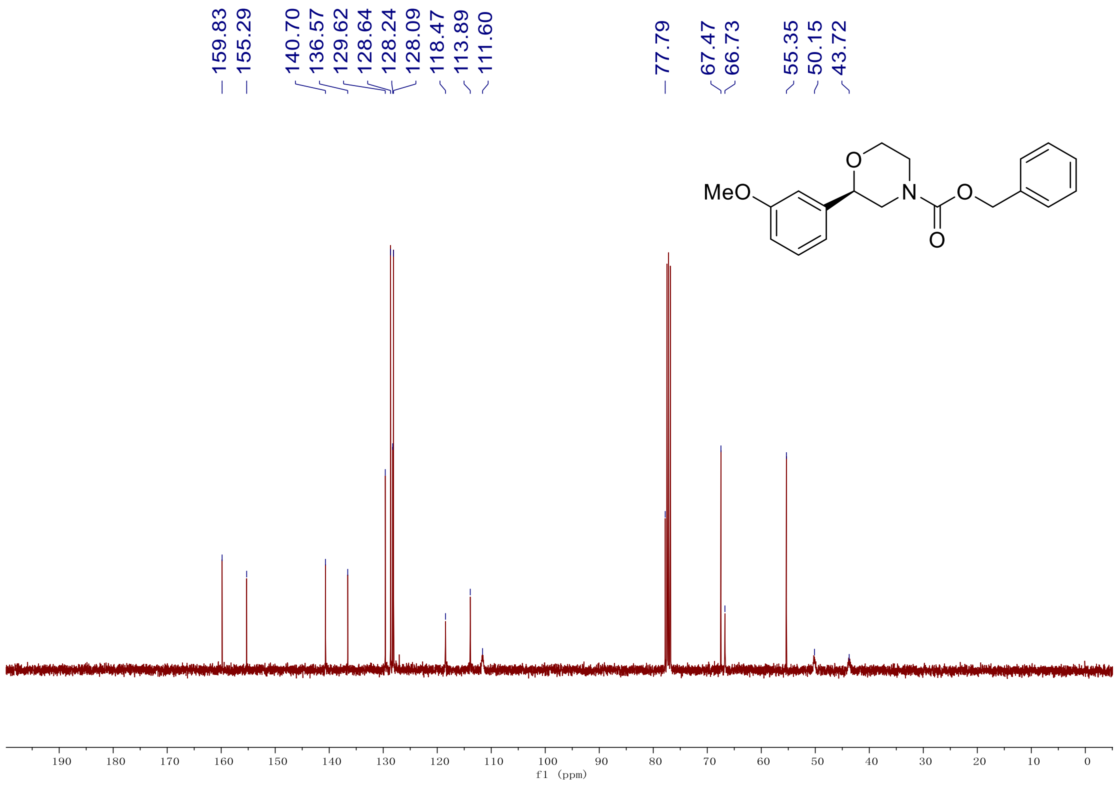

Benzyl (*R*)-2-(2-fluorophenyl)morpholine-4-carboxylate (2m)

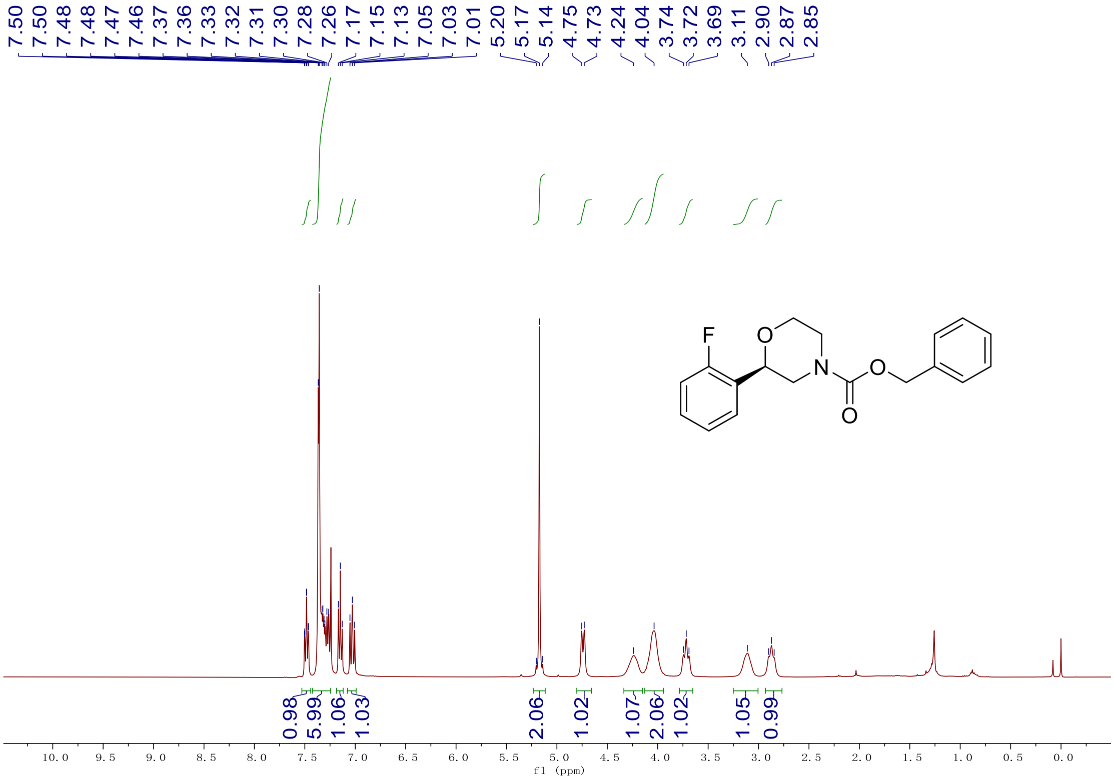

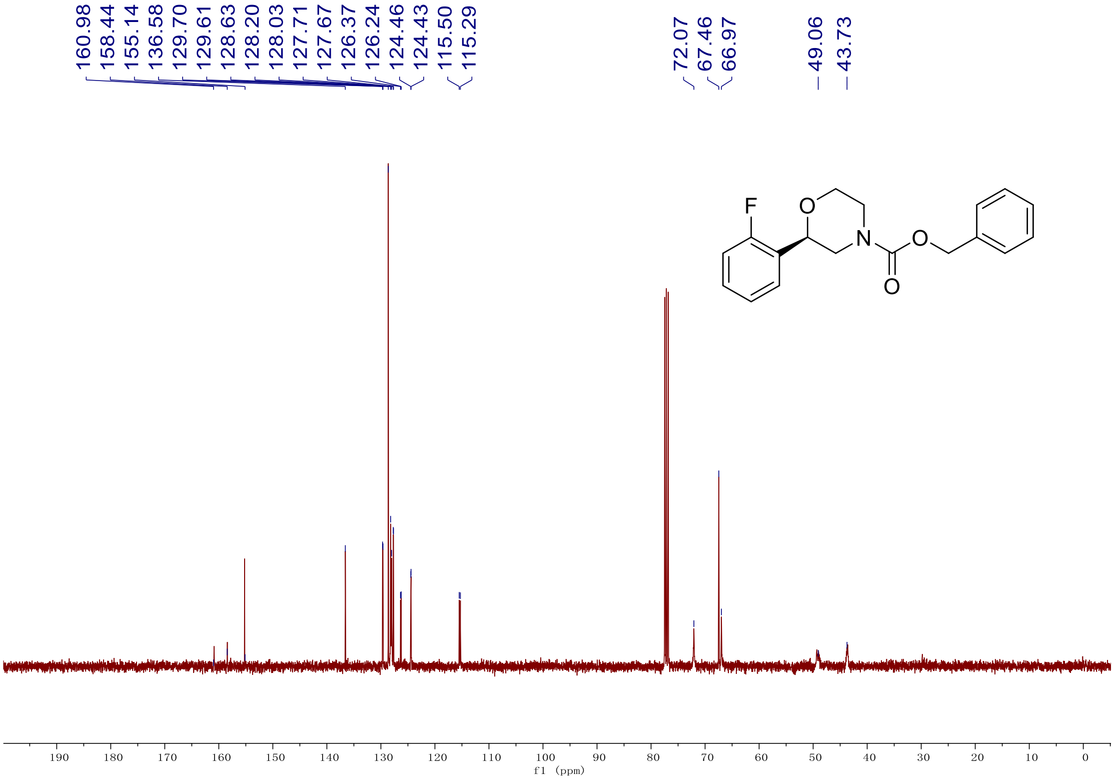

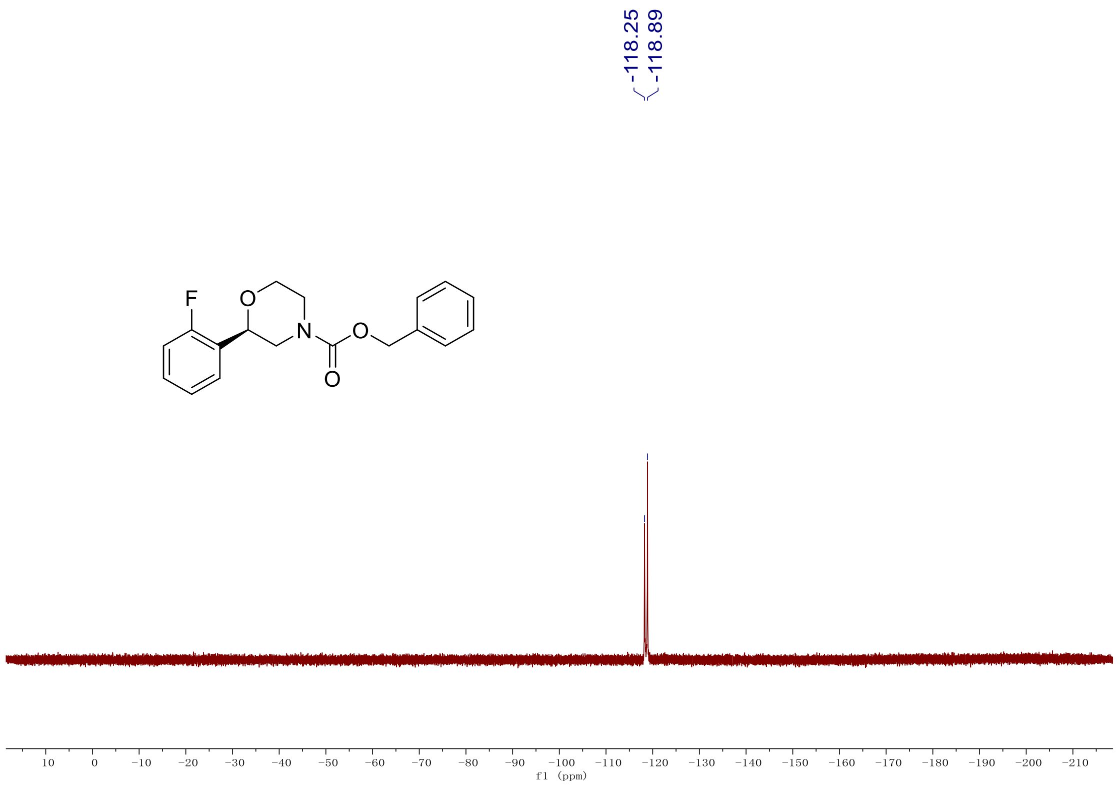
-118.25
-118.89
F
O
N
O
O
10 0 −10 −20 −30 −40 −50 −60 −70 −80 −90 −100 −110 −120 −130 −140 −150 −160 −170 −180 −190 −200 −210
f1 (ppm)

Benzyl (*R*)-2-(2-chlorophenyl)morpholine-4-carboxylate (2n)

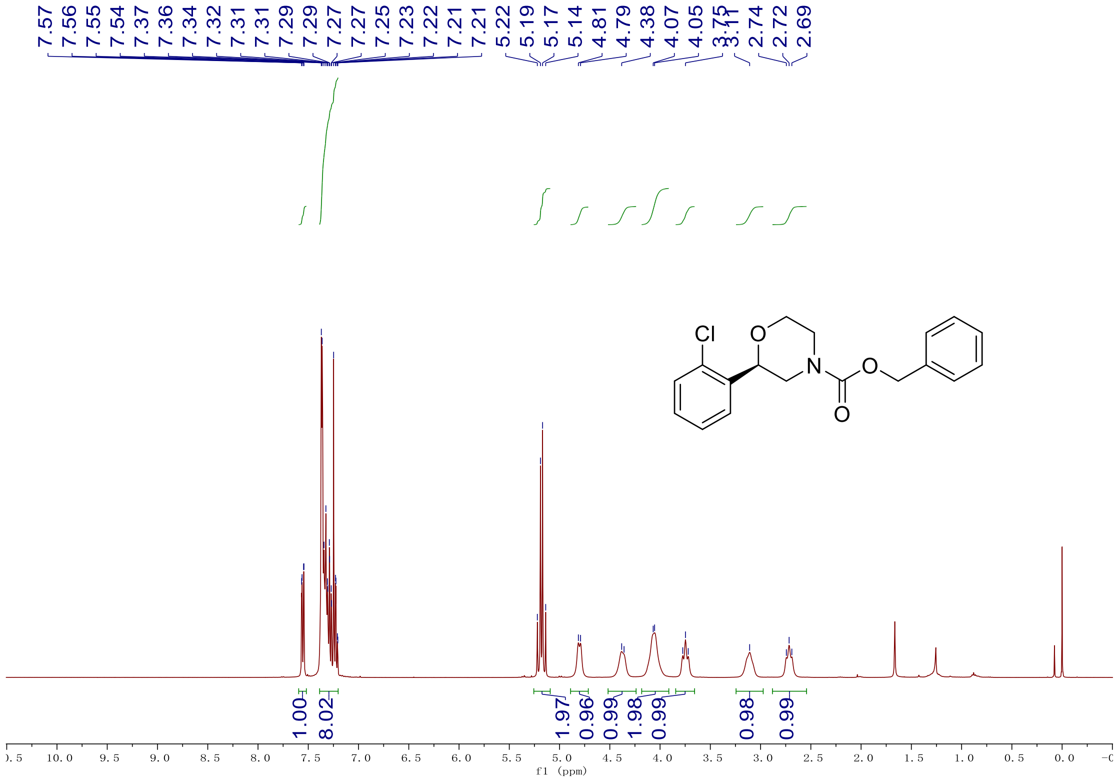

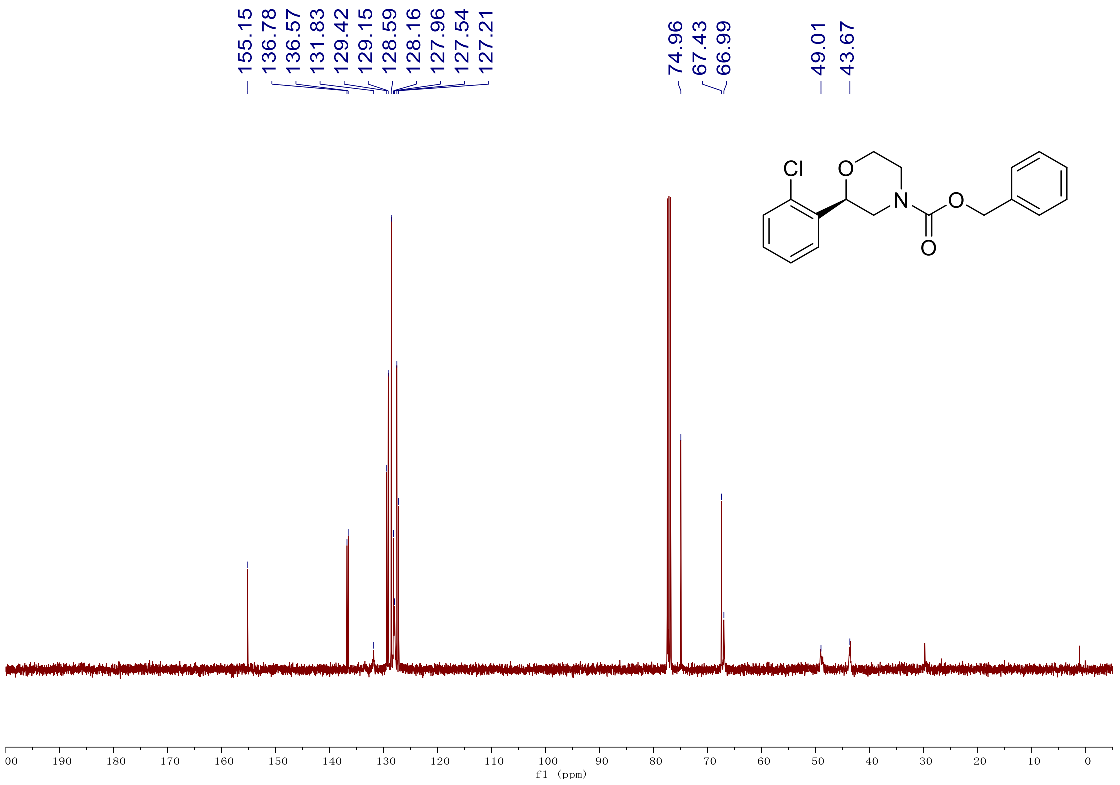

Benzyl (*R*)-2-(2-*o*-tolyl)morpholine-4-carboxylate (2o)

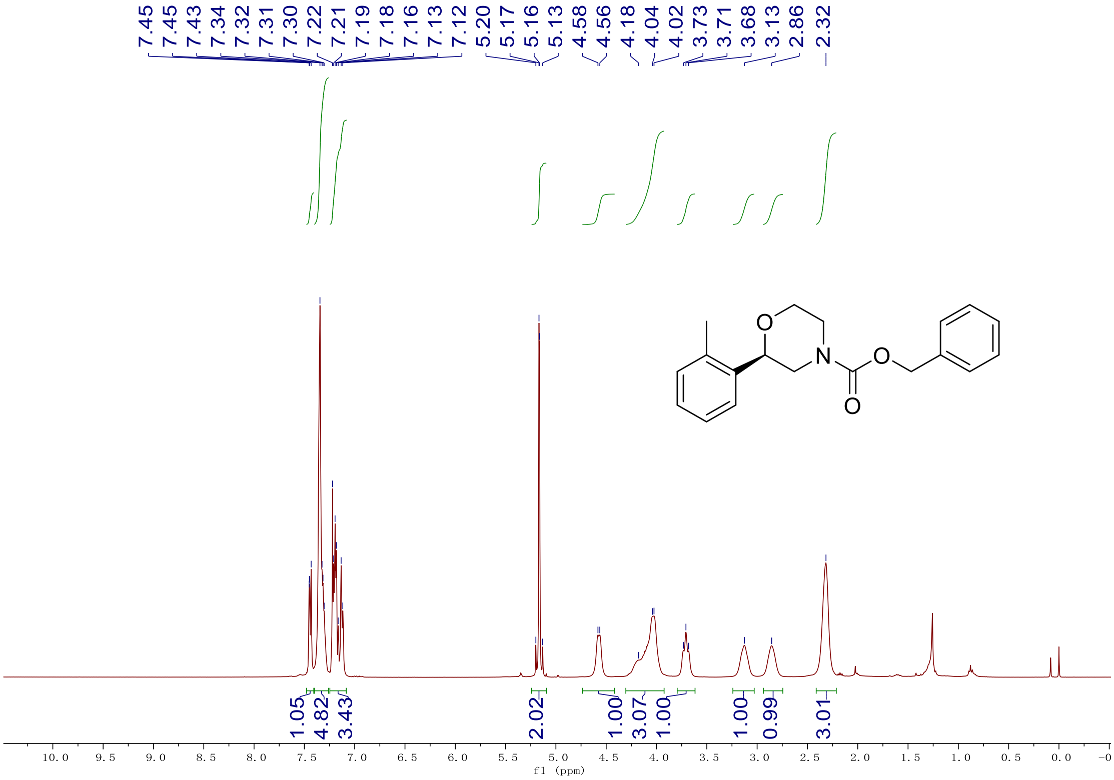

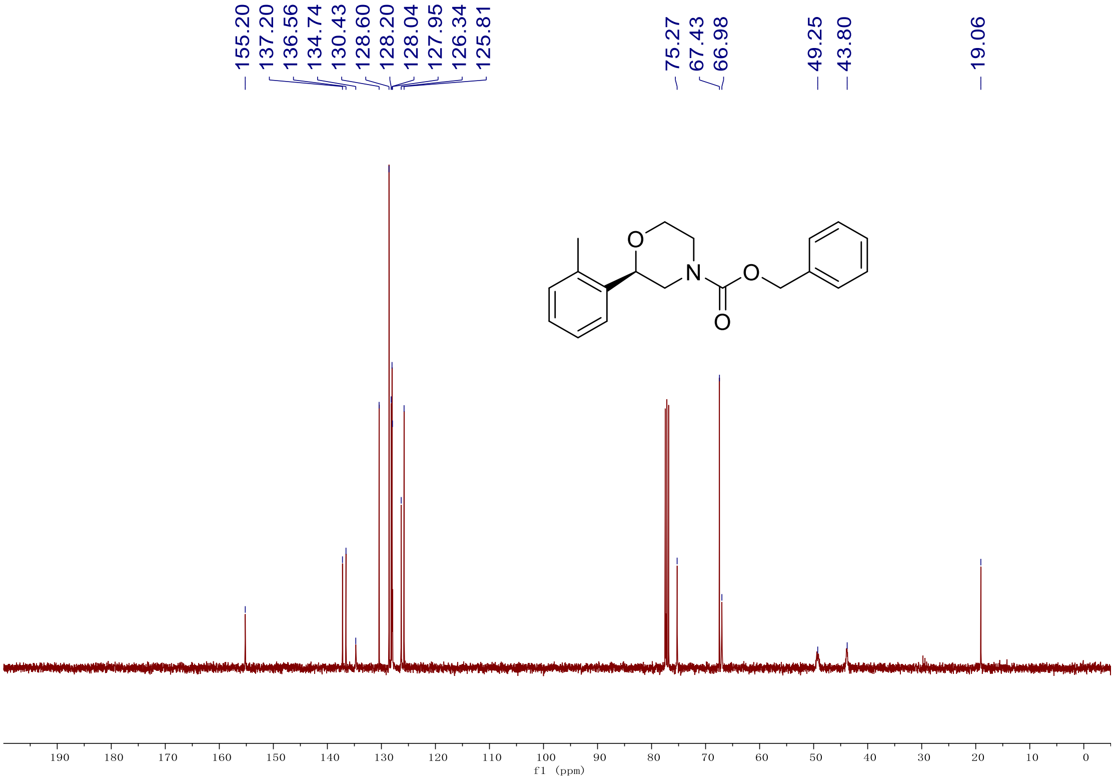

Benzyl (*R*)-2-(2-methoxyphenyl)morpholine-4-carboxylate (2p)

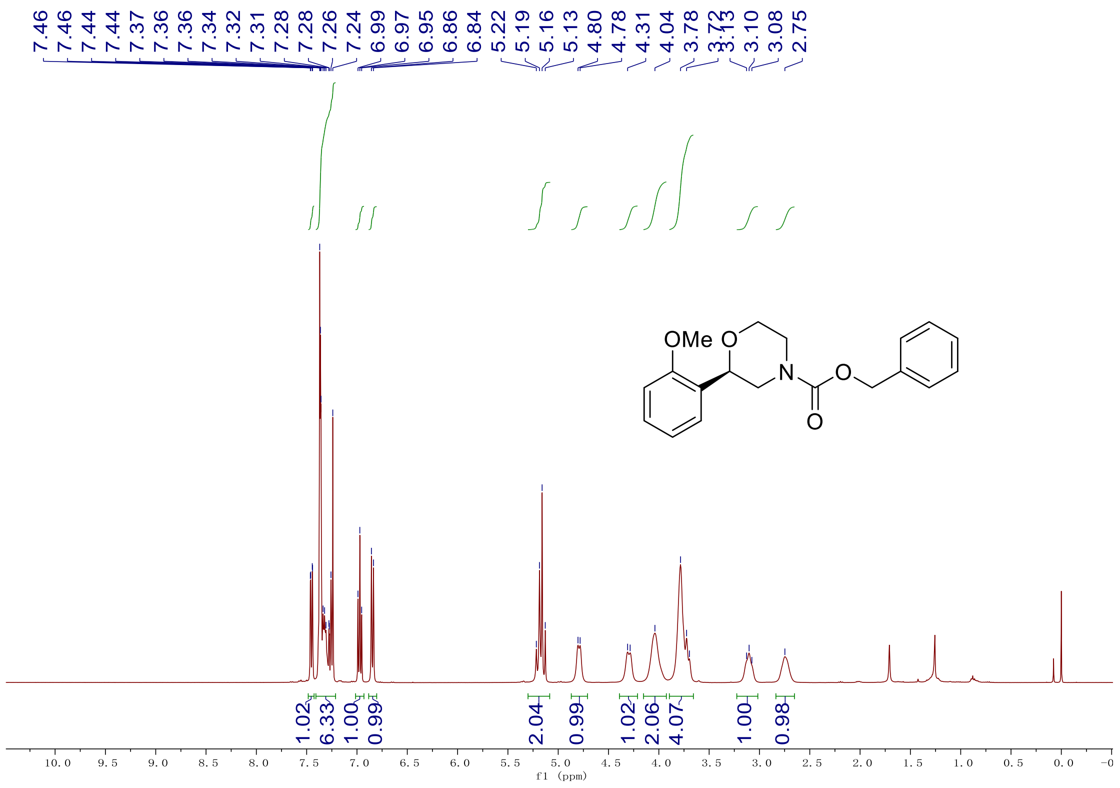

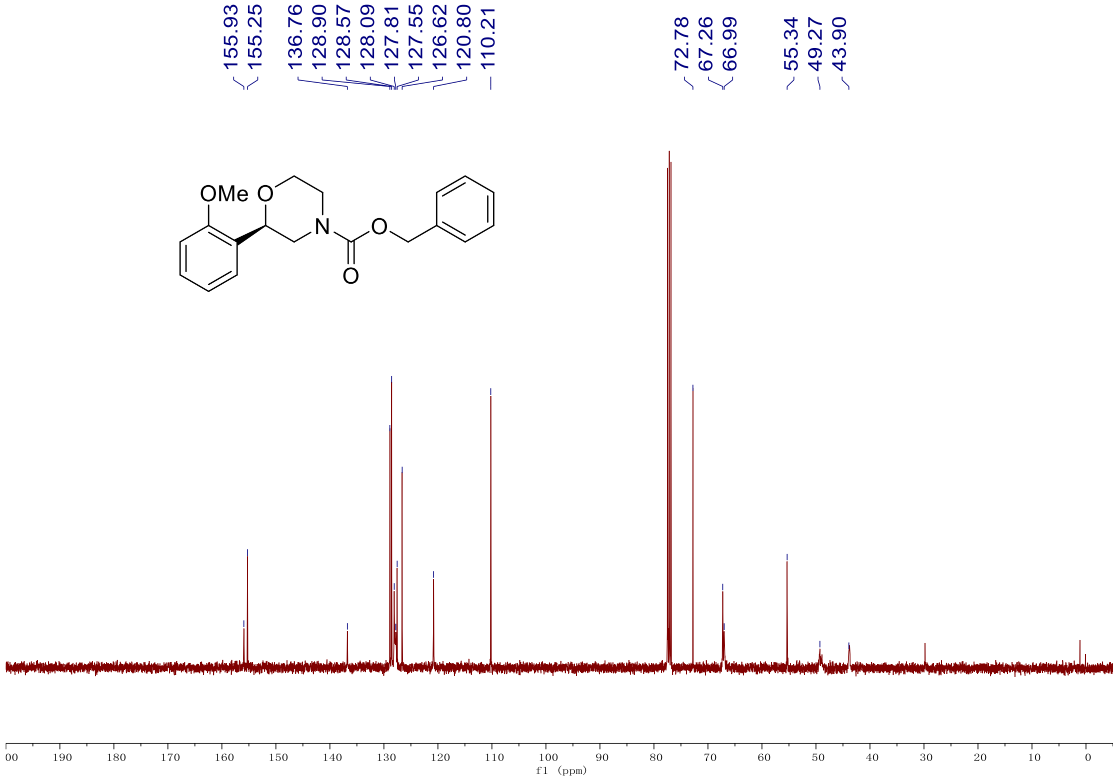

Benzyl (*R*)-2-(3,4-dichlorophenyl)morpholine-4-carboxylate (2q)

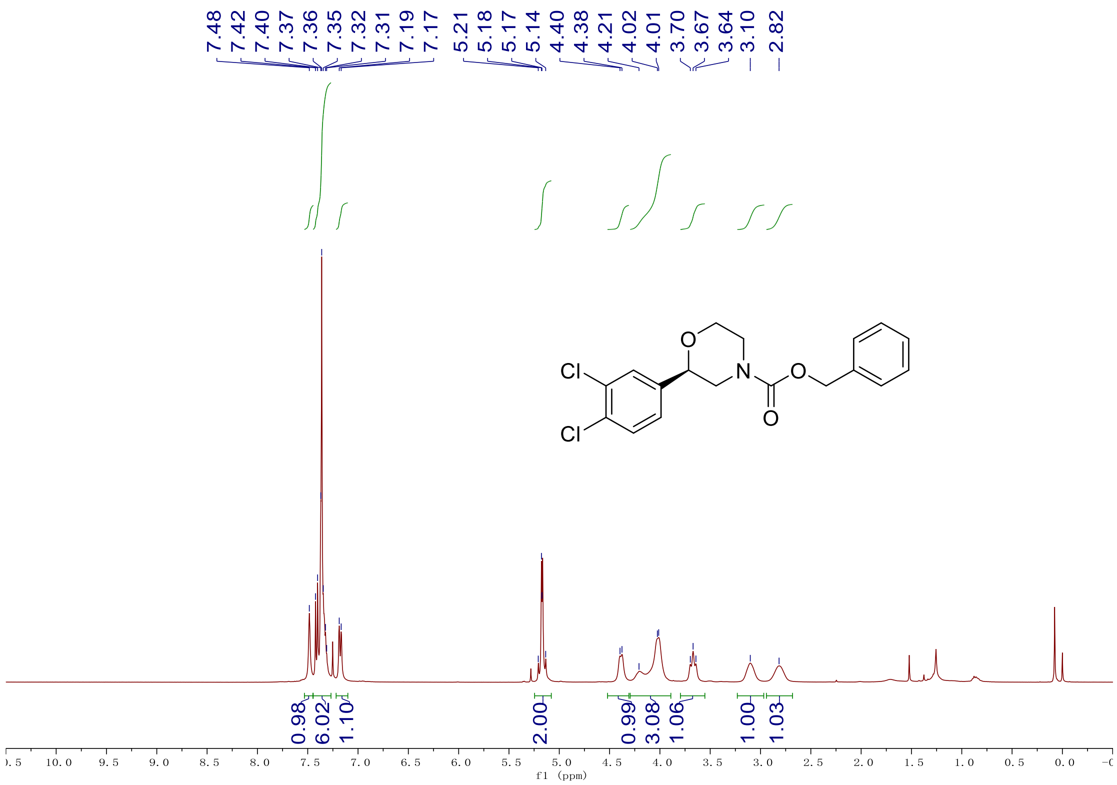

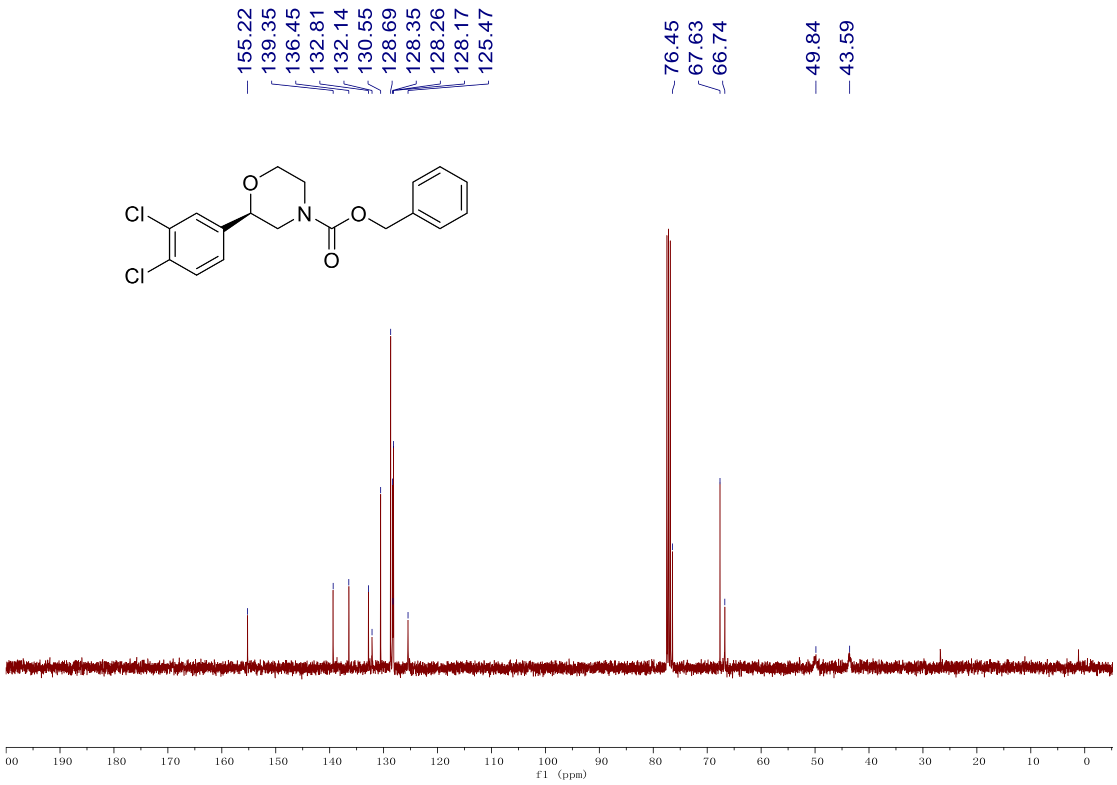

Benzyl (*R*)-2-(3,4-dimethoxyphenyl)morpholine-4-carboxylate (2r)

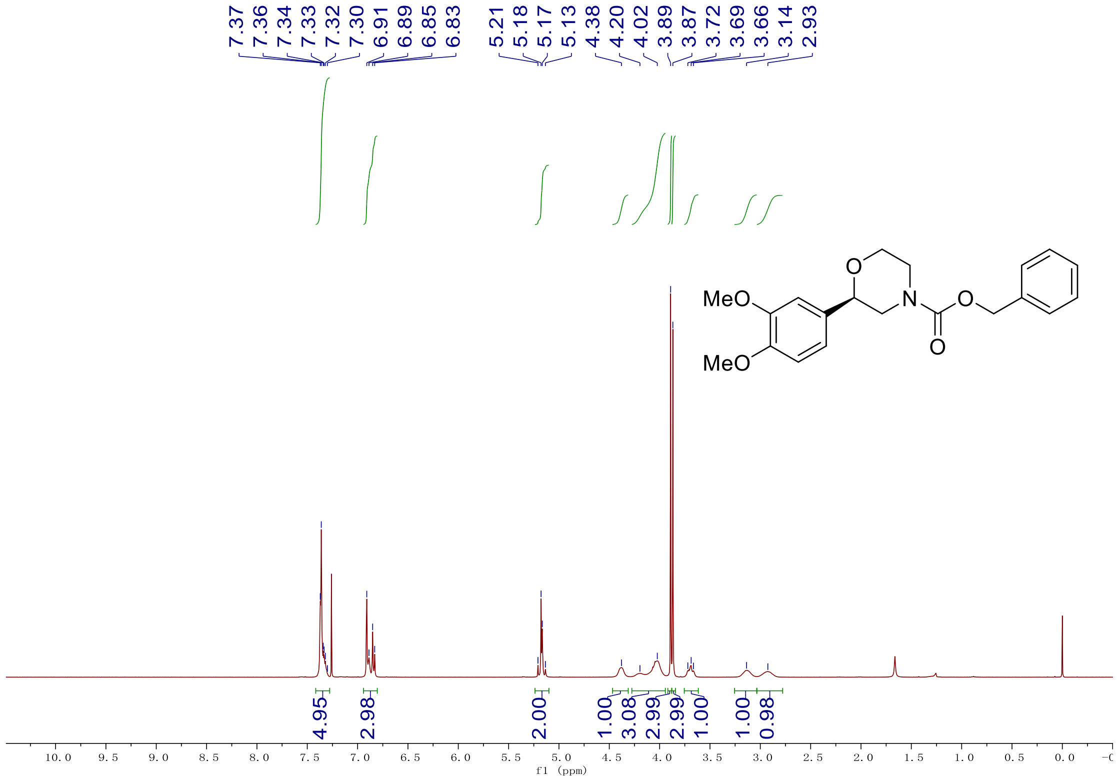

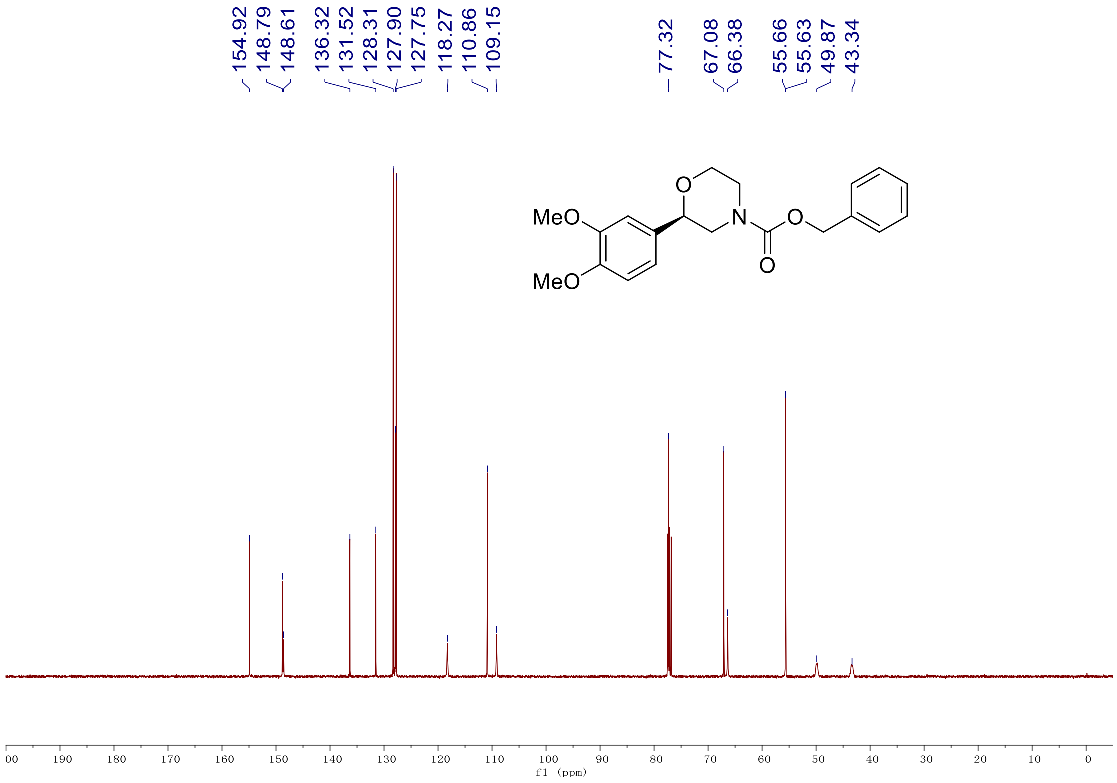

Benzyl (*R*)-2-(naphthalen-2-yl)morpholine-4-carboxylate (2s)

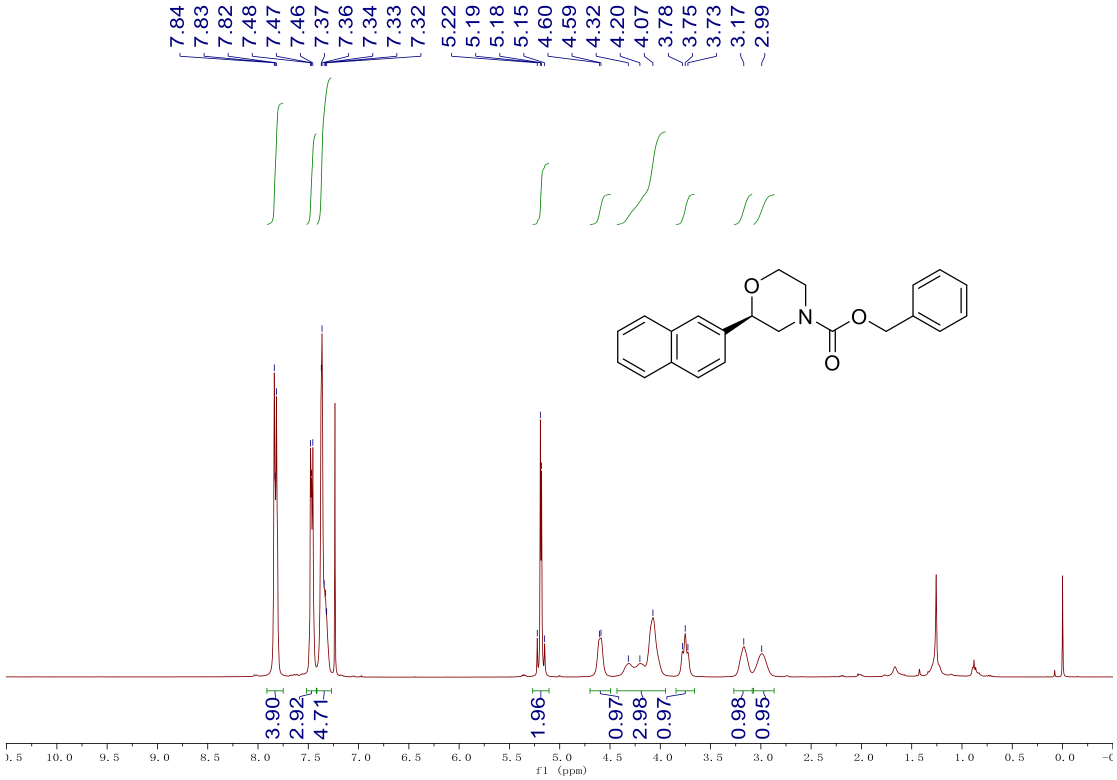

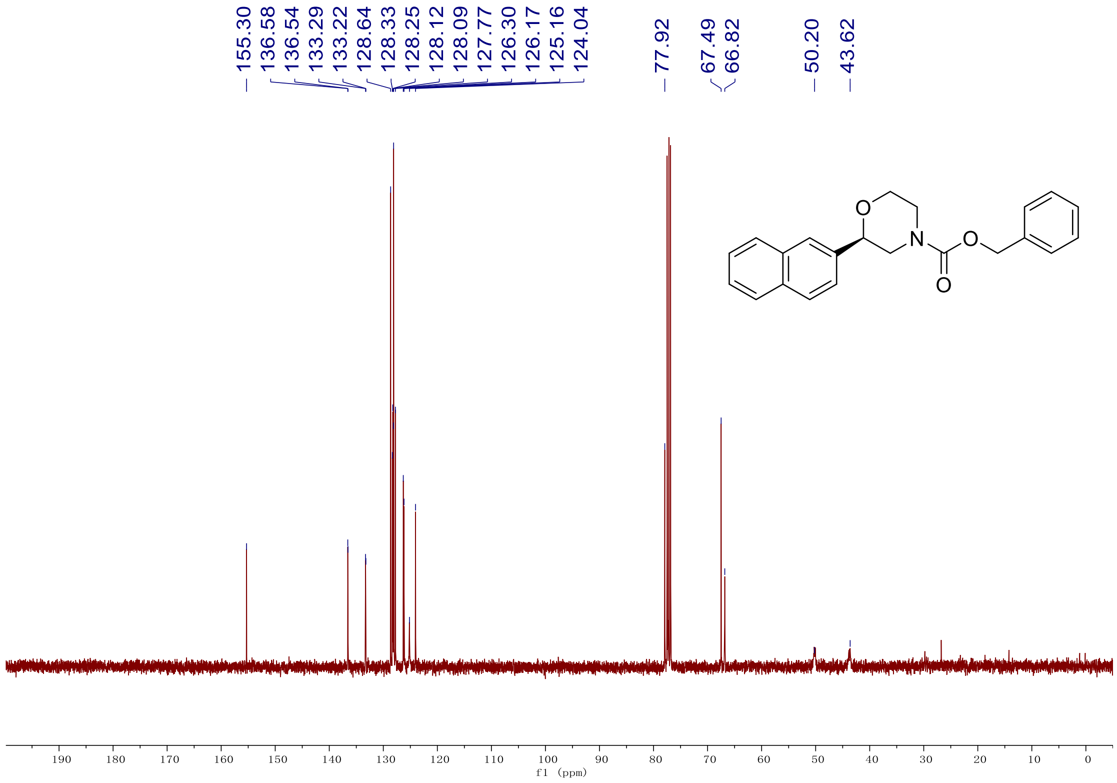

Benzyl (*S*)-2-(thiophen-2-yl)morpholine-4-carboxylate (2t)

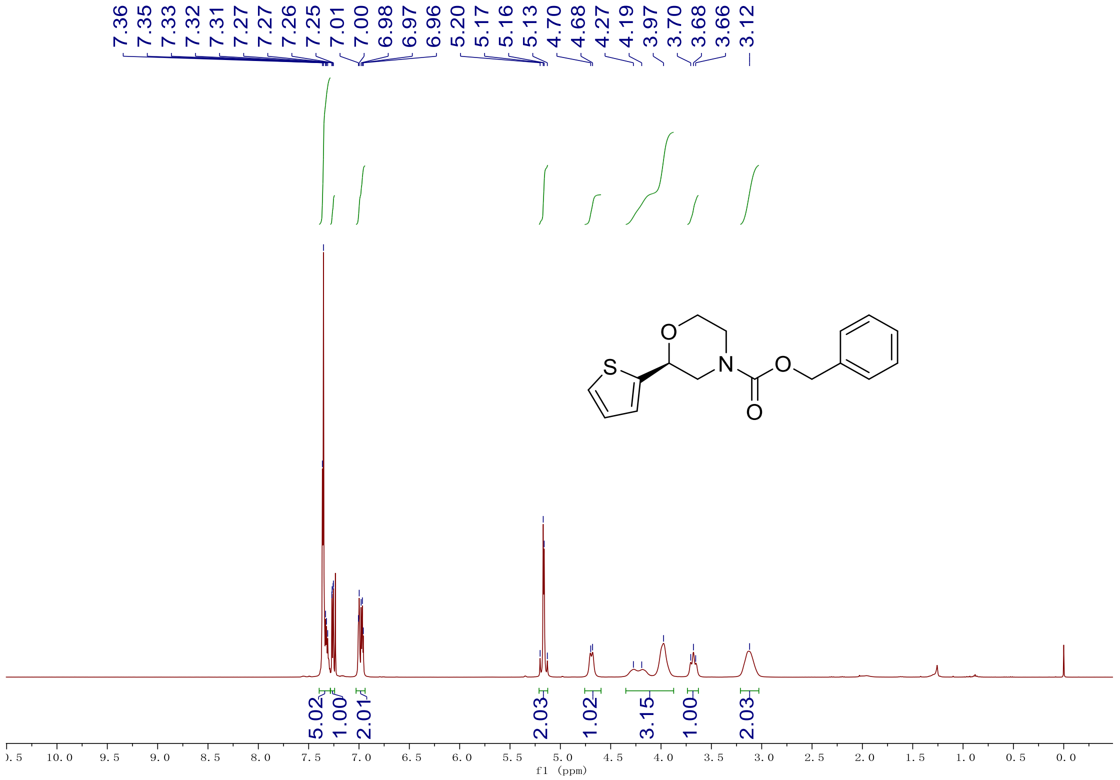

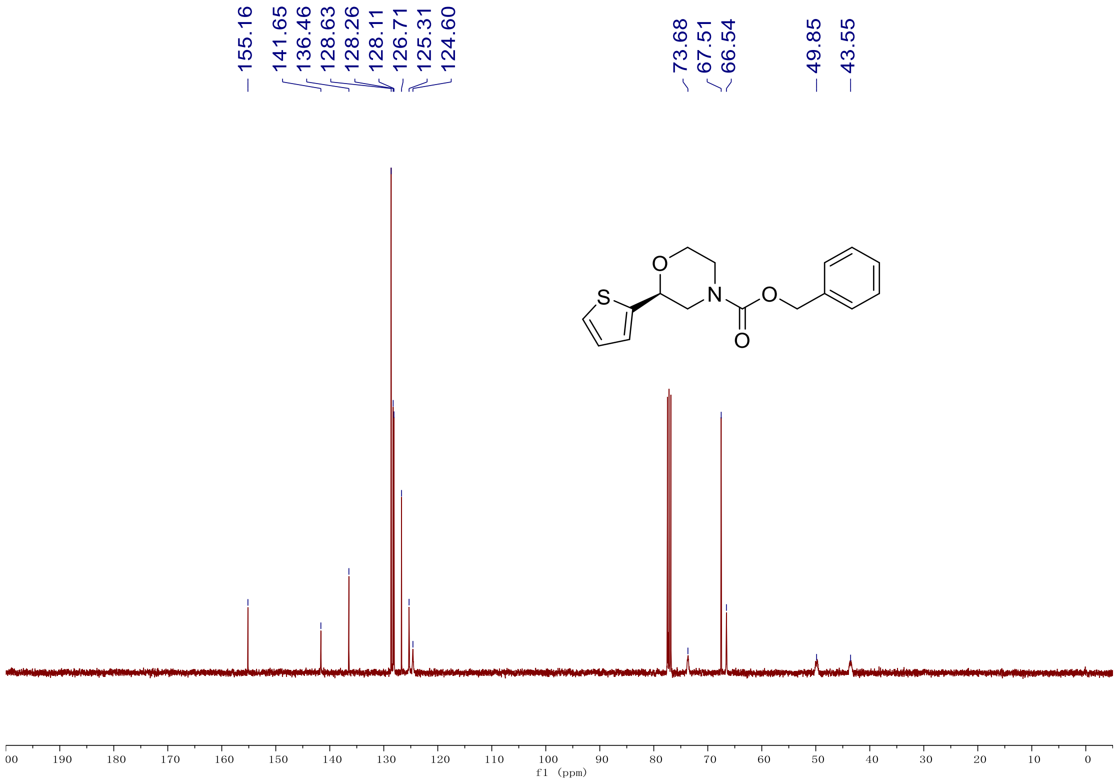

Benzyl (*R*)-2-(*tert*-butyl)morpholine-4-carboxylate (2u)

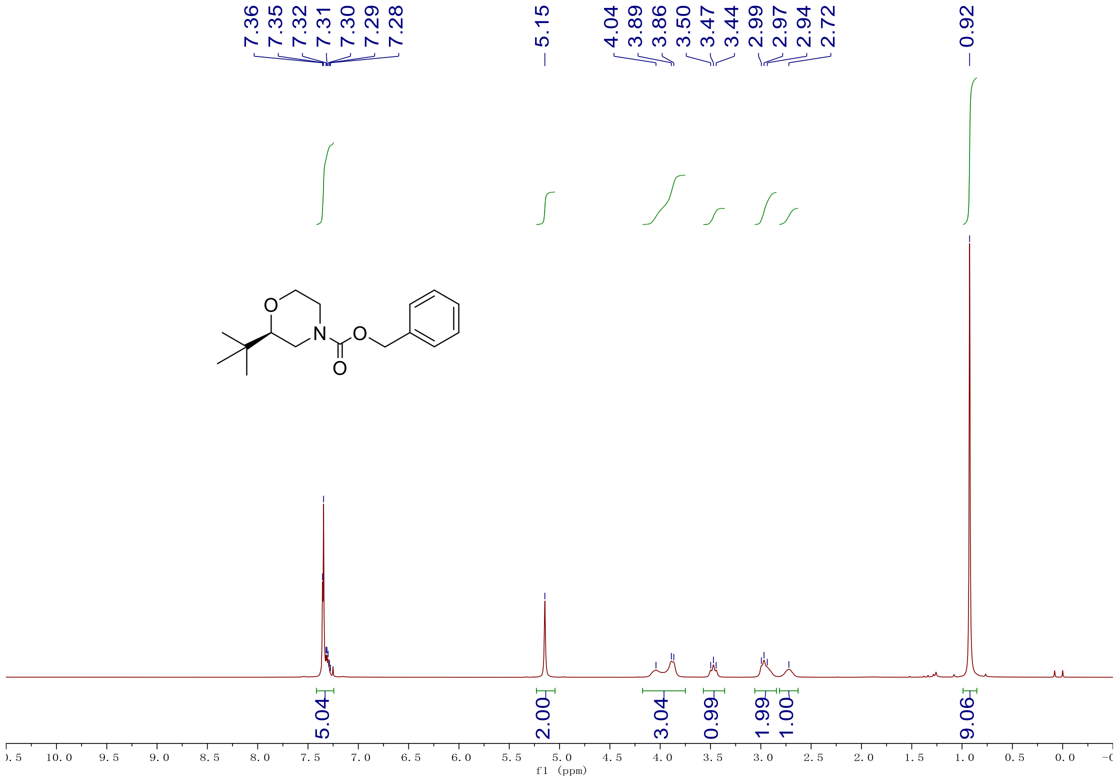

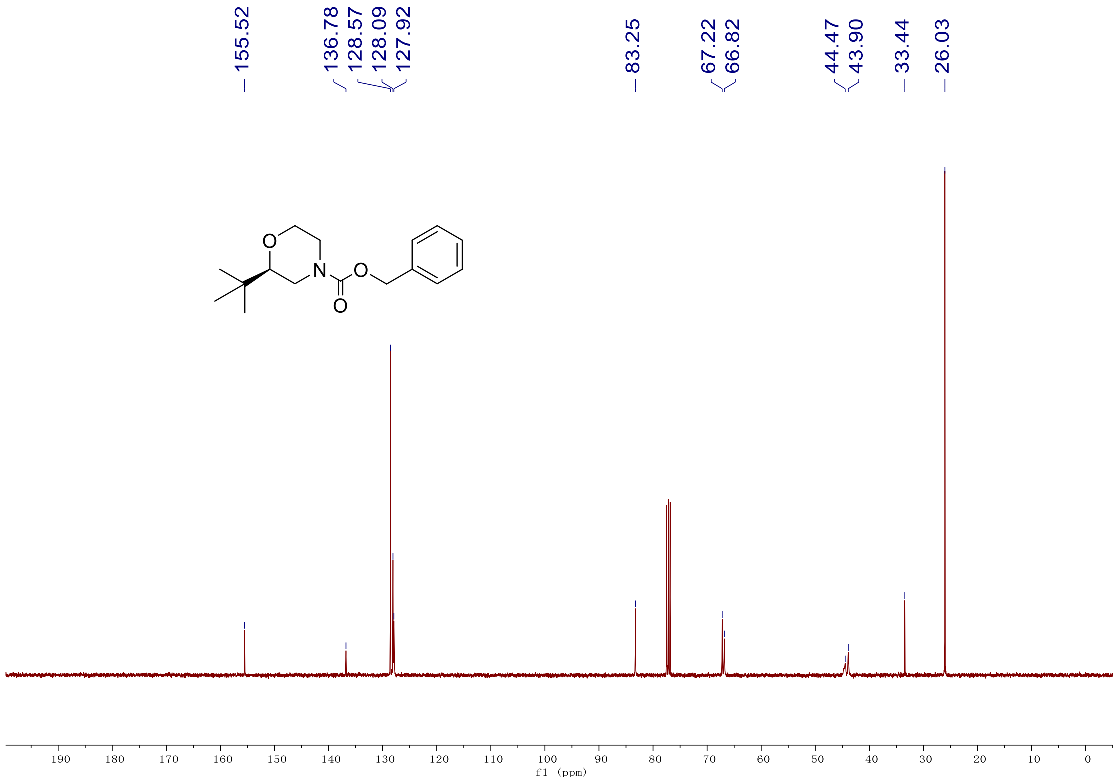

Benzyl (*R*)-2-isopropylmorpholine-4-carboxylate (2v)

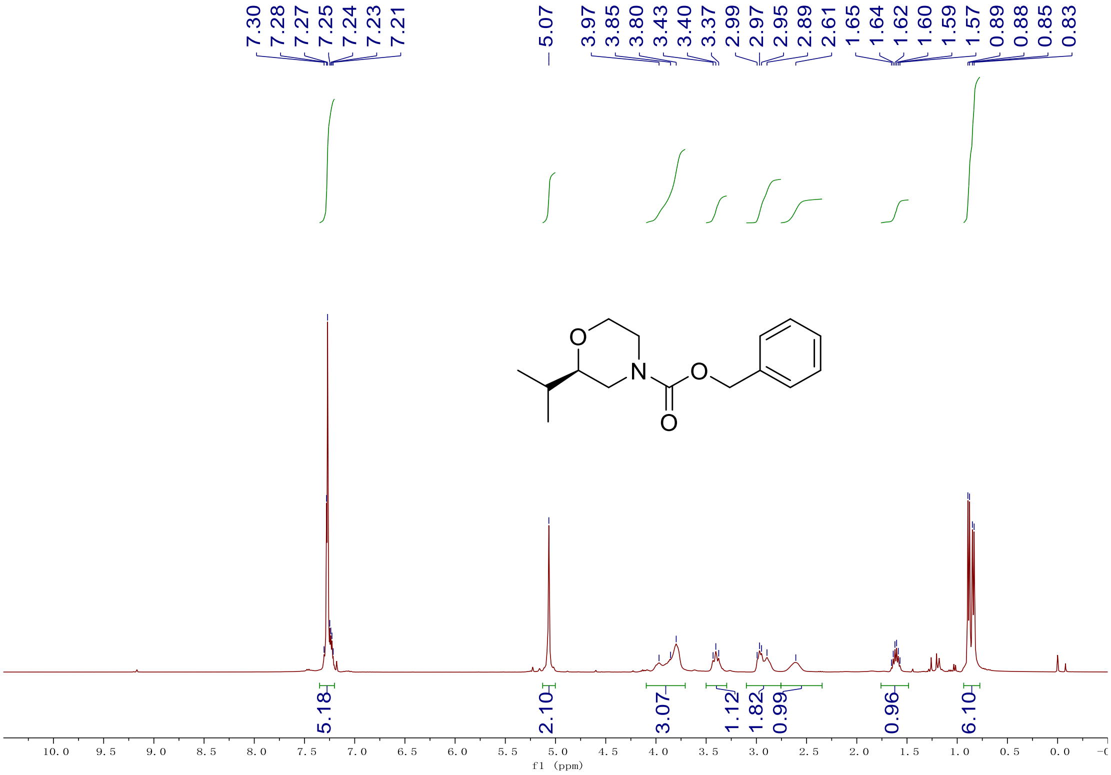

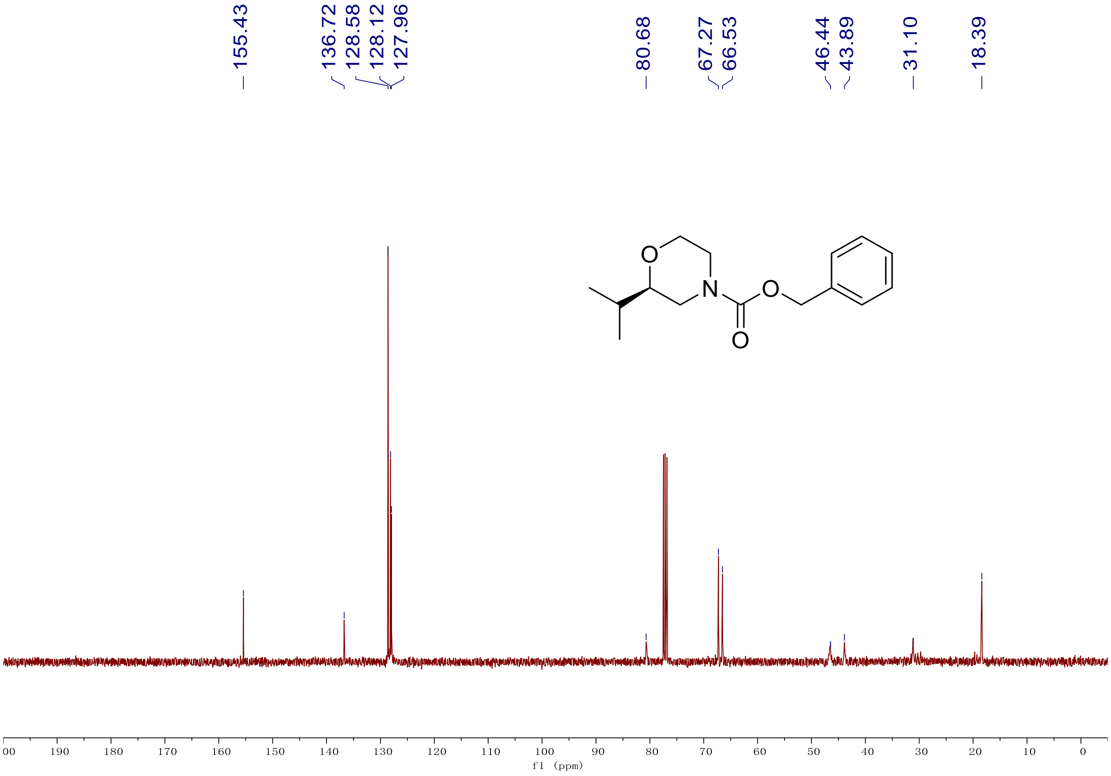

Benzyl (*R*)-2-ethylmorpholine-4-carboxylate (2w)

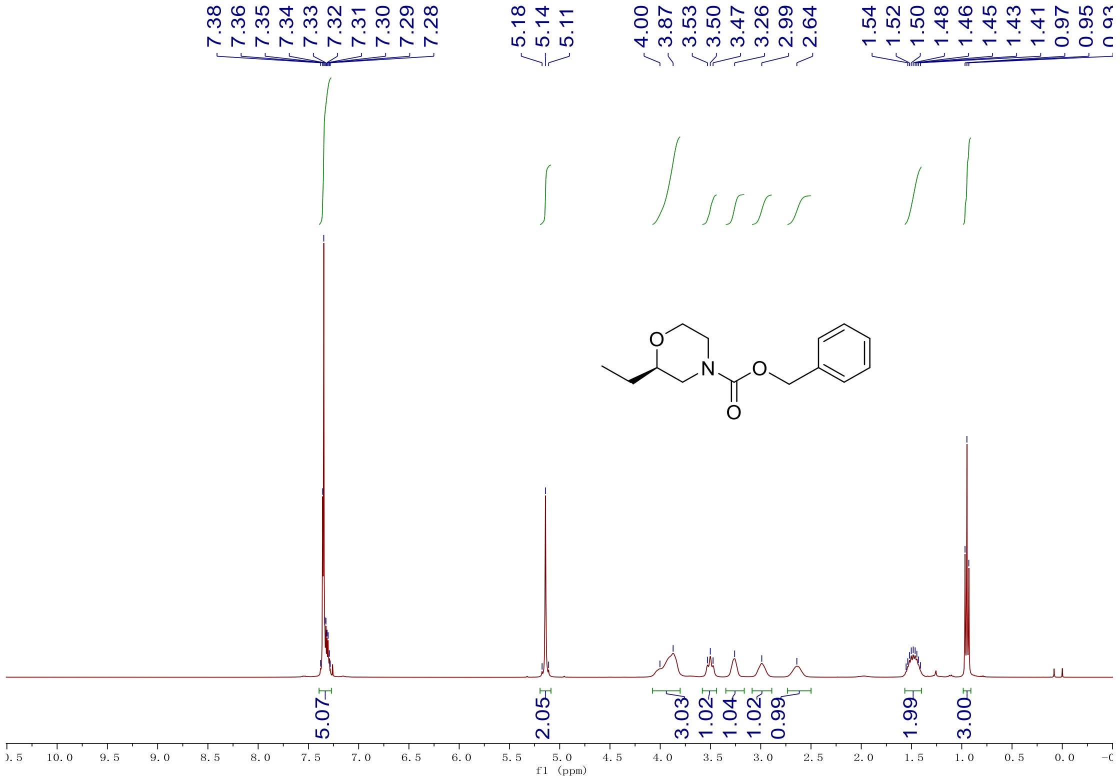

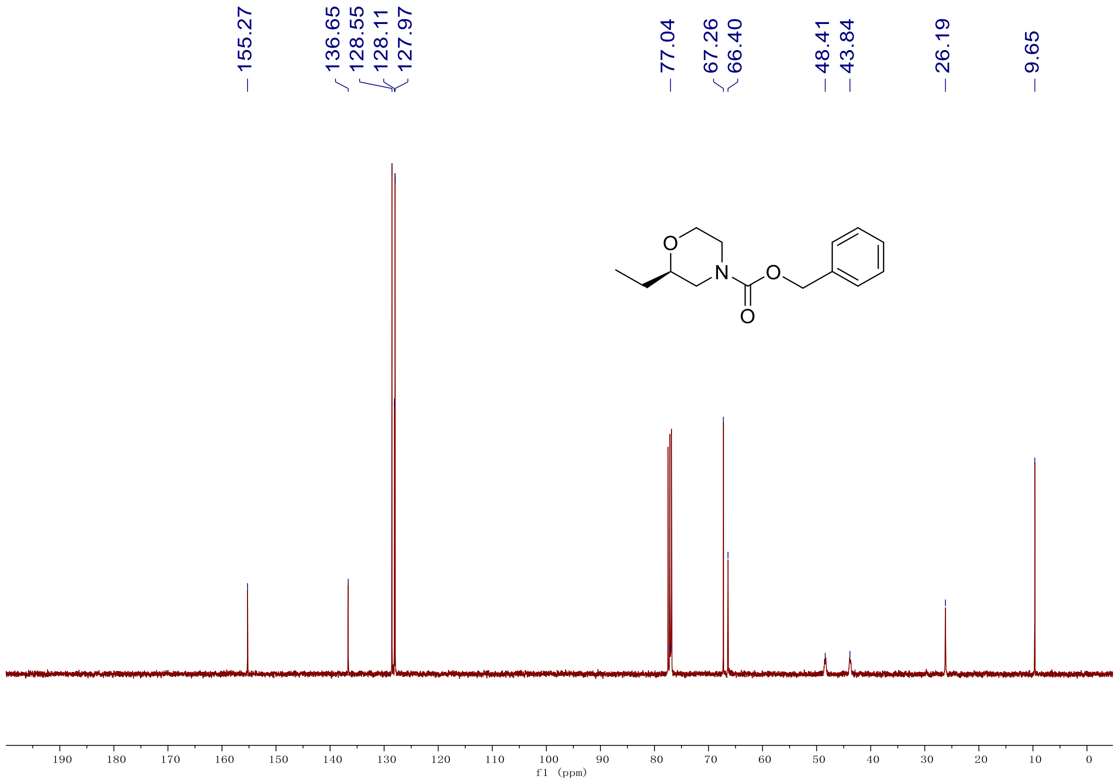

Benzyl 3-phenylmorpholine-4-carboxylate (2x)

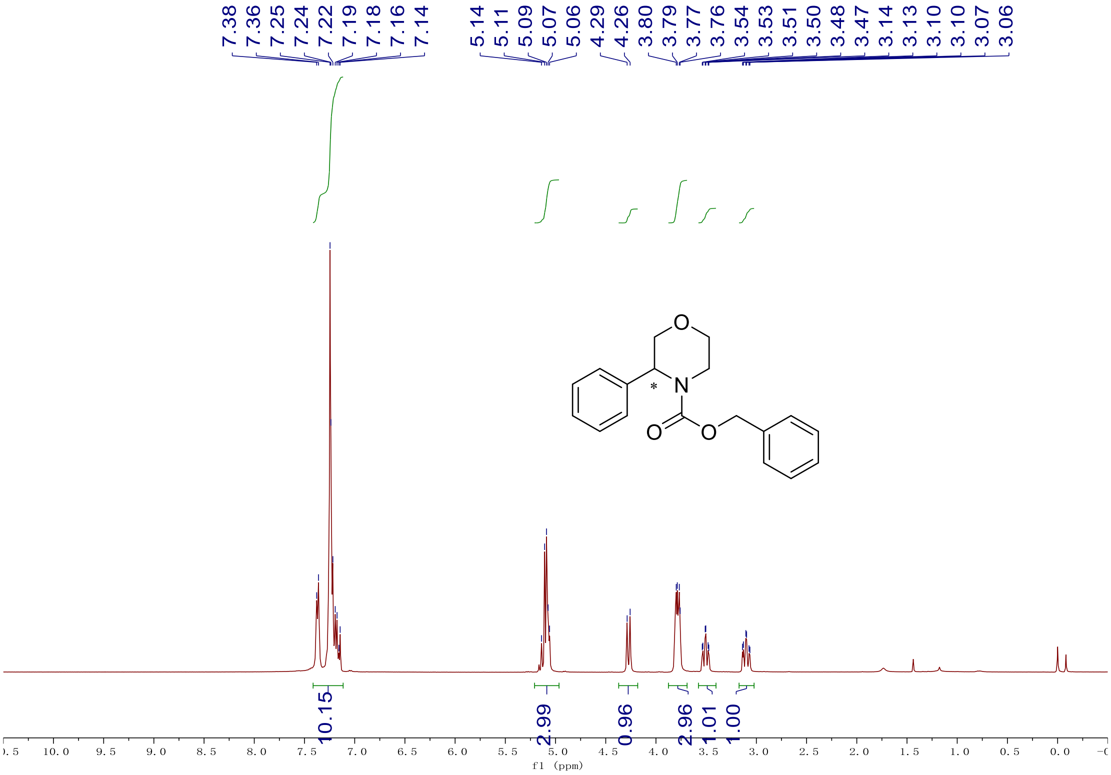

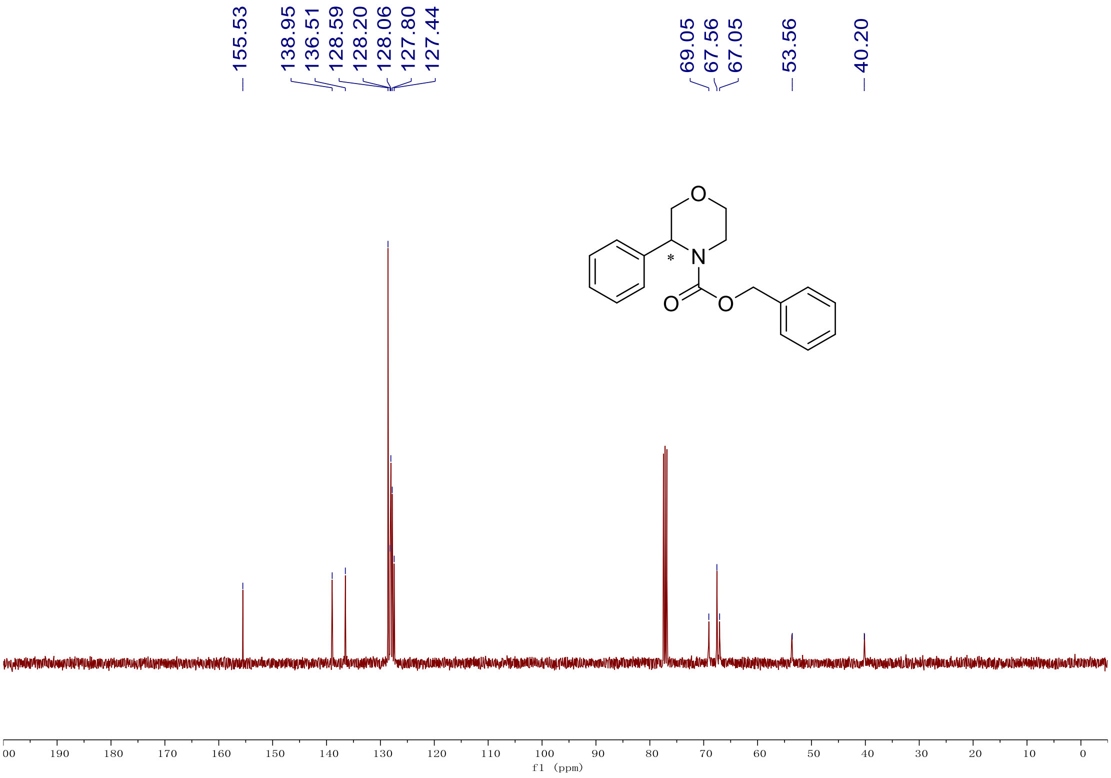

Benzyl 2-(4-bromophenyl)-3-methylmorpholine-4-carboxylate (2y)

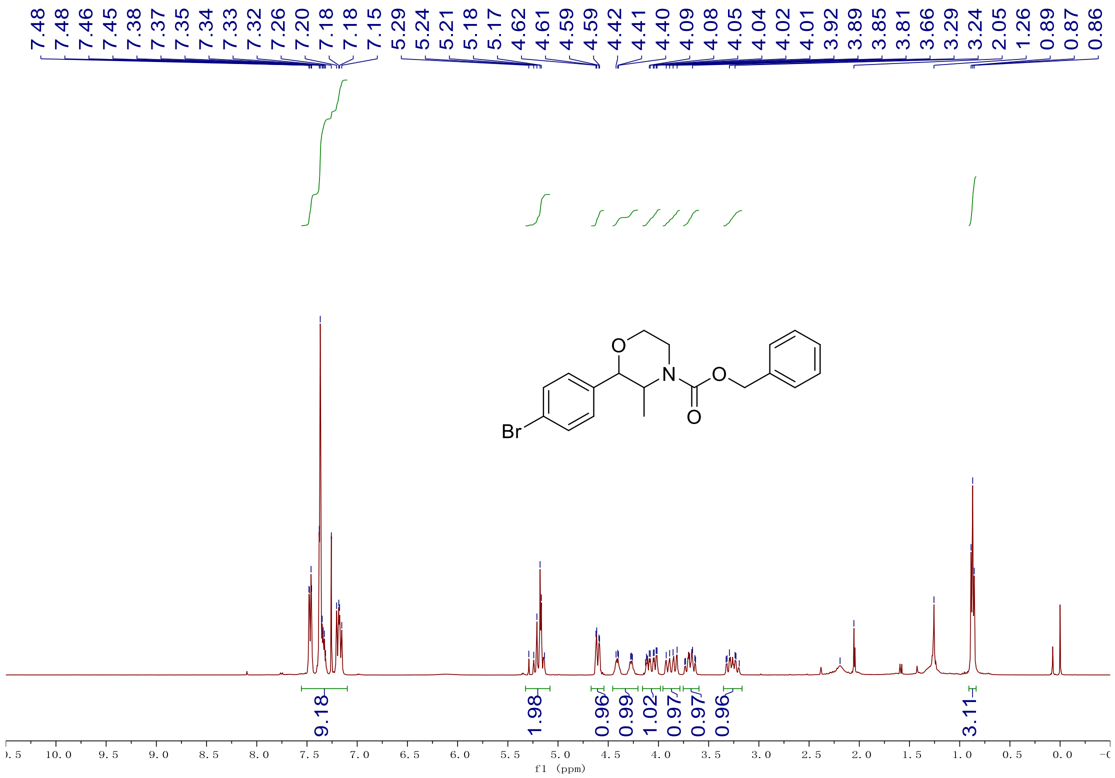

4-Nitrobenzyl (*R*)-2-phenylmorpholine-4-carboxylate (2a-NO_2)

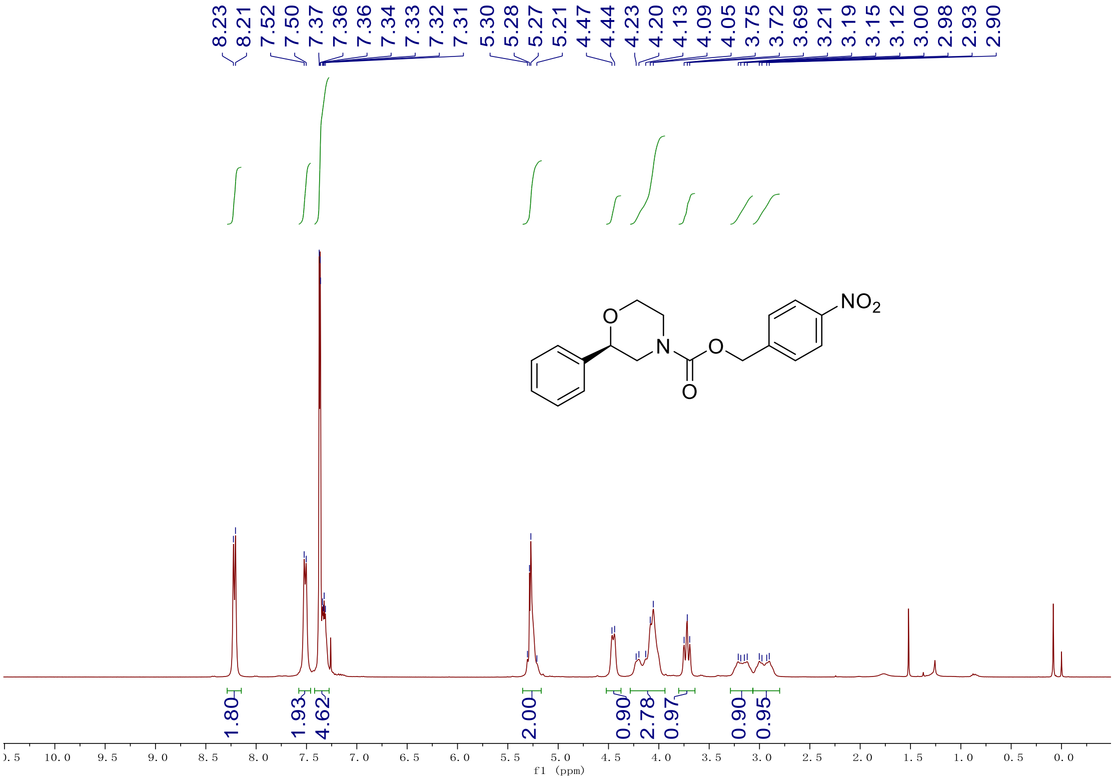

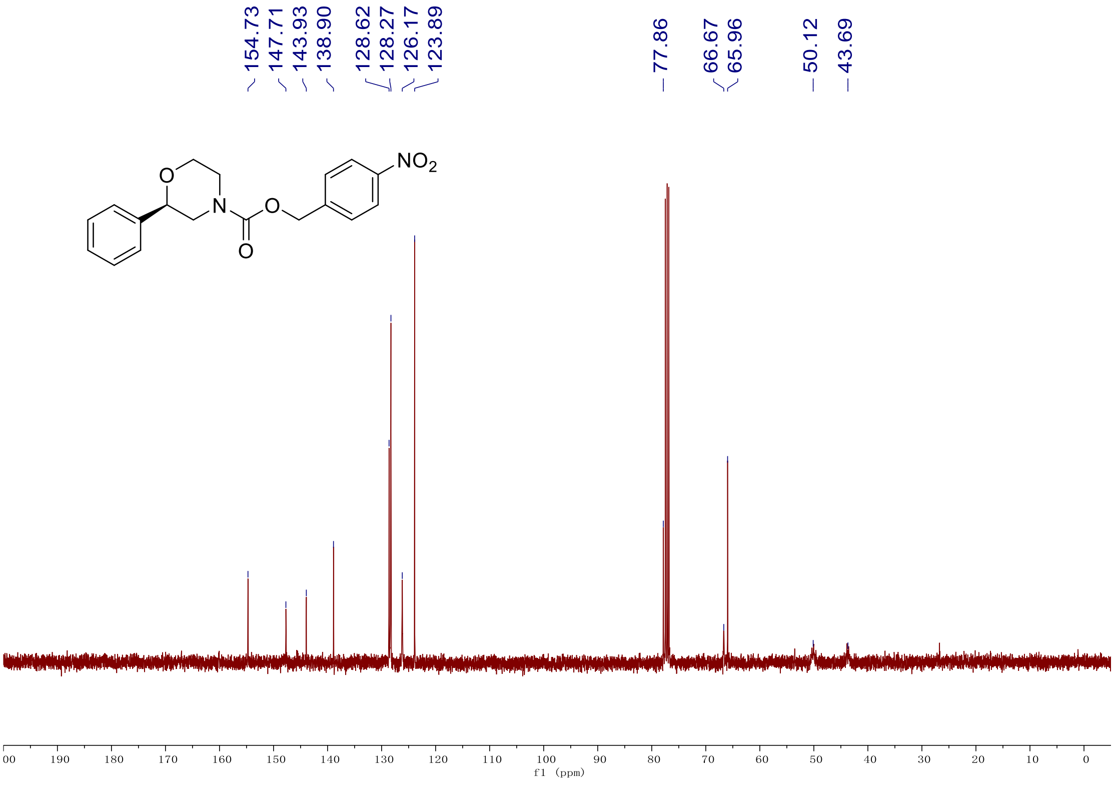

Isobutyl (*R*)-2-phenylmorpholine-4-carboxylate (2a-COO*i*Bu)

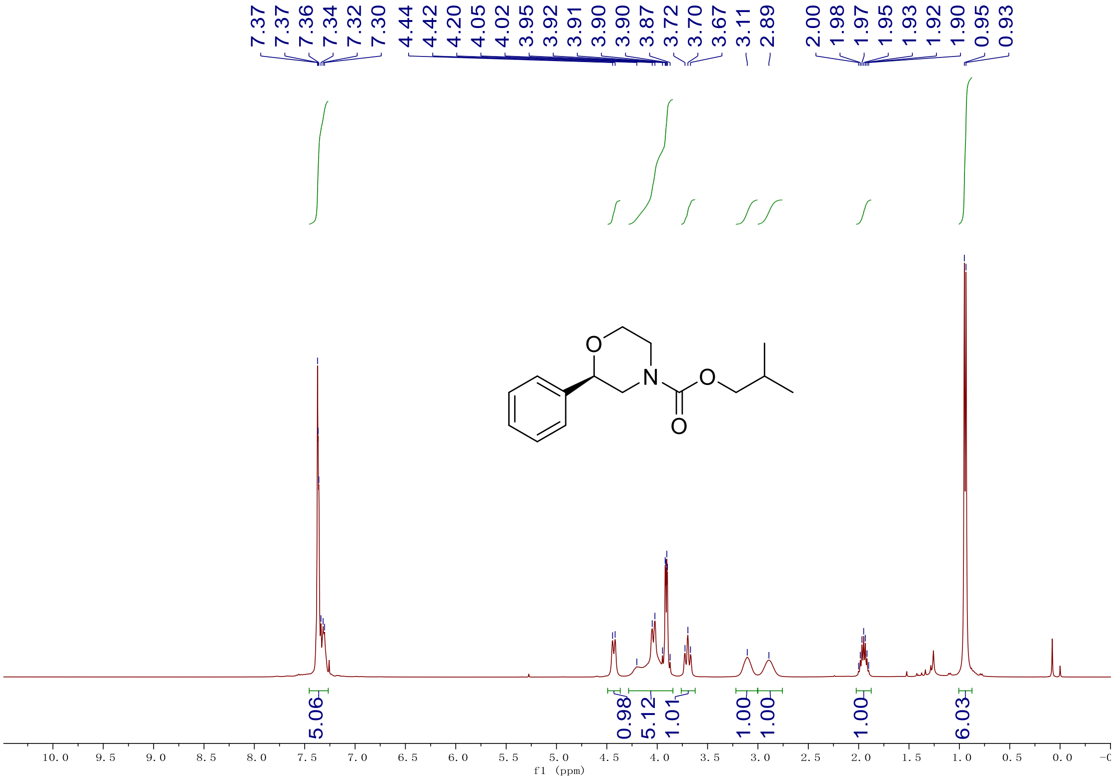

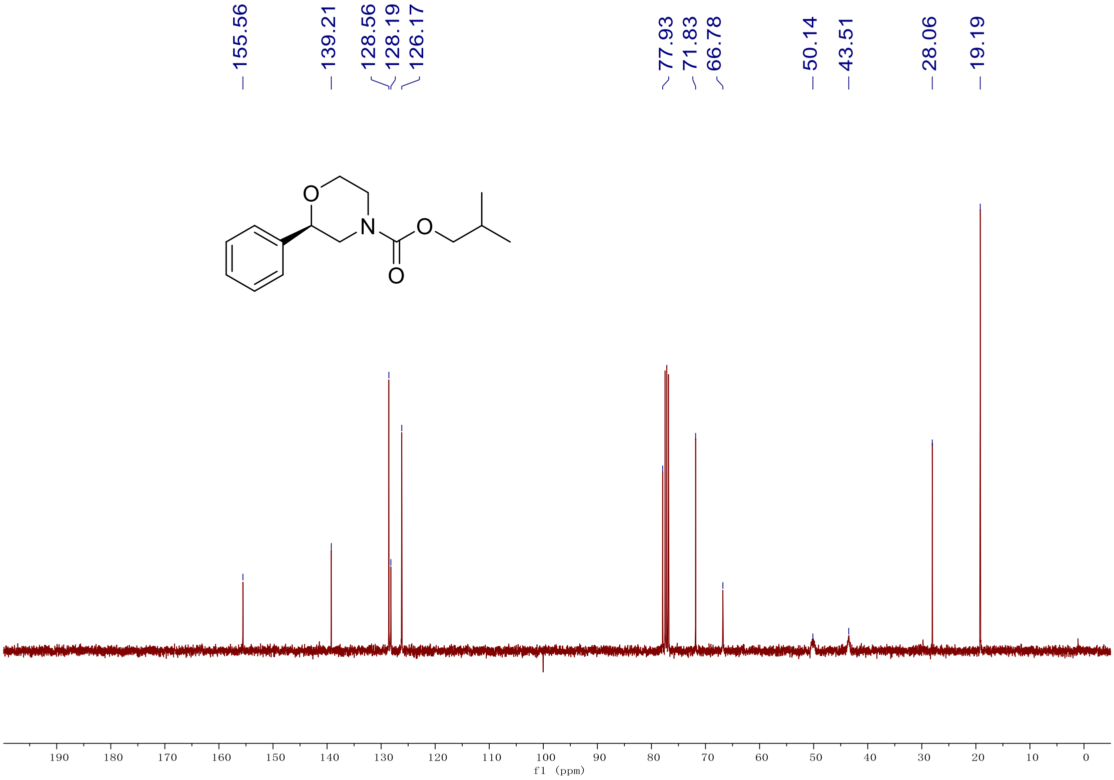

***tert*-Butyl (*R*)-2-phenylmorpholine-4-carboxylate (2a-Boc)**

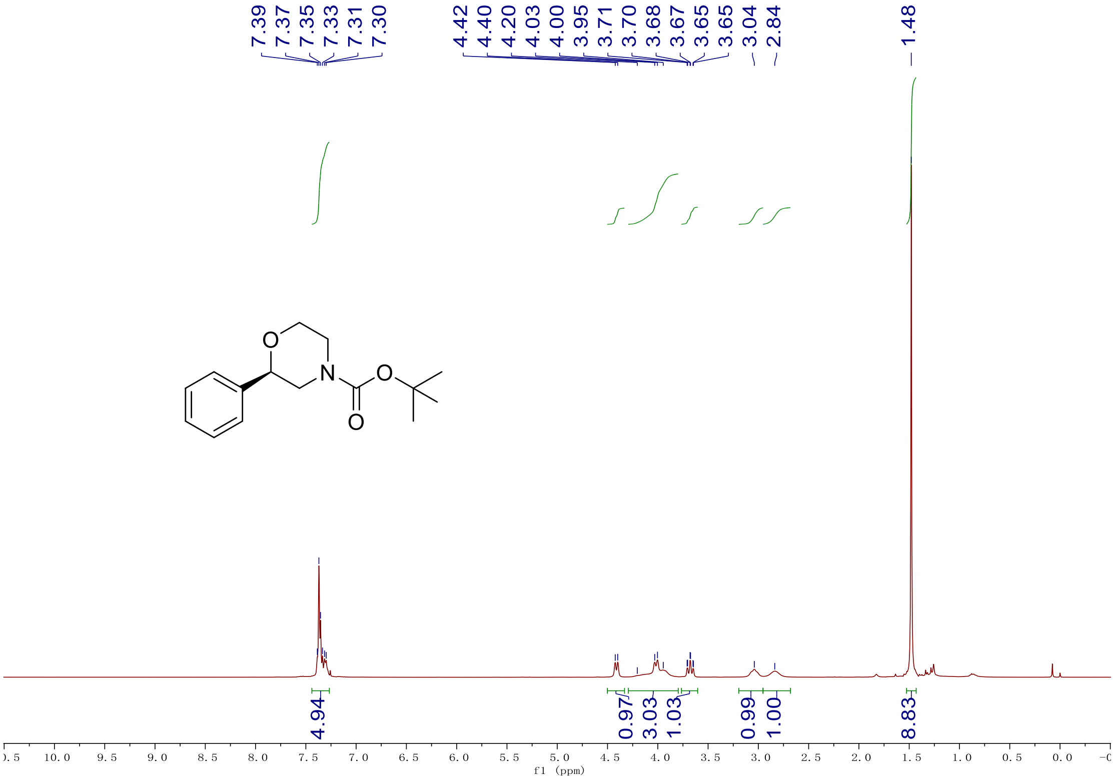

Benzyl (2*R*)-2,3-d_2-2-phenylmorpholine-4-carboxylate (2a-D)

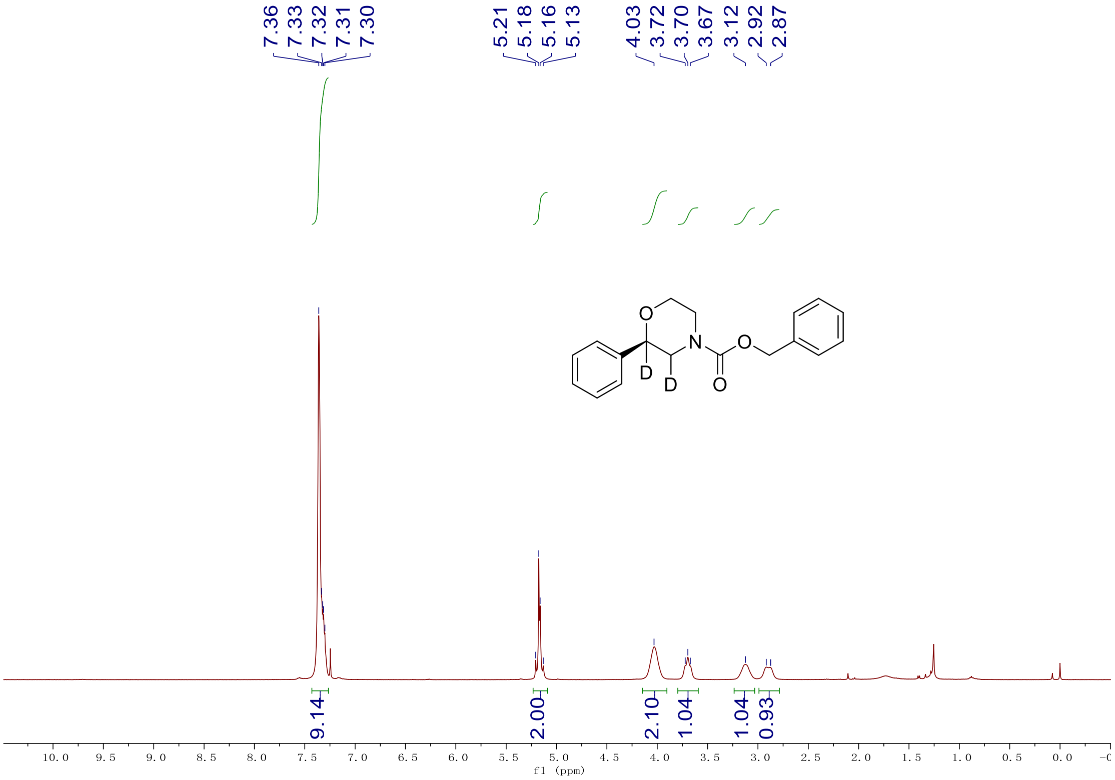

(*R*)-2-(4-fluorophenyl)morpholine (3b)

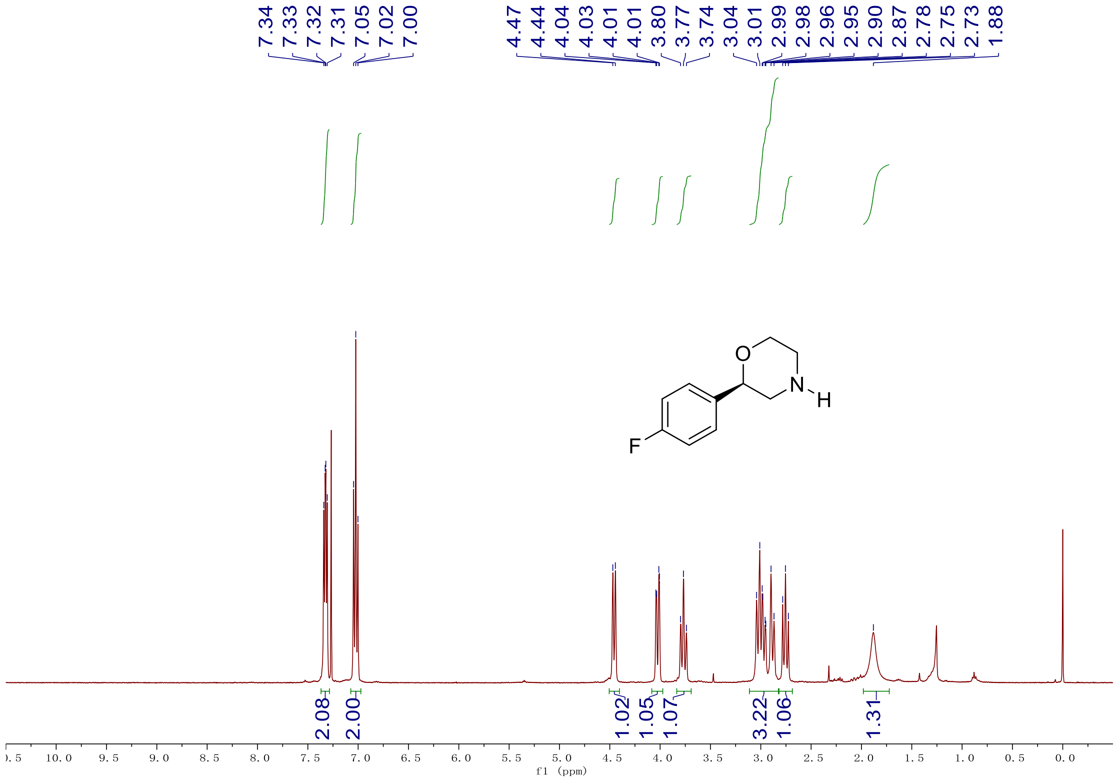

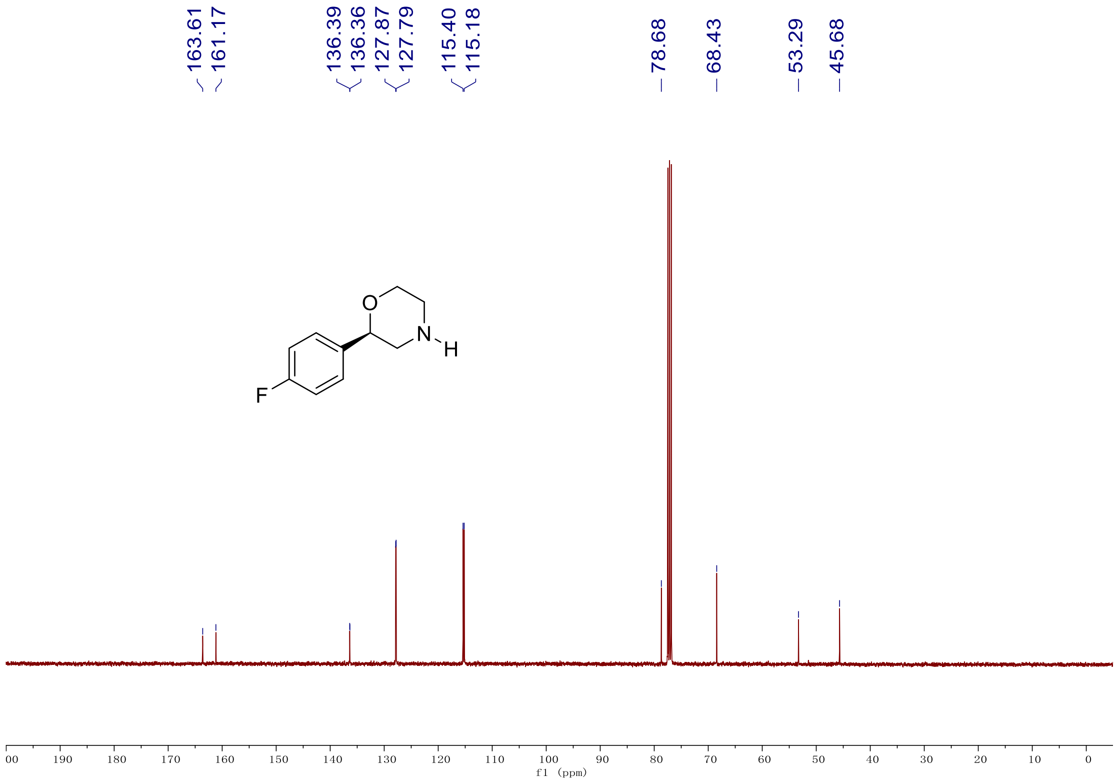

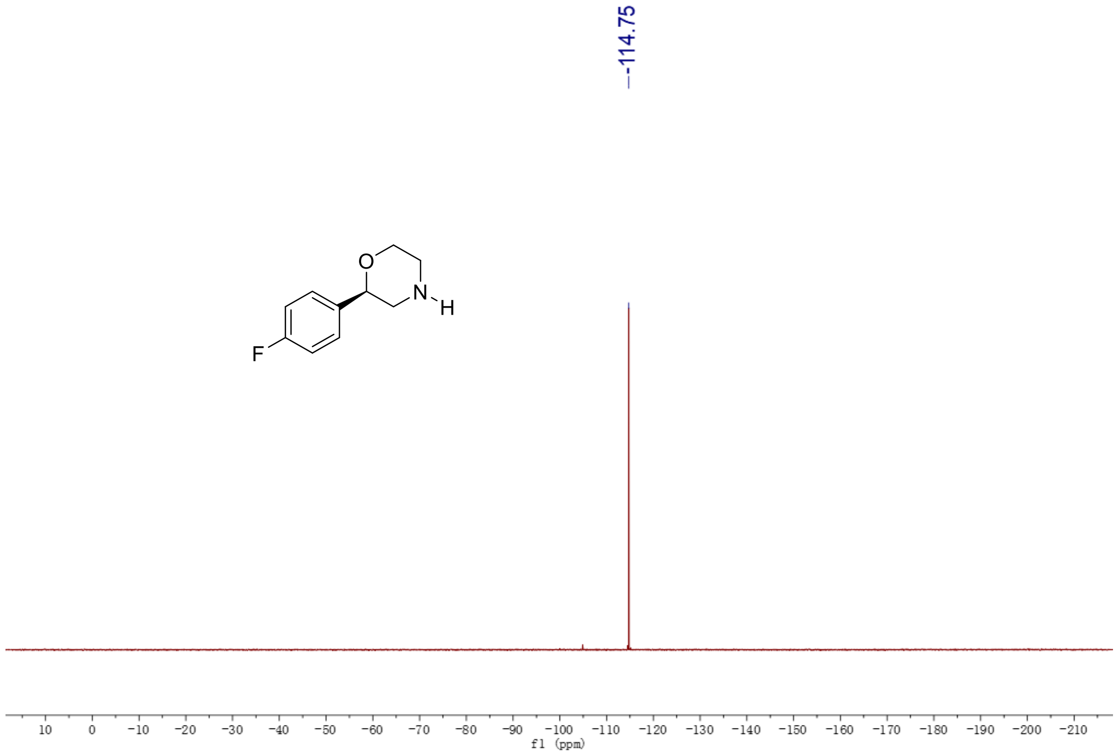
-114.75
O
N
H
F
10 0 −10 −20 −30 −40 −50 −60 −70 −80 −90 −100 −110 −120 −130 −140 −150 −160 −170 −180 −190 −200 −210
f1 (ppm)

(*R*)-2-(3-methoxyphenyl)-4-propylmorpholine (3l')

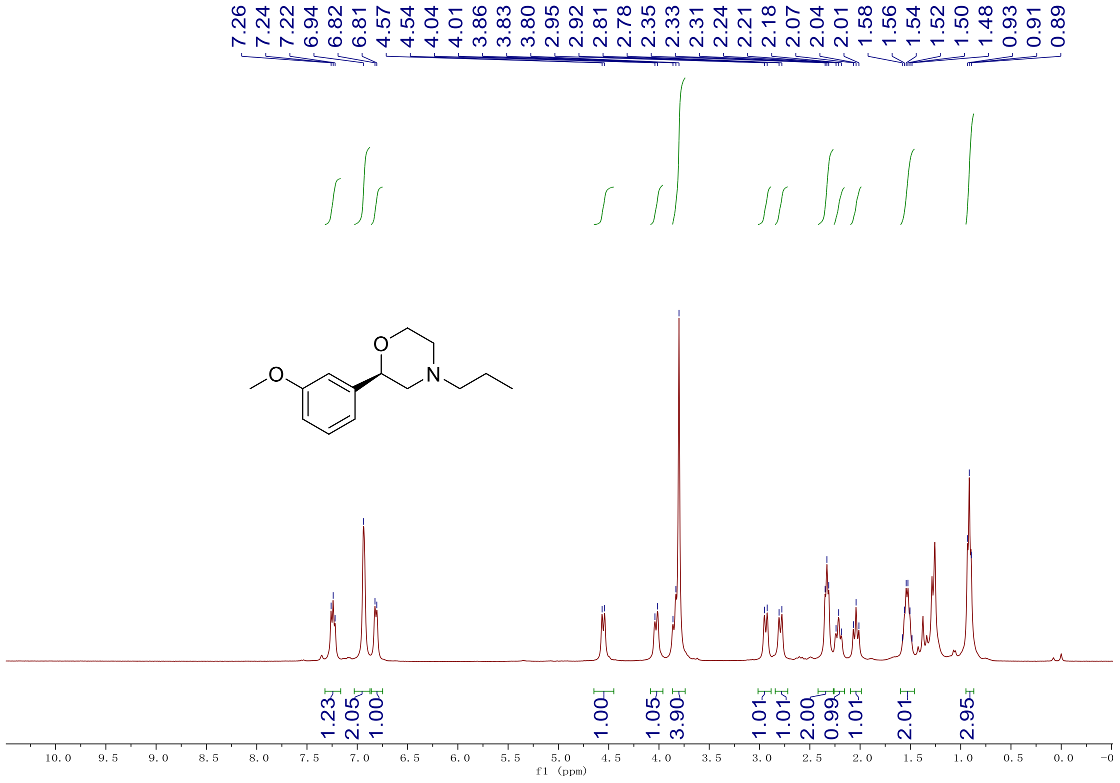

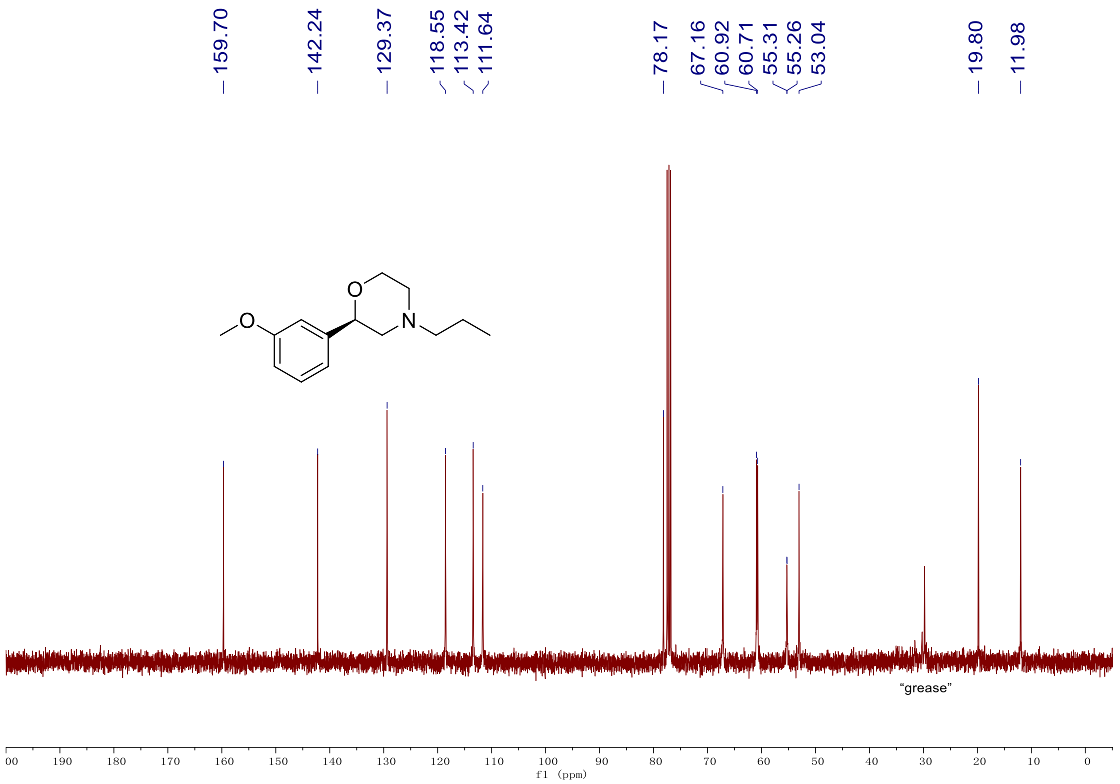

8. HPLC Charts

Benzyl (*R*)-2-phenylmorpholine-4-carboxylate (2a)

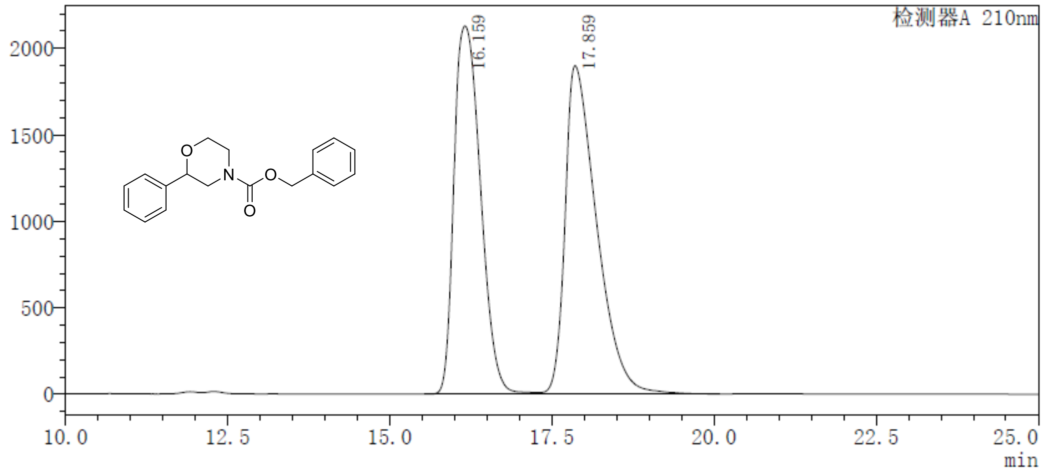

No.	RT	Area	Height	Area%
1	16.159	60298841	2127194	48.346
2	17.859	64424164	1897386	51.654

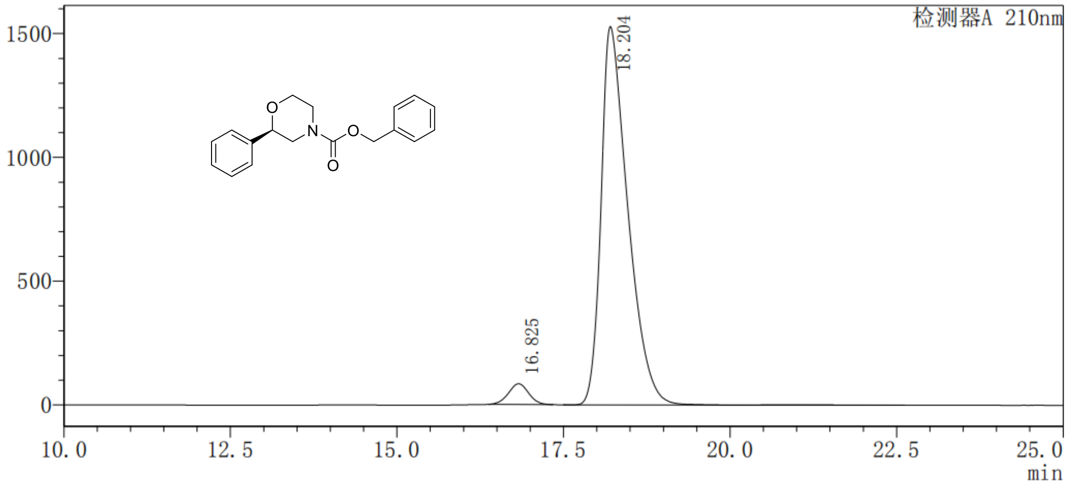

No.	RT	Area	Height	Area%
1	16.825	1771320	83949	3.972
2	18.204	42818762	1528038	96.028

Benzyl (*R*)-2-(4-fluorophenyl)morpholine-4-carboxylate (2b)

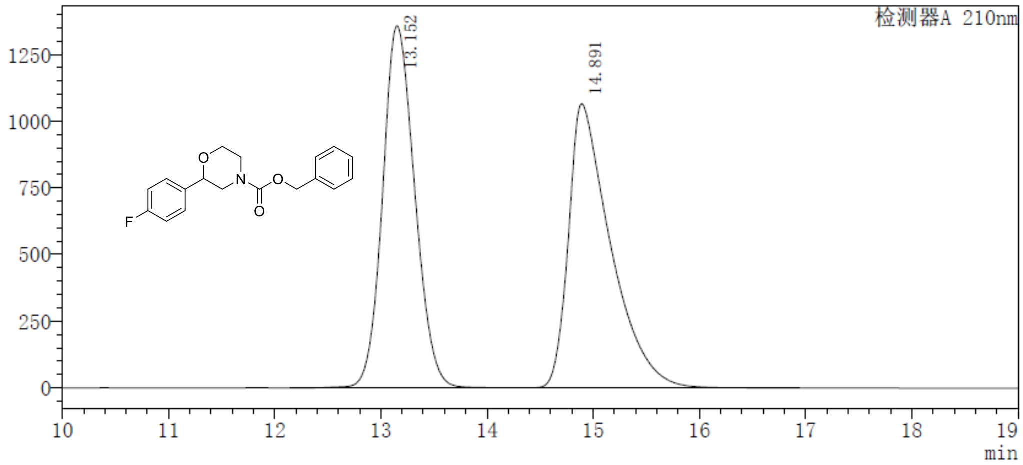

No.	RT	Area	Height	Area%
1	13.152	28712096	1357277	49.408
2	14.891	29400687	1065514	50.592

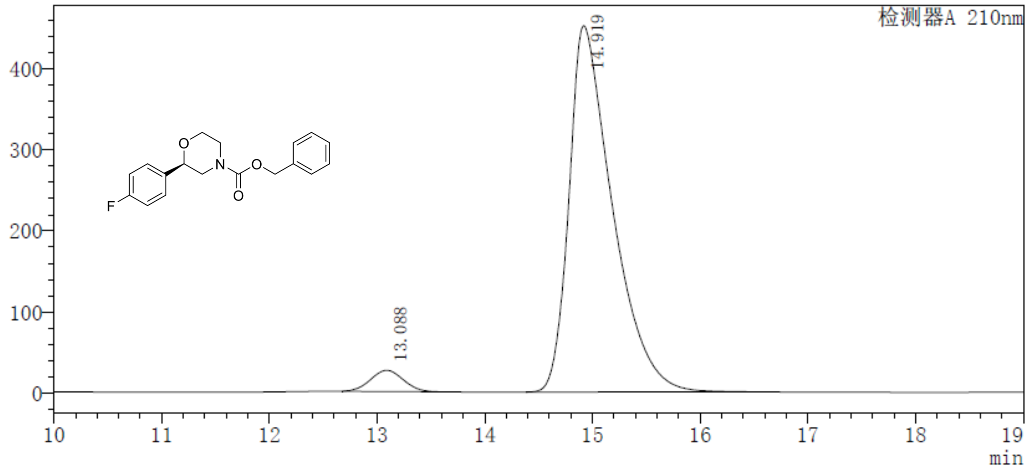

No.	RT	Area	Height	Area%
1	13.088	554668	26237	4.184
2	14.919	12667224	452391	95.543

Benzyl (*R*)-2-(4-chlorophenyl)morpholine-4-carboxylate (2c)

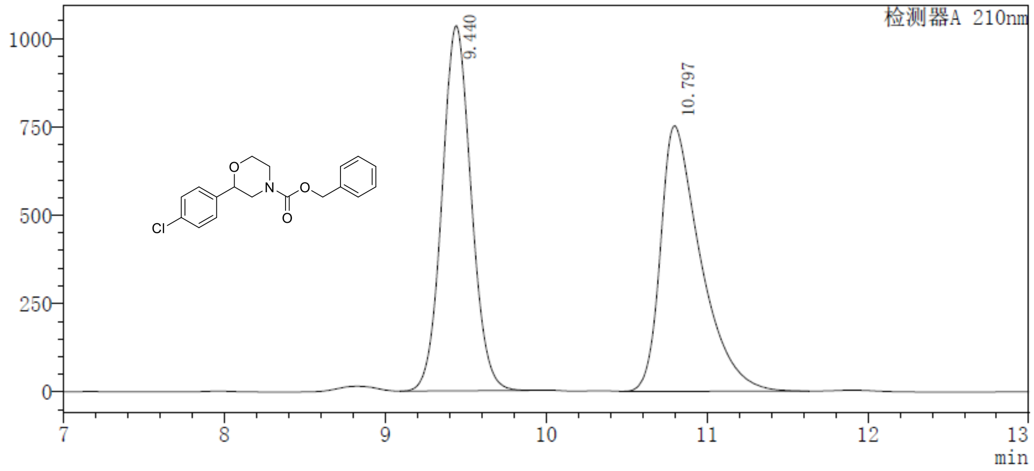

No.	RT	Area	Height	Area%
1	9.440	12943728	1032654	49.841
2	10.797	13026441	750661	50.159

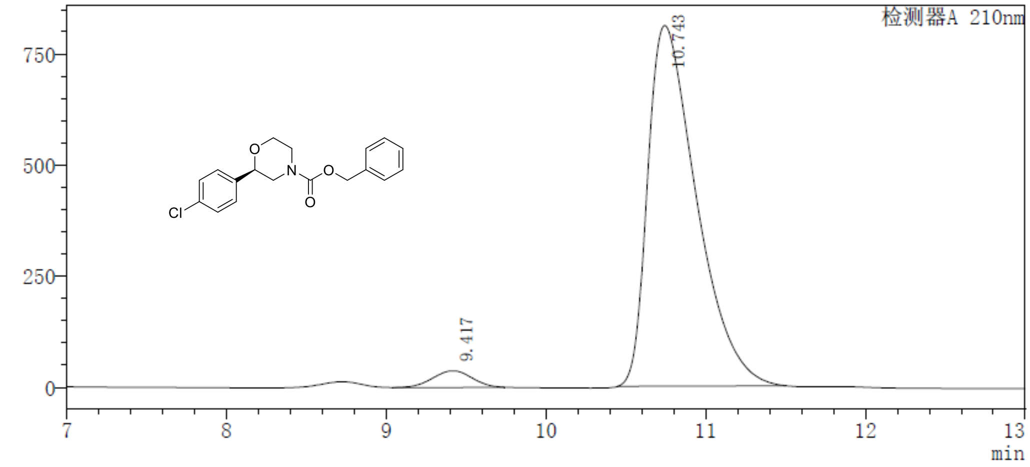

No.	RT	Area	Height	Area%
1	9.417	633264	37231	3.548
2	10.743	17217213	811586	96.452

Benzyl (*R*)-2-(4-bromophenyl)morpholine-4-carboxylate (2d)

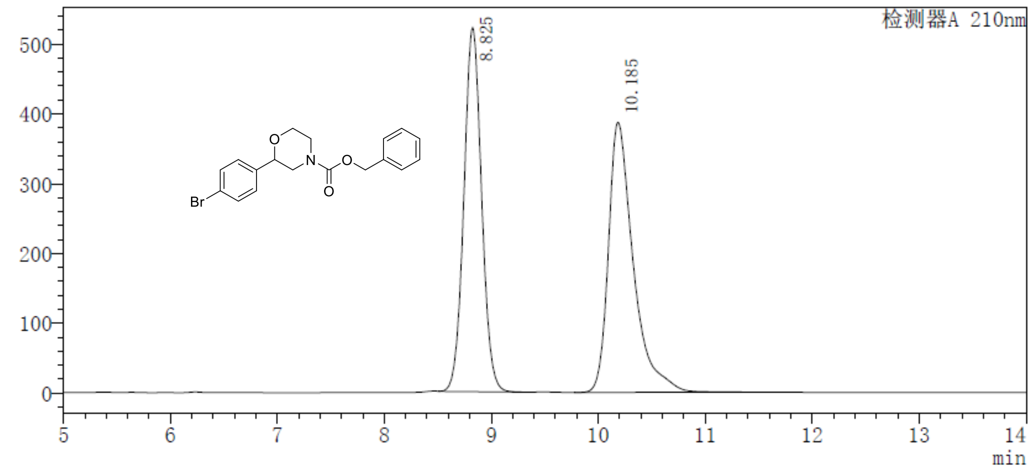

No.	RT	Area	Height	Area%
1	8.825	6010546	521515	48.963
2	10.185	6265133	387234	51.037

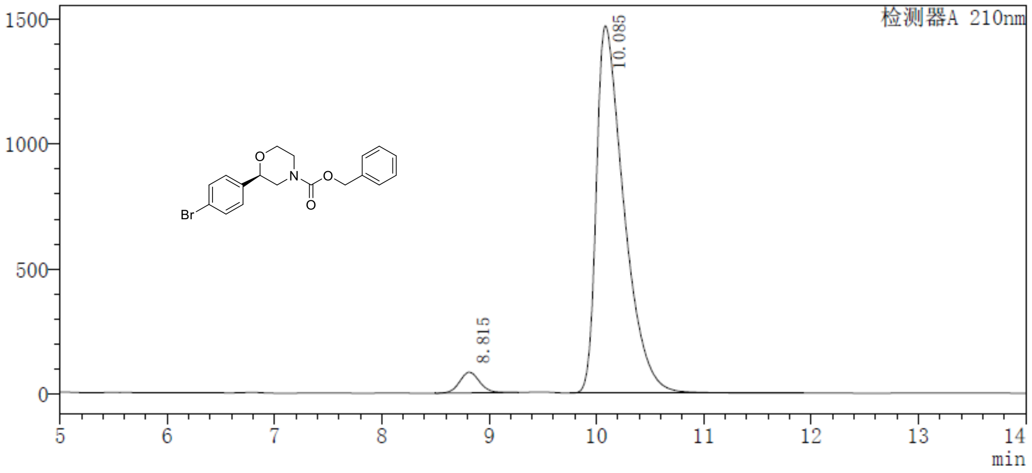

No.	RT	Area	Height	Area%
1	8.815	1065076	82202	3.900
2	10.085	26229814	1466175	96.049

Benzyl (*R*)-2-([1,1'-biphenyl]-4-yl)morpholine-4-carboxylate (2e)

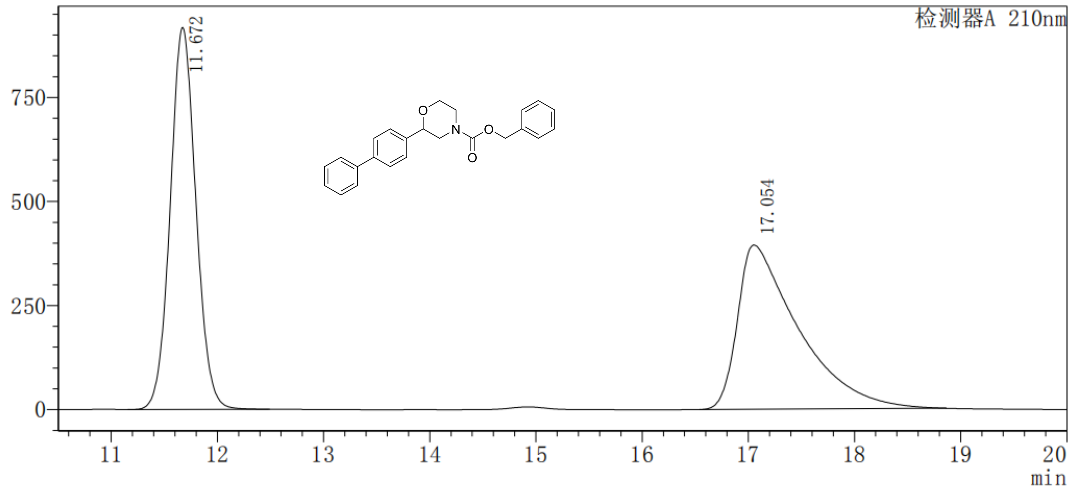

No.	RT	Area	Height	Area%
1	11.672	15434929	917512	49.029
2	17.054	16046101	395648	50.971

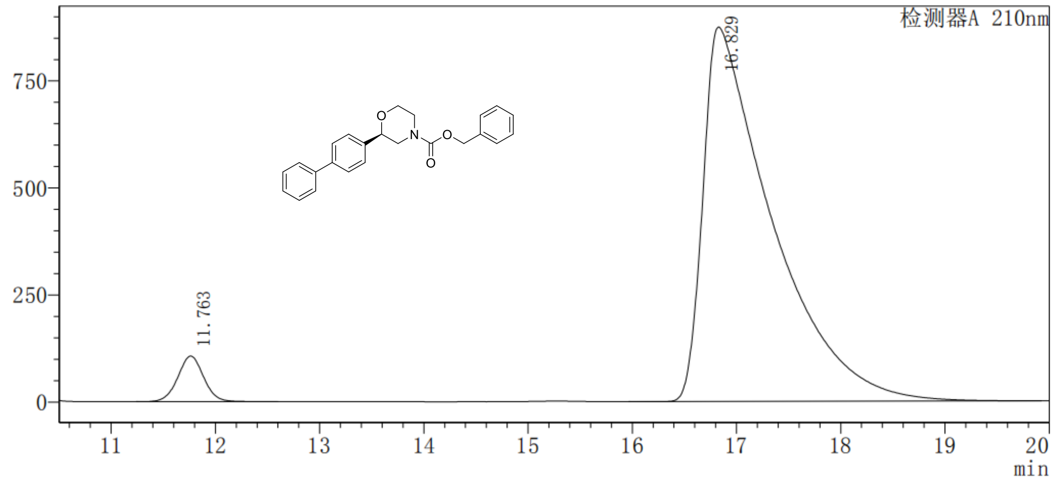

No.	RT	Area	Height	Area%
1	11.763	1837230	106393	4.373
2	16.829	40178564	874173	95.627

Benzyl (*R*)-2-(4-(trifluoromethyl)phenyl)morpholine-4-carboxylate (2f)

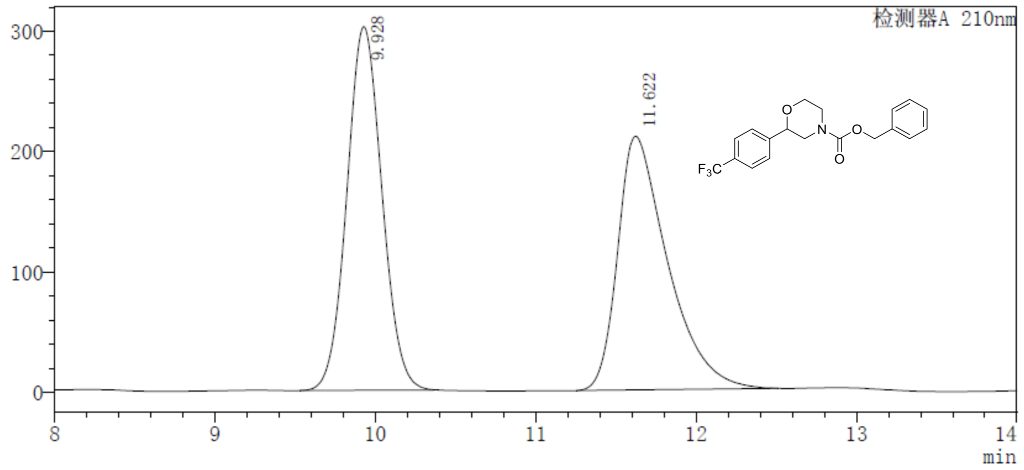

No.	RT	Area	Height	Area%
1	9.928	4603465	301868	50.285
2	11.622	4551327	210610	49.715

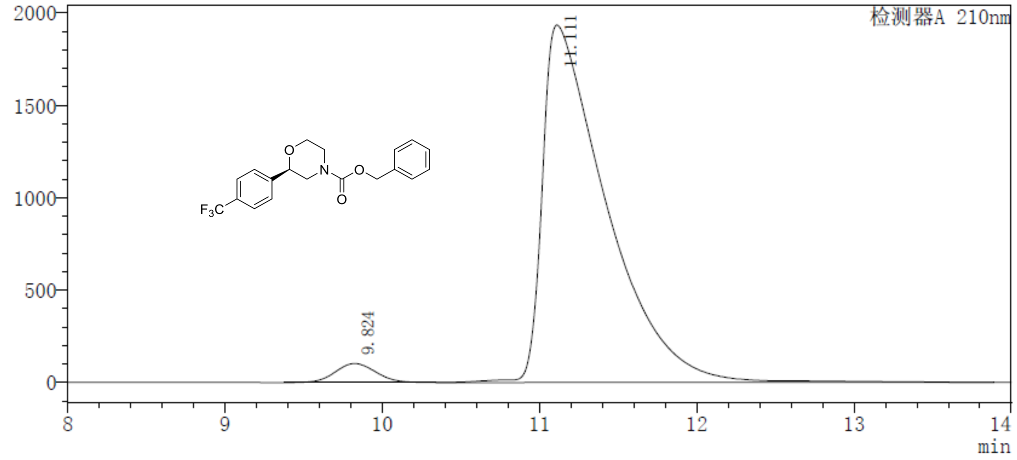

No.	RT	Area	Height	Area%
1	9.824	1833028	102286	3.231
2	11.111	54908138	1934755	96.769

Benzyl (*R*)-2-(*p*-tolyl)morpholine-4-carboxylate (2g)

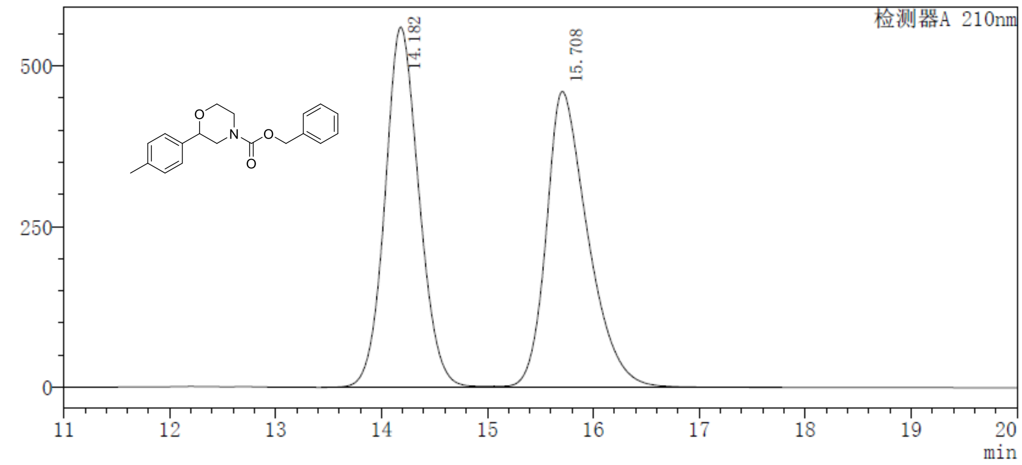

No.	RT	Area	Height	Area%
1	14.182	12564684	561043	49.884
2	15.708	12623284	461038	50.116

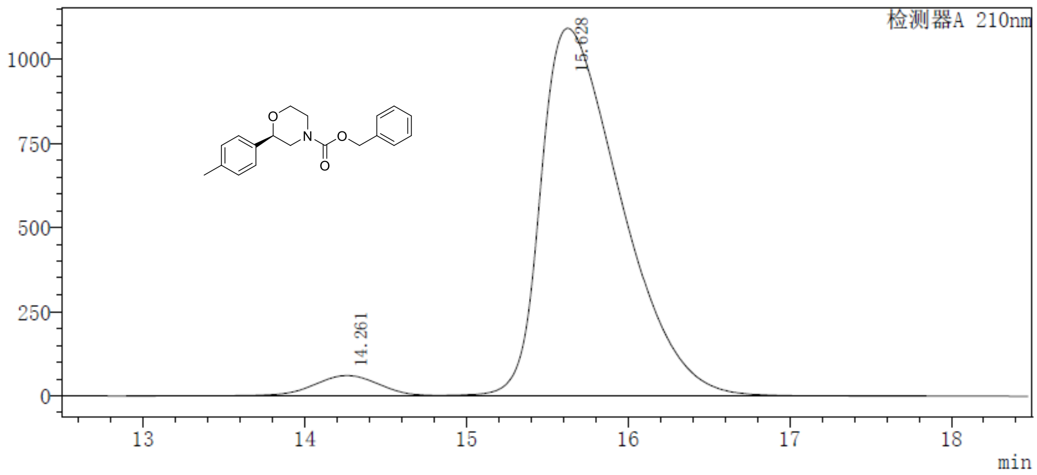

No.	RT	Area	Height	Area%
1	14.261	1743892	60707	4.410
2	15.628	37802579	1093345	95.590

Benzyl (*R*)-2-(4-methoxyphenyl)morpholine-4-carboxylate (2h)

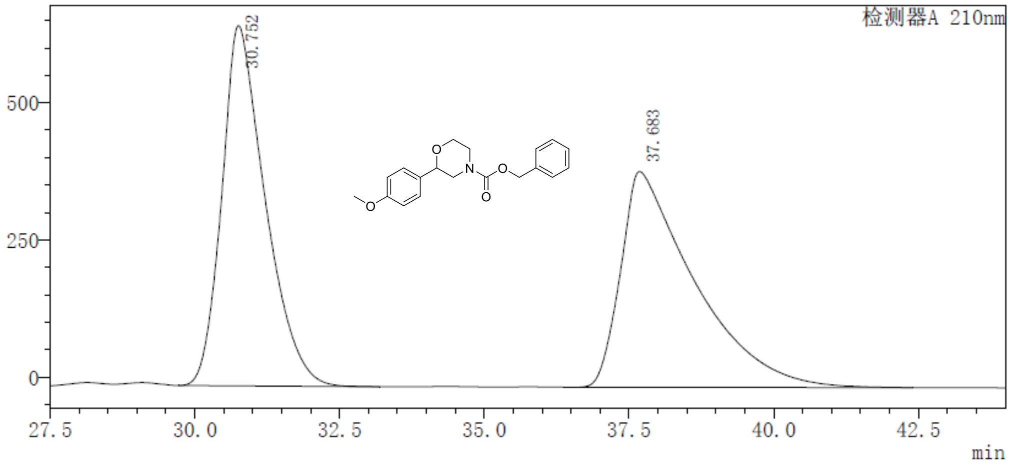

No.	RT	Area	Height	Area%
1	30.752	34334527	657280	49.649
2	37.683	34819644	393710	50.351

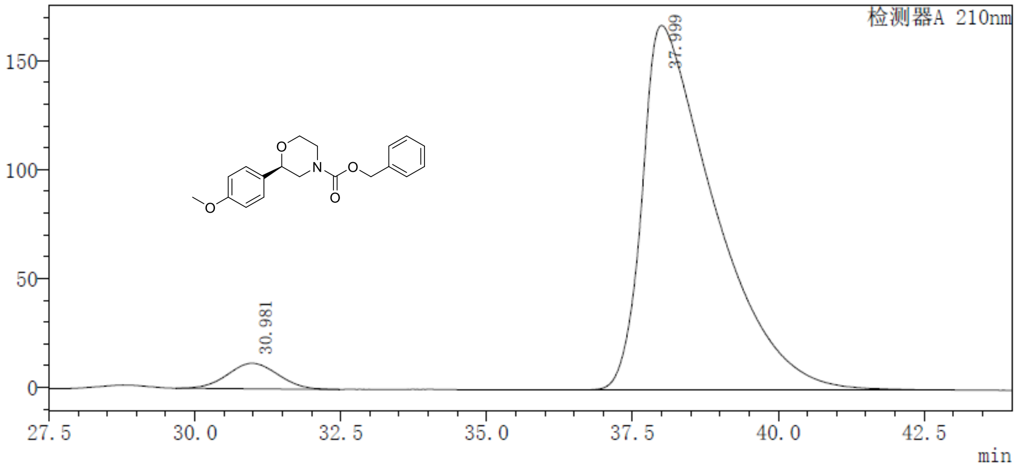

No.	RT	Area	Height	Area%
1	30.981	702800	11689	4.632
2	37.999	14470164	167379	95.368

Benzyl (*R*)-2-(3-fluorophenyl)morpholine-4-carboxylate (2i)

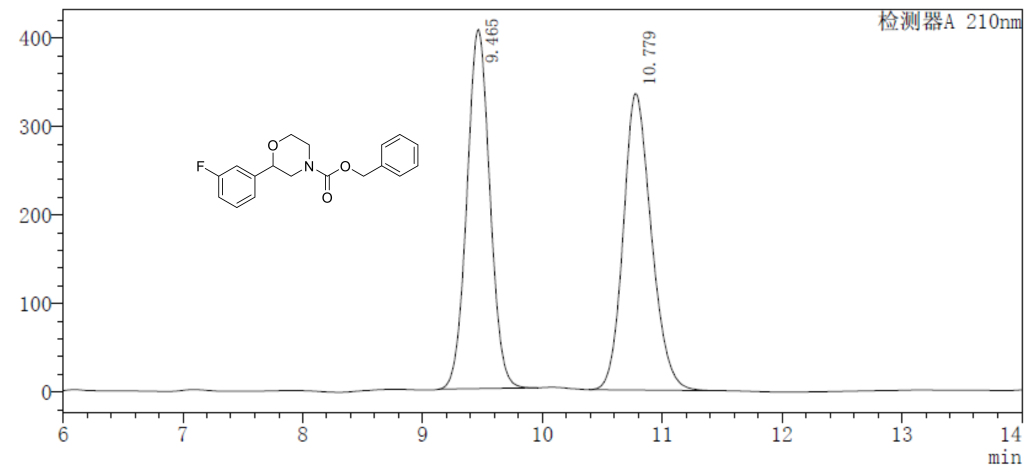

No.	RT	Area	Height	Area%
1	9.465	5422396	405555	49.805
2	10.779	5464962	334734	50.195

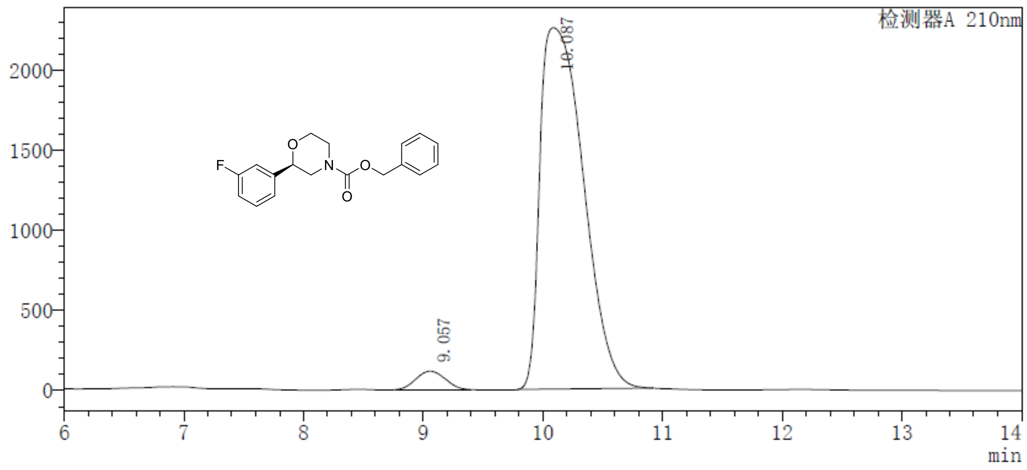

No.	RT	Area	Height	Area%
1	9.057	1939980	114594	3.300
2	10.087	56843266	2255863	96.700

Benzyl (*R*)-2-(3-chlorophenyl)morpholine-4-carboxylate (2j)

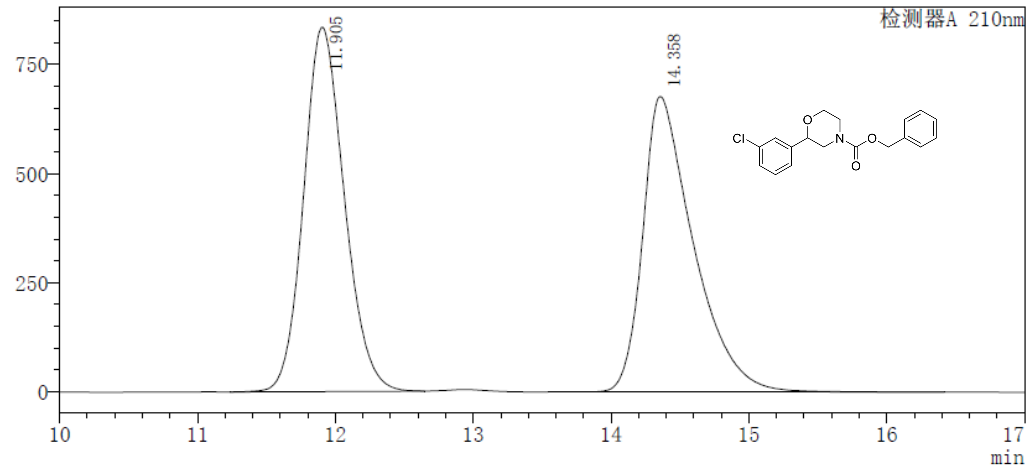

No.	RT	Area	Height	Area%
1	11.905	17139525	833888	49.847
2	14.358	17244537	676444	50.153

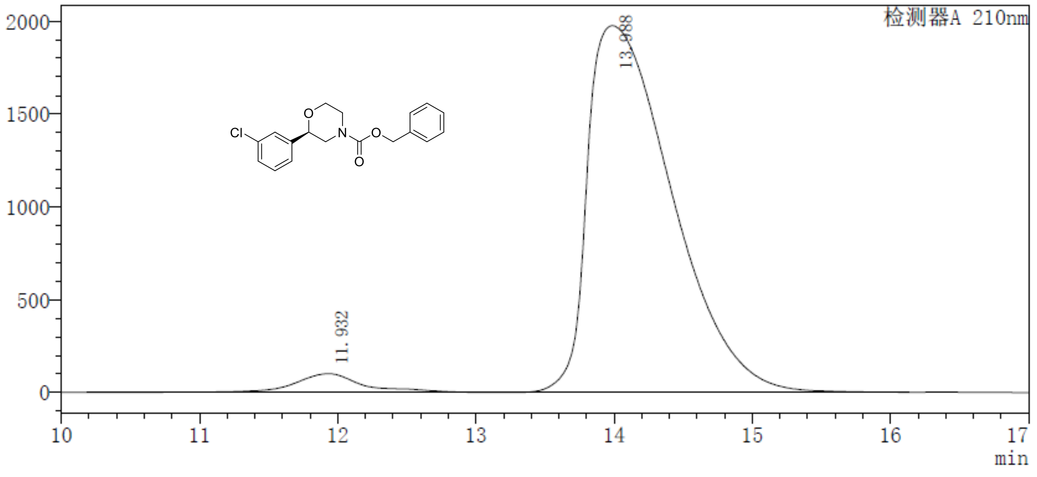

No.	RT	Area	Height	Area%
1	11.932	3518815	100947	4.021
2	13.988	83983651	1973649	95.979

Benzyl (*R*)-2-(3-bromophenyl)morpholine-4-carboxylate (2k)

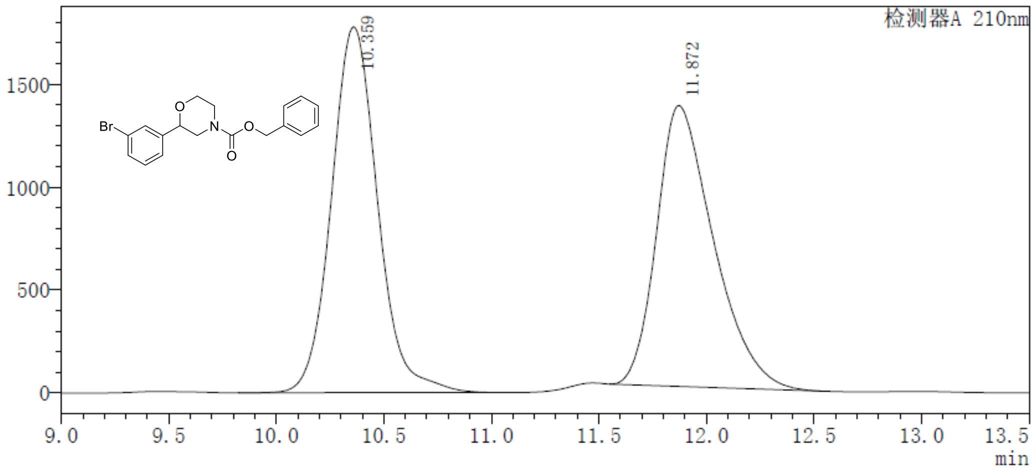

No.	RT	Area	Height	Area%
1	10.359	26581315	1777724	51.338
2	11.872	25195510	1364065	48.662

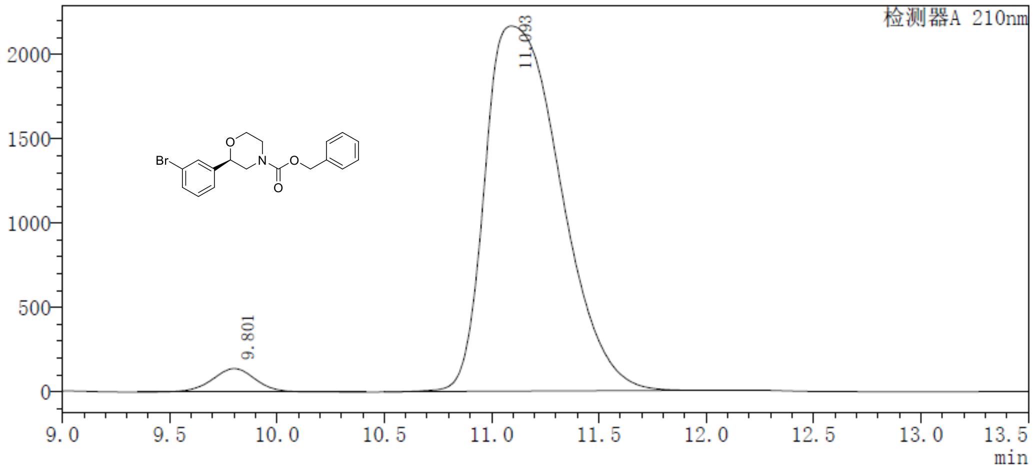

No.	RT	Area	Height	Area%
1	9.801	1935021	138672	3.518
2	11.093	53065641	2166331	96.482

Benzyl (*R*)-2-(3-methoxyphenyl)morpholine-4-carboxylate (2l)

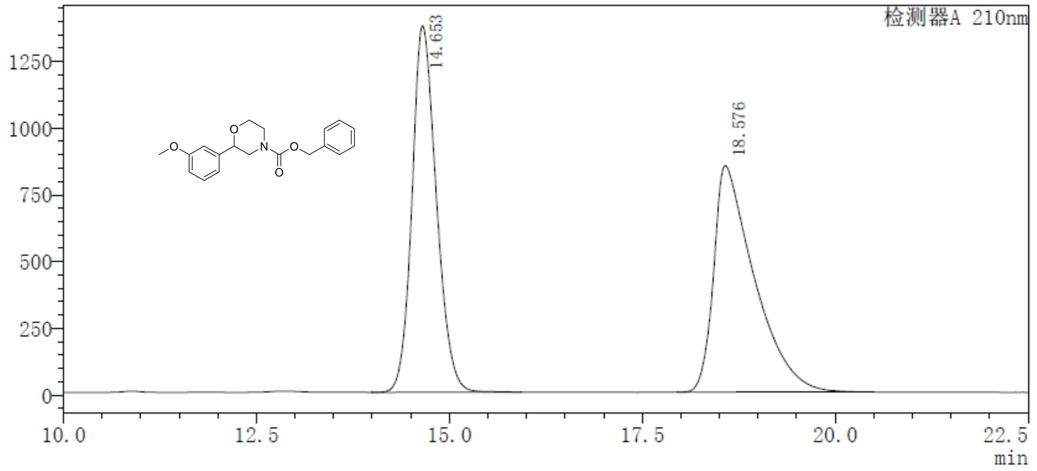

No.	RT	Area	Height	Area%
1	14.653	30215494	1373289	49.103
2	18.576	31319000	849651	50.897

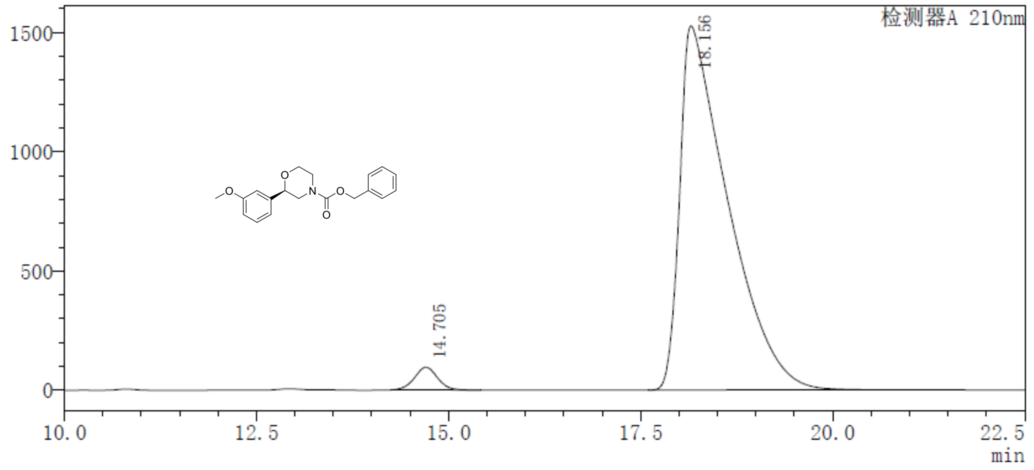

No.	RT	Area	Height	Area%
1	14.705	1975787	94979	2.864
2	18.156	66758314	1526099	96.778

Benzyl (*R*)-2-(2-fluorophenyl)morpholine-4-carboxylate (2m)

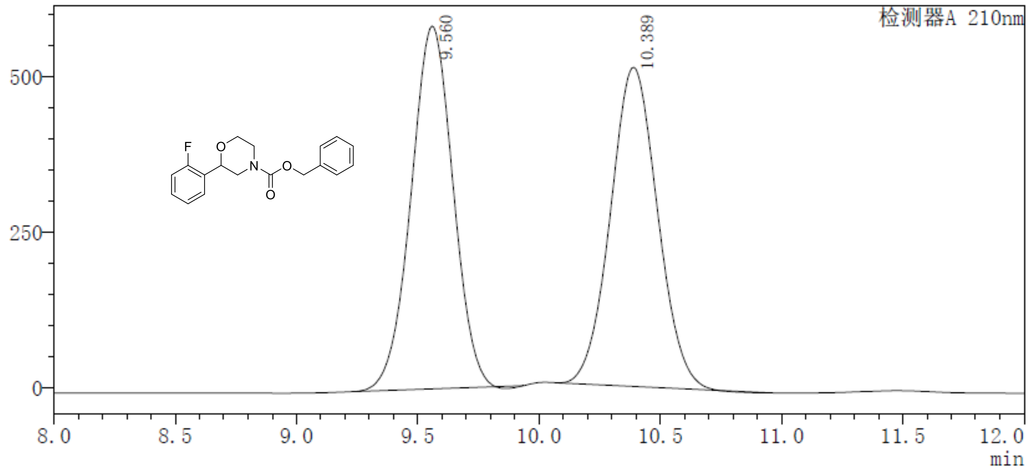

No.	RT	Area	Height	Area%
1	9.560	7472705	1373289	49.103
2	10.389	7173643	849651	50.897

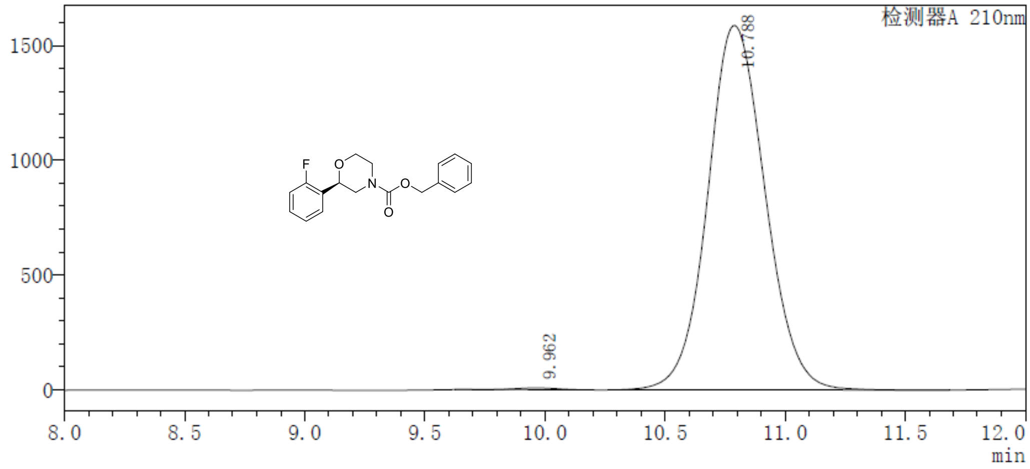

No.	RT	Area	Height	Area%
1	9.962	96546	8396	0.364
2	10.788	26412318	1587657	99.636

Benzyl (*R*)-2-(2-chlorophenyl)morpholine-4-carboxylate (2n)

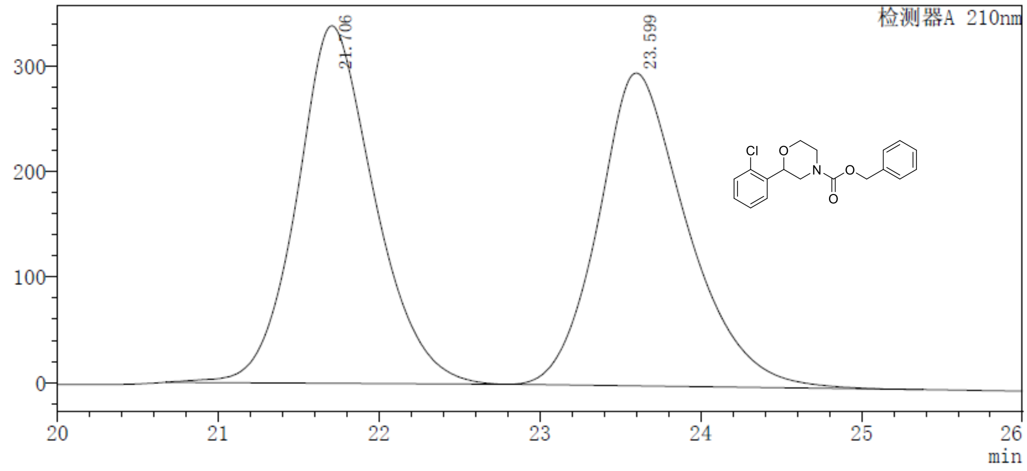

No.	RT	Area	Height	Area%
1	21.706	11423183	338749	49.975
2	23.599	11434524	296541	50.025

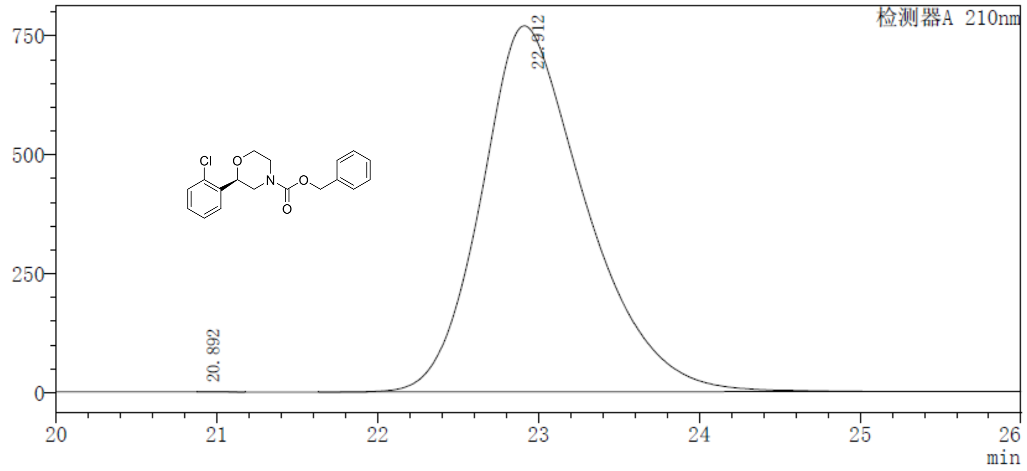

No.	RT	Area	Height	Area%
1	20.892	291	8	0.0001
2	22.912	34872703	769197	99.999

Benzyl (*R*)-2-(*o*-tolyl)morpholine-4-carboxylate (2o)

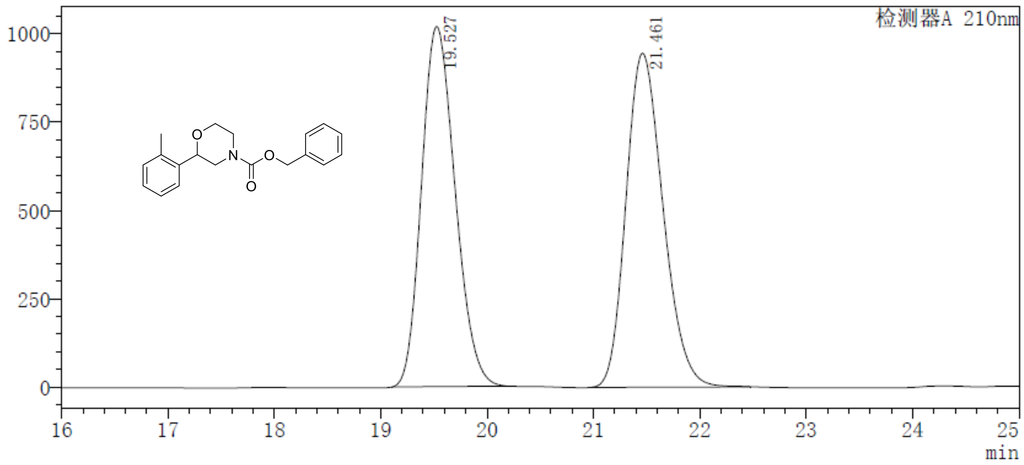

No.	RT	Area	Height	Area%
1	19.527	22373249	1019461	49.420
2	21.461	22898600	945942	50.580

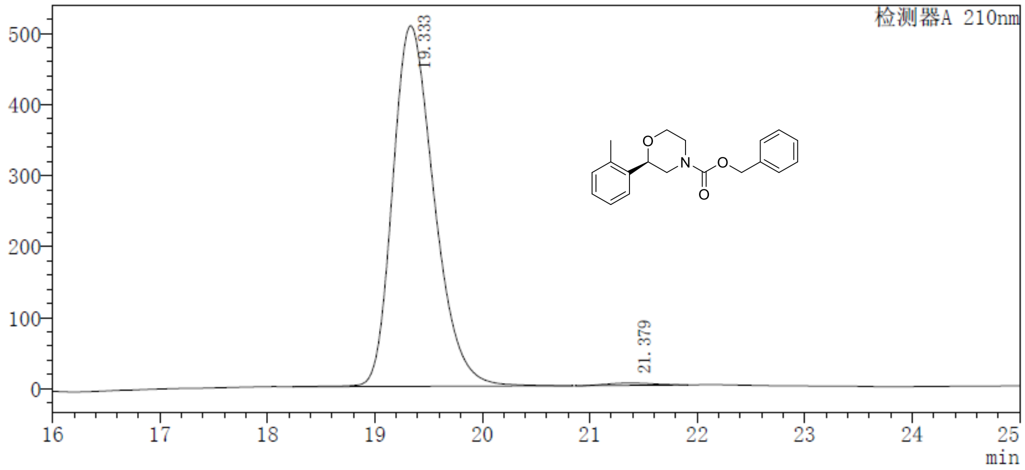

No.	RT	Area	Height	Area%
1	19.333	13611212	507834	99.285
2	21.379	97993	3152	0.715

Benzyl (*R*)-2-(2-methoxyphenyl)morpholine-4-carboxylate (2p)

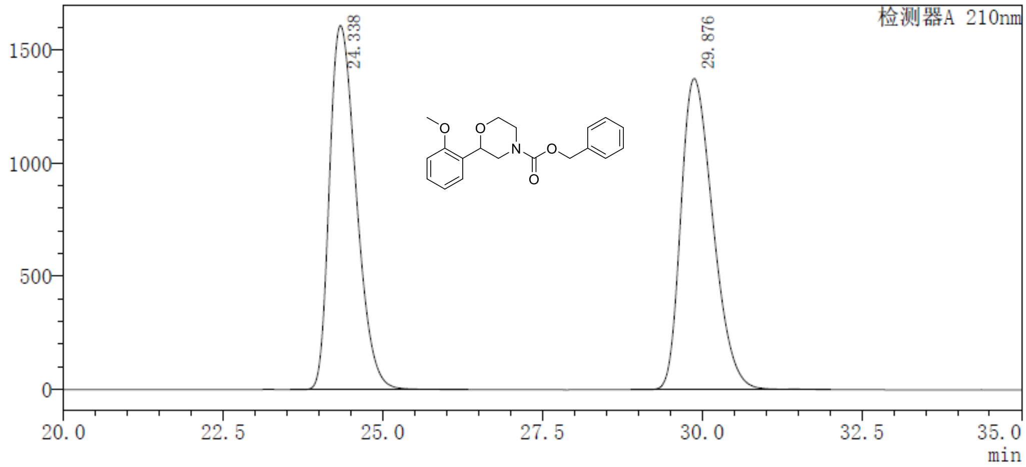

No.	RT	Area	Height	Area%
1	24.338	47038734	1609333	49.417
2	29.876	48149500	1375287	50.583

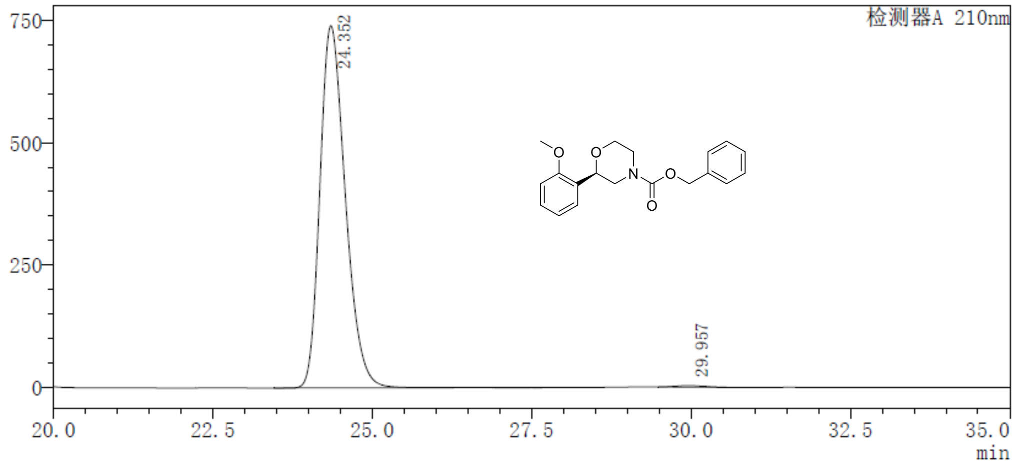

No.	RT	Area	Height	Area%
1	24.352	20569065	740984	99.516
2	29.957	100102	3362	0.484

Benzyl (*R*)-2-(3,4-dichlorophenyl)morpholine-4-carboxylate (2q)

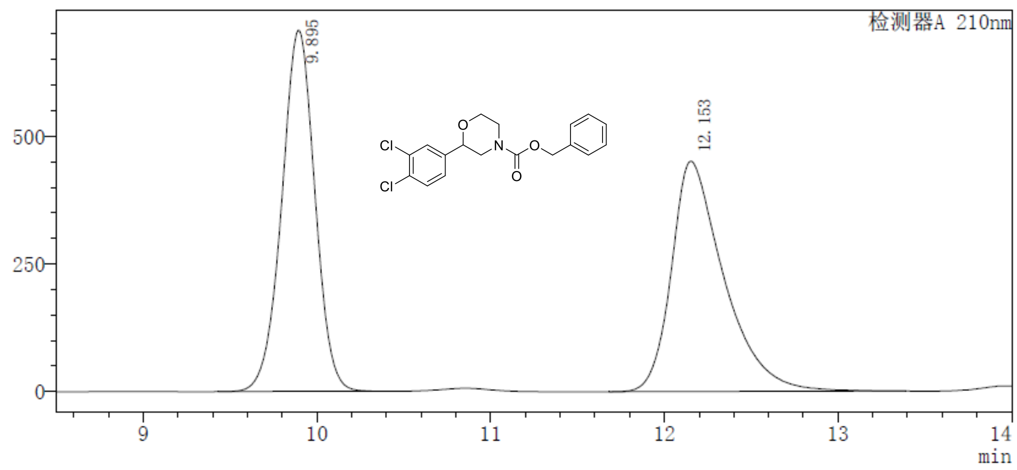

No.	RT	Area	Height	Area%
1	9.895	9399441	706930	49.773
2	12.153	9485227	450738	50.227

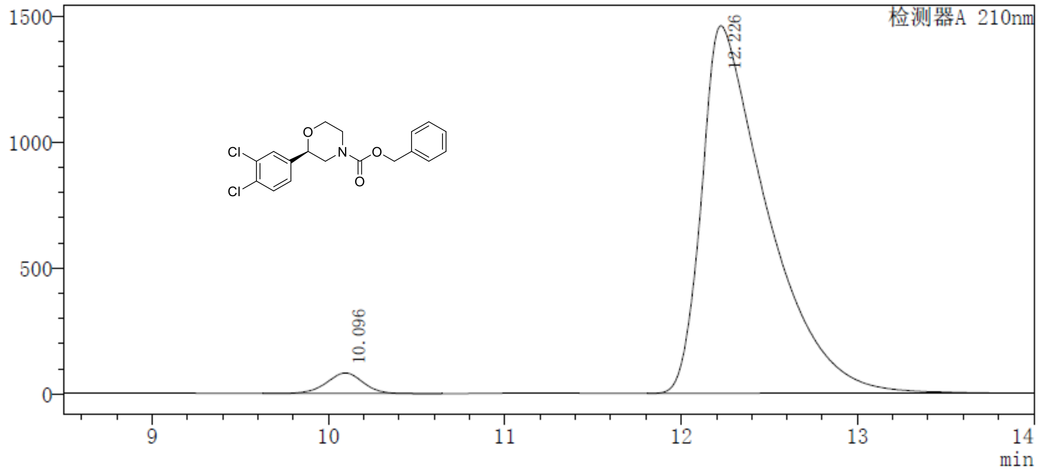

No.	RT	Area	Height	Area%
1	10.096	1163672	81168	2.963
2	12.226	38113113	1460667	97.037

Benzyl (*R*)-2-(3,4-dimethoxyphenyl)morpholine-4-carboxylate (2r)

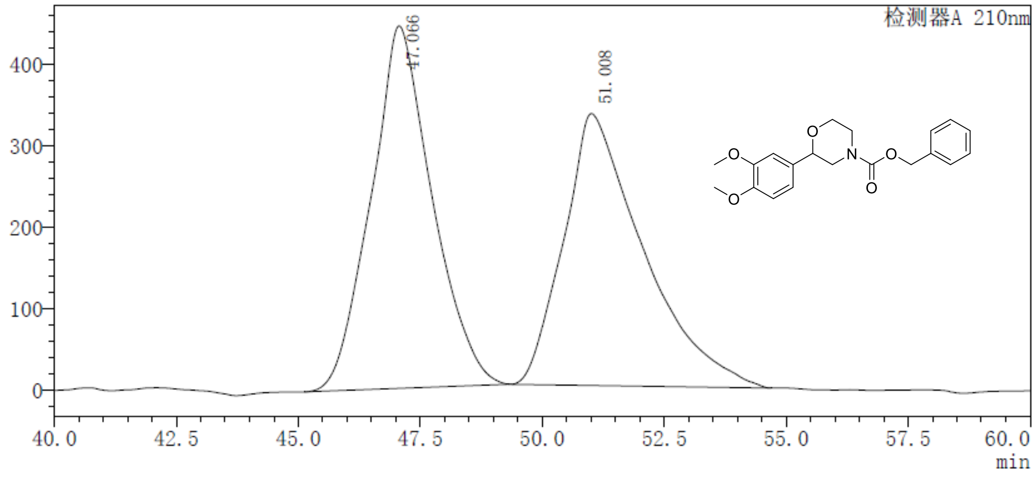

No.	RT	Area	Height	Area%
1	47.066	38858524	445270	50.640
2	51.008	37876688	333882	49.360

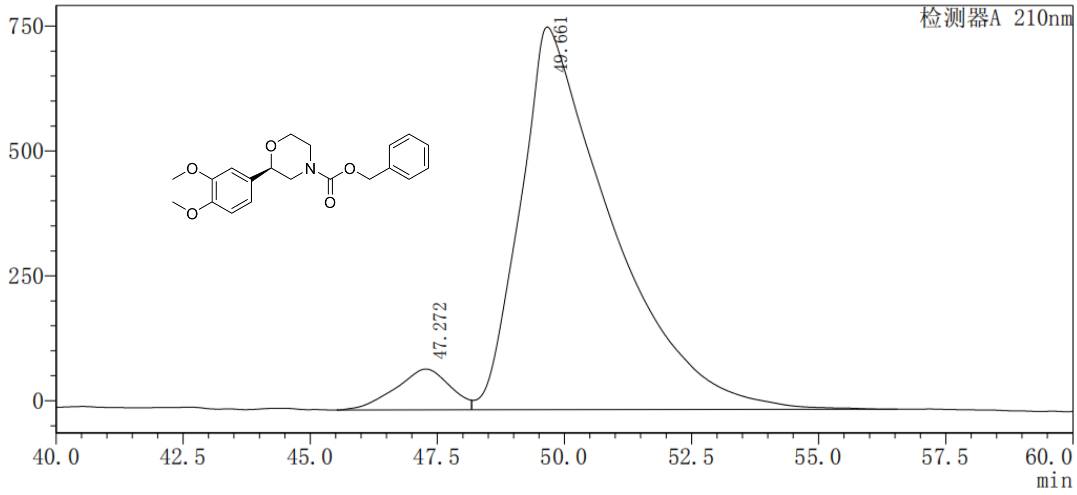

No.	RT	Area	Height	Area%
1	47.272	6121958	81791	6.007
2	49.661	95789599	766188	93.993

Benzyl (*R*)-2-(naphthalen-2-yl)morpholine-4-carboxylate (2s)

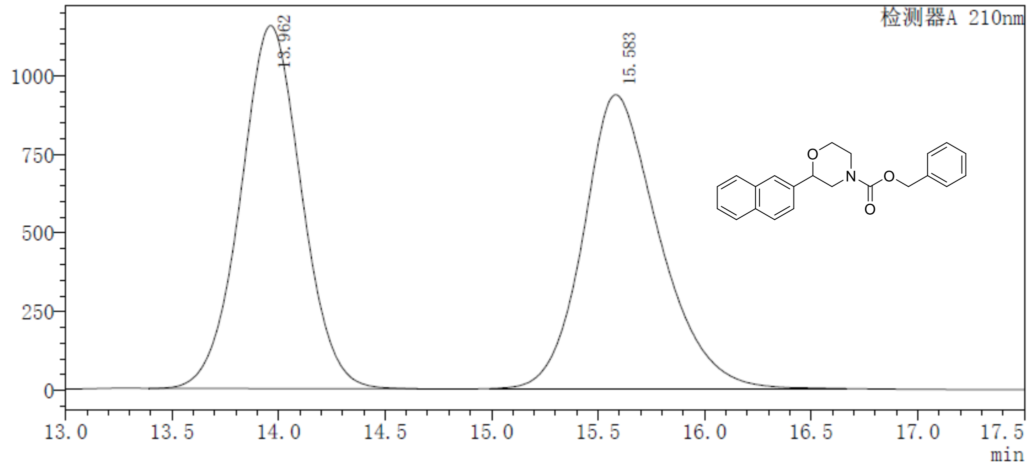

No.	RT	Area	Height	Area%
1	13.962	23002630	1153372	49.806
2	15.583	23182288	934425	50.194

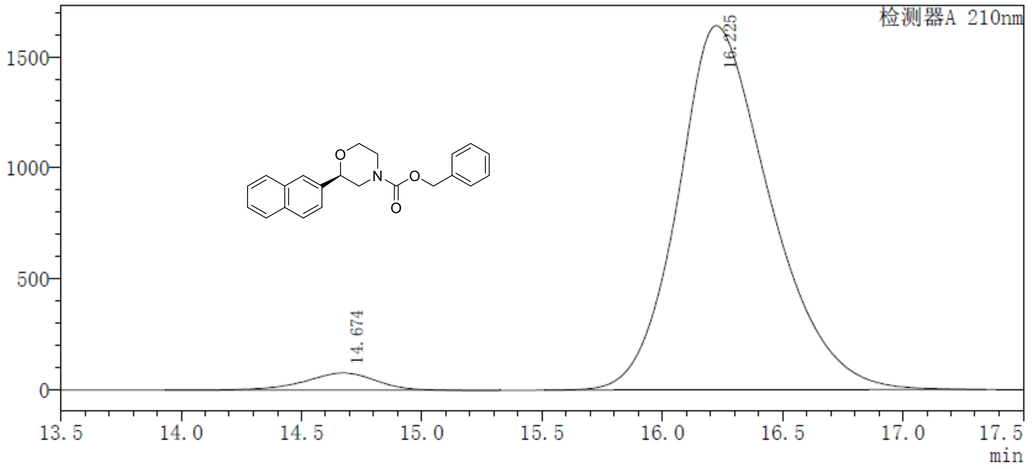

No.	RT	Area	Height	Area%
1	14.674	1608099	77540	3.514
2	16.225	44158977	1643629	96.486

Benzyl (*S*)-2-(thiophen-2-yl)morpholine-4-carboxylate (2t)

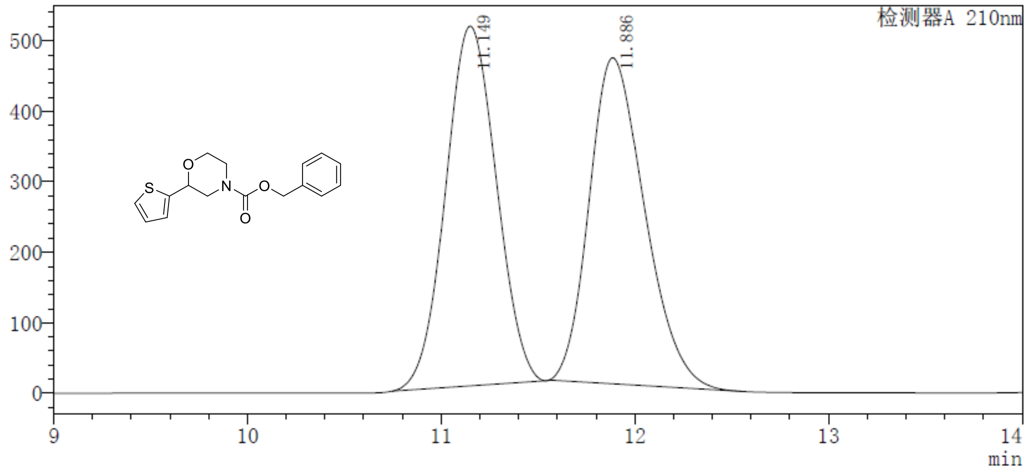

No.	RT	Area	Height	Area%
1	11.149	9294084	510300	50.230
2	11.886	9209018	462400	49.770

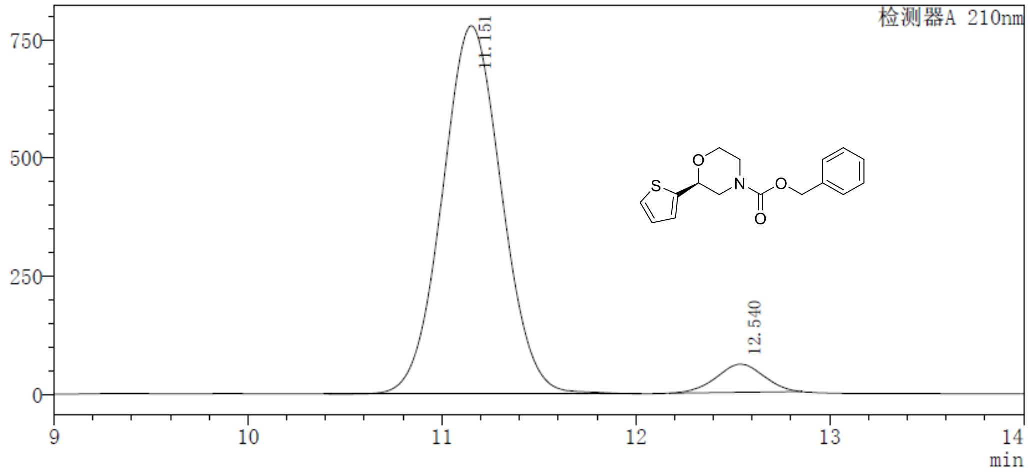

No.	RT	Area	Height	Area%
1	11.151	16531105	778020	94.179
2	12.540	1021779	59663	5.821

Benzyl (*R*)-2-(*tert*-butyl)morpholine-4-carboxylate (2u)

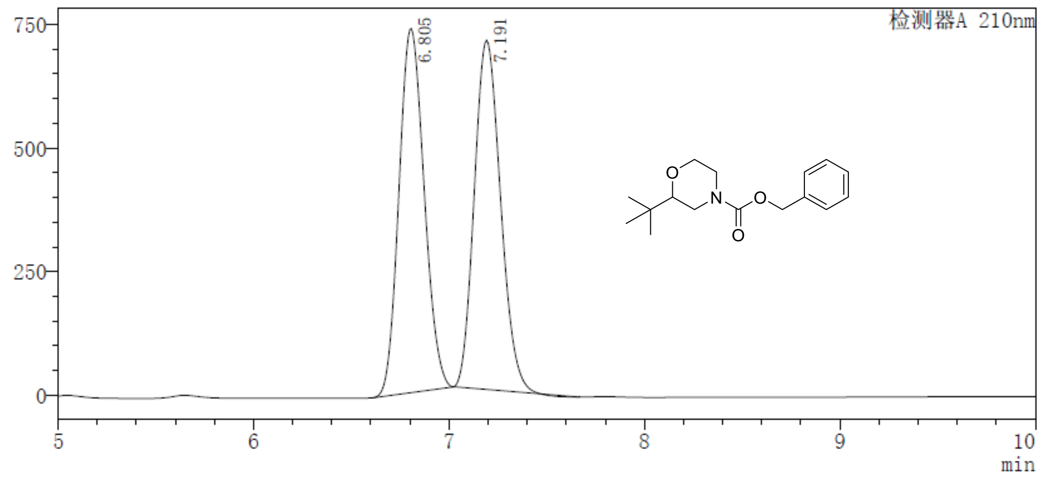

No.	RT	Area	Height	Area%
1	6.805	6593302	735205	50.039
2	7.191	6583030	705704	49.961

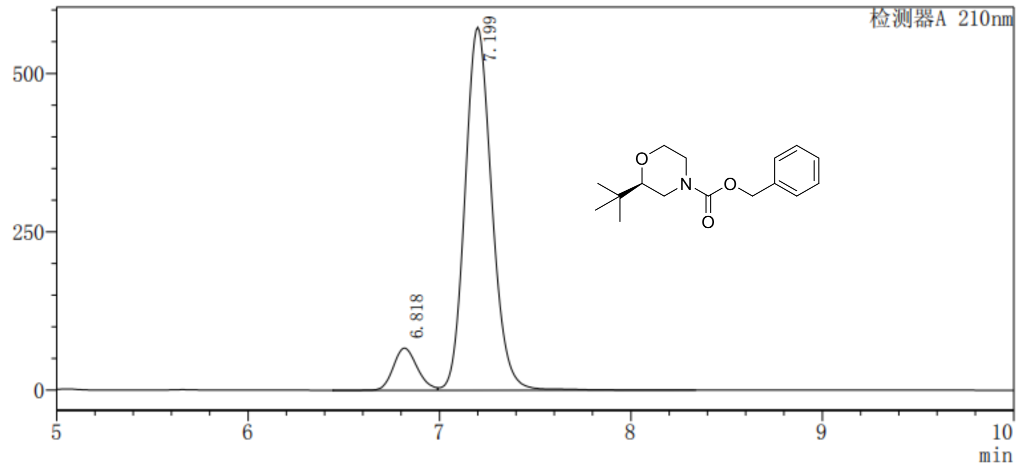

No.	RT	Area	Height	Area%
1	6.818	578631	66704	9.402
2	7.199	5575501	572879	90.598

Benzyl (*R*)-2-isopropylmorpholine-4-carboxylate (2v)

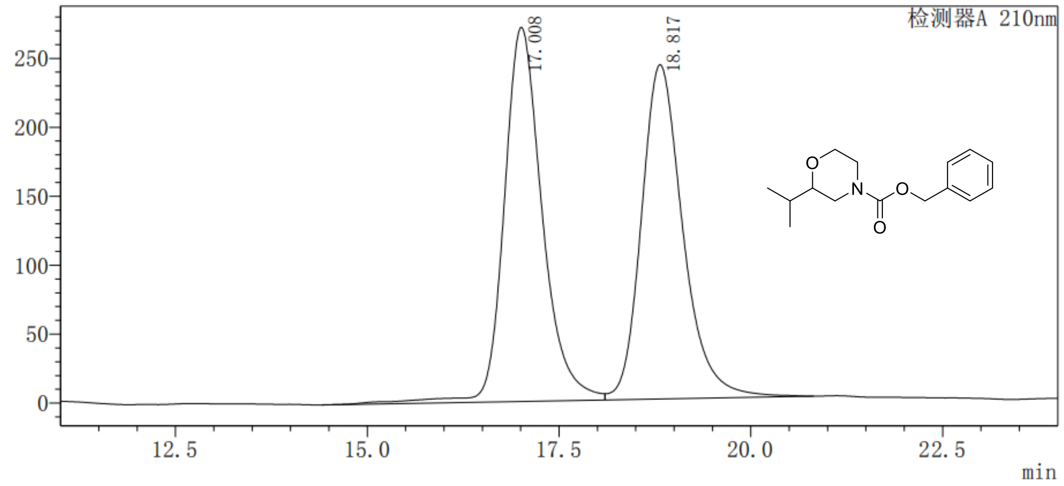

No.	RT	Area	Height	Area%
1	17.008	9401845	271274	50.709
2	18.817	9138997	242452	49.291

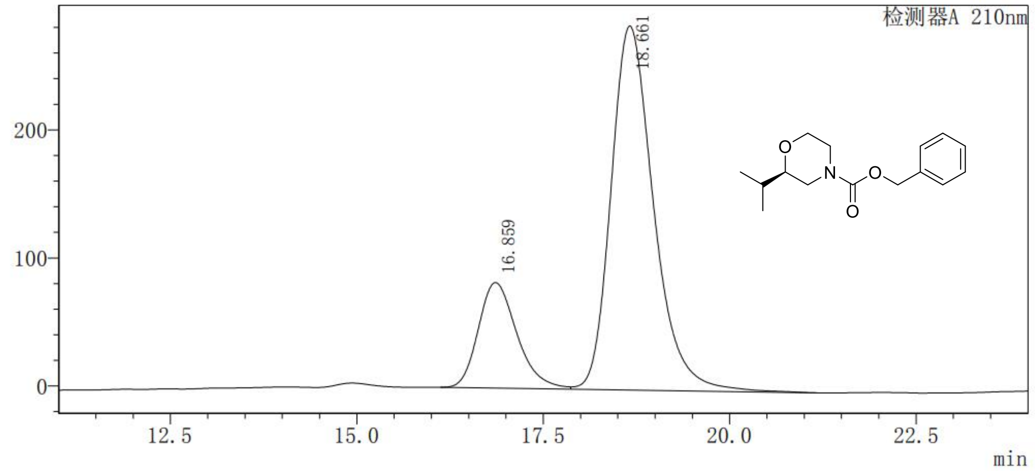

No.	RT	Area	Height	Area%
1	16.859	2962759	82420	20.768
2	18.661	11303564	284366	79.232

Benzyl (*R*)-2-ethylmorpholine-4-carboxylate (2w)

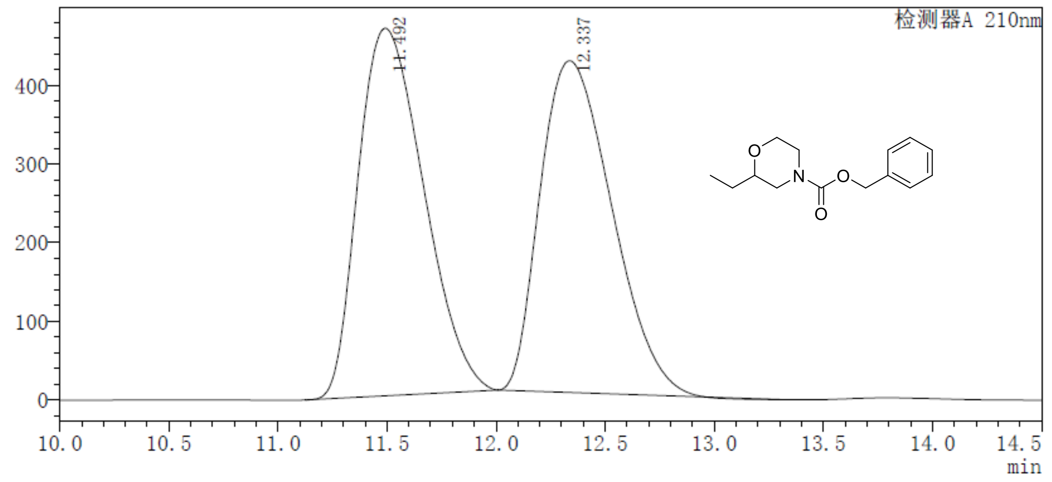

No.	RT	Area	Height	Area%
1	11.492	9772753	467601	50.271
2	12.337	9667395	421909	49.729

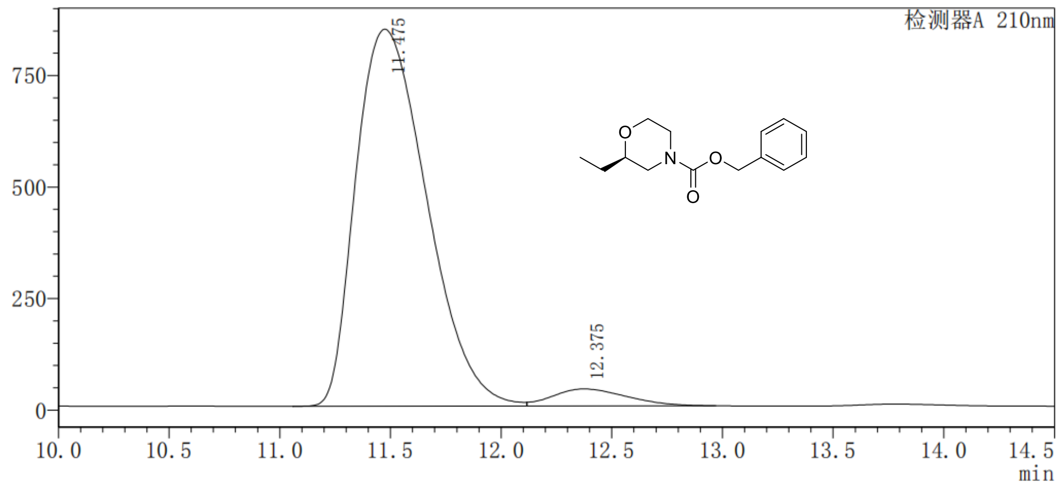

No.	RT	Area	Height	Area%
1	11.475	18850050	845117	95.530
2	12.375	881972	38084	4.470

Benzyl 3-phenylmorpholine-4-carboxylate (2x)

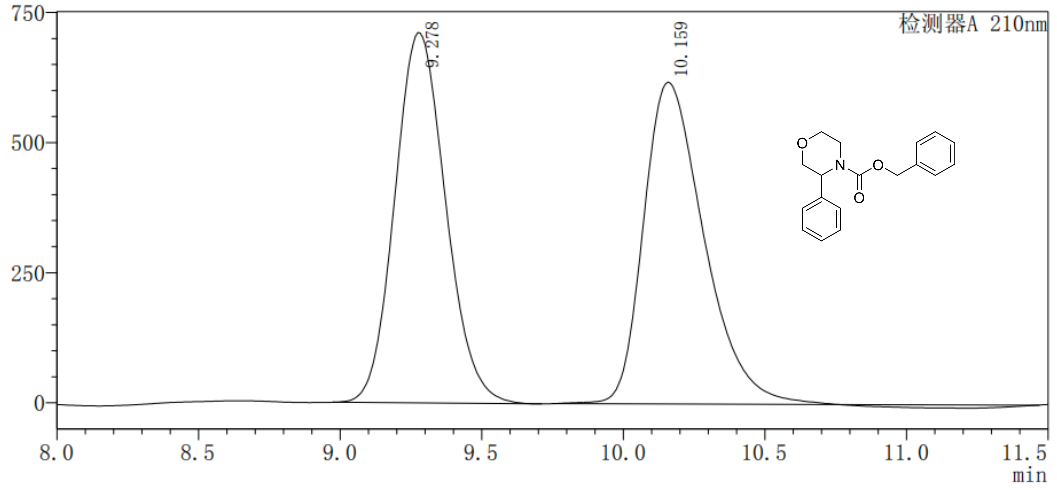

No.	RT	Area	Height	Area%
1	9.278	8902764	711548	49.679
2	10.159	9017648	618384	50.321

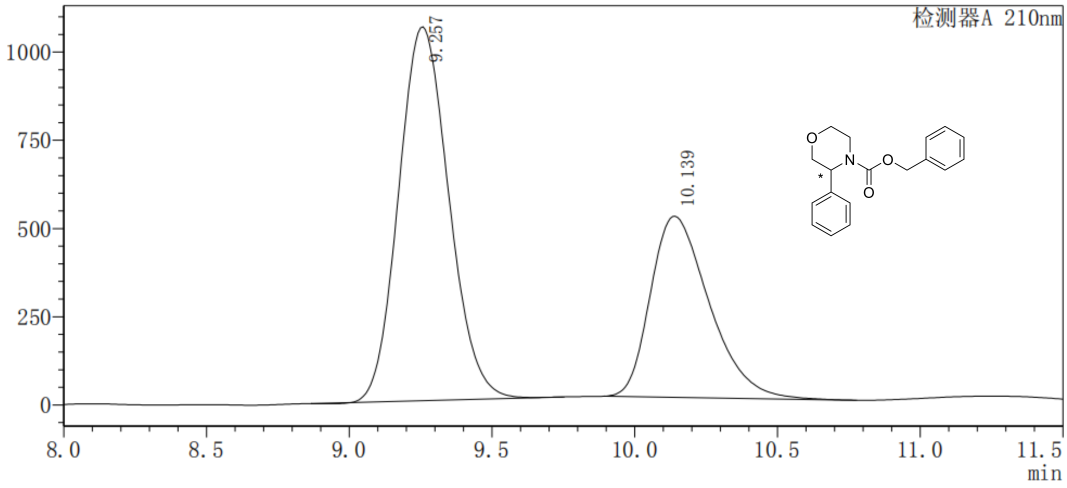

No.	RT	Area	Height	Area%
1	9.257	12943561	1059205	63.764
2	10.139	7355541	513096	36.236

4-Nitrobenzyl (*R*)-2-phenylmorpholine-4-carboxylate (2a-NO_2)

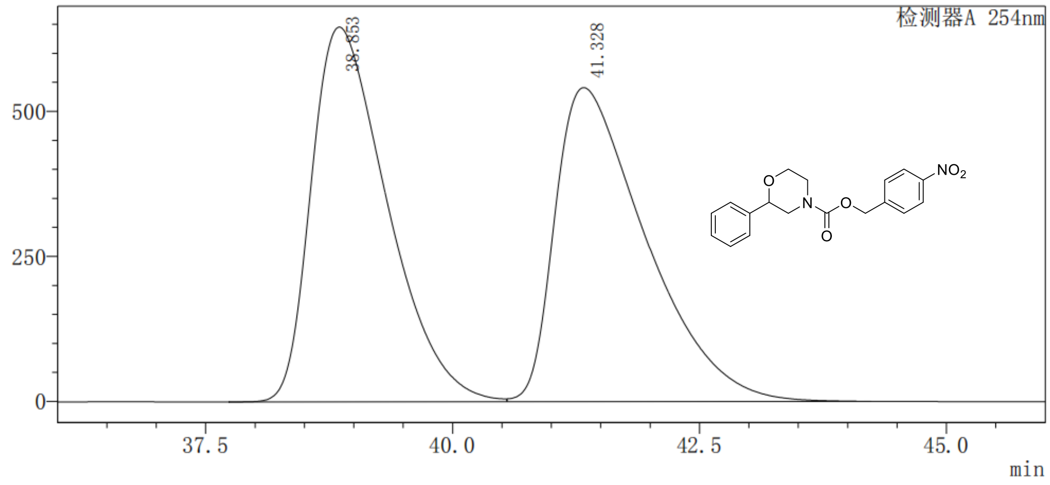

No.	RT	Area	Height	Area%
1	38.853	34612708	645849	49.946
2	41.328	34687027	541219	50.054

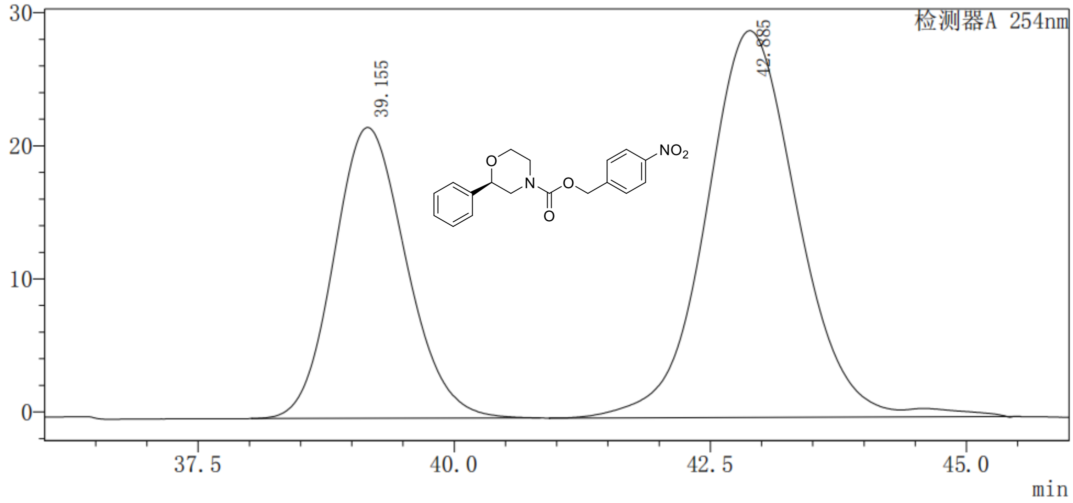

No.	RT	Area	Height	Area%
1	39.155	1076801	21861	37.143
2	42.885	1822232	29067	62.857

Isobutyl (*R*)-2-phenylmorpholine-4-carboxylate (2a-COO*i*Bu)

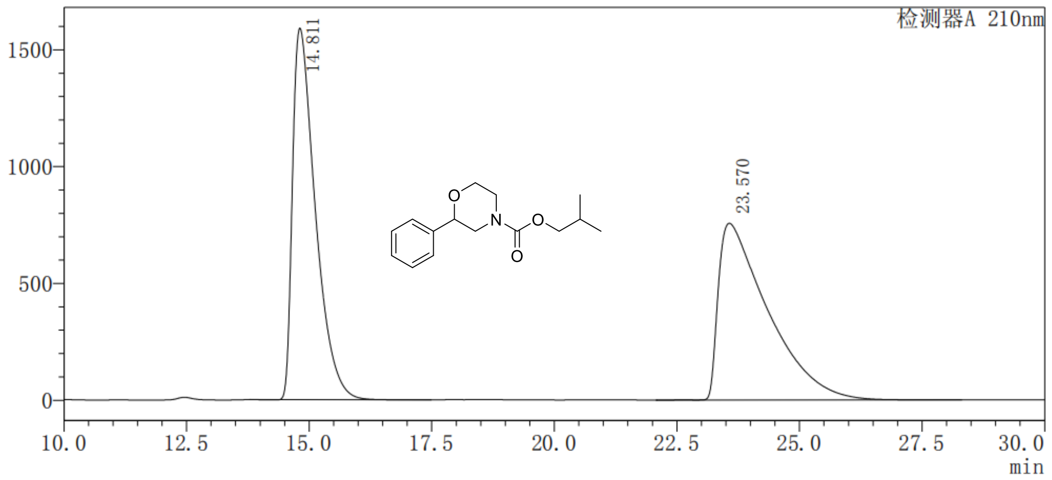

No.	RT	Area	Height	Area%
1	14.811	51611288	1590349	49.259
2	23.570	53163621	756013	50.741

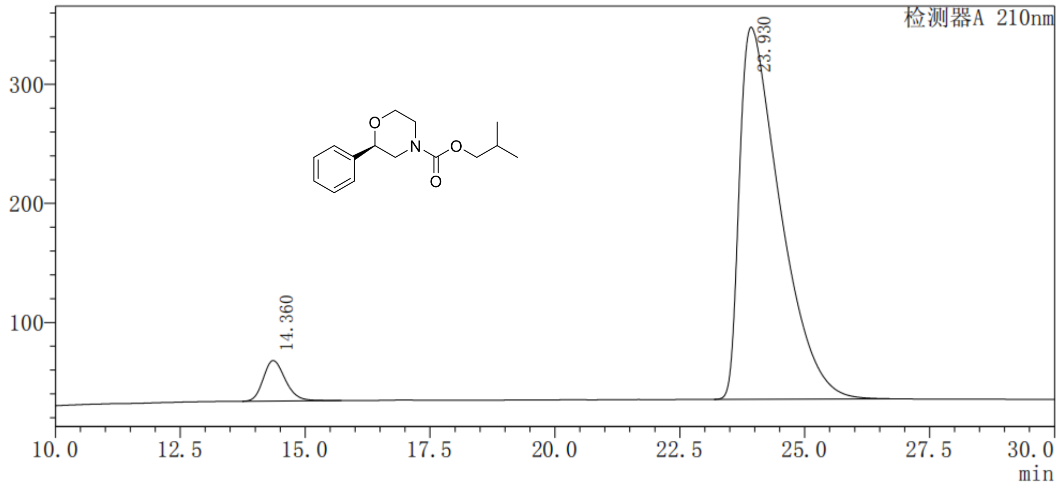

No.	RT	Area	Height	Area%
1	14.360	1047351	34036	5.587
2	23.930	17698495	312596	94.413

tert-Butyl (_R_)-2-phenylmorpholine-4-carboxylate (2a-Boc)

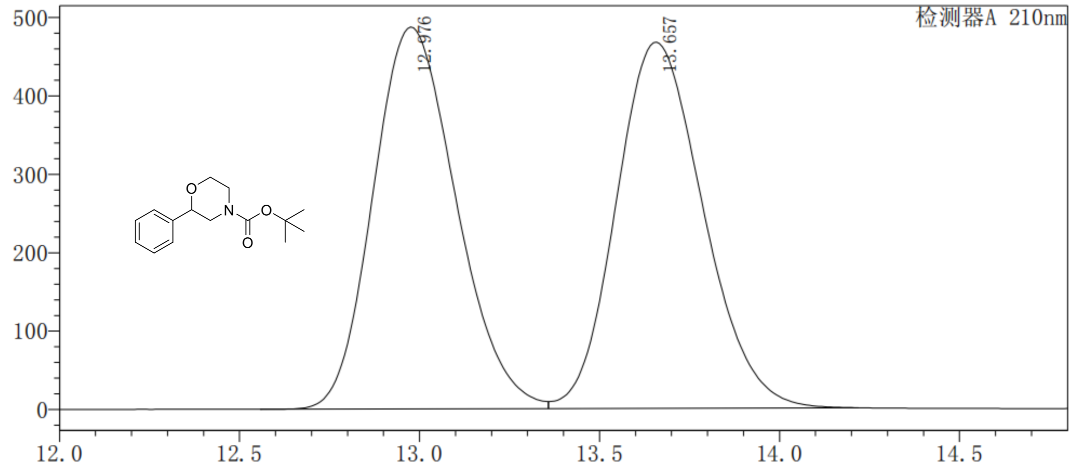

No.	RT	Area	Height	Area%
1	12.976	7813668	487017	49.906
2	13.657	7843029	467112	50.094

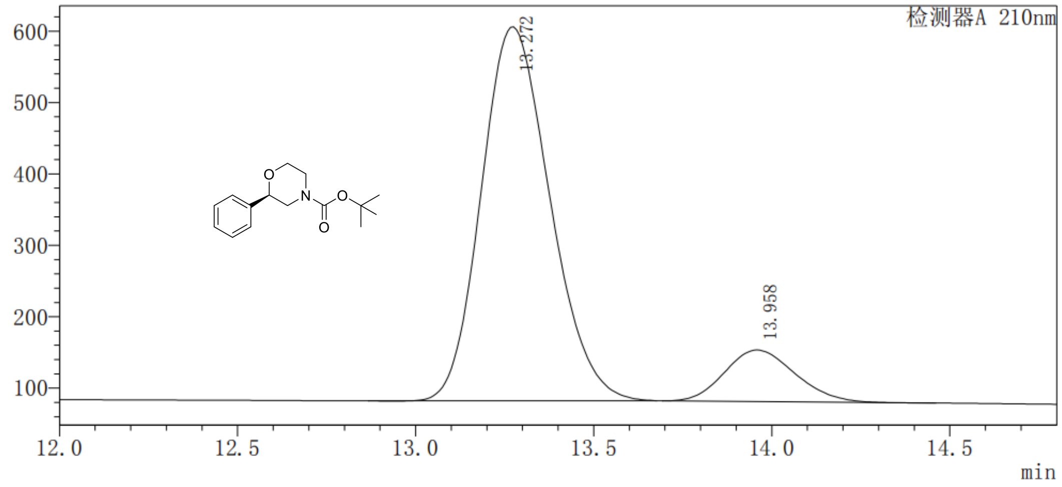

No.	RT	Area	Height	Area%
1	13.272	7007976	523556	87.593
2	13.958	992667	72200	12.407

Benzyl (2*R*)-2,3-d_2-2-phenylmorpholine-4-carboxylate (2a-D)

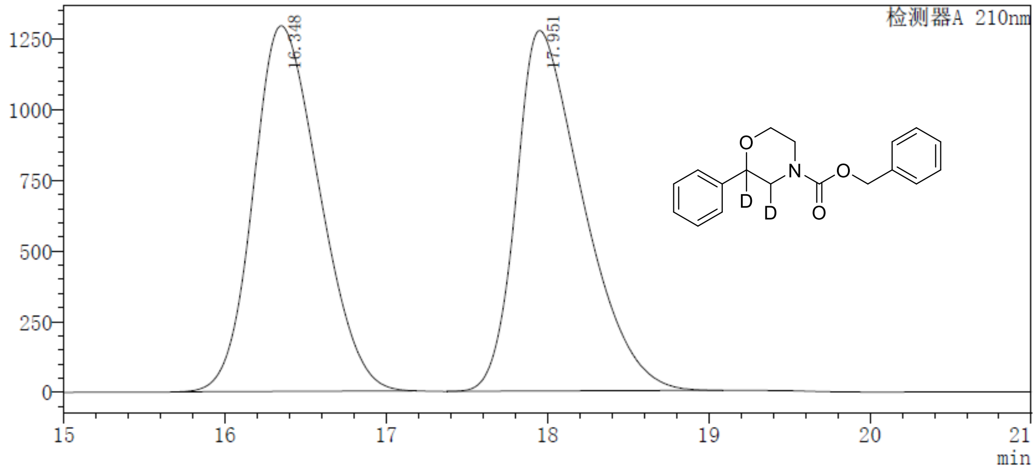

No.	RT	Area	Height	Area%
1	16.348	36790788	1291930	49.955
2	17.951	36856797	1273629	50.045

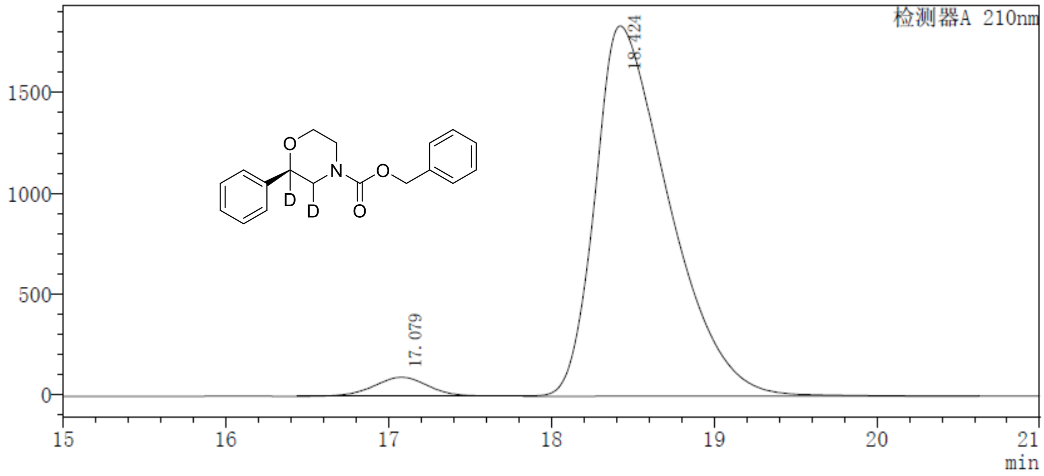

No.	RT	Area	Height	Area%
1	17.079	2211610	94069	3.633
2	18.424	58165541	1841800	96.337

(*R*)-2-(4-fluorophenyl)morpholine (3b) (Determined after acylation by CbzCl)

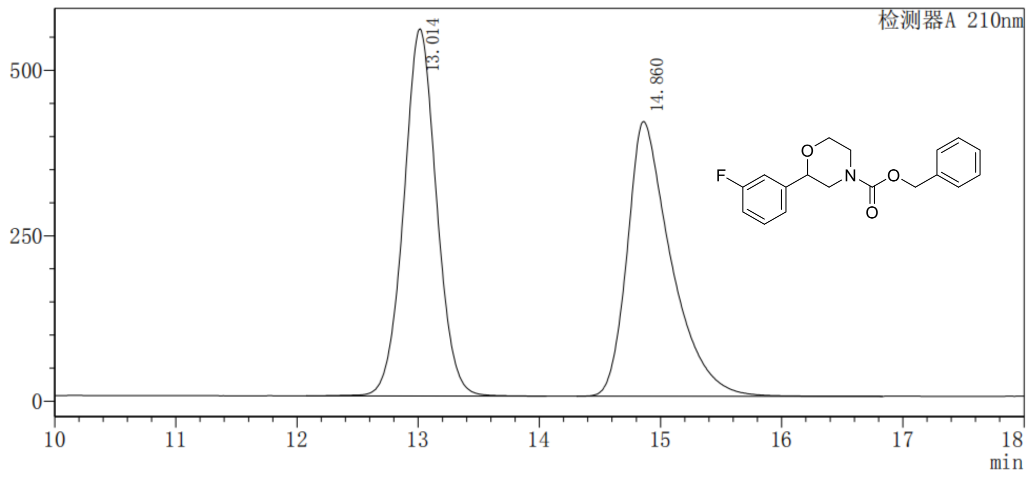

No.	RT	Area	Height	Area%
1	13.014	10427437	554401	49.927
2	14.860	10457835	415102	50.073

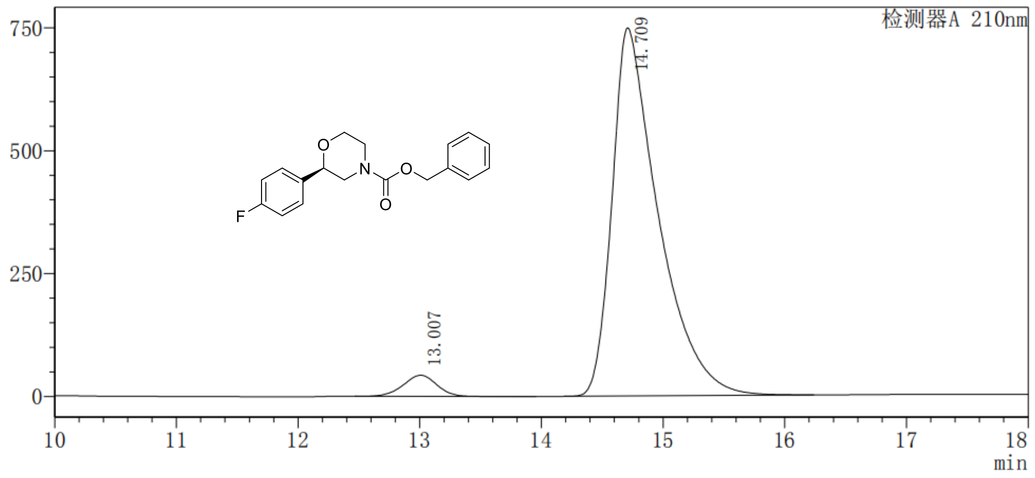

No.	RT	Area	Height	Area%
1	13.007	820225	42913	3.980
2	14.709	19785998	748503	96.020

(*R*)-2-(3-methoxyphenyl)-4-propylmorpholine (3l’)

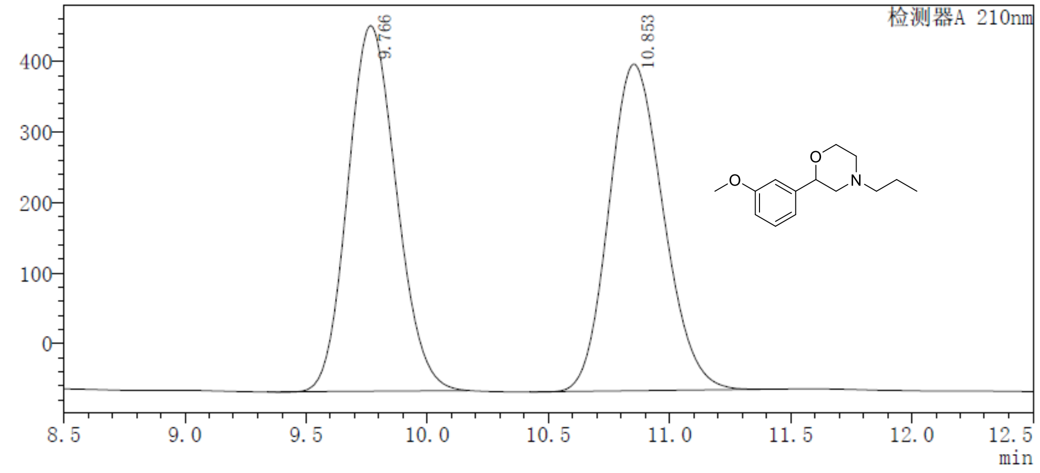

No.	RT	Area	Height	Area%
1	9.766	7253056	518513	49.977
2	10.853	7259685	463041	50.023

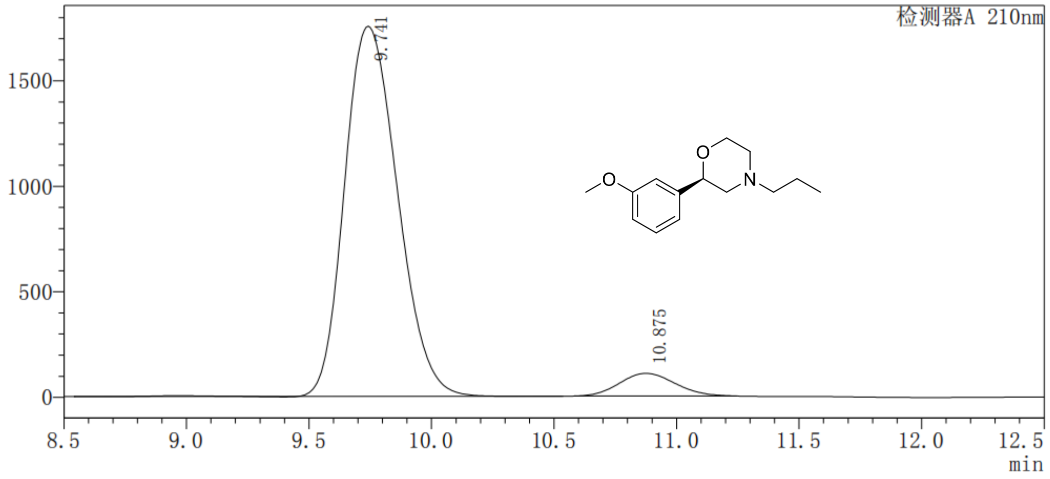

No.	RT	Area	Height	Area%
1	9.741	26390546	1755021	93.919
2	10.875	1708653	108089	6.081

Benzyl (*S*)-2-phenylmorpholine-4-carboxylate (2a) (using (*S*,*S*,*S*)-Tol-SKP)

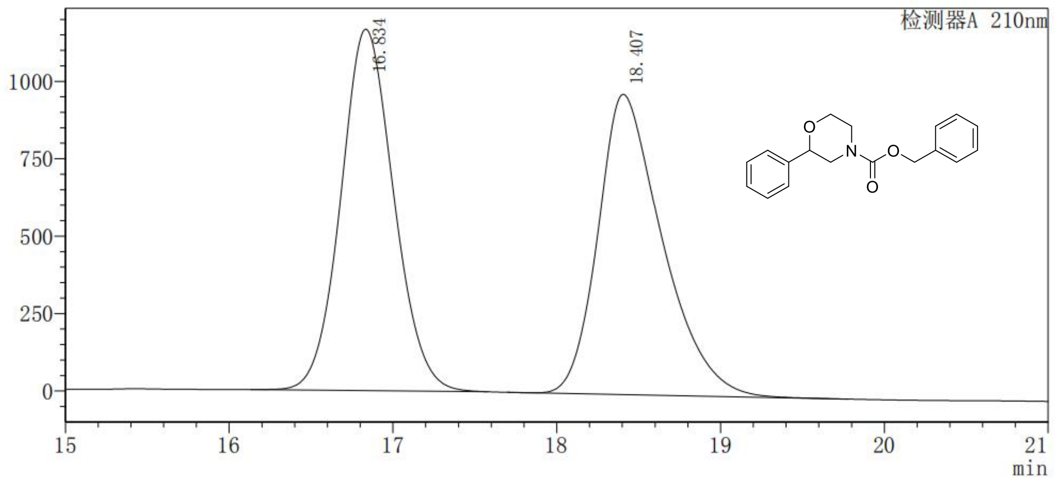

No.	RT	Area	Height	Area%
1	16.834	26255215	1167219	49.741
2	18.407	26528298	969900	50.259

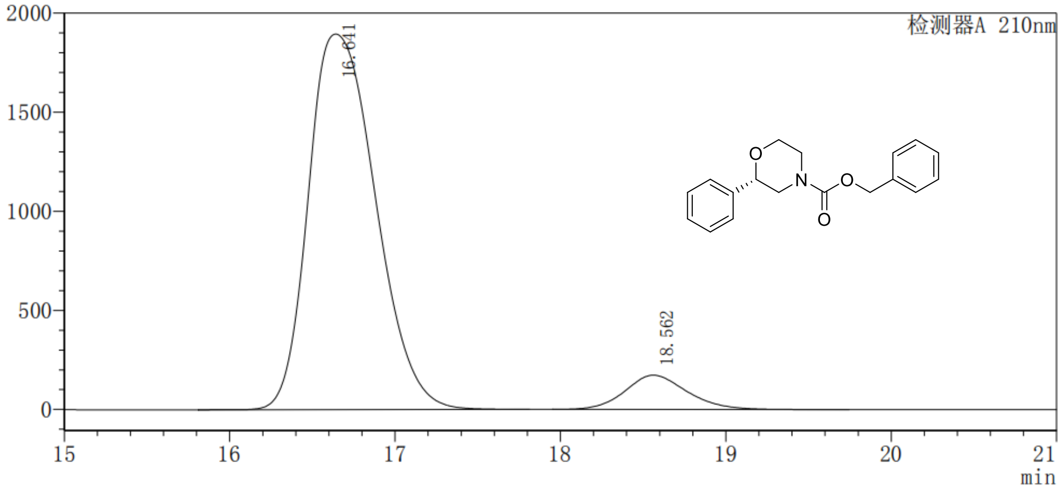

No.	RT	Area	Height	Area%
1	16.641	53539871	1894701	92.186
2	18.562	4537923	173103	7.814

84% ee. Determined by HPLC analysis using a Daicel Chiralcel IE column (25 cm × 0.46 cm), hexane/isopropanol = 95/5, 0.8 mL/min, 210 nm, t_R (major) = 16.6 min, t_R (minor) = 18.6 min.

Benzyl (*S*)-2-(3,4-dimethoxyphenyl)morpholine-4-carboxylate (2r) (using (*S*,*S*,*S*)-Tol-SKP)

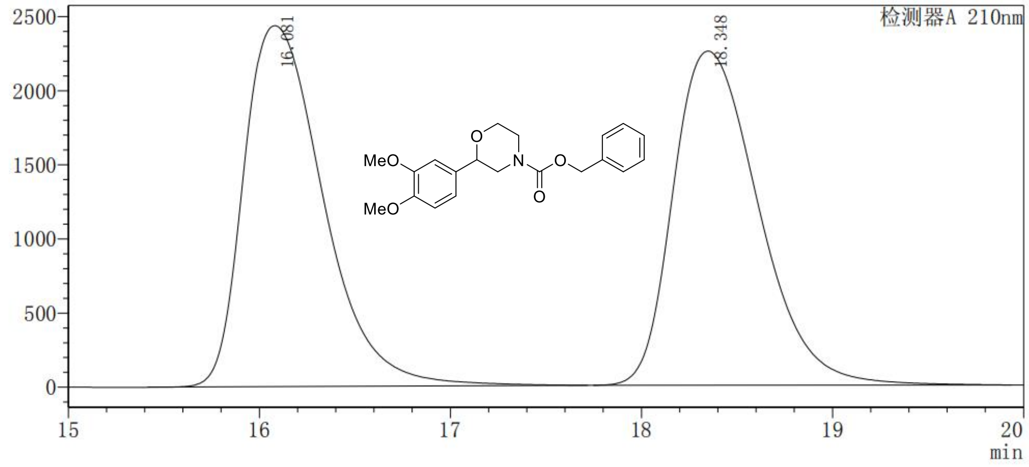

No.	RT	Area	Height	Area%
1	16.081	70684553	2434980	50.199
2	18.348	70125432	2253388	49.360

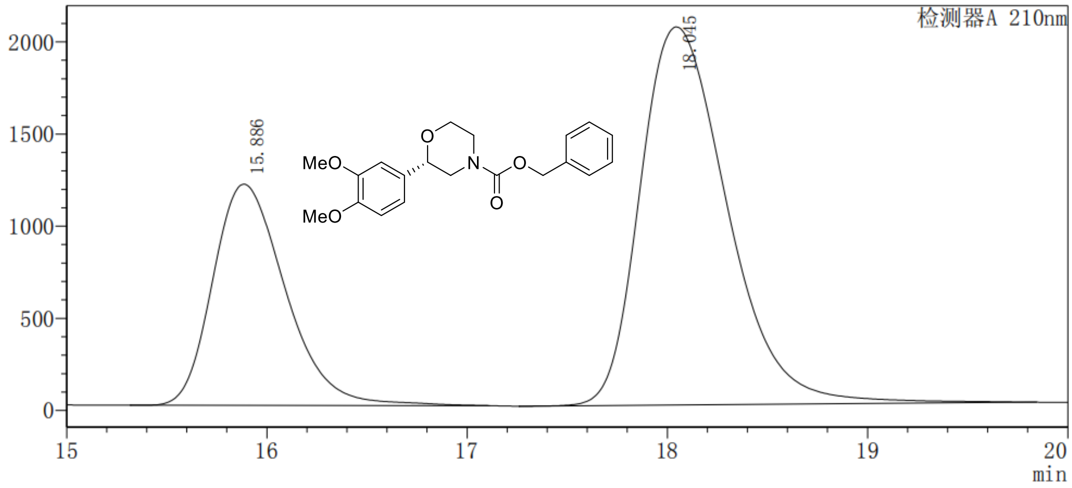

No.	RT	Area	Height	Area%
1	15.886	30243174	1200046	32.771
2	18.045	62043572	2051205	67.229

34% ee. Determined by HPLC analysis using a Daicel Chiralcel IA column (25 cm × 0.46 cm), hexane/isopropanol = 85/15, 0.8 mL/min, 210 nm, t_R (minor) = 15.9 min, t_R (major) = 18.0 min.

9. Reference

[1] D. Wang, Y. Liu, W. Zhu, H. Shen, H. Liu, L. Fu, *Chem. Lett.* **2020**, *49*, 709–712.

[2] R. W. Hungate, J. L. Chen, K. E. Starbuck, S. A. Macaluso, R. S. Rubino, *Tetrahedron Lett*. **1996**, *37*, 4113–4116.

[3] F. Lepifre, S. Clavier, P. Bouyssou, G. Coudert, *Tetrahedron Lett*. **2001**, *57*, 6969–6975.
